# Supplementary material for: Ligand-modulated nickel-catalyzed regioselective silylalkylation of alkenes
Source: Nat Commun. 2023 Nov 23;14:7670. doi: 10.1038/s41467-023-43642-z (PMC10667358; doi:10.1038/s41467-023-43642-z)
Supplement: Supplementary file 1 — Supplementary Information [file 41467_2023_43642_MOESM1_ESM.pdf]

Supplementary Information

Ligand-Modulated Nickel-Catalyzed Regioselective Silylalkylation of Alkenes

Chao Ding#, Yaoyu Ren#, Yue Yu#, Guoyin Yin*

[1]The Institute for Advanced Studies, Wuhan University, Wuhan, Hubei 430072, P. R. China.

[2]These authors contributed equally to this work.

*E-mail: yinguoyin@whu.edu.cn.

Table of Contents

1. Supplementary Methods 1
1.1 General Information 1
1.2 Reaction Condition Optimizations 2
1.3 General Procedure for the Alkylsilylation of Akenes 3
1.4 Analytical Data of Compounds 4
1.5 Extended Scope and Synthetic Applications 29
1.6 Non-reactive and Inefficient Substrates 36
1.7 Mechanism Studies 37
1.7.1 Control Experiments 37
1.7.2 Radical Clock Experiment 37
1.8 Identification of product structure 38
1.9 NMR Spectra 40
2. Supplementary References 143

1. Supplementary Methods

1.1 General Information

General information: All reactions were run under a dry argon atmosphere fitted on a glass tube or vial. All glassware was over dried at 120 ^oC for 2 h and cooled down under vacuum. Thin layer chromatography (TLC) employed glass 0.25 mm silica gel plates. Flash chromatography columns were packed with 200-300 mesh silica gel in petroleum (bp. 60-90 ^oC). GC-MS spectra were recorded on a Varian GC-MS 3900-2100 T. GC analyses were performed on an Agilent 7890B gas chromatograph with an FID detector using a J & W DB-1 column (10 m, 0.1 mm I.D.). The high-resolution mass spectra were measured on Thermo Fisher Scientific Exactive Plus (ESI). Optical rotation was determined using a Perkin Elmer 343 polarimeter. All new compounds were characterized by ^1H NMR, ^{13}C NMR, ^{19}F NMR and HRMS. The known compounds were characterized by ^1H NMR, ^{13}C NMR. ^1H, ^{13}C and ^{19}F NMR data were recorded with JNM–ECZ 400 and Bruker 600 MHz with tetramethylsilane as an internal standard. Data for ^1H, ^{13}C and ^{19}F NMR are reported as follows: chemical shift (δ ppm), multiplicity (s = singlet, d = doublet, t = triplet, q = quartet, dd = doublet of doublet, dt = doublet of triplet, dq = doublet of quartet, m = multiplet), integration, and coupling constant (Hz). All chemical shifts (δ) were reported in ppm and coupling constants (*J*) in Hz. All chemical shifts were reported relative to tetramethylsilane (0 ppm for ^1H), Chloroform-*d* (77.16 ppm for ^{13}C), respectively.

Materials: $NiBr_2$·DME (CAS: 28923-39-9) were purchased from Heowns®. LiOMe, anhydrous NMP, $PhMe_2SiBpin$ were purchased from Energy Chemical. CuI were purchased from bidepharm. Some alkenes were synthesized according to the references. Unless otherwise noted, alkene, alkyl bromide were obtained from commercial suppliers (Energy Chemical, Leyan, bidepharm, Adamas-beta® and Innochem so on) and used without further purification.

1.2 Reaction Condition Optimizations

Supplementary Table 1. Ligand Screening[a]

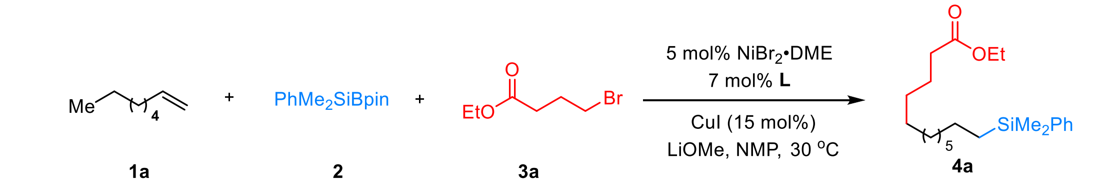

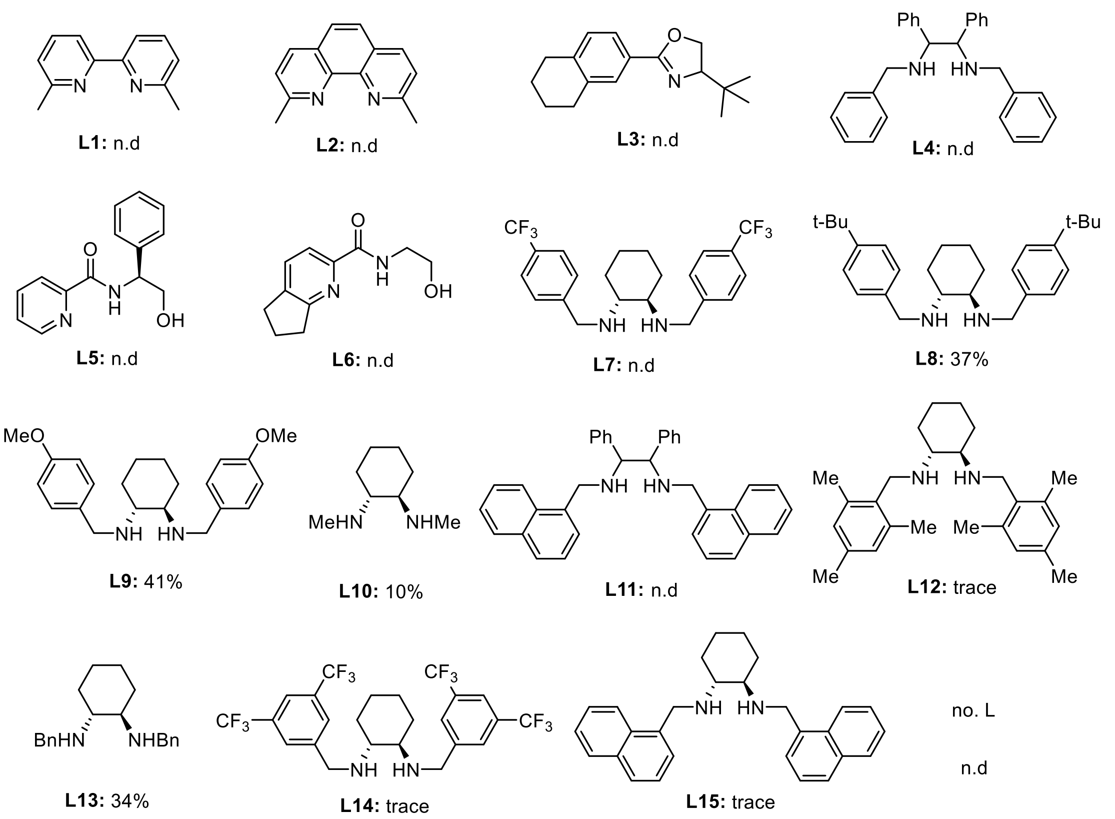

[a] Conditions: **1a** (0.4 mmol, 1.0 equiv), **2** (0.8 mmol, 2.0 equiv), **3a** (0.6 mmol, 1.5 equiv), $NiBr_2$·DME (5 mol%), **L** (7 mol%), LiOMe (0.8 mmol, 2.0 equiv), CuI (15 mol%), NMP (2 mL), 30 °C, 36 h.

Supplementary Table 2. Effect of Solvent, Temperature and Additive[a]

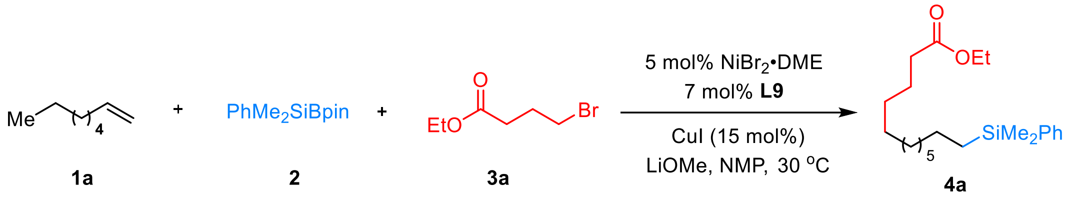

Entry	Ligand	Solvent	Temperature (°C)	Yield (%)
1	L 9	Dioxane	30 °C	n.d
2	L 9	DMF	30 °C	8%
3[b]	L 9	NMP	30 °C	n.d
4	L 9	NMP	50 °C	5%
5	L 9	NMP	40 °C	27%
6	L 13	NMP	30 °C	34%
7	L 9	NMP	30 °C	44%
8	L 9	DMA	30 °C	41%
9	L 9	NMP	5 °C	64%
**10[c]**	**L 9**	**NMP**	**5 °C**	**82%**
11[c]	L 9	NMP	10 °C	71%

[a] Conditions: **1a** (0.4 mmol, 1.0 equiv), **2** (0.8 mmol, 2.0 equiv), **3a** (0.6 mmol, 1.5 equiv), $NiBr_2$·DME (5 mol%), **L** (7 mol%), LiOMe (0.8 mmol, 2.0 equiv), CuI (15 mol%), NMP (2 mL), 30 °C, 36 h. [b] no CuI. [c] Addition of KI (0.5 equiv).

1.3 General Procedure for the Alkylsilylation of Akenes

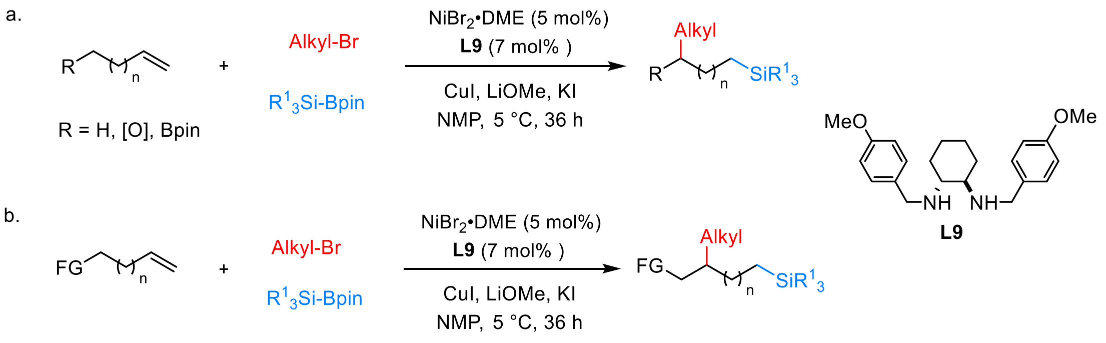

Supplementary Figure 1. Alkylsilyation of alkenes. a Alkenes contain R groups; **b** Alkenes with specific functional groups.

General Procedure: Under N_2 atmosphere in a glove box, an oven-dried 10 mL reaction tube equipped with a magnetic stir bar and sealed with a rubber stopper was used. Sequentially, $NiBr_2 \cdot DME$ (6.2 mg, 0.02 mmol, 5 mol%), **L9** (10.0 mg, 0.028 mmol, 7 mol%), LiOMe (30.4 mg, 0.8 mmol, 2.0 equiv), CuI (11.4 mg, 0.06 mmol, 15 mol%), KI (33.0 mg, 0.20 mmol, 0.5 equiv) and $PhMe_2SiBpin$ (220.8 mg, 0.8 mmol, 2.0 equiv) were added. Subsequently, anhydrous NMP (1 mL), alkenes (0.4 mmol, 1.0 equiv), alkyl bromide (0.6 mmol, 1.5 equiv) and anhydrous NMP (1 mL) were added in sequence, and the mixture was stirred at room temperature. After stirring at 5 °C for 36 h (or stirring at 30 °C for 24 h), the resulting mixture was quenched with water (2 mL) and further diluted with ethyl acetate (3 mL). The mixture was then extracted with ethyl acetate, and the combined organic layers were dried over anhydrous Na_2SO_4, filtered, and concentrated under vacuum. The crude material was separated on a silica gel column to afford the desired product. The regioisomers were determined by GC or MNR.

1.4 Analytical Data of Compounds

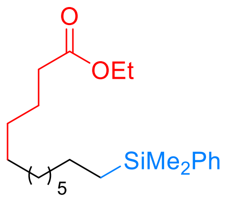

ethyl 12-(dimethyl(phenyl)silyl)dodecanoate (4a). This compound was synthesized according to general procedure. The residue was purified by column chromatography on silica gel to afford the product **4a** (119.0 mg, 82% yield) as a colorless oil; **^{1}H NMR (400 MHz, Chloroform-d)** δ 7.53 - 7.48 (m, 2H), 7.36 - 7.32 (m, 3H), 4.12 (q, *J* = 7.1 Hz, 2H), 2.28 (t, *J* = 7.6 Hz, 2H), 1.68 - 1.55 (m, 2H), 1.34 - 1.19 (m, 19H), 0.78 - 0.70 (m, 2H), 0.25 (s, 6H) ppm; **^{13}C NMR (101 MHz, Chloroform-d)** δ 174.1, 139.9, 133.7, 128.8, 127.8, 60.3, 34.5, 33.7, 29.7, 29.7, 29.6, 29.4, 29.4, 29.3, 25.1, 24.0, 15.8, 14.4, -2.9 ppm; **^{29}Si NMR (79 MHz, Chloroform-d)** δ -2.62 ppm; **IR(ATR):** $\tilde{\nu}$ 3068, 2923, 2853, 1738, 1464, 1427, 1372, 1248, 1176, 1113, 1035, 837, 777, 729, 700 cm^{-1}; **HRMS (ESI)** calculated $[M-C_6H_5]^+$ for $C_{16}H_{33}O_2Si^+$ = 285.2244, found: 285.2249.

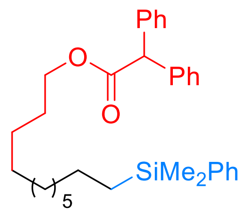

11-(dimethyl(phenyl)silyl)undecyl 2,2-diphenylacetate (4b). This compound was synthesized according to general procedure. The residue was purified by column chromatography on silica gel to afford the product **4b** (164.2 mg, 82% yield) as a colorless oil; **^{1}H NMR (400 MHz, Chloroform-d)** δ 7.57 - 7.53 (m, 2H), 7.40 - 7.35 (m, 7H), 7.36 - 7.32 (m, 4H), 7.31 - 7.26 (m, 2H), 5.09 - 5.03 (m, 1H), 4.24 - 4.13 (m, 2H), 1.72 - 1.61 (m, 2H), 1.39 - 1.23 (m, 16H), 0.83 - 0.74 (m, 2H), 0.30 (s, 6H) ppm; **^{13}C NMR (101 MHz, Chloroform-d)** δ 172.7, 139.9, 138.9, 133.7, 128.8, 128.74, 128.66, 127.8, 127.3, 65.4, 57.3, 33.7, 29.7, 29.6, 29.4, 29.3, 28.6, 25.9, 24.0, 15.8, -2.9 ppm; **^{29}Si NMR (79 MHz, Chloroform-d)** δ -2.58 ppm; **IR(ATR):** $\tilde{\nu}$ 3066, 3028, 2923, 2853, 1737, 1600, 1496, 1454, 1427,1306, 1247, 1188, 1149, 1113, 1080, 1032, 1105, 836, 776, 730, 700 cm^{-1};**HRMS (ESI)** calculated [M-C_6H_5]$^+$ for $C_{27}H_{39}O_2Si^+$ = 423.2714, found: 423.2701.

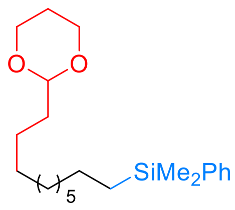

(10-(1,3-dioxan-2-yl)decyl)dimethyl(phenyl)silane (4c). This compound was synthesized according to general procedure. The residue was purified by column chromatography on silica gel to afford the product **4c** (106.0 mg, 73% yield) as a colorless oil; **^{1}H NMR (600 MHz, Chloroform-d)** δ 7.53 - 7.48 (m, 2H), 7.36 - 7.31 (m, 3H), 4.50 (t, *J* = 5.2 Hz, 1H), 4.09 (ddd, *J* = 11.9, 5.0, 1.4 Hz, 2H), 3.77 - 3.72 (m, 2H), 2.07 (dtt, *J* = 13.5, 12.5, 5.0 Hz, 1H), 1.62 - 1.53 (m, 2H), 1.40 - 1.20 (m, 17H), 0.75 - 0.71 (m, 2H), 0.25 (s, 6H) ppm; **^{13}C NMR (101 MHz, Chloroform-d)** δ 139.9, 133.7, 128.8, 127.8, 102.6, 67.0, 35.4, 33.73, 29.68, 29.63, 29.4, 26.0, 24.1, 24.0, 15.8, -2.9 ppm; **^{29}Si NMR (79 MHz, Chloroform-d)** δ -2.60 ppm; **IR(ATR):** $\tilde{\nu}$ 3068, 2922, 2851, 1467, 1427, 1404, 1377, 1247, 1146, 1114, 997, 836, 777, 729, 700 cm^{-1}; **HRMS (ESI)** calculated [M-C_6H_5]$^+$ for $C_{16}H_{33}O_2Si^+$ = 285.2244, found: 285.2234.

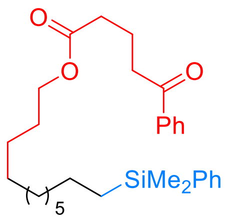

11-(dimethyl(phenyl)silyl)undecyl 5-oxo-5-phenylpentanoate (4d). This compound was synthesized according to general procedure. The residue was purified by column chromatography on silica gel to afford the product **4d** (138.4 mg, 72% yield) as a colorless oil; **^{1}H NMR (400 MHz,**

Chloroform-d) δ 7.98 - 7.96 (m, 1H), 7.96 - 7.94 (m, 1H), 7.57 - 7.48 (m, 3H), 7.47 - 7.40 (m, 2H), 7.37 - 7.31 (m, 3H), 4.07 (t, J = 6.8 Hz, 2H), 3.05 (t, J = 7.2 Hz, 2H), 2.43 (t, J = 7.2 Hz, 2H), 2.07 (p, J = 7.2 Hz, 2H), 1.64 - 1.56 (m, 2H), 1.39 - 1.16 (m, 16H), 0.77 - 0.70 (m, 2H), 0.25 (s, 6H) ppm; **^{13}C NMR (101 MHz, Chloroform-d)** δ 199.5, 173.5, 139.8, 136.9, 133.6, 133.2, 128.8, 128.7, 128.1, 127.8, 64.7, 37.6, 33.7, 33.5, 29.67, 29.63, 29.59, 29.37, 29.35, 28.7, 26.0, 23.9, 19.5, 15.8, -2.9 ppm; **^{29}Si NMR (79 MHz, Chloroform-d)** δ -2.61 ppm; **IR(ATR):** $\tilde{\nu}$ 3068, 2923, 2853, 1733, 1688, 1598, 1581, 1449, 1427, 1370, 1247, 1206, 1179, 1113, 1072, 1001, 836, 777, 732, 700 cm^{-1};**HRMS (ESI)** calculated $[M+H]^+$ for $C_{30}H_{45}O_3Si^+$ = 481.3133, found: 481.3141.

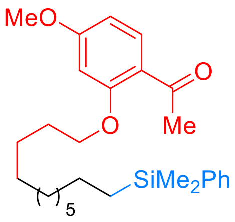

1-(2-((11-(dimethyl(phenyl)silyl)undecyl)oxy)-4-methoxyphenyl)ethan-1-one (4e). This compound was synthesized according to general procedure. The residue was purified by column chromatography on silica gel to afford the product **4e** (136.4 mg, 75% yield) as a colorless oil; **^{1}H NMR (400 MHz, Chloroform-d)** δ 7.86 - 7.80 (m, 1H), 7.54 - 7.48 (m, 2H), 7.36 - 7.31 (m, 3H), 6.54 - 6.44 (m, 1H), 6.45 - 6.41 (m, 1H), 4.02 (t, J = 6.3 Hz, 2H), 3.83 (s, 3H), 2.59 (s, 3H), 1.90 - 1.80 (m, 2H), 1.53 - 1.41 (m, 2H), 1.37 - 1.21 (m, 14H), 0.77 - 0.71 (m, 2H), 0.24 (s, 6H) ppm; **^{13}C NMR (101 MHz, Chloroform-d)** δ 198.0, 164.6, 160.8, 139.9, 133.7, 132.8, 128.8, 127.8, 121.4, 105.1, 99.0, 68.7, 55.6, 33.7, 32.2, 29.69, 29.67, 29.63, 29.45, 29.42, 29.3 26.4, 24.0, 15.8, -2.9 ppm; **^{29}Si NMR (79 MHz, Chloroform-d)** δ -2.62 ppm; **IR(ATR):** $\tilde{\nu}$ 3068, 3000, 2923, 2853, 1666, 1600, 1574, 1499, 1464, 1443, 1427, 1357, 1265, 1201, 1170, 1138, 1035, 966, 835, 776, 729, 700 cm^{-1};**HRMS (ESI)** calculated $[M+H]^+$ for $C_{28}H_{43}O_3Si^+$ = 455.2976, found: 455.2962.

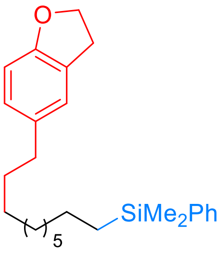

(10-(2,3-dihydrobenzofuran-5-yl)decyl)dimethyl(phenyl)silane (4f). This compound was synthesized according to general procedure. The residue was purified by column chromatography on silica gel to afford the product **4f** (86.8 mg, 55% yield) as a colorless oil; **^{1}H NMR (400 MHz, Chloroform-d)** δ 7.53 - 7.48 (m, 2H), 7.36 - 7.30 (m, 3H), 7.02 - 6.97 (m,

1H), 6.91 - 6.87 (m, 1H), 6.69 (d, *J* = 8.1 Hz, 1H), 4.53 (t, *J* = 8.7 Hz, 2H), 3.17 (t, *J* = 8.6 Hz, 2H), 2.51 (dd, *J* = 8.7, 6.8 Hz, 2H), 1.61 - 1.51 (m, 2H), 1.32 - 1.22 (m, 14H), 0.78 - 0.67 (m, 2H), 0.25 (s, 6H) ppm; **^{13}C NMR (101 MHz, Chloroform-d)** δ 158.2, 139.9, 135.1, 133.7, 128.9, 127.8, 126.9, 125.0, 108.9, 71.2, 35.5, 33.7, 32.2, 30.0, 29.8, 29.76, 29.72, 29.4, 24.0, 15.8, -2.9 ppm; **^{29}Si NMR (7 9 MHz, Chloroform-d)** δ -2.60 ppm; **IR(ATR):** $\tilde{\nu}$ 3068, 3015, 2923, 2852, 1615, 1492, 1465, 1427, 1247, 1113, 985, 945, 836, 814, 728, 700 cm^{-1};**HRMS (ESI)** calculated [M-e]$^+$ for $C_{26}H_{38}OSi^+$ = 394.2686, found: 394.2673.

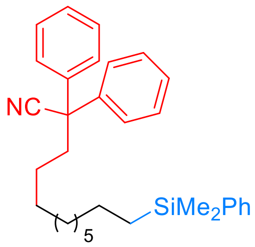

12-(dimethyl(phenyl)silyl)-2,2-diphenyldodecanenitrile (4g). This compound was synthesized according to general procedure. The residue was purified by column chromatography on silica gel to afford the product **4g** (93.6 mg, 50% yield) as a colorless oil; **^{1}H NMR (400 MHz, Chloroform-d)** δ 7.52 - 7.48 (m, 2H), 7.41 - 7.31 (m, 11H), 7.30 - 7.22 (m, 2H), 2.39 - 2.28 (m, 2H), 1.48 - 1.37 (m, 2H), 1.37 - 1.16 (m, 14H), 0.76 - 0.69 (m, 2H), 0.25 (s, 6H) ppm; **^{13}C NMR (101 MHz, Chloroform-d)** δ 140.5, 139.9, 133.7, 129.0, 128.8, 127.9, 127.8, 127.0, 122.6, 51.9, 39.8, 33.7, 29.64, 29.60, 29.39, 29.35, 25.8, 24.0, 15.8, -2.9 ppm; **^{29}Si NMR (79 MHz, Chloroform-d)** δ -2.59 ppm; **IR(ATR):** $\tilde{\nu}$ 3066, 2923, 2852, 2235, 1599, 1494, 1449, 1427, 1247, 1113, 1033, 836, 752, 730, 700 cm^{-1}; **HRMS (ESI)** calculated [M-C_6H_5]$^+$ for $C_{26}H_{36}NSi^+$ =390.2612, found: 390.2604.

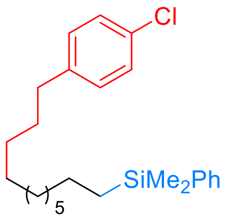

(11-(4-chlorophenyl)-undecyl)-dimethyl-(phenyl)-silane (4h). This compound was synthesized according to general procedure. The residue was purified by column chromatography on silica gel to afford the product **4h** (103.7 mg, 65% yield) as a colorless oil; **^{1}H NMR (400 MHz, Chloroform-d)** δ 7.53 - 7.47 (m, 2H), 7.36 - 7.31 (m, 3H), 7.24 - 7.20 (m, 2H), 7.11 - 7.05 (m, 2H), 2.55 (dd, *J* = 8.7, 6.8 Hz, 2H), 1.60 - 1.54 (m, 2H), 1.32 - 1.15 (m, 16H), 0.78 - 0.69 (m, 2H), 0.24 (s, 6H) ppm; **^{13}C NMR (101 MHz, Chloroform-d)** δ 141.5, 139.9, 133.7, 131.4, 129.9, 128.9, 128.4, 127.8, 35.4, 33.8, 31.5, 29.8, 29.71, 29.69, 29.6, 29.4, 29.3, 24.0, 15.8, -2.9 ppm; **^{29}Si NMR (79 MHz, Chloroform-d)** δ -2.59 ppm; **IR(ATR):** $\tilde{\nu}$ 3068, 2923, 2853,

1492, 1464, 1427, 1247, 1113, 1093, 1015, 836, 728, 700 cm^{-1}; **HRMS (ESI)** calculated $[M+Na]^+$ for $C_{15}H_{31}OSiNa^+$ = 423.2245, found: 423.2237.

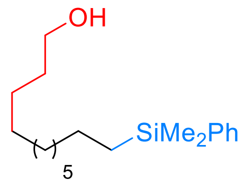

11-(dimethyl(phenyl)silyl)undecan-1-ol (4i). This compound was synthesized according to general procedure. The residue was purified by column chromatography on silica gel to afford the product **4i** (113.8 mg, 93% yield) as a colorless oil; **^{1}H NMR (600 MHz, Chloroform-*d*)** δ 7.53 - 7.48 (m, 2H), 7.37 - 7.31 (m, 3H), 3.62 (t, J = 6.7 Hz, 2H), 1.58 - 1.53 (m, 2H), 1.47 - 1.37 (m, 1H), 1.34 - 1.23 (m, 16H), 0.75 - 0.71 (m, 2H), 0.25 (s, 6H); **^{13}C NMR (151 MHz, Chloroform-*d*)** δ 139.9, 133.7, 128.8, 127.8, 63.2, 33.7, 32.9, 29.73, 29.72, 29.69, 29.56, 29.4, 25.9, 24.0, 15.8, -2.9; **^{29}Si NMR (119 MHz, Chloroform-*d*)** δ -3.12; **IR(ATR):** $\tilde{\nu}$ 3334, 3068, 2923, 2853, 1465, 1427, 1247, 1113, 1056, 836, 777, 728, 700 cm^{-1}; **HRMS (ESI)** calculated $[M-C_6H_5]^+$ for $C_{13}H_{29}OSi^+$ = 229.1982, found: 229.1974.

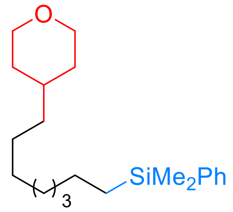

dimethyl(phenyl)(8-(tetrahydro-2H-pyran-4-yl)octyl)silane (4j). This compound was synthesized according to general procedure. The residue was purified by column chromatography on silica gel to afford the product **4j** (92.4 mg, 69% yield) as a colorless oil; **^{1}H NMR (400 MHz, Chloroform-*d*)** δ 7.53 - 7.48 (m, 2H), 7.35 (dp, *J* = 5.1, 1.8 Hz, 3H), 3.94 (dtd, *J* = 11.6, 2.5, 1.2 Hz, 2H), 3.36 (td, *J* = 11.8, 2.2 Hz, 2H), 1.6 (ddq, J = 12.7, 3.4, 1.9 Hz, 1H), 1.44 (dddt, J = 13.1, 9.6, 6.1, 3.1 Hz, 1H), 1.32 - 1.20 (m, 16H), 0.77 - 0.71 (m, 2H), 0.25 (s, 6H) ppm; **^{13}C NMR (101 MHz, Chloroform-*d*)** δ 139.9, 133.7, 128.9, 127.8, 68.3, 37.1, 35.1, 33.7, 33.4, 30.0, 29.7, 29.4, 26.5, 24.0, 15.8, -2.9 ppm; **^{29}Si NMR (79 MHz, Chloroform-d)** δ -2.62 ppm; **IR(ATR):** $\tilde{\nu}$ 3068, 2923, 2852, 1464, 1429, 1247, 1159, 1113, 1099, 1013, 981, 836, 778, 728, 700 cm^{-1};**HRMS (ESI)** calculated $[M-C_6H_5]^+$ for $C_{15}H_{31}OSi^+$ = 255.2139, found: 255.2137.

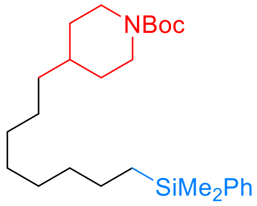

tert-butyl 4-(8-(dimethyl(phenyl)silyl)octyl)piperidine-1-carboxylate (4k). This compound was synthesized according to general procedure. The residue was purified by column chromatography on silica gel to afford the product **4k** (97.9 mg, 57% yield) as a colorless oil; **^{1}H NMR (400 MHz, Chloroform-d)** δ 7.53 - 7.48 (m, 2H), 7.37 - 7.30 (m, 3H), 4.38 - 3.78 (m, 2H), 2.66 (t, J = 12.8 Hz, 2H), 1.62 - 1.61 (m, 1H), 1.45 (s, 9H), 1.33 - 1.16 (m, 16H), 1.05 (qd, J = 12.5, 4.4 Hz, 2H), 0.75 - 0.70 (m, 2H), 0.25 (s, 6H) ppm; **^{13}C NMR (151 MHz, Chloroform-d)** δ 155.1, 139.9, 133.7, 128.9, 127.8, 79.3, 44.6, 43.8, 36.7, 36.1, 33.7, 32.4, 30.0, 29.7, 29.4, 28.6, 26.7, 24.0, 15.8, -2.9 ppm; **^{29}Si NMR (79 MHz, Chloroform-d)** δ -2.62 ppm; **IR(ATR):** $\tilde{\nu}$ 3068, 2923, 2852, cm^{-1}; **IR(ATR):** $\tilde{\nu}$ 3068, 2923, 2852, 1695, 1464, 1425, 1365, 1278, 1247, 1174, 1113, 967, 836, 770, 729, 700 cm^{-1}; **HRMS (ESI)** calculated $[M+H]^+$ for $C_{26}H_{46}O_2NSi^+$ = 432.3292,found: 432.3277.

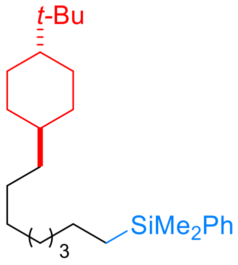

***trans*-(6-(4-(tert-butyl)cyclohexyl)hexyl)dimethyl(phenyl)silane (4l).** This compound was synthesized according to general procedure. The residue was purified by column chromatography on silica gel to afford the product **4l** (91.0 mg, 59% yield, rr > 20:1) as a colorless oil; **^{1}H NMR (400 MHz, Chloroform-d)** δ 7.53 - 7.47 (m, 2H), 7.37 - 7.33 (m, 3H), 1.81 - 1.67 (m, 3H), 1.35 - 1.19 (m, 13H), 1.17 - 1.03 (m, 3H), 0.99 - 0.90 (m, 3H), 0.89 - 0.85 (m, 1H), 0.83 (s, 9H), 0.81 - 0.77 (m, 1H), 0.76 - 0.71 (m, 2H), 0.25 (s, 6H) ppm; **^{13}C NMR (101 MHz, Chloroform-d)** δ 140.0, 133.7, 128.9, 127.8, 48.5, 37.9, 37.6, 34.0, 33.8, 32.6, 30.2, 29.8, 29.5, 27.8, 27.5, 27.1, 24.0, 15.8, -2.9 ppm; **^{29}Si NMR (79 MHz, Chloroform-d)** δ -2.61 ppm; **IR(ATR):** $\tilde{\nu}$ 3069, 2921, 2852, 1466, 1427, 1384, 1247, 1113, 835, 778, 727, 700 cm^{-1}; **HRMS (ESI)** calculated $[M+H]^+$ for $C_{26}H_{47}Si^+$ = 387.3365, found: 387.3368.

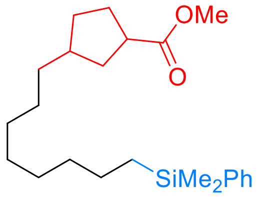

methyl 3-(8-(dimethyl(phenyl)silyl)octyl)cyclopentane-1-carboxylate (4m). This compound was synthesized according to general procedure. The residue was purified by column chromatography on silica gel to afford the product **4m** (81.1 mg, 54% yield, dr = 1.5:1) as

a colorless oil; **^{1}H NMR (400 MHz, Chloroform-d)** δ 7.54 - 7.47 (m, 2H), 7.36 - 7.31 (m, 3H), 3.66 (s, 3H), 2.88 - 2.67 (m, 1H), 2.13 - 1.71 (m, 5H), 1.50 - 1.36 (m,1H), 1.35 - 1.18 (m, 14H), 1.12 (m, 1H), 0.78 - 0.69 (m, 2H), 0.25 (s, 6H) ppm; **^{13}C NMR (101 MHz, Chloroform-d)** δ 177.6, 177.4, 139.9, 133.7, 128.8, 127.8, 51.7, 51.7, 43.7, 42.9, 40.8, 39.7, 37.2, 36.2, 35.9, 35.8, 33.7, 33.1, 32.1, 30.0, 29.9, 29.7, 29.4, 29.0, 28.8, 28.7, 24.0, 15.8, -2.9 ppm; **^{29}Si NMR (79 MHz, Chloroform-d)** δ -2.63 ppm; **IR(ATR):** $\tilde{\nu}$ 3069, 2922, 2852, 1737, 1435, 1427, 1362, 1248, 1196, 1170, 1113, 836, 778, 729, 700 cm^{-1}; **HRMS (ESI)** calculated $[M\text{-}C_6H_5]^+$ for $C_{17}H_{33}O_2Si^+$ = 297.2244, found: 297.2235.

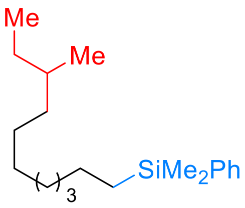

dimethyl(9-methylundecyl)(phenyl)silane (4n). This compound was synthesized according to general procedure. The residue was purified by column chromatography on silica gel to afford the product **4n** (65.3 mg, 54% yield) as a colorless oil; **^{1}H NMR (400 MHz, Chloroform-d)** δ 7.56 - 7.52 (m, 2H), 7.40 - 7.35 (m, 3H), 1.40 - 1.22 (m, 15H), 1.19 - 1.05 (m, 2H), 0.94 - 0.84 (m, 6H), 0.80 - 0.74 (m, 2H), 0.28 (s, 6H) ppm; **^{13}C NMR (101 MHz, Chloroform-d)** δ 139.9, 133.7, 128.9, 127.8, 36.8, 34.6, 33.8, 30.2, 29.8, 29.7, 29.5, 27.3, 24.0, 19.4, 15.8, 11.6, -2.9 ppm; **^{29}Si NMR (79 MHz, Chloroform-d)** δ -2.59 ppm; **IR(ATR):** $\tilde{\nu}$ 3069, 2957, 2923, 2853, 1463, 1427, 1378, 1248, 1113, 846, 777, 727, 700 cm^{-1}; **HRMS (ESI)** calculated $[M+H]^+$ for $C_{20}H_{37}Si^+$ = 305.2659, found: 305.2637.

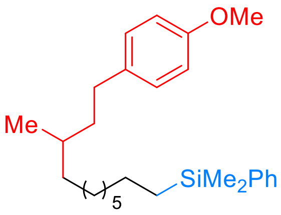

(11-(4-methoxyphenyl)-9-methylundecyl)dimethyl(phenyl)silane (4o). This compound was synthesized according to general procedure. The residue was purified by column chromatography on silica gel to afford the product **4o** (91.0 mg, 55% yield) as a colorless oil; **^{1}H NMR (600 MHz, Chloroform-d)** δ 7.57 - 7.45 (m, 2H), 7.37 - 7.31 (m, 3H), 7.13 - 7.08 (m, 2H), 6.83 - 6.80 (m, 2H), 3.77 (s, 3H), 2.58 (ddd, J = 13.5, 10.5, 5.2 Hz, 1H), 2.49 (ddd, J = 13.6, 10.0, 6.0 Hz, 1H), 1.61 - 1.55 (m, 1H), 1.46 - 1.34 (m, 2H), 1.32 - 1.20 (m, 13H), 1.15 - 1.10 (m, 1H), 0.90 (d, J = 6.4 Hz, 3H), 0.76 - 0.70 (m, 2H), 0.25 (s, 6H) ppm; **^{13}C NMR (151 MHz, Chloroform-d)** δ 157.7, 139.9, 135.4, 133.7, 129.3, 128.9, 127.8, 113.8, 55.4, 39.4, 37.1,

33.8, 32.7, 32.6, 30.2, 29.8, 29.5, 27.1, 24.0, 19.8, 15.8, -2.9 ppm; **^{29}Si NMR (79 MHz, Chloroform-d)** δ -2.60 ppm; **IR(ATR):** $\tilde{\nu}$ 3068, 2998, 2953, 2923, 2852, 1613, 1512, 1464, 1427, 1300, 1246, 1176, 1113, 1040, 835, 779, 729, 700 cm^{-1}; **HRMS (ESI)** calculated [M-C_6H_5]$^+$ for $C_{21}H_{37}OSi^+$ = 333.2608, found: 333.2607.

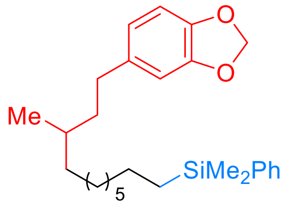

(11-(benzo[d][1,3]dioxol-5-yl)-9-methylundecyl)dimethyl(phenyl)silane (4p). This compound was synthesized according to general procedure using L8 instead of L9. The residue was purified by column chromatography on silica gel to afford the product **4p** (81.5 mg, 48% yield) as a colorless oil; **^{1}H NMR (400 MHz, Chloroform-d)** δ 7.54 - 7.47 (m, 2H), 7.36 - 7.31 (m, 3H), 6.74 - 6.59 (m, 3H), 5.90 (s, 2H), 2.63 - 2.41 (m, 2H), 1.60 - 1.56 (m, 1H), 1.46 - 1.36 (m, 2H), 1.35 - 1.19 (m, 13H), 1.16 - 1.07 (m, 1H), 0.90 (d, J = 6.3 Hz, 3H), 0.78 - 0.71 (m, 2H), 0.25 (s, 6H) ppm; **^{13}C NMR (101 MHz, Chloroform-d)** δ 147.6, 145.5, 139.9, 137.3, 133.7, 128.9, 127.8, 121.1, 109.0, 108.2, 100.8, 39.4, 37.1, 33.8, 33.4, 32.5, 30.2, 29.8, 29.5, 27.1, 24.0, 19.7, 15.8, -2.9 ppm; **^{29}Si NMR (79 MHz, Chloroform-d)** δ -2.60 ppm; **IR(ATR):** $\tilde{\nu}$ 3068, 2955, 2923, 2853, 1504, 1489, 1442, 1376, 1246, 1188, 1113, 1042, 940, 836, 811, 777, 729, 700 cm^{-1}; **HRMS (ESI)** calculated [M-e]$^+$ for $C_{27}H_{40}O_2Si^+$ = 424.2792, found: 424.2777.

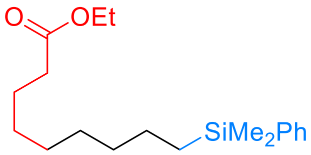

ethyl 9-(dimethyl(phenyl)silyl)nonanoate (4q). This compound was synthesized according to general procedure. The residue was purified by column chromatography on silica gel to afford the product **4q** (112.4 mg, 88 % yield, rr > 20:1) as a colorless oil; **^{1}H NMR (400 MHz, Chloroform-d)**δ 7.54 - 7.46 (m, 2H), 7.37 - 7.31 (m, 3H), 4.12 (q, J = 7.1 Hz, 2H), 2.28 (t, J = 7.6 Hz, 2H), 1.65 - 1.56 (m, 2H), 1.33 - 1.24 (m, 13H), 0.78 - 0.70 (m, 2H), 0.25 (s, 3H) ppm; **^{13}C NMR (101 MHz, Chloroform-d)** δ 174.0, 139.8, 133.7, 128.8, 127.8, 60.3, 34.5, 33.6, 29.28, 29.26, 29.18, 25.1, 23.9, 15.8, 14.4, -2.9 ppm; **^{29}Si NMR (79 MHz, Chloroform-d)** δ -2.62 ppm; **IR(ATR):** $\tilde{\nu}$ 3069, 2923, 2854, 1737, 1464, 1426, 1372, 1248, 1178, 1114, 1113,

1034, 837, 775, 730, 701 cm^{-1}; **HRMS (ESI)** calculated $[M-C_6H_5]^+$ for $C_{13}H_{27}O_2Si^+$ = 243.1775, found: 243.1770.

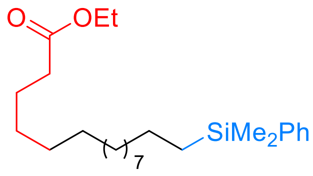

ethyl 15-(dimethyl(phenyl)silyl)pentadecanoate (4r). This compound was synthesized according to general procedure. The residue was purified by column chromatography on silica gel to afford the product **4r** (151.3 mg, 93% yield, rr > 20:1) as a colorless oil; **^{1}H NMR (400 MHz, Chloroform-d)** δ 7.55 - 7.49 (m, 2H), 7.39 - 7.32 (m, 3H), 4.14 (q, *J* = 7.1 Hz, 2H), 2.30 (t, *J* = 7.5 Hz, 2H), 1.70 - 1.58 (m, 2H), 1.37 - 1.18 (m, 25H), 0.78 - 0.72 (m, 2H), 0.27 (s, 6H) ppm; **^{13}C NMR (101 MHz, Chloroform-d)** δ 173.98, 139.82, 133.64, 128.81, 127.78, 60.22, 34.48, 33.73, 29.81, 29.76, 29.71, 29.58, 29.42, 29.39, 29.26, 25.09, 23.97, 15.79, 14.36, -2.91 ppm; **^{29}Si NMR (79 MHz, Chloroform-d)** δ -2.62 ppm; **IR(ATR):** $\tilde{\nu}$ 3069, 2923, 2853, 1738, 1485, 1427, 1372, 1248, 1178, 1113, 1034, 836, 776, 729, 700 cm^{-1}; **HRMS (ESI)** calculated $[M+NH_4]^+$ for $C_{25}H_{48}O_2NSi^+$ = 422.3449, found: 422.3445.

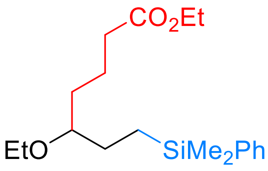

ethyl 7-(dimethyl(phenyl)silyl)-5-ethoxyheptanoate (4s). This compound was synthesized according to general procedure. The residue was purified by column chromatography on silica gel to afford the product **4s** (72.8 mg, 54% yield, rr > 20:1) as a colorless oil; **^{1}H NMR (400 MHz, Chloroform-d)** δ 7.54 - 7.47 (m, 2H), 7.36 - 7.33 (m, 3H), 4.12 (q, *J* = 7.1 Hz, 2H), 3.50 - 3.33 (m, 2H), 3.21 - 3.11 (m, 1H), 2.34 - 2.22 (m, 2H), 1.76 - 1.65 (m, 1H), 1.63 - 1.55 (m, 1H), 1.51 - 1.43 (m, 4H), 1.25 (t, *J* = 7.1 Hz, 3H), 1.16 (t, *J* = 7.0 Hz, 3H), 0.85 - 0.61 (m, 2H), 0.26 (s, 6H) ppm; **^{13}C NMR (151 MHz, Chloroform-d)** δ 173.9, 139.4, 133.7, 129.0, 127.9, 81.0, 64.3, 60.3, 34.6, 33.0, 27.8, 21.1, 15.8, 14.4, 10.7, -3.0 -3.1 ppm; **^{29}Si NMR (79 MHz, Chloroform-d)** δ -1.87 ppm; **IR(ATR):** $\tilde{\nu}$ 3069, 2955, 2928, 2870, 1737, 1427, 1371, 1346, 1248, 1179, 1114, 1098, 1034, 838, 776, 730, 701 cm^{-1}; **HRMS (ESI)** calculated $[M-C_6H_5]^+$ for $C_{13}H_{27}O_3Si^+$ = 259.1724, found: 259.1718.

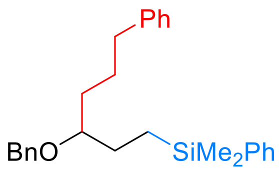

(3-(benzyloxy)-6-phenylhexyl)dimethyl(phenyl)silane (4t). This compound was synthesized according to general procedure at 30 °C for 36 h. The residue was purified by column chromatography on silica gel to afford the product **4t** (95.2 mg, 59% yield, rr > 20:1) as a colorless oil; **^{1}H NMR (400 MHz, Chloroform-d)** δ 7.52 - 7.46 (m, 2H), 7.35 - 7.21 (m, 10H), 7.20 - 7.12 (m, 3H), 4.48 - 4.35 (m, 2H), 3.38 - 3.27 (m, 1H), 2.58 (t, *J* = 7.2 Hz, 2H), 1.79 - 1.67 (m, 1H), 1.64 - 1.41 (m, 5H), 0.84 - 0.66 (m, 2H), 0.25 (d, *J* = 1.6 Hz, 6H) ppm; **^{13}C NMR (101 MHz, Chloroform-*d*)** δ 142.7, 139.4, 139.2, 133.7, 129.0, 128.6, 128.44, 128.40, 127.89, 127.87, 127.5, 125.8, 80.8, 70.9, 36.2, 32.9, 27.5, 27.3, 10.5, -2.96, -3.04 ppm; **^{29}Si NMR (79 MHz, Chloroform-d)** δ -1.81 ppm; **IR(ATR):** $\tilde{\nu}$ 3067, 3026, 2929, 2859, 1603, 1496, 1454, 1427, 1347, 1248, 1172, 1113, 1093, 1068, 837, 815, 775, 732, 699 cm^{-1}; **HRMS (ESI)** calculated $[M-C_6H_5]^+$ for $C_{21}H_{29}OSi^+$ = 325.1982, found: 325.1973.

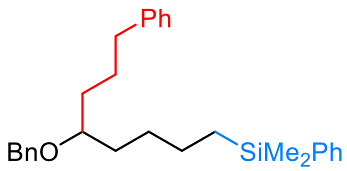

(5-(benzyloxy)-8-phenyloctyl)dimethyl(phenyl)silane (4u). This compound was synthesized according to general procedure at 30 °C for 36 h. The residue was purified by column chromatography on silica gel to afford the product **4u** (50.0 mg, 29% yield, rr > 20:1) as a colorless oil; **^{1}H NMR (600 MHz, Chloroform-*d*)** δ 7.42 (dt, *J* = 4.9, 2.7 Hz, 2H), 7.29 - 7.22 (m, 7H), 7.21 - 7.16 (m, 3H), 7.13 - 7.07 (m, 3H), 4.37 (s, 2H), 3.30 - 3.24 (m, 1H), 2.51 (t, *J* = 7.7 Hz, 2H), 1.69 - 1.61 (m, 1H), 1.60 - 1.53 (m, -H), 1.52 - 1.42 (m, 3H), 1.41 - 1.36 (m, 1H), 1.34 - 1.27 (m, 1H), 1.27 - 1.20 (m, 3H), 0.66 (t, *J* = 7.5 Hz, 2H), 0.17 (s, 6H) ppm; **^{13}C NMR (151 MHz, Chloroform-*d*)** δ 142.7, 139.7, 139.2, 133.7, 128.9, 128.5, 128.43, 128.39, 127.9, 127.8, 127.5, 125.8, 78.9, 70.9, 36.2, 33.59, 33.56, 29.4, 27.2, 24.2, 15.9, -2.9 ppm;**^{29}Si NMR (119 MHz, Chloroform-*d*)** δ -3.11 ppm;**HRMS (ESI)**calculated $[M+NH_4]^+$ for $C_{29}H_{42}ONSi^+$ =448.3030, found: 448.3036.

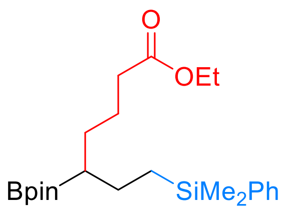

ethyl 7-(dimethyl-(phenyl)-silyl)-5-(4,4,5,5-tetramethyl-1,3,2-dioxaborolan-2-yl)-heptanoate (4w). This compound was synthesized according to general procedure. The residue was purified by column chromatography on silica gel to afford the product **4w** (116.4 mg, 70% yield, rr > 20:1) as a colorless oil; **^{1}H NMR (400 MHz, Chloroform-*d*)** δ 7.52 - 7.47 (m, 2H), 7.35 - 7.31 (m, 3H), 4.10 (q, *J* = 7.1 Hz, 2H), 2.26 (dd, *J* = 8.2, 6.9 Hz, 2H), 1.59 (dq, J = 9.5, 7.5 Hz, 2H), 1.49 - 1.32 (m, 4H), 1.27 - 1.20 (m, 15H), 0.97 (tt, *J* = 8.6, 5.9 Hz, 1H), 0.77 - 0.71 (m, 2H), 0.24 (d, *J* = 0.8 Hz, 6H) ppm; **^{13}C NMR (101 MHz, Chloroform-*d*)** δ 173.9, 139.7, 133.7, 128.8, 127.8, 83.0, 60.2, 34.8, 30.6, 25.3, 25.0, 24.9, 24.7, 15.1, 14.4, -2.9, -3.0 ppm; **^{11}B NMR (128 MHz, Chloroform-*d*)** δ 33.62 ppm; **^{29}Si NMR (79 MHz, Chloroform-d)** δ -2.60 ppm; **IR(ATR):** $\tilde{\nu}$ 3069, 2978, 2954, 2926, 2867, 1737, 1460, 1427, 1387, 1371, 1314, 1247, 1166, 1145, 1113, 1031, 968, 865, 837, 815, 777, 730, 701 cm^{-1}; **HRMS (ESI)** calculated $[M+H]^+$ for $C_{23}H_{40}O_4BSi^+$ = 419.2783, found: 419.2770.

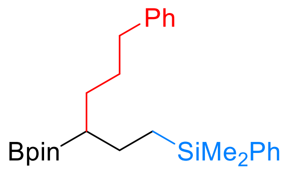

dimethyl-(phenyl)-(6-phenyl-3-(4,4,5,5-tetramethyl-1,3,2-dioxa-borolan-2-yl)-hexyl)-silane (**4x**). This compound was synthesized according to general procedure. The residue was purified by column chromatography on silica gel to afford the product **4x** (128.2 mg, 76% yield, rr > 20:1) as a colorless oil; **^{1}H NMR (400 MHz, Chloroform-*d*)** δ 7.51 - 7.48 (m, 2H), 7.34 - 7.31 (m, 3H), 7.28 - 7.23 (m, 2H), 7.17 - 7.13 (m, 3H), 2.57 (t, *J* = 7.7 Hz, 2H), 1.62 - 1.54 (m, 2H), 1.49 - 1.42 (m, 2H), 1.41 - 1.34 (m, 2H), 1.22 (d, *J* = 0.8 Hz, 12H), 1.00 (tt, *J* = 8.6, 5.8 Hz, 1H), 0.78 - 0.68 (m, 2H), 0.23 (d, *J* = 0.8 Hz, 6H) ppm; **^{13}C NMR (101 MHz, Chloroform-*d*)** δ 143.0, 139.8, 133.7, 128.8, 128.5, 128.3, 127.8, 125.6, 83.0, 36.4, 31.2, 30.9, 25.4, 25.0, 25.0, 15.2, -2.9, -2.9 ppm; **^{11}B NMR (193 MHz, Chloroform-*d*)** δ 34.82 ppm; **^{29}Si NMR (119 MHz, Chloroform-d)** δ -3.13 ppm; **IR(ATR):** $\tilde{\nu}$ 3068, 3025, 2977, 2924, 2855, 1604, 1454, 1427, 1410, 1387, 1379, 1371, 1313, 1247, 1166, 1144, 1113, 968, 867, 837, 815, 776, 729, 699 cm^{-1}; **HRMS (ESI)** calculated $[M-C_6H_5]^+$ for $C_{20}H_{34}O_2BSi^+$ = 345.2416, found: 345.2405.

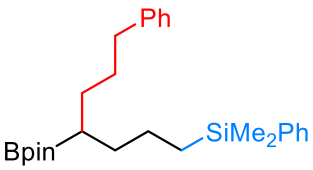

dimethyl(phenyl)(7-phenyl-4-(4,4,5,5-tetramethyl-1,3,2-dioxaborolan-2-yl)-heptyl)-silane (**4y**). This compound was synthesized according to general procedure. The residue was purified by column chromatography on silica gel to afford the product **4y** (97.8 mg, 56% yield, rr > 20:1) as a colorless oil; **^{1}H NMR (600 MHz, Chloroform-*d*)** δ 7.51 - 7.47 (m, 2H), 7.35 - 7.30 (m, 3H), 7.27 - 7.23 (m, 2H), 7.17 - 7.13 (m, 3H), 2.58 (td, *J* = 7.6, 2.4 Hz, 2H), 1.62 - 1.57 (m, 2H), 1.47 - 1.40 (m, 2H), 1.39 - 1.33 (m, 2H), 1.32 - 1.27 (m, 2H), 1.18 (s, 12H), 1.00 (tt, *J* = 9.1, 5.6 Hz, 1H), 0.79 - 0.66 (m, 2H), 0.23 (s, 6H) ppm; **^{13}C NMR (151 MHz, Chloroform-*d*)** δ 143.1, 139.8, 133.7, 128.8, 128.5, 128.3, 127.8, 125.6, 83.0, 36.3, 35.7, 31.3, 24.9, 23.7, 16.2, -2.8, -2.9 ppm; **^{29}Si NMR (119 MHz, Chloroform-d)** δ -3.10 ppm; **HRMS (ESI)** calculated $[M+NH_4]^+$ for $C_{27}H_{45}O_2NBSi^+$ = 454.3307,found: 454.3319.

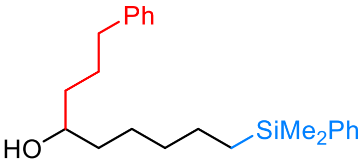

9-(dimethyl(phenyl)silyl)-1-phenylnonan-4-ol (**4z**). This compound was synthesized according to general procedure with oxidation[4]. The residue was purified by column chromatography on silica gel to afford the product **4z** (79.4 mg, 56% yield, rr > 20:1) as a colorless oil; **^{1}H NMR (600 MHz, Chloroform-*d*)** δ 7.52 - 7.47 (m, 2H), 7.35 - 7.32 (m, 3H), 7.29 - 7.25 (m, 2H), 7.20 - 7.14 (m, 3H), 3.62 - 3.52 (m, 1H), 2.67 - 2.56 (m, 2H), 1.82 - 1.71 (m, 1H), 1.69 - 1.58 (m, 1H), 1.52 - 1.42 (m, 3H), 1.41 - 1.35 (m, 3H), 1.34 - 1.31 (m, 2H), 1.29 - 1.24 (m, 2H), 0.76 - 0.70 (m, 2H), 0.25 (s, 6H) ppm; **^{13}C NMR (151 MHz, Chloroform-*d*)** δ 142.5, 139.8, 133.7, 128.9, 128.5, 128.4, 127.8, 125.8, 71.9, 37.5, 37.1, 36.0, 33.7, 27.6, 25.4, 23.9, 15.8, -2.9 ppm; **^{29}Si NMR (119 MHz, Chloroform-*d*)** δ -3.11 ppm; **HRMS (ESI)** calculated $[M+NH_4]^+$ for $C_{23}H_{38}ONSi^+$ = 372.2717, found:372.2726.

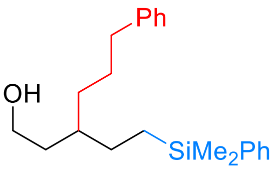

3-(2-(dimethyl(phenyl)silyl)ethyl)-6-phenylhexan-1-ol (**4aa**). This compound was synthesized according to general procedure. The residue was purified by column chromatography on silica gel to afford the product **4aa** (102.3 mg, 75% yield) as a colorless oil; **^{1}H NMR (600 MHz, Chloroform-*d*)** δ 7.50 - 7.47 (m, 2H), 7.35 - 7.32 (m, 3H), 7.28 - 7.24 (m, 2H), 7.19 -7.12 (m,

3H), 3.56 (t, J = 7.0 Hz, 2H), 2.55 (dd, J = 8.7, 6.8 Hz, 2H), 1.54 (ddd, J = 15.8, 8.9, 7.4 Hz, 2H), 1.48 (dq, J = 16.7, 6.7 Hz, 2H), 1.43 - 1.39 (m, 1H), 1.36 - 1.32 (m, 1H), 1.32 - 1.26 (m, 4H), 0.68 - 0.63 (m, 2H), 0.24 (s, 6H) ppm; **^{13}C NMR (101 MHz, Chloroform-*d*)** δ 142.8, 139.5, 133.6, 128.9, 128.5, 128.4, 127.8, 125.7, 61.2, 36.5, 36.3, 32.7, 28.5, 27.1, 11.5, -3.0 ppm; **^{29}Si NMR (79 MHz, Chloroform-d)** δ -1.91 ppm; **IR(ATR):** $\tilde{\nu}$ 3325, 3067, 3025, 2926, 2858, 1603, 1496, 1453, 1427, 1247, 1176, 1113, 1052, 837, 815, 772, 729, 699 cm^{-1}; **HRMS (ESI)** calculated $[M-C_6H_5]^+$ for $C_{16}H_{27}OSi^+$ = 263.1826, found: 263.1816.

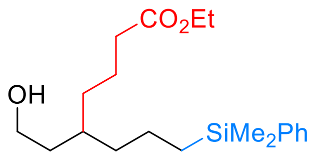

ethyl 8-(dimethyl(phenyl)silyl)-5-(2-hydroxyethyl)-octanoate (4ab). This compound was synthesized according to general procedure. The residue was purified by column chromatography on silica gel to afford the product **4ab** (76.6 mg, 55% yield) as a colorless oil; **^{1}H NMR (400 MHz, Chloroform-d)** δ 7.51 - 7.47 (m, 2H), 7.36 - 7.32 (m, 3H), 4.11 (q, J = 7.2 Hz, 2H), 3.61 (t, J = 6.7 Hz, 2H), 2.25 (t, J = 7.4 Hz, 2H), 1.62 - 1.52 (m, 2H), 1.52 - 1.40 (m, 4H), 1.31 - 1.20 (m, 9H), 0.74 - 0.68 (m, 2H), 0.24 (s, 6H) ppm; **^{13}C NMR (101 MHz, Chloroform-d)** δ 174.0, 139.7, 133.7, 128.9, 127.8, 61.1, 60.4, 37.8, 36.70, 34.68, 33.8, 33.1, 22.0, 21.0, 16.1, 14.4, -2.9 ppm; **^{29}Si NMR (79 MHz, Chloroform-d)** δ -2.64 ppm; **IR(ATR):** $\tilde{\nu}$ 3413, 3069, 2924, 2862, 1735, 1456, 1427, 1372, 1248, 1178, 1113, 1039, 836, 731, 700 cm^{-1}; **HRMS (ESI)** calculated $[M+H]^+$ for $C_{20}H_{35}O_3Si^+$ = 351.2350, found: 351.2336.

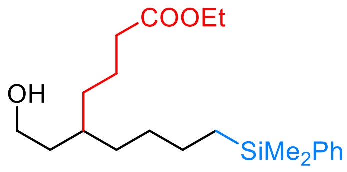

ethyl-9-(dimethyl(phenyl)silyl)-5-(2-hydroxyethyl)-nonanoate (**4ac**). This compound was synthesized according to general procedure. The residue was purified by column chromatography on silica gel to afford the product **4ac** (77.3 mg, 53% yield, rr > 20:1) as a colorless oil; **^{1}H NMR (400 MHz, Chloroform-*d*)** δ 7.53 - 7.46 (m, 2H), 7.38 - 7.28 (m, 3H), 4.11 (q, J = 7.1 Hz, 2H), 3.62 (t, J = 6.8 Hz, 2H), 2.26 (t, J = 7.4 Hz, 2H), 1.78 - 1.66 (m, 1H), 1.63 - 1.54 (m, 2H), 1.52 - 1.46 (m, 2H), 1.46 - 1.37 (m, 1H), 1.31 - 1.09 (m, 11H), 0.81 - 0.65 (m, 2H), 0.25 (s, 3H), 0.24 (s, 3H) ppm; **^{13}C NMR (101 MHz, Chloroform-*d*)** δ 174.0,

139.7, 133.6, 128.8, 127.8, 61.0, 60.3, 36.8, 34.7, 34.0, 33.3, 33.1, 30.5, 24.3, 22.0, 15.8, 14.3, -2.9 ppm; **^{29}Si NMR (119 MHz, Chloroform-d)** δ -3.15 ppm; **HRMS (ESI)** calculated $[M+NH_4]^+$ for $C_{21}H_{40}O_3NSi^+$ = 382.2773, found: 382.2774.

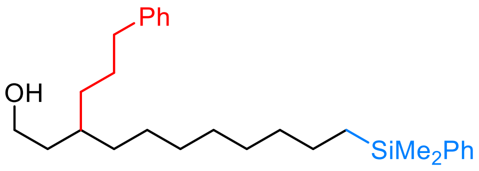

11-(dimethyl(phenyl)silyl)-3-(3-phenylpropyl)-undecan-1-ol (**4ad**). This compound was synthesized according to general procedure. The residue was purified by column chromatography on silica gel to afford the product **4ad** (130.8 mg, 77% yield, rr > 20:1) as a colorless oil; **^{1}H NMR (400 MHz, Chloroform-*d*)** δ 7.54 - 7.47 (m, 2H), 7.38 - 7.31 (m, 3H), 7.29 - 7.23 (m, 2H), 7.20 - 7.12 (m, 3H), 3.63 (t, *J* = 6.9 Hz, 2H), 2.58 (t, *J* = 7.7 Hz, 2H), 1.66 - 1.55 (m, 2H), 1.55 - 1.48 (m, 2H), 1.48 - 1.40 (m, 1H), 1.35 - 1.27 (m, 6H), 1.26 - 1.17 (m, 10H), 0.73 (t, *J* = 7.7 Hz, 2H), 0.25 (s, 6H) ppm; **^{13}C NMR (101 MHz, Chloroform-*d*)** δ 142.9, 139.9, 133.7, 128.9, 128.5, 128.4, 127.8, 125.8, 61.4, 37.0, 36.4, 34.2, 33.8, 33.8, 33.5, 30.2, 29.7, 29.4, 28.6, 26.7, 24.0, 15.8, -2.9 ppm; **^{29}Si NMR (119 MHz, Chloroform-d)** δ -3.12 ppm; **HRMS (ESI)** calculated $[M+NH_4]^+$ for $C_{28}H_{48}ONSi^+$= 442.3500, found:442.3515.

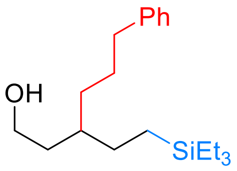

6-phenyl-3-(2-(triethylsilyl)-ethyl)-hexan-1-ol (**4ae**). This compound was synthesized according to general procedure using $Et_3SiBpin$, stirring at 30 °C for 24 h. The residue was purified by column chromatography on silica gel to afford the product **4ae** (74.2 mg, 58% yield) as a colorless oil; **^{1}H NMR** (400 MHz, Chloroform-*d*) δ 7.29 - 7.25 (m, 2H), 7.20 - 7.15 (m, 3H), 3.63 (t, *J* = 7.0 Hz, 2H), 2.64 - 2.53 (m, 2H), 1.62 - 1.48 (m, 4H), 1.41 (q, *J* = 6.2 Hz, 2H), 1.35 - 1.23 (m, 4H), 0.91 (td, J = 7.9, 2.3 Hz, 9H), 0.52 - 0.40 (m, 8H) ppm; **^{13}C NMR (101 MHz, Chloroform-*d*)** δ 142.8, 128.5, 128.4, 125.8, 61.4, 36.9, 36.4, 32.8, 28.6, 27.1, 7.6, 7.1, 3.3 ppm; **^{29}Si NMR (79 MHz, Chloroform-d)** δ 7.82 ppm; **IR(ATR):** $\tilde{\nu}$ 3311, 3063, 3026, 2949, 2933, 2911, 2873, 1604, 1496, 1454, 1416, 1377, 1238, 1016, 971, 777, 729, 699 cm^{-1}; **HRMS (ESI)** calculated $[M+H]^+$ for $C_{20}H_{37}OSi^+$ = 321.2608, found: 321.2600.

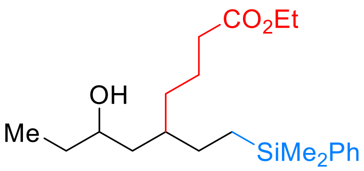

ethyl 5-(2-(dimethyl(phenyl)silyl)ethyl)-7-hydroxynonanoate (4af). This compound was synthesized according to general procedure. The residue was purified by column chromatography on silica gel to afford the product **4af** (123.0 mg, 84% yield) as a colorless oil; **^{1}H NMR (400 MHz, Chloroform-*d*)** δ 7.53 - 7.47 (m, 2H), 7.37 - 7.32 (m, 3H), 4.15 - 4.09 (m, 2H), 3.59 - 3.43 (m, 1H), 2.26 (td, *J* = 7.4, 1.4 Hz, 2H), 1.60 - 1.52 (m, 2H), 1.51 - 1.43 (m, 2H), 1.41 - 1.36 (m, 2H), 1.35 - 1.28 (m, 4H), 1.25 (t, *J* = 7.2 Hz, 4H), 0.92 (td, *J* = 7.5, 3.3 Hz, 3H), 0.77 - 0.59 (m, 2H), 0.26 (d, *J* = 1.9 Hz, 6H) ppm; **^{13}C NMR (101 MHz, Chloroform-*d*)** δ 174.0, 139.6, 133.7, 129.0, 127.9, 71.1, 60.4, 41.0, 36.4, 34.8, 32.2, 31.0, 27.8, 21.9, 14.4, 11.8, 10.0, -3.03, -3.08 ppm; **^{29}Si NMR (79 MHz, Chloroform-*d*)** δ -1.97 ppm; **IR(ATR):** $\tilde{\nu}$ 3443, 3069, 2956, 2922, 2875, 1735, 1461, 1427, 1373, 1248, 1177, 1113, 1031, 966, 837, 815, 774, 729, 701 cm^{-1}; **HRMS (ESI)** calculated $[M+H]^+$ for $C_{21}H_{37}O_3Si^+$ = 365.2507, found: 365.2500.

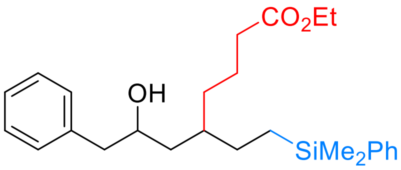

ethyl 5-(2-(dimethyl(phenyl)silyl)ethyl)-7-hydroxy-8-phenyloctanoate (4ag). This compound was synthesized according to general procedure. The residue was purified by column chromatography on silica gel to afford the product **4ag** (128.7 mg, 75% yield) as a colorless oil; **^{1}H NMR (400 MHz, Chloroform-*d*)** δ 7.50 (dtd, *J* = 7.1, 4.1, 2.0 Hz, 2H), 7.35 (dq, *J* = 3.7, 1.6 Hz, 3H), 7.30 (dt, *J* = 6.9, 1.3 Hz, 2H), 7.25 - 7.21 (m, 1H), 7.18 (dd, *J* = 8.0, 1.5 Hz, 2H), 4.12 (qd, *J* = 7.1, 1.7 Hz, 2H), 3.90 - 3.77 (m, 1H), 2.77 (dd, *J* = 13.6, 4.1 Hz, 1H), 2.60 (dd, *J* = 13.5, 8.4 Hz, 1H), 2.25 (td, *J* = 7.4, 1.7 Hz, 2H), 1.51 (ddt, *J* = 18.6, 8.6, 6.0 Hz, 5H), 1.38 - 1.28 (m, 4H), 1.26 - 1.22 (m, 3H), 0.76 - 0.62 (m, 2H), 0.26 (d, *J* = 1.7 Hz, 6H) ppm; **^{13}C NMR (101 MHz, Chloroform-*d*)** δ 173.9, 139.5, 138.7, 133.7, 129.5, 129.0, 128.7, 127.9, 126.6, 70.6, 60.4, 44.8, 40.8, 36.4, 34.8, 32.2, 27.6, 21.9, 14.4, 11.7, -3.0 ppm; **^{29}Si NMR (79 MHz, Chloroform-*d*)** δ -1.94 ppm; **IR(ATR):** $\tilde{\nu}$ 3455, 3067, 3025, 2918, 2872, 1733, 1602, 1496, 1454, 1427, 1372, 1300, 1248, 1180, 1113, 1031, 837, 815, 774, 730, 701 cm^{-1}; **HRMS (ESI)** calculated $[M+H]^+$ for $C_{26}H_{39}O_3Si^+$ = 427.2663, found:427.2649.

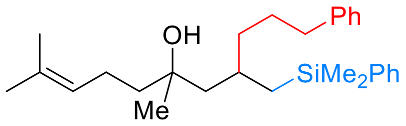

8-((dimethyl-(phenyl)-silyl)-methyl)-2,6-dimethyl-11-phenylundec-2-en-6-ol (**4ah**). This compound was synthesized according to general procedure. The residue was purified by column chromatography on silica gel to afford the product **4ah** (105.1 mg, 62% yield, dr = 1.5/1) as a colorless oil; **^{1}H NMR (400 MHz, Chloroform-*d*)** δ 7.50 (dtd, *J* = 7.1, 4.1, 3.6, 2.0 Hz, 2H), 7.34 (tt, *J* = 3.1, 1.5 Hz, 3H), 7.26 - 7.23 (m, 2H), 7.19 - 7.09 (m, 3H), 5.10 - 4.98 (m, 1H), 2.45 (td, *J* = 7.7, 2.9 Hz, 2H), 1.94 (dq, *J* = 13.5, 6.7, 6.2 Hz, 2H), 1.77 - 1.70 (m, 1H), 1.68 (d, *J* = 1.4 Hz, 3H), 1.59 (d, *J* = 1.6 Hz, 3H), 1.56 - 1.50 (m, 2H), 1.44 - 1.38 (m, 1H), 1.37 - 1.25 (m, 5H), 1.05 (s, 3H), 0.97 - 0.82 (m, 2H), 0.28 (d, *J* = 2.4 Hz, 6H) ppm; **^{13}C NMR (101 MHz, Chloroform-*d*)** δ 142.8, 140.1, 133.7, 131.7, 128.9, 128.5, 128.3, 127.9, 125.7, 124.6, 73.4, 48.8, 43.1, 38.0, 36.3, 30.0, 28.6, 27.0, 25.8, 23.6, 22.7, -1.8, -2.0 ppm; **^{29}Si NMR (79 MHz, Chloroform-*d*)** δ -3.58 ppm; **IR(ATR):** $\tilde{\nu}$ 3461, 3067, 3025, 2925, 2856, 1603, 1496, 1453, 1427, 1375, 1248, 1112, 1030, 910, 832, 734, 699 cm^{-1}; **HRMS (ESI)** calculated $[M-OH]^+$ for $C_{28}H_{41}Si^+$ = 405.2972, found: 405.2971.

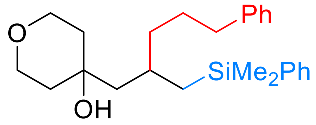

4-(2-((dimethyl-(phenyl)-silyl)-methyl)-5-phenylpentyl)-tetra-hydro-2H-pyran-4-ol (**4ai**). This compound was synthesized according to general procedure. The residue was purified by column chromatography on silica gel to afford the product **4ai** (146.1 mg, 92% yield) as a colorless oil; **^{1}H NMR (400 MHz, Chloroform-*d*)** δ 7.52 - 7.47 (m, 2H), 7.38 - 7.31 (m, 3H), 7.28 - 7.21 (m, 2H), 7.19 - 7.08 (m, 3H), 3.62 (ddd, *J* = 10.3, 7.7, 2.9 Hz, 4H), 2.53 - 2.40 (m, 2H), 1.81 - 1.67 (m, 1H), 1.58 - 1.43 (m, 4H), 1.42 - 1.22 (m, 6H), 0.93 - 0.81 (m, 3H), 0.28 (d, *J* = 1.7 Hz, 6H) ppm; **^{13}C NMR (101 MHz, Chloroform-*d*)** δ 142.7, 139.9, 133.7, 129.1, 128.5, 128.4, 128.0, 125.8, 69.6, 63.8, 50.3, 38.4, 38.2, 38.0, 36.2, 28.9, 28.6, 23.8, -1.8, -2.1 ppm; **^{29}Si NMR (79 MHz, Chloroform-*d*)** δ -3.53 ppm; **IR(ATR):** $\tilde{\nu}$ 3445, 3067, 3024, 2946, 2859, 1603, 1495, 1453, 1427, 1386, 1300, 1247, 1112, 1017, 981, 833, 815, 734, 700 cm^{-1}; **HRMS (ESI)** calculated $[M-H_2O+H]^+$ for $C_{25}H_{35}OSi^+$ = 379.2452, found: 379.2442.

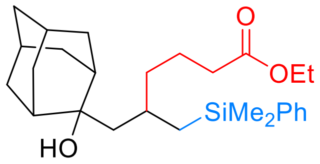

ethyl 6-(dimethyl-(phenyl)-silyl)-5-(((1r,3r)-2-hydroxyadamantan-2-yl)-methyl)-hexanoate (4aj). This compound was synthesized according to general procedure. The residue was purified by column chromatography on silica gel to afford the product **4aj** (168.8 mg, 95% yield) as a colorless oil; **^{1}H NMR (400 MHz, Chloroform-*d*)** δ 7.55 - 7.48 (m, 2H), 7.37 - 7.30 (m, 3H), 4.11 (q, *J* = 7.1 Hz, 2H), 2.16 (t, *J* = 7.5 Hz, 2H), 2.09 (d, *J* = 12.7 Hz, 2H), 1.84 - 1.67 (m, 4H), 1.67 - 1.51 (m, 12H), 1.49 - 1.44 (m, 2H), 1.25 (t, *J* = 7.2 Hz, 4H), 0.98 - 0.84 (m, 2H), 0.30 (d, *J* = 4.8 Hz, 6H) ppm; **^{13}C NMR (101 MHz, Chloroform-*d*)** δ 174.0, 140.1, 133.8, 128.9, 127.9, 75.7, 60.3, 44.8, 38.5, 37.6, 37.5, 37.4, 34.8, 34.6, 33.1, 33.0, 29.0, 27.4, 23.5, 21.8, 14.4, -1.7, -2.0 ppm; **^{29}Si NMR (119 MHz, Chloroform-*d*)** δ -4.05 ppm; **IR(ATR):** $\tilde{\nu}$ 3523, 3068, 2904, 2857, 1734, 1456, 1427, 1372, 1247, 1180, 1112, 1029, 999, 929, 832, 729, 703 cm^{-1}; **HRMS (ESI)** calculated $[M-H_2O+H]^+$ for $C_{27}H_{41}O_2Si^+$ = 425.2870, found: 425.2861.

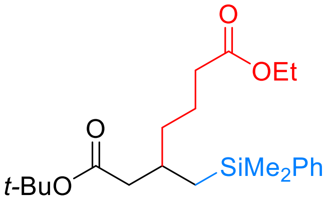

1-(tert-butyl)-7-ethyl 3-((dimethyl-(phenyl)-silyl)-methyl) - heptanedioate (4am). This compound was synthesized according to general procedure. The residue was purified by column chromatography on silica gel to afford the product **4am** (114.4 mg, 71% yield) as a colorless oil; **^{1}H NMR (400 MHz, Chloroform-*d*)** δ 7.53 - 7.48 (m, 2H), 7.34 (dt, *J* = 4.4, 1.5 Hz, 3H), 4.10 (q, *J* = 7.1 Hz, 2H), 2.19 - 2.05 (m, 4H), 2.00 (p, *J* = 6.5 Hz, 1H), 1.55 (ddd, *J* = 15.5, 8.4, 5.6 Hz, 2H), 1.42 (s, 9H), 1.29 - 1.26 (m, 1H), 1.24 (t, *J* = 7.2 Hz, 4H), 0.83 (dd, *J* = 6.8, 2.0 Hz, 2H), 0.31 (d, *J* = 2.0 Hz, 6H) ppm; **^{13}C NMR (101 MHz, Chloroform-*d*)** δ 173.7, 172.6, 139.6, 133.6, 129.0, 127.9, 80.2, 60.3, 43.1, 36.2, 34.5, 31.8, 28.2, 22.0, 21.2, 14.4, -2.0, -2.1 ppm; **^{29}Si NMR (79 MHz, Chloroform-*d*)** δ -3.52 ppm; **IR(ATR):** $\tilde{\nu}$ 3069, 2977, 2965, 2883, 1732, 1456, 1427, 1367, 1250, 1146, 1113, 1035, 950, 833, 731, 701 cm^{-1}; **HRMS (ESI)** calculated $[M+H]^+$ for $C_{22}H_{37}O_4Si^+$ = 393.2456, found: 393.2449.

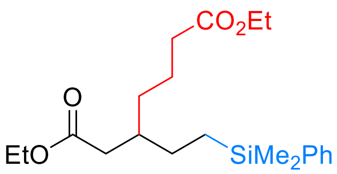

diethyl 3-(2-(dimethyl(phenyl)silyl)ethyl)heptanedioate (4an). This compound was synthesized according to general procedure. The residue was purified by column chromatography on silica gel to afford the product **4an** (113.6 mg, 75% yield) as a colorless oil; **^{1}H NMR (400 MHz, Chloroform-*d*)** δ 7.49 (tdd, *J* = 5.7, 4.6, 2.7 Hz, 2H), 7.39 - 7.30 (m, 3H), 4.10 (dq, *J* = 10.2, 7.1 Hz, 4H), 2.29 - 2.20 (m, 4H), 1.88 - 1.77 (m, 1H), 1.56 (q, J = 7.8 Hz, 2H), 1.36 - 1.29 (m, 3H), 1.28 - 1.18 (m, 7H), 0.75 - 0.64 (m, 2H), 0.25 (s, 6H) ppm; **^{13}C NMR (101 MHz, Chloroform-*d*)** δ 173.8, 173.5, 139.3, 133.7, 129.0, 127.9, 60.4, 60.3, 38.7, 37.5, 34.6, 32.8, 27.6, 22.2, 14.4, 11.9, -3.05, -3.10 ppm; **^{29}Si NMR (79 MHz, Chloroform-*d*)** δ -2.00 ppm; **IR(ATR):** $\tilde{\nu}$ 3335, 3069, 3065, 3024, 2977, 2900, 2856, cm^{-1}; **IR(ATR):** $\tilde{\nu}$ 3069, 2957, 2924, 1734, 1447, 1427, 1372, 1248, 1182, 1113, 1035, 838, 814, 775, 730, 701 cm^{-1}; **HRMS (ESI)** calculated $[M+H]^+$ for $C_{21}H_{35}O_4Si^+$ = 379.2299, found: 379.2291.

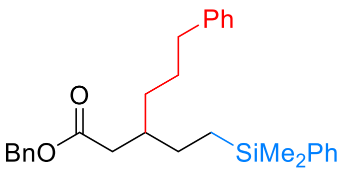

benzyl 3-(2-(dimethyl(phenyl)silyl)ethyl)-6-phenylhexanoate (4ao). This compound was synthesized according to general procedure. The residue was purified by column chromatography on silica gel to afford the product **4ao** (115.6 mg, 65% yield) as a colorless oil; **^{1}H NMR (400 MHz, Chloroform-d)** δ 7.49 - 7.44 (m, 2H), 7.36 - 7.30 (m, 8H), 7.28 - 7.24 (m, 2H), 7.19 - 7.10 (m, 3H), 5.06 (s, 2H), 2.52 (dd, *J* = 8.8, 6.7 Hz, 2H), 2.36 - 2.19 (m, 2H), 1.93 - 1.79 (m, 1H), 1.53 (dq, *J* = 9.2, 7.5, 6.9 Hz, 2H), 1.41 - 1.20 (m, 4H), 0.65 (dd, *J* = 9.4, 8.1 Hz, 2H), 0.22 (s, 6H) ppm; **^{13}C NMR (101 MHz, Chloroform-d)** δ 173.5, 142.6, 139.3, 136.2, 133.7, 129.0, 128.7, 128.5, 128.4, 128.3, 127.9, 125.8, 66.2, 38.8, 37.6, 36.2, 32.9, 28.5, 27.6, 11.8, -3.06, -3.10 ppm; **^{29}Si NMR (79 MHz, Chloroform-d)** δ -2.00 ppm; **IR(ATR):** $\tilde{\nu}$ 3066, 3026, 2926, 2856, 1733, 1603, 1496, 1454, 1427, 1379, 1248, 1145, 1113, 1029, 998, 837, 814, 774, 732, 699 cm^{-1}; **HRMS (ESI)** calculated $[M-C_6H_5]^+$ for $C_{23}H_{31}O_2Si^+$ = 367.2088, found: 367.2076.

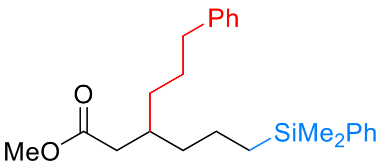

methyl 6-(dimethyl(phenyl)silyl)-3-(3-phenylpropyl)-hexanoate (4ap). This compound was synthesized according to general procedure. The residue was purified by column chromatography on silica gel to afford the product **4ap** (113.6 mg, 32% yield, rr = 10:1) as a colorless oil; **^{1}H NMR (400 MHz, Chloroform-*d*)** δ 7.44 - 7.38 (m, 2H), 7.27 (q, *J* = 3.3 Hz, 3H), 7.21 - 7.17 (m, 2H), 7.11 - 7.05 (m, 3H), 3.54 (s, 3H), 2.49 (t, *J* = 7.8 Hz, 2H), 2.12 (d, *J* = 6.9 Hz, 2H), 1.84 - 1.76 (m, 1H), 1.51 - 1.47 (m, 2H), 1.27 - 1.17 (m, 6H), 0.63 (dd, *J* = 8.9, 6.3 Hz, 2H), 0.17 (s, 6H) ppm; **^{13}C NMR (151 MHz, Chloroform-*d*)** δ 174.1, 142.6, 139.6, 133.7, 128.9, 128.5, 128.4, 127.9, 125.8, 51.5, 39.1, 38.0, 36.2, 34.8, 33.6, 28.5, 21.0, 15.9, -2.86, -2.89 ppm; **^{29}Si NMR (119 MHz, Chloroform-*d*)** δ -3.15 ppm.

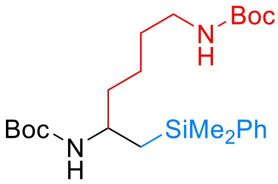

di-tert-butyl (6-(dimethyl(phenyl)-silyl)-hexane-1,5-diyl)-dicar-bamate (**4aq**). This compound was synthesized according to general procedure. The residue was purified by column chromatography on silica gel to afford the product **4aq** (113.5 mg, 63% yield) as a yellow oil; **^{1}H NMR (600 MHz, Chloroform-*d*)** δ 7.51 - 7.48 (m, 2H), 7.36 - 7.33 (m, 3H), 4.57 (s, 1H), 4.20 (d, J = 9.2 Hz, 1H), 3.77 - 3.68 (m, 1H), 3.08 - 3.00 (m, 2H), 1.45 - 1.42 (m, 11H), 1.41 (s, 9H), 1.37 - 1.32 (m, 2H), 1.29 - 1.25 (m, 2H), 1.04 - 0.93 (m, 2H), 0.32 (s, 6H) ppm; **^{13}C NMR (151 MHz, Chloroform-*d*)** δ 156.1, 155.2, 139.3, 133.6, 129.1, 128.0, 79.0, 78.9, 47.7, 40.5, 39.0, 29.6, 28.6, 28.5, 23.6, 23.0, -2.4, -2.6 ppm; **^{29}Si NMR (119 MHz, Chloroform-*d*)** δ -4.67 ppm; **IR(ATR):** $\tilde{\nu}$ 3332, 3069, 2978, 2934, 2868, 1734, 1680, 1533, 1453, 1427, 1391, 1366, 1300, 1277, 1250, 1173, 1113, 1088, 1010, 835, 800, 741, 712 cm^{-1}; **HRMS (ESI)** calculated $[M+H]^+$ for $C_{24}H_{43}O_4N_2Si^+$ = 451.2987, found: 451.2977.

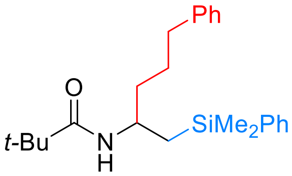

N-(1-(dimethyl-(phenyl)-silyl)-5-phenylpentan-2-yl)-pivalam-ide (4ar). This compound was synthesized according to general procedure. The residue was purified by column chromatography on silica gel to afford the product **4ar** (114.5 mg, 75% yield, rr > 20:1) as a colorless solid; **^{1}H NMR (600 MHz, Chloroform-*d*)** δ 7.51 - 7.42 (m, 2H), 7.36 - 7.32 (m, 3H), 7.27 - 7.22 (m,

2H), 7.18 - 7.14 (m, 1H), 7.12 - 7.09 (m, 2H), 5.19 (d, J = 8.6 Hz, 1H), 4.16 - 4.07 (m, 1H), 2.61 - 2.42 (m, 2H), 1.60 - 1.51 (m, 2H), 1.51 - 1.44 (m, 1H), 1.43 - 1.36 (m, 1H), 1.03 - 1.02 (m, 2H), 1.01 (s, 9H), 0.30 (s, 3H), 0.27 (s, 3H) ppm; **^{13}C NMR (151 MHz, Chloroform-*d*)** δ 177.2, 142.5, 139.3, 133.6, 129.2, 128.5, 128.4, 128.1, 125.8, 46.3, 38.5, 38.2, 35.7, 27.8, 27.5, 22.8, -2.0, -2.7 ppm; **^{29}Si NMR (119 MHz, Chloroform-*d*)** δ -4.96 ppm; **IR(ATR):** $\tilde{\nu}$ 3373, 3086, 3065, 3025, 2955, 2905, 2862, 1631, 1604, 1517, 1478, 1455, 1427, 1397, 1247, 1211, 1114, 1095, 1029, 902, 838, 823, 802, 752, 726, 699 cm^{-1}; **HRMS (ESI)** calculated $[M+H]^+$ for $C_{24}H_{36}ONSi^+$ = 382.2561, found: 382.2550.

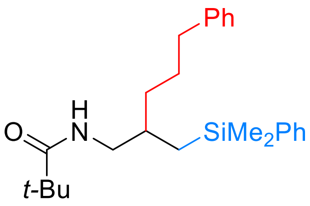

N-(2-((dimethyl-(phenyl)-silyl)-methyl)-5-phenylpentyl)-pivalamide (4as). This compound was synthesized according to general procedure. The residue was purified by column chromatography on silica gel to afford the product **4as** (103.2 mg, 64% yield, rr > 20:1) as a colorless oil; **^{1}H NMR (600 MHz, Chloroform-d)** δ 7.51 - 7.48 (m, 2H), 7.36 - 7.33 (m, 3H), 7.26 - 7.21 (m, 2H), 7.17 - 7.13 (m, 1H), 7.12 - 7.09 (m, 2H), 5.46 - 5.37 (m, 1H), 3.13 (dt, J = 13.4, 6.0 Hz, 1H), 3.06 (dt, J = 13.3, 6.0 Hz, 1H), 2.51 - 2.43 (m, 2H), 1.73 - 1.65 (m, 1H), 1.62 - 1.50 (m, 2H), 1.28 - 1.16 (m, 2H), 1.10 (s, 9H), 0.78 - 0.68 (m, 2H), 0.29 (d, J = 2.4 Hz, 6H) ppm; **^{13}C NMR (151 MHz, Chloroform-d)** δ 178.4, 142.4, 139.5, 133.6, 129.1, 128.5, 128.4, 128.0, 125.8, 44.7, 38.7, 36.1, 34.7, 34.0, 28.2, 27.7, 19.2, -2.1, -2.2 ppm; **^{29}Si NMR (79 MHz, Chloroform-d)** δ -3.71 ppm; **IR(ATR):** $\tilde{\nu}$ 3349, 3067, 3025, 2954, 2919, 2866, 1636, 1533, 1496, 1454, 1427, 1366, 1295, 1248, 1208, 1112, 1030, 833, 783, 731, 699 cm^{-1}; **HRMS (ESI)** calculated $[M+H]^+$ for $C_{25}H_{38}ONSi^+$ = 396.2717, found: 396.2704.

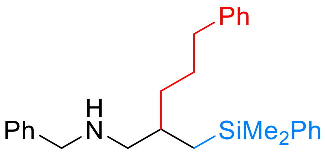

N-benzyl-3-((dimethyl-(phenyl)-silyl)-methyl)-6-phenylhexanamide (4at). This compound was synthesized according to general procedure. The residue was purified by column chromatography on silica gel to afford the product **4at** (109.3 mg, 68% yield, rr > 20:1) as a colorless oil; **^{1}H NMR (600 MHz, Chloroform-*d*)** δ 7.54 - 7.43 (m, 2H), 7.41 - 7.27 (m, 5H), 7.27 - 7.21 (m, 5H), 7.19 - 7.14 (m, 1H), 7.14 - 7.06 (m, 2H), 3.63 (d, J = 1.5 Hz, 2H), 2.52 -

2.36 (m, 4H), 1.73 - 1.66 (m, 1H), 1.55 - 1.47 (m, 3H), 1.38 (ddt, J = 13.2, 9.2, 6.3 Hz, 1H), 1.31 - 1.24 (m, 1H), 0.82 (dd, J = 14.9, 6.7 Hz, 1H), 0.76 (dd, J = 14.9, 7.0 Hz, 1H), 0.25 (d, J = 0.9 Hz, 6H) ppm; **^{13}C NMR (151 MHz, Chloroform-*d*)** δ 142.8, 140.6, 140.0, 133.6, 128.9, 128.5, 128.44, 128.36, 128.2, 127.9, 127.0, 125.8, 55.1, 54.1, 36.3, 34.63, 34.60, 28.4, 19.4, -1.97 -2.02 ppm; **^{29}Si NMR (119 MHz, Chloroform-*d*)** δ -3.70 ppm; **IR(ATR):** $\tilde{\nu}$ 3335, 3069, 3065, 3024, 2977, 2900, 2856, 1603, 1495, 1453, 1427, 1248, 1112, 1029, 832, 787, 733, 698 cm^{-1}; **HRMS (ESI)** calculated $[M+H]^+$ for $C_{27}H_{36}NSi^+$ = 402.2612, found: 402.2606.

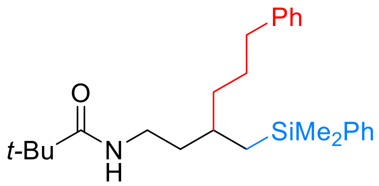

N-(3-((dimethyl-(phenyl)-silyl)-methyl)-6-phenylhexyl)-pivalamide (4au). This compound was synthesized according to general procedure. The residue was purified by column chromatography on silica gel to afford the product **4au** (111.1 mg, 68% yield, rr > 20:1) as a colorless oil; **^{1}H NMR (600 MHz, Chloroform-d)** δ 7.52 - 7.42 (m, 2H), 7.35 - 7.32 (m, 3H), 7.28 - 7.23 (m, 2H), 7.19 - 7.11 (m, 3H), 5.46 - 5.40 (m, 1H), 3.19 - 3.08 (m, 2H), 2.59 - 2.55 (m, 2H), 1.58 (p, J = 7.8 Hz, 2H), 1.52 - 1.43 (m, 1H), 1.33 - 1.19 (m, 4H), 1.11 (s, 9H), 0.72 - 0.66 (m, 2H), 0.24 (s, 6H) ppm; **^{13}C NMR (151 MHz, Chloroform-d)** δ 178.3, 142.5, 139.2, 133.6, 129.0, 128.5, 128.4, 127.9, 125.8, 42.0, 40.3, 38.8, 36.2, 30.7, 28.4, 27.7, 25.3, 11.8, -3.08, -3.11 ppm; **^{29}Si NMR (79 MHz, Chloroform-d)** δ -2.45 ppm; **IR(ATR):** $\tilde{\nu}$ 3350, 3067, 3025, 2954, 2926, 2862, 1636, 1537, 1496, 1453, 1427, 1366, 1248, 1209, 1113, 1030, 837, 815, 772, 729, 699 cm^{-1}; **HRMS (ESI)** calculated $[M+H]^+$ for $C_{26}H_{40}ONSi^+$ = 410.2874, found: 410.2861.

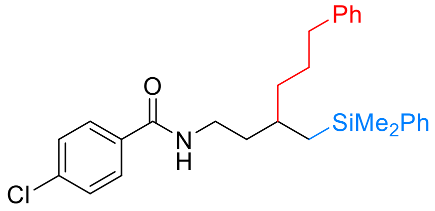

4-chloro-N-(3-((dimethyl-(phenyl)-silyl)-methyl)-6-phenylhexyl)-benzamide (4av). This compound was synthesized according to general procedure. The residue was purified by column chromatography on silica gel to afford the product **4av** (78.0 mg, 42% yield, rr > 20:1) as a colorless oil; **^{1}H NMR (600 MHz, Chloroform-*d*)** δ 7.54 - 7.49 (m, 2H), 7.49 - 7.45 (m, 2H), 7.38 - 7.28 (m, 5H), 7.27 - 7.22 (m, 2H), 7.20 - 7.09 (m, 3H), 5.91 (t, J = 5.9 Hz, 1H), 3.33 (t, J = 6.1 Hz, 2H), 2.57

(t, J = 7.5 Hz, 2H), 1.66 - 1.53 (m, 3H), 1.38 - 1.27 (m, 4H), 0.78 - 0.65 (m, 2H), 0.25 (d, J = 1.6 Hz, 6H) ppm; **^{13}C NMR (101 MHz, Chloroform-*d*)** δ 166.6, 142.4, 139.2, 137.5, 133.6, 133.3, 129.0, 128.8, 128.5, 128.41, 128.36, 127.9, 125.8, 42.6, 40.2, 36.1, 30.5, 28.3, 25.2, 11.6, -3.08, -3.13 ppm; **^{29}Si NMR (119 MHz, Chloroform-*d*)** δ -2.37 ppm; **IR(ATR):** $\tilde{\nu}$ 3301, 3067, 3025, 2924, 2856, 1635, 1597, 1545, 1487, 1453, 1427, 1312, 1248, 1179, 1113, 1093, 1015, 895, 838, 773, 729, 699 cm^{-1}; **HRMS (ESI)** calculated [M+H]$^+$ for $C_{28}H_{35}ONClSi^+$ = 464.2171, found: 464.2157.

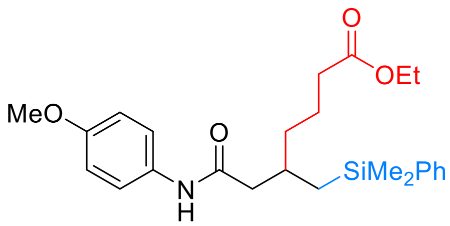

ethyl 5-((dimethyl(phenyl)-silyl)-methyl)-7-((4-methoxyphenyl)-amino)-7-oxoheptanoate (4aw). This compound was synthesized according to general procedure. The residue was purified by column chromatography on silica gel to afford the product **4aw** (134.4 mg, 76% yield) as a colorless oil; **^{1}H NMR (400 MHz, Chloroform-*d*)** δ 7.53 (dd, *J* = 6.5, 3.0 Hz, 2H), 7.35 (td, *J* = 5.7, 4.6, 2.1 Hz, 5H), 6.97 (s, 1H), 6.88 - 6.79 (m, 2H), 4.15 - 4.03 (m, 2H), 3.78 (s, 3H), 2.28 - 2.02 (m, 5H), 1.62 - 1.50 (m, 2H), 1.33 (dt, *J* = 9.2, 6.2 Hz, 2H), 1.22 (t, *J* = 7.1 Hz, 3H), 0.90 (d, *J* = 6.2 Hz, 2H), 0.32 (s, 6H) ppm; **^{13}C NMR (101 MHz, Chloroform-*d*)** δ 174.1, 170.6, 156.4, 139.6, 133.8, 131.3, 129.1, 128.0, 121.8, 114.2, 60.5, 55.6, 44.8, 36.1, 34.2, 32.3, 21.6, 21.2, 14.4, -2.0, -2.2 ppm; **^{29}Si NMR (79 MHz, Chloroform-*d*)** δ -3.35 ppm; **IR(ATR):** $\tilde{\nu}$ 3293, 3133, 3068, 2952, 2856, 1732, 1652, 1603, 1540, 1512, 1464, 1427, 1412, 1371, 1301, 1246, 1180, 1112, 1036, 830, 732, 702 cm^{-1}; **HRMS (ESI)** calculated [M+H]$^+$ for $C_{25}H_{36}O_4NSi^+$ = 442.2408, found: 442.2400.

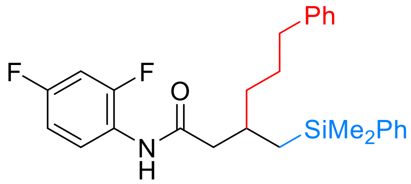

N-(2,4-difluorophenyl)-3-((dimethyl-(phenyl)-silyl)-methyl)-6-phenylhexanamide (4ax). This compound was synthesized according to general procedure. The residue was purified by column chromatography on silica gel to afford the product **4ax** (129.6 mg, 71% yield) as a yellow oil; **^{1}H NMR (400 MHz, Chloroform-*d*)** δ 8.17 (td, *J* = 8.9, 5.9 Hz, 1H), 7.54 - 7.47 (m, 2H), 7.34 (tt, *J* = 5.0, 2.2 Hz,

3H), 7.26 - 7.21 (m, 2H), 7.18 - 7.12 (m, 1H), 7.12 - 7.07 (m, 2H), 6.96 - 6.88 (m, 1H), 6.88 - 6.81 (m, 2H), 2.54 - 2.41 (m, 2H), 2.28 - 2.07 (m, 3H), 1.58 – 1.52 (m, 2H), 1.36 (dt, *J* = 8.4, 5.4 Hz, 2H), 0.93 - 0.82 (m, 2H), 0.30 (s, 6H) ppm; **^{13}C NMR (151 MHz, Chloroform-*d*)** δ 170.8, 158.6 (dd, *J* = 246.1, 11.5 Hz), 152.5 (dd, *J* = 246.0, 11.9 Hz), 142.5, 139.5, 133.7, 129.2, 128.5, 128.4, 128.0, 125.8, 123.1 (dd, *J* = 9.1, 2.0 Hz), 122.68 (dd, J = 10.4, 3.3 Hz), 111.3 (dd, J = 21.7, 3.7 Hz), 103.6 (dd, J = 26.7, 23.3 Hz), 45.2, 36.4, 36.1, 32.3, 28.6, 21.3, -2.0, -2.2 ppm; **^{29}Si NMR (79 MHz, Chloroform-*d*)** δ -3.47 ppm; **^{19}F NMR (565 MHz, Chloroform-*d*)** δ -115.31, -126.28 ppm; **IR(ATR):** $\tilde{\nu}$ 3261, 3068, 3065, 3025, 2930, 2857, 1663, 1612, 1525, 1515, 1428, 1372, 1258, 1205, 1141, 1112, 1097, 969, 835, 731, 699 cm^{-1}; **HRMS (ESI)** calculated [M+H]$^+$ for $C_{27}H_{32}ONF_2Si^+$ = 452.2216, found: 452.2221.

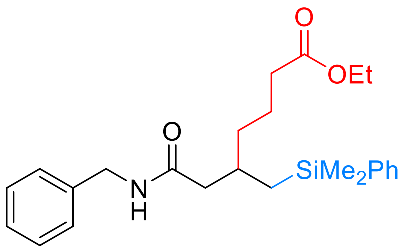

ethyl 7-(benzylamino)-5-((dimethyl-(phenyl)-silyl)-methyl)-7-oxoheptanoate (4ay). This compound was synthesized according to general procedure. The residue was purified by column chromatography on silica gel to afford the product **4ay** (150.9 mg, 87% yield) as a colorless oil; **^{1}H NMR (400 MHz, Chloroform-*d*)** δ 7.48 (ddt, J = 5.7, 3.8, 2.2 Hz, 2H), 7.34 - 7.26 (m, 6H), 7.25 - 7.21 (m, 2H), 5.59 (t, *J* = 5.7 Hz, 1H), 4.35 (d, *J* = 5.7 Hz, 2H), 4.07 (q, *J* = 7.2 Hz, 2H), 2.18 – 2.08 (m, 3H), 2.08 – 1.97 (m, 2H), 1.60 - 1.48 (m, 2H), 1.31 - 1.26 (m, 2H), 1.23 (t, *J* = 7.1 Hz, 3H), 0.84 (d, *J* = 6.3 Hz, 2H), 0.30 (d, *J* = 2.9 Hz, 6H) ppm; **^{13}C NMR (101 MHz, Chloroform-*d*)** δ 173.9, 172.2, 139.6, 138.6, 133.7, 129.1, 128.7, 128.0, 127.9, 127.5, 60.3, 44.0, 43.6, 36.0, 34.3, 32.2, 21.7, 21.1, 14.3, -2.1, -2.2 ppm; **^{29}Si NMR (79 MHz, Chloroform-d)** δ -3.43 ppm; **IR(ATR):** $\tilde{\nu}$ 3287, 3026, 2953, 2857, 1732, 1654, 1604, 1547, 1464, 1427, 1372, 1301, 1248, 1180, 1112, 1030, 835, 731, 700 cm^{-1}; **HRMS (ESI)** calculated[M+H]$^+$ for $C_{25}H_{36}O_3NSi^+$ = 426.2459,found: 426.2444.

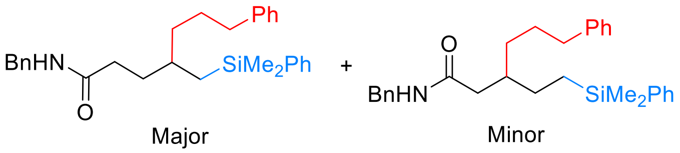

N-benzyl-4-((dimethyl-(phenyl)-silyl)-methyl)-7-phenylheptanamide (4az). This

compound was synthesized accordi-ng to general procedure. The residue was purified by column chromatography on silica gel to afford the product **4az** (153.0 mg, 87% yield，rr = 2:1) as a colorless oil; Major isomer **^{1}H NMR (600 MHz, Chloroform-*d*)** δ 7.47 (m, 1H), 7.34-7.18 (m, 11H), 7.17 - 7.09 (m, 3H), 5.39 (s , 1H), 4.34 - 4.26 (m, 2H), 2.50 - 2.41 (m, 2H), 2.04 - 1.94 (m, 2H), 1.60 - 1.45 (m, 5H), 1.37 - 1.27 (m, 1H), 1.24 (m, 1H), 0.82 - 0.70 (m, 2H), 0.26 (d, *J* = 6.0 Hz, 6H) ppm; Minor isomer **^{1}H NMR (600 MHz, Chloroform-*d*)** δ 7.47 (m, 4H), 7.34 - 7.18 (m, 7H), 7.17 - 7.07 (m, 4H), 5.71 (s, 1H), 4.35 (d, *J* = 5.8 Hz, 2H), 2.54 (td, *J* = 7.5, 2.5 Hz, 2H), 2.10 (dd, *J* = 14.1, 6.9 Hz, 1H), 2.04 - 2.00 (m, 1H), 1.88 (m, 1H), 1.62 - 1.45 (m, 2H), 1.34 - 1.28 (m, 2H), 1.24 (m, 2H), 0.68 - 0.62 (m, 2H), 0.23 (s, 6H) ppm; The mixture **^{13}C NMR (101 MHz, Chloroform-*d*)** δ 173.1, 172.6, 142.6, 140.0, 139.3, 138.6, 138.5, 133.62, 133.59, 128.94, 128.89, 128.73, 128.70, 128.49, 128.45, 128.33, 128.31, 127.9, 127.5, 125.74, 125.71, 43.55, 43.52, 41.3, 37.6, 36.2, 36.1, 35.7, 33.79, 33.76, 32.7, 32.2, 28.4, 28.3, 27.3, 20.8, 11.7, -1.9, -2.4, -3.1, -3.2 ppm; **^{29}Si NMR (119 MHz, Chloroform-d)** δ -2.50, -3.80 ppm; **IR(ATR):** $\tilde{\nu}$ 3287, 3066, 3026, 2927, 2857, 1739, 1644, 1604, 1548, 1496, 1454, 1427, 1372, 1248, 1112, 1030, 910, 835, 776, 731, 699 cm^{-1}; **HRMS (ESI)** calculated $[M+H]^+$ for $C_{29}H_{38}ONSi^+$ = 444.2717, found: 444.2713.

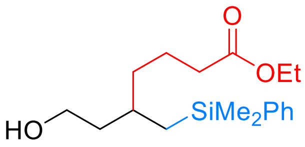

ethyl 5-((dimethyl-(phenyl)-silyl)-methyl)-7-hydroxyhe-ptano-ate (4ba). This compound was synthesized according to general procedure. The residue was purified by column chromatography on silica gel to afford the product **4ba** (73.1 mg, 57% yield) as a colorless oil; **^{1}H NMR (400 MHz, Chloroform-*d*)** δ 7.53 - 7.49 (m, 2H), 7.34 (dp, *J* = 4.8, 1.7 Hz, 3H), 4.10 (q, *J* = 7.1 Hz, 2H), 3.56 (td, *J* = 6.8, 1.6 Hz, 2H), 2.16 (t, *J* = 7.4 Hz, 2H), 1.65 (p, J = 6.4 Hz, 1H), 1.59 - 1.51 (m, 3H), 1.50 - 1.41 (m, 2H), 1.28 - 1.22 (m, 5H), 0.80 (dd, *J* = 6.6, 2.8 Hz, 2H), 0.30 (s, 6H) ppm; **^{13}C NMR (101 MHz, Chloroform-*d*)** δ 173.9, 139.8, 133.6, 128.9, 127.9, 60.7, 60.3, 39.4, 36.0, 34.5, 30.6, 21.8, 21.1, 14.3, -2.1 ppm; **^{29}Si NMR (79 MHz, Chloroform-d)** δ -3.29 ppm; **IR(ATR):** $\tilde{\nu}$ 3423, 3069, 2951, 2923, 2857, 1734, 1446, 1427, 1373, 1301, 1249, 1190, 1112, 1055, 833, 724, 701 cm^{-1}; **HRMS (ESI)** calculated $[M+H]^+$ for $C_{18}H_{31}O_3Si^+$ = 323.2037, found: 323.2028.

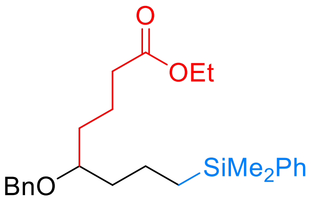

ethyl 5-(benzyloxy)-8-(dimethyl-(phenyl)-silyl)-octanoate (4bb). This compound was synthesized according to general procedure. The residue was purified by column chromatography on silica gel to afford the product **4bb** (72.3 mg, 44% yield, rr > 20:1) as a colorless oil; **^{1}H NMR (400 MHz, Chloroform-*d*)** δ 7.50 (ddd, *J* = 6.5, 2.9, 1.7 Hz, 2H), 7.35 - 7.33 (m, 3H), 7.29 (tdd, *J* = 8.7, 5.8, 2.2 Hz, 5H), 4.45 (d, *J* = 3.0 Hz, 2H), 4.12 (q, *J* = 7.1 Hz, 2H), 3.37 (ddd, *J* = 11.4, 6.2, 5.0 Hz, 1H), 2.28 (t, *J* = 7.4 Hz, 2H), 1.76 - 1.68 (m, 1H), 1.65 - 1.56 (m, 2H), 1.53 - 1.32 (m, 5H), 1.24 (t, *J* = 7.2 Hz, 3H), 0.73 (ddd, *J* = 9.8, 6.2, 2.8 Hz, 2H), 0.25 (d, *J* = 0.8 Hz, 6H) ppm; **^{13}C NMR (101 MHz, Chloroform-*d*)** δ 173.8, 139.6, 139.0, 133.7, 128.9, 128.4, 127.9, 127.8, 127.6, 78.3, 70.9, 60.3, 37.8, 34.5, 33.3, 21.0, 19.8, 16.0, 14.4, -2.9 ppm; **^{29}Si NMR (79 MHz, Chloroform-d)** δ -2.61 ppm; **IR(ATR):** $\tilde{\nu}$ 3068, 2932, 2863, 1734, 1496, 1455, 1427, 1372, 1349, 1248, 1178, 1113, 1067, 1028, 835, 733, 699 cm^{-1}; **HRMS (ESI)** calculated $[M+H]^+$ for $C_{25}H_{37}O_3Si^+$ = 413.2507, found: 413.2490.

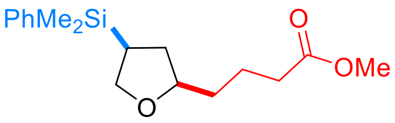

***cis*-methyl 4-(4-(dimethyl-(phenyl)-silyl)-tetrahydro-furan-2-yl)-butanoate (4bc).** This compound was synthesized according to general procedure. The residue was purified by column chromatography on silica gel to afford the product **4bc** (117.3 mg, 96% yield, rr > 20:1, dr > 20:1) as a colorless oil; **^{1}H NMR (400 MHz, Chloroform-d)** δ 7.49 - 7.45 (m, 2H), 7.38 - 7.33 (m, 3H), 3.95 (t, *J* = 8.4 Hz, 1H), 3.80 - 3.73 (m, 1H), 3.67 - 3.61 (m, 4H), 2.32 (ddd, *J* = 7.7, 7.0, 2.2 Hz, 2H), 2.06 (ddd, *J* = 12.1, 7.0, 5.1 Hz, 1H), 1.74 - 1.70 (m, 1H), 1.69 - 1.62 (m, 2H), 1.60 - 1.51 (m, 1H), 1.49 - 1.39 (m, 1H), 1.25 (td, *J* = 12.4, 9.4 Hz, 1H), 0.28 (d, *J* = 3.0 Hz, 6H) ppm; **^{13}C NMR (101 MHz, Chloroform-d)** δ 174.2, 137.8, 133.8, 129.3, 128.0, 80.1, 69.9, 51.6, 35.1, 35.0, 34.1, 27.5, 22.1, -4.4, -4.4 ppm; **^{29}Si NMR (79 MHz, Chloroform-d)** δ -3.04 ppm; **IR(ATR):** $\tilde{\nu}$ 3069, 2953, 2923, 2859, 1739, 1428, 1362, 1250, 1195, 1172, 1112, 1087, 1037, 932, 837, 817, 776, 735, 701 cm^{-1}; **HRMS (ESI)** calculated $[M+H]^+$ for $C_{17}H_{27}O_3Si^+$ = 307.1724, found: 307.1717.

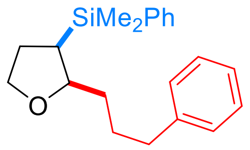

***cis*-dimethyl-(phenyl)-(2-(3-phenylpropyl)-tetrahydrofuran-3-yl)-silane (4bd).** This compound was synthesized according to general procedure. The residue was purified by column chromatography on silica gel to afford the product **4bd** (86.6 mg, 67% yield, rr > 20:1, dr > 20:1) as a colorless oil; **^{1}H NMR (600 MHz, Chloroform-*d*)** δ 7.48 - 7.43 (m, 2H), 7.37 - 7.32 (m, 3H), 7.26 - 7.22 (m, 2H), 7.17 - 7.10 (m, 3H), 3.73 (ddd, *J* = 9.6, 8.2, 2.9 Hz, 1H), 3.67 (dd, *J* = 8.0, 5.3 Hz, 2H), 2.58 - 2.49 (m, 2H), 1.98 (ddt, *J* = 12.2, 8.5, 5.3 Hz, 1H), 1.79 - 1.70 (m, 2H), 1.65 - 1.58 (m, 1H), 1.43 (dddd, *J* = 13.4, 10.4, 5.7, 2.9 Hz, 1H), 1.39 - 1.33 (m, 1H), 1.18 (ddd, *J* = 10.7, 9.6, 8.4 Hz, 1H), 0.29 (d, *J* = 0.7 Hz, 6H) ppm; **^{13}C NMR (151 MHz, Chloroform-d)** δ 142.6, 137.8, 133.9, 129.3, 128.5, 128.3, 128.0, 125.7, 81.5, 67.5, 35.9, 35.9, 31.7, 29.8, 28.2, -4.0, -4.2 ppm; **^{29}Si NMR (119 MHz, Chloroform-*d*)** δ -3.42 ppm; **IR(ATR):** $\tilde{\nu}$ 3067, 3025 , 2957, 2932, 2858, 1603, 1496, 1453, 1427, 1378, 1250, 1112, 1089, 1030, 944, 830, 816, 772, 734, 699 cm^{-1}; **HRMS (ESI)** calculated [M+H]$^{+}$ for $C_{21}H_{29}O_{3}Si^{+}$ = 325.1982, found: 325.1979.

1.5 Extended Scope and Synthetic Applications

2,2-dimethyl-4-(3-phenylpropyl)-1,2-oxasilinane[1]

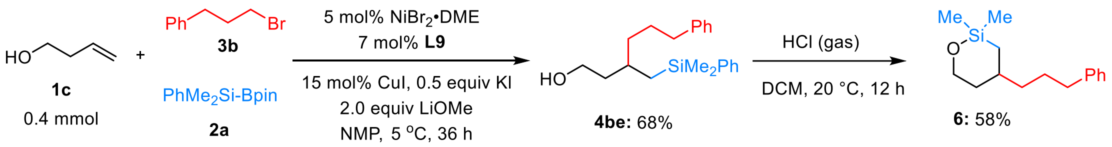

3-((dimethyl-(phenyl)-silyl)-methyl)-6-phenylhexan-1-ol (**4be**) was synthesized according to general procedure, obtained the pure compound **4be** as a colorless oil (88.8 mg, 68% yield).

^{1}H NMR (600 MHz, Chloroform-*d*) δ 7.52 - 7.48 (m, 2H), 7.36 - 7.32 (m, 3H), 7.27 - 7.24 (m, 2H), 7.19 - 7.14 (m, 1H), 7.13 - 7.09 (m, 2H), 3.54 (td, J = 6.8, 1.0 Hz, 2H), 2.47 (td, J = 7.7, 1.4 Hz, 2H), 1.67 - 1.62 (m, 1H), 1.57 - 1.48 (m, 3H), 1.45 - 1.40 (m, 1H), 1.31 - 1.24 (m, 2H), 0.82 - 0.75 (m, 2H), 0.28 (s, 6H) ppm; **^{13}C NMR (151 MHz, Chloroform-*d*)** δ 142.8, 140.0, 133.6, 129.0, 128.5, 128.4, 127.9, 125.8, 61.0, 39.7, 36.3, 36.2, 30.8, 28.4, 21.3, -1.99, -2.03 ppm; **^{29}Si NMR (119 MHz, Chloroform-*d*)** δ -3.79 ppm; **HRMS (ESI)** calculated [M-

$C_6H_5]^+$ for $C_{15}H_{25}OSi^+$ = 249.1669, found: 249.1662.

To an oven-dried round bottom flask containing a stirring bar was added a solution of **4be** (65.3 mg, 0.2 mmol, 1.0 equiv) in DCM (4.0 mL) under N_2 atmosphere. Then dry HCl was bubbled continuously through the solution for 12 h. The mixture was concentrated under reduced pressure and purified by flash column chromatography to provide the title compound **6** as a colorless oil (28.8 mg, 58% yield).

^{1}H NMR (600 MHz, Chloroform-*d*) δ 7.29 - 7.25 (m, 2H), 7.20 - 7.15 (m, 3H), 3.97 (ddd, J = 11.5, 4.0, 2.6 Hz, 1H), 3.77 (td, J = 11.8, 2.0 Hz, 1H), 2.61 - 2.57 (m, 2H), 1.69 - 1.60 (m, 3H), 1.56 (dq, J = 13.9, 2.3 Hz, 1H), 1.40 - 1.35 (m, 1H), 1.32 - 1.26 (m, 2H), 0.75 - 0.71 (m, 1H), 0.29 (dd, J = 14.2, 12.6 Hz, 1H), 0.14 (d, J = 8.1 Hz, 6H) ppm; **^{13}C NMR (151 MHz, Chloroform-*d*)** δ 142.9, 128.5, 128.4, 125.8, 64.7, 40.5, 37.1, 36.2, 34.7, 28.7, 20.6, -0.2, -2.4 ppm; **^{29}Si NMR (119 MHz, Chloroform-*d*)** δ 15.70 ppm; **IR(ATR):** $\tilde{\nu}$ 2959, 2924, 2852, 1647, 1496, 1454, 1427, 1374, 1330, 1260, 1171, 1131, 1029, 907, 801, 745, 700 cm^{-1}; **HRMS (ESI)** calculated $[M+H]^+$ for $C_{15}H_{25}OSi^+$ = 249.1669, found: 249.1660.

1-(2-((dimethyl-(phenyl)-silyl)-methyl)-pyrrolidin-1-yl)-2,2-dimethylpropan-1-one[2]

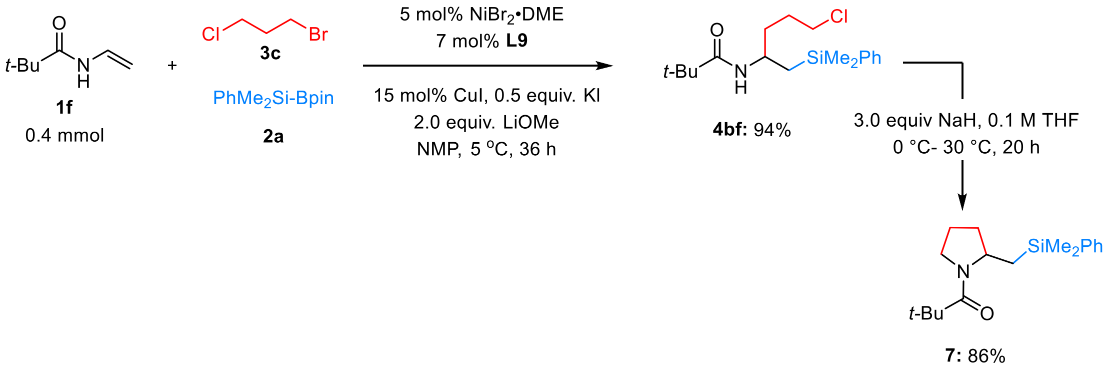

N-(5-chloro-1-(dimethyl-(phenyl)-silyl)-pentan-2-yl)-pivalamide (**4bf**) was synthesized according to general procedure, obtained the pure compound **4 bf** as a white solid (127.8 mg, 94 % yield);

^{1}H NMR (600 MHz, Chloroform-*d*) δ 7.54 - 7.49 (m, 2H), 7.40 - 7.32 (m, 3H), 5.22 (d, J = 8.6 Hz, 1H), 4.14 - 4.07 (m, 1H), 3.48 (t, J = 6.3 Hz, 2H), 1.72 - 1.68 (m, 2H), 1.67 - 1.61 (m, 1H), 1.51 - 1.45 (m, 1H), 1.06 (d, J = 7.2 Hz, 2H), 1.01 (s, 9H), 0.32 (d, J = 13.8 Hz, 6H) ppm; **^{13}C NMR (151 MHz, Chloroform-*d*)** δ 177.4, 139.1, 133.6, 129.3, 128.2, 45.9, 45.0, 38.6,

36.1, 29.1, 27.5, 23.1, -1.9, -2.8 ppm; **^{29}Si NMR (119 MHz, Chloroform-*d*)** δ -4.69 ppm; **IR(ATR):** $\tilde{\nu}$ 3338, 3068, 2969, 2941, 1627, 1532, 1481, 1427, 1366, 1296, 1248, 1215, 1113, 1086, 1030, 929, 905, 845, 815, 732, 705 cm^{-1}.

To an oven-dried round bottom flask containing a stirring bar was added a solution of **4bf** (136.0 mg, 0.4 mmol, 1.0 equiv) in THF (4.0 mL) under N_2 atmosphere. The reaction mixture was cooled with an ice-water bath. Then NaH (48 mg, 1.2 mmol, 3.0 equiv) was added in one portion. After the addition, the reaction mixture was warmed to room temperature and additionally stirred for 20 h. Then the reaction was quenched with saturated aqueous solution of NaCl (5 mL) and extracted with EtOAc (3 ×5 mL), dried over Na_2SO_4, concentrated under reduced pressure. The mixture was purified by flash column chromatography to provide the title compound **7** as a colorless oil (104.4 mg, 86% yield).

^{1}H NMR (600 MHz, Chloroform-*d*) δ 7.57 - 7.51 (m, 2H), 7.37 - 7.31 (m, 3H), 4.30 - 4.23 (m, 1H), 3.65 (dt, J = 10.2, 6.3 Hz, 1H), 3.44 (dt, J = 10.2, 6.7 Hz, 1H), 1.90 - 1.85 (m, 1H), 1.79 - 1.74 (m, 1H), 1.73 - 1.66 (m, 2H), 1.33 - 1.27 (m, 1H), 1.23 (s, 9H), 0.77 (t, J = 12.7 Hz, 1H), 0.34 (d, J = 13.2 Hz, 6H) ppm; **^{13}C NMR (151 MHz, Chloroform-*d*)** δ 178.2, 141.8, 136.1, 131.3, 130.2, 58.9, 49.7, 41.6, 33.5, 30.1, 28.0, 24.3, 0.4, -0.0 ppm; **^{29}Si NMR (119 MHz, Chloroform-*d*)** δ -4.81 ppm; **IR(ATR):** $\tilde{\nu}$ 3068, 3048, 2958, 2874, 1619, 1478, 1427, 1404, 1377, 1362, 1248, 1192, 1113, 915, 838, 791, 728, 701 cm^{-1}; **HRMS (ESI)** calculated $[M+H]^+$ for $C_{18}H_{30}ONSi^+$ =304.2091, found: 304.2081.

1-(3-((dimethyl-(phenyl)-silyl)-methyl)-piperidin-1-yl)-2,2-dimethylpropan-1-one[2]

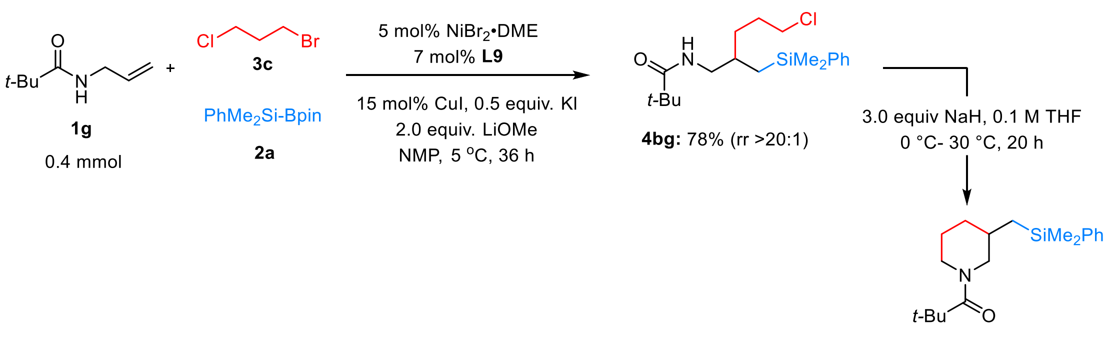

8: 47%

N-(5-chloro-2-((dimethyl-(phenyl)-silyl)-methyl)-pentyl)-pivalamide (**4bg**) was synthesized accord--ing to general procedure, obtained the pure compound **4bg** as a colorless oil (109.7 mg,

78% yield, rr > 20:1).

^{1}H NMR (600 MHz, Chloroform-*d*) δ 7.57 - 7.48 (m, 2H), 7.41 - 7.32 (m, 3H), 5.52 (d, J = 6.1 Hz, 1H), 3.46 - 3.35 (m, 2H), 3.17 (dt, J = 13.6, 5.9 Hz, 1H), 3.05 (dt, J = 13.6, 6.1 Hz, 1H), 1.72 (tq, J = 13.6, 6.7 Hz, 3H), 1.38 - 1.32 (m, 2H), 1.14 (s, 9H), 0.79 - 0.71 (m, 2H), 0.33 (d, J = 5.0 Hz, 6H) ppm; **^{13}C NMR (151 MHz, Chloroform-*d*)** δ 178.5, 139.3, 133.5, 129.2, 128.1, 45.4, 44.4, 38.8, 34.3, 31.6, 29.4, 27.7, 19.4, -2.1, -2.3 ppm; **^{29}Si NMR (119 MHz, Chloroform-*d*)** δ -3.72 ppm; **HRMS (ESI)** calculated $[M+H]^+$ for $C_{19}H_{33}ONClSi^+$ = 354.2015, found: 354.2003.

To an oven-dried round bottom flask containing a stirring bar was added a solution of **4bg** (35.0 mg, 0.1 mmol, 1.0 equiv) in THF (1.0 mL) under N_2 atmosphere. The reaction mixture was cooled with an ice-water bath. Then NaH (12 mg, 0.3 mmol, 3.0 equiv) was added in one portion. After the addition, the reaction mixture was warmed to room temperature and additionally stirred for 20 h. Then the reaction was quenched with saturated aqueous solution of NaCl (5 mL) and extracted with EtOAc (3 × 5 mL), dried over Na_2SO_4, concentrated under reduced pressure. The mixture was purified by flash column chromatography to provide the title compound **8** as a colorless oil (15.0 mg, 47% yield).

^{1}H NMR (600 MHz, Chloroform-*d*) δ 7.52 - 7.48 (m, 2H), 7.38 - 7.30 (m, 3H), 4.37 - 4.10 (m, 2H), 2.71 - 2.55 (m, 1H), 2.38 (t, J = 12.0 Hz, 1H), 1.81 (ddq, J = 12.3, 3.7, 1.8 Hz, 1H), 1.64 - 1.53 (m, 2H), 1.38 (dtd, J = 16.5, 12.7, 12.2, 8.2 Hz, 1H), 1.19 (s, 9H), 1.08 (tdd, J = 12.8, 11.2, 4.0 Hz, 1H), 0.80 - 0.67 (m, 2H), 0.31 (d, J = 5.8 Hz, 6H) ppm; **^{13}C NMR (151 MHz, Chloroform-*d*)** δ 177.9, 141.3, 135.4, 130.9, 129.8, 40.6, 36.5, 35.1, 30.3, 27.8, 23.2, -0.0, -0.2 ppm; **^{29}Si NMR (119 MHz, Chloroform-*d*)** δ -4.00 ppm; **IR(ATR):** $\tilde{\nu}$ 3069, 3048, 2931, 2854, 1628, 1479, 1419, 1365, 1264, 1249, 1199, 1152, 1137, 1113, 1021, 972, 836, 785, 730, 702 cm^{-1}; **HRMS (ESI)** calculated $[M+H]^+$ for $C_{19}H_{32}ONSi^+$ = 318.2248, found: 318.2239.

2-((dimethyl-(phenyl)-silyl)-methyl)-piperidine[3]

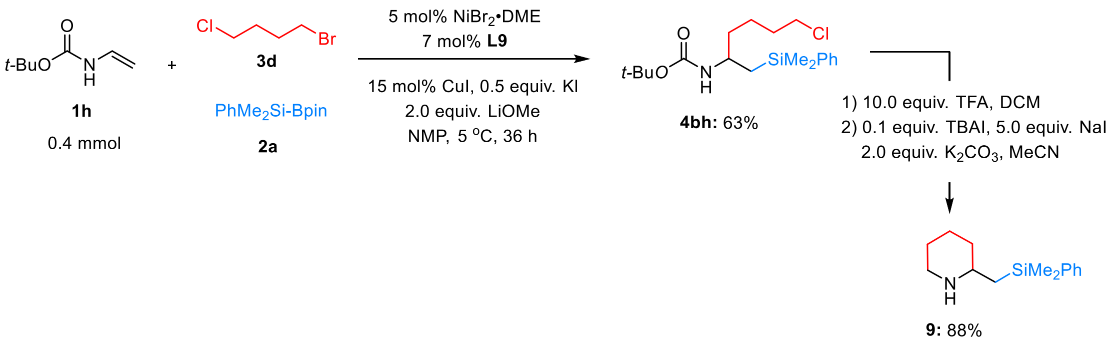

tert-butyl (6-chloro-1-(dimethyl-(phenyl)-silyl)-hexan-2-yl)-carbamate (**4bh**) was synthesized according to general procedure, obtained the pure compound **4bh** as a colorless oil (93.0 mg, 63% yield).

^{1}H NMR (600 MHz, Chloroform-*d*) δ 7.53 - 7.47 (m, 2H), 7.38 - 7.32 (m, 3H), 4.18 (d, J = 9.2 Hz, 1H), 3.80 - 3.66 (m, 1H), 3.49 - 3.40 (m, 2H), 1.76 - 1.60 (m, 2H), 1.46 - 1.34 (m, 13H), 1.07 - 0.90 (m, 2H), 0.32 (s, 6H) ppm; **^{13}C NMR (151 MHz, Chloroform-*d*)** δ 155.1, 139.3, 133.6, 129.1, 128.0, 79.0, 47.8, 45.1, 38.4, 32.3, 28.5, 23.4, 23.1, -2.5, -2.6 ppm; **^{29}Si NMR (79 MHz, Chloroform-*d*)** δ -4.10 ppm; **HRMS (ESI)** calculated $[M-C_6H_5]^+$ for $C_{13}H_{27}O_2NClSi^+$ = 292.1494, found: 292.1483.

To an oven-dried round bottom flask containing a stirring bar was added a solution of **4bh** (75.1 mg, 0.2 mmol, 1.0 equiv) in DCM (2.0 mL) and TFA (152 µL, 2 mmol, 10.0 equiv). The reaction mixture was stirred for 20 h under 20 °C. Then the reaction was concentrated under reduced pressure and used for the next reaction without further purification. To a solution of the crude mixture obtained above in MeCN (5.0 mL) was added TBAI (7.5 mg, 0.02 mmol, 0.1 equiv), NaI (150 mg, 1.0 mmol, 5.0 equiv) and K_2CO_3 (55.3 mg, 0.4 mmol, 2.0 equiv). The resulting mixture was refluxed overnight and quenched by saturated aqueous solution of NaCl. The mixture was extracted with EtOAc, dried over Na_2SO_4, concentrated under reduced pressure. The mixture was purified by flash column chromatography to provide the title compound **9** as a yellow solid (41.1 mg, 88% yield).

^{1}H NMR (600 MHz, Chloroform-*d*) δ 8.55 (s, 1H), 8.10 (s, 1H), 7.57 - 7.48 (m, 2H), 7.41 - 7.33 (m, 3H), 3.44 - 3.37 (m, 1H), 3.28 - 3.21 (m, 1H), 2.79 (dtd, J = 12.9, 10.2, 2.7 Hz, 1H), 2.01 - 1.93 (m, 1H), 1.82 - 1.72 (m, 4H), 1.63 (tdd, J = 14.5, 11.2, 3.7 Hz, 1H), 1.51 (dd, J = 13.9, 12.4 Hz, 1H), 1.36 - 1.28 (m, 1H), 0.36 (d, J = 5.2 Hz, 6H) ppm; **^{13}C NMR (151 MHz,**

Chloroform-*d*) δ 136.9, 133.7, 129.6, 128.2, 56.5, 44.5, 30.5, 22.3, 22.0, 21.5, -1.9, -2.2 ppm; **^{29}Si NMR (119 MHz, Chloroform-*d*)** δ -4.70 ppm; **IR(ATR):** $\tilde{\nu}$ 3392, 3140, 2951, 2823, 2800, 1570, 1456, 1426, 1416, 1303, 1251, 1233, 1173, 1115, 1066, 1001, 907, 824, 815, 728, 701 cm^{-1}; **HRMS (ESI)** calculated [M+H]$^+$ for $C_{14}H_{24}NSi^+$ = 234.1673, found: 234.1667.

Alpha-Lipoic Acid[4-6]

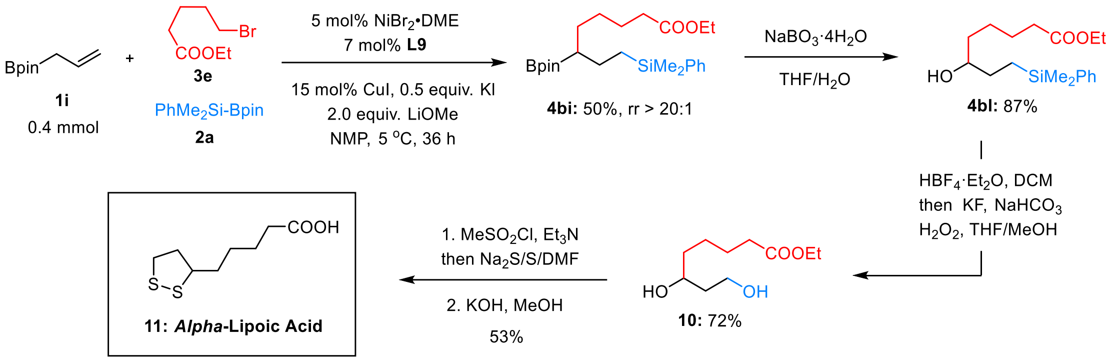

Ethyl-8-(dimethyl-(phenyl)-silyl)-6-(4,4,5,5-tetramethyl-1,3,2-dioxaborolan-2-yl)-octan-oate was synthesized according to general procedure, obtained the pure compound **4bi** as a colorless oil (86.5 mg, 50% yield, rr > 20:1).

^{1}H NMR (400 MHz, Chloroform-d) δ 7.52 - 7.46 (m, 2H), 7.34 - 7.30 (m, 3H), 4.09 (q, *J* = 7.1 Hz, 2H), 2.25 (t, *J* = 7.6 Hz, 2H), 1.59 (p, *J* = 7.5 Hz, 2H), 1.49 - 1.32 (m, 4H), 1.31 - 1.26 (m, 2H), 1.25 - 1.21 (m, 15H), 1.00 - 0.86 (m, 1H), 0.78 - 0.68 (m, 2H), 0.23 (s, 6H) ppm; **^{13}C NMR (101 MHz, Chloroform-d)** δ 174.0, 139.8, 133.7, 128.8, 127.8, 83.0, 60.2, 34.5, 30.7, 28.9, 25.4, 25.0, 24.9, 15.2, 14.4, -2.9, -3.0 ppm; **^{29}Si NMR (79 MHz, Chloroform-d)** δ -2.62 ppm; **IR(ATR):** $\tilde{\nu}$ 3069, 2978, 2928, 2856, 1737, 1463, 1387, 1371, 1314, 1248, 1145, 1113, 1034, 968, 837, 815, 777, 730, 701 cm^{-1}; **HRMS (ESI)** calculated [M+NH$_4$]$^+$ for $C_{24}H_{45}O_4NBSi^+$ = 450.3205, found: 450.3200.

The **4bi** (64.5 mg, 0.2 mmol, 1.0 equiv) and $NaBO_3 \cdot 4H_2O$ (126.8 mg, 0.8 mmol, 4.0 equiv) were dissolved in THF and H_2O (2mL, 1:1). After stirred at ambient temperature 16 h, the reaction was quenched by saturated aqueous solution of NaCl (5 mL). The mixture was extracted with EtOAc, dried over Na_2SO_4, concentrated under reduced pressure. The mixture was purified by flash column chromatography to provide the compound **4bl** as a colorless oil (56.1 mg, 87% yield).

^{1}H NMR (400 MHz, Chloroform-*d*) δ 7.54 - 7.46 (m, 2H), 7.37 - 7.32 (m, 3H), 4.11 (q, *J* = 7.1 Hz, 2H), 3.53 - 3.45 (m, 1H), 2.29 (t, *J* = 7.5 Hz, 2H), 1.68 - 1.56 (m, 3H), 1.52 - 1.30 (m, 6H), 1.24 (t, *J* = 7.1 Hz, 3H), 0.87 (ddd, *J* = 14.1, 12.6, 4.8 Hz, 1H), 0.68 (ddd, *J* = 14.2, 12.3, 5.0 Hz, 1H), 0.27 (s, 6H) ppm; **^{13}C NMR (101 MHz, $CDCl_3$)** δ 173.9, 139.2, 133.6, 129.0, 127.9, 73.8, 60.3, 36.3, 34.4, 31.6, 25.3, 25.0, 14.4, 11.3, -3.1 ppm; **^{29}Si NMR (79 MHz, Chloroform-d)** δ -1.90 ppm; **HRMS (ESI)** calculated $[M-C_6H_5]^+$ for $C_{12}H_{25}O_3Si^+$ = 245.1568, found: 245.1559.

To a solution of ethyl 8-(dimethyl(phenyl)silyl)-6-hydroxyoctanoate (51.2 mg, 0.16 mmol, 1.0 equiv) in DCM (1.6 mL) under N_2 atmosphere was added $HBF_4 \cdot Et_2O$ (87 μL, 50% in Et_2O, 0.32 mmol, 2.0 equiv) by dropping at 0 °C, the mixture was stirred at this temperature 1 h. The resulting mixture was quenched with saturated aqueous solution of $NaHCO_3$, the aqueous layer was extracted with DCM, dried over Na_2SO_4, concentrated under reduced pressure, which was used directly without further purification. To a solution of the crude fluorosilane in THF (1.6 mL) and MeOH (1.6 mL) was added KF (55.8 mg, 0.96 mmol, 6.0 equiv) and $NaHCO_3$ (80.7 mg, 0.96 mmol, 6.0 equiv). After being cooled to 0 °C, 30% H_2O_2 solution was added dropwise and the resulting mixture was stirred at 30 °C for 3 h. Then the reaction was quenched with saturated aqueous solution of $Na_2S_2O_3$ at 0 °C and extracted with EtOAc (3 ×5 mL), dried over Na_2SO_4, concentrated under reduced pressure. The mixture was purified by flash column chromatography to provide the title compound **10** as a colorless oil (23.5 mg, 72% yield).

^{1}H NMR (600 MHz, Chloroform-*d*) δ 4.13 (q, *J* = 7.1 Hz, 2H), 3.92 - 3.85 (m, 2H), 3.85 - 3.80 (m, 1H), 2.75 (s, 2H), 2.32 (t, *J* = 7.4 Hz, 2H), 1.75 - 1.60 (m, 4H), 1.56 - 1.43 (m, 3H), 1.43 - 1.34 (m, 1H), 1.26 (t, *J* = 7.1 Hz, 3H) ppm; **^{13}C NMR (151 MHz, $CDCl_3$)** δ 174.0, 72.0, 61.9, 60.5, 38.4, 37.4, 34.3, 25.1, 24.9, 14.4 ppm; **IR(ATR):** $\tilde{\nu}$ 3376, 2939, 2867, 1732, 1716, 1446, 1374, 1188, 1098, 1055 cm^{-1}; **HRMS (ESI)** calculated $[M+H]^+$ for $C_{10}H_{21}O_4^+$ = 205.1434, found: 205.1430.

To a solution of diol **10** (85.4 mg, 0.42 mmol, 1.0 equiv) in anhydrous DCM (2.0 mL) under N_2 atmosphere was added Et_3N (17 μL, 0.84 mmol, 2.0 equiv) and MsCl (83 μL, 0.84 mmol, 2.0 equiv) by dropping at 0 °C, the mixture was stirred at room temperature for 4 h. The reaction mixture was poured into saturated aqueous solution of NaCl, the aqueous layer was extracted

with DCM, dried over Na_2SO_4, concentrated under reduced pressure, which was rapidly purified by silico gel column chromatography. Under N_2 atmosphere, an oven-dried reaction tube which equipped with a magnetic stir bar was added the half of mixture of dimesylate, Na_2S (21.9 mg, 0.25 mmol, 1.2 equiv) and S (8.1 mg, 0.25 mmol, 1.2 equiv) in anhydrous DMF (1.1 mL). The resulting mixture was heated at 90 °C for 18 h. Then the reaction was quenched with saturated aqueous solution of NaCl at room temperature and extracted with EtOAc (3 × 5 mL), dried over Na_2SO_4, concentrated under reduced pressure, which was used directly without further purification. To a solution of dithiolane in MeOH (2.1 mL) was added aqueous KOH (0.1 M, 3.0 mL) and stirred at 30 °C for 20 h. The mixture was acidified carefully with 1N HCl to pH 2. MeOH and Water were evaporated under reduced pressure. Then the mixture was purified by column chromatography to provide the title compound **11** as a yellow solid (23.0 mg, 53% yield).

^{1}H NMR (600 MHz, Chloroform-*d*) δ 3.62 - 3.51 (m, 1H), 3.26 - 3.05 (m, 2H), 2.51 - 2.42 (m, 1H), 2.39 - 2.35 (m, 2H), 1.92 (dq, J = 13.6, 6.9 Hz, 1H), 1.73 - 1.63 (m, 4H), 1.55 - 1.47 (m, 2H) ppm; **^{13}C NMR (151 MHz, Chloroform-*d*)** δ 178.2, 55.4, 39.4, 37.6, 32.8, 32.8, 27.8, 23.5, 23.4, 0.1 ppm; **IR(ATR):** $\tilde{\nu}$ 2922, 2851, 1704, 1456, 1439, 1406, 1300, 1277, 1265, 1203, 1130, 960 cm^{-1}; **HRMS (ESI)** calculated $[M+H]^+$ for $C_8H_{15}O_2S_2^+$ = 207.0508, found: 207.0503.

1.6 Non-reactive and Inefficient Substrates

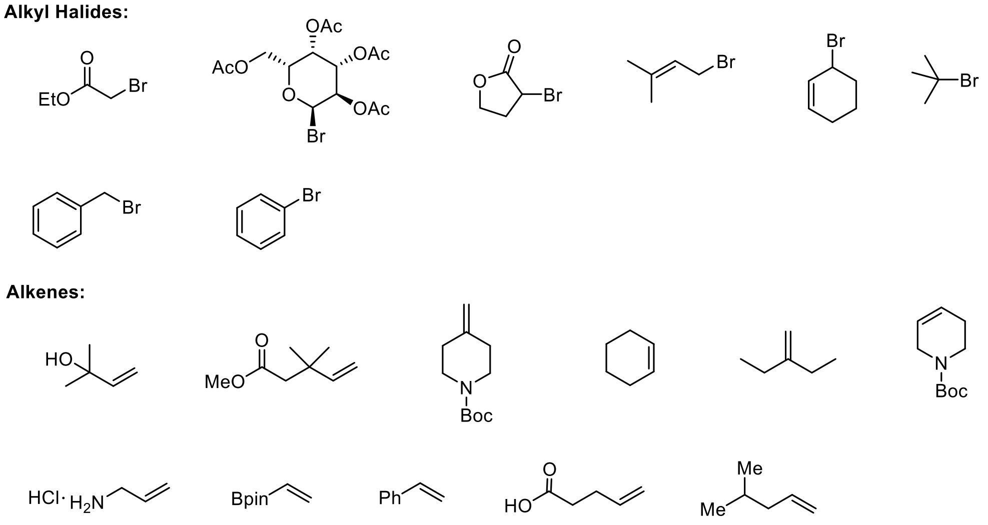

1.7 Mechanism Studies

1.7.1 Control Experiments

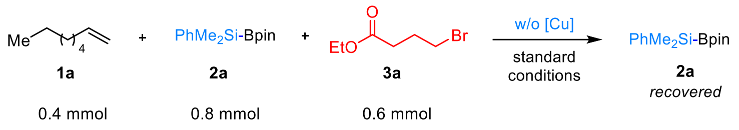

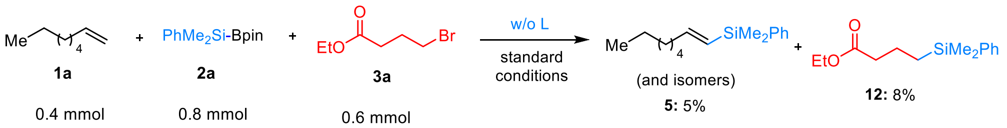

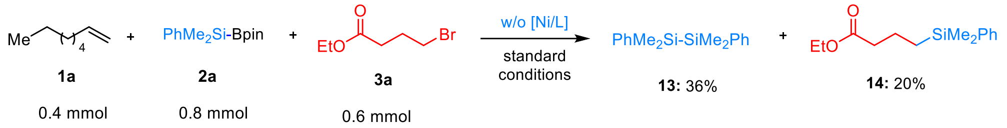

1.7.2 Radical Clock Experiment

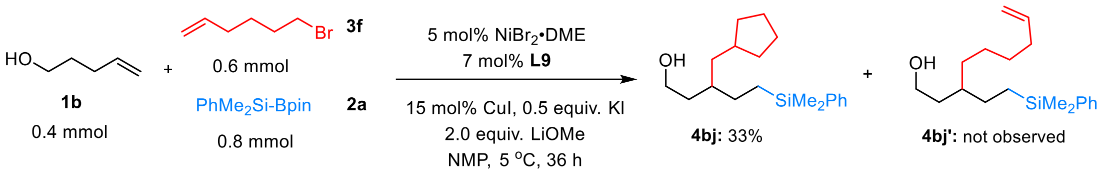

Following the General Procedure, the crude product was purified by flash column chromatography on silica gel to afford **4bj** as a colorless oil (44.0 mg, 33%).

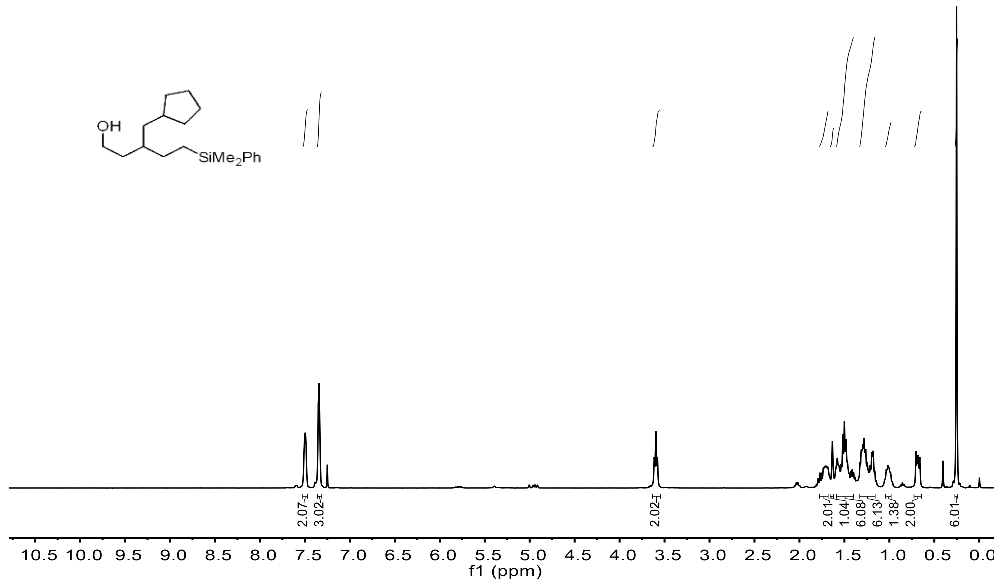

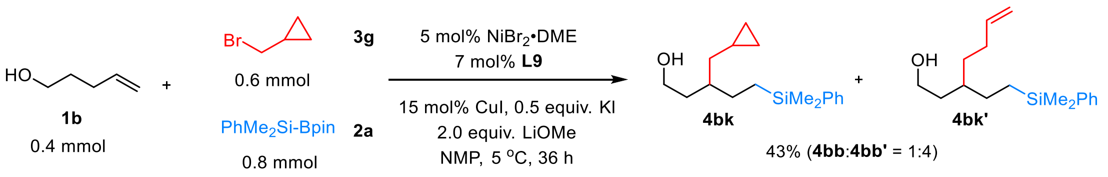

Following the General Procedure, the crude product was purified by flash column chromatography on silica gel to afford the mixture of **4bl** and **4bl'** as colorless oil (47.6 mg, 43%, rr = 1:4).

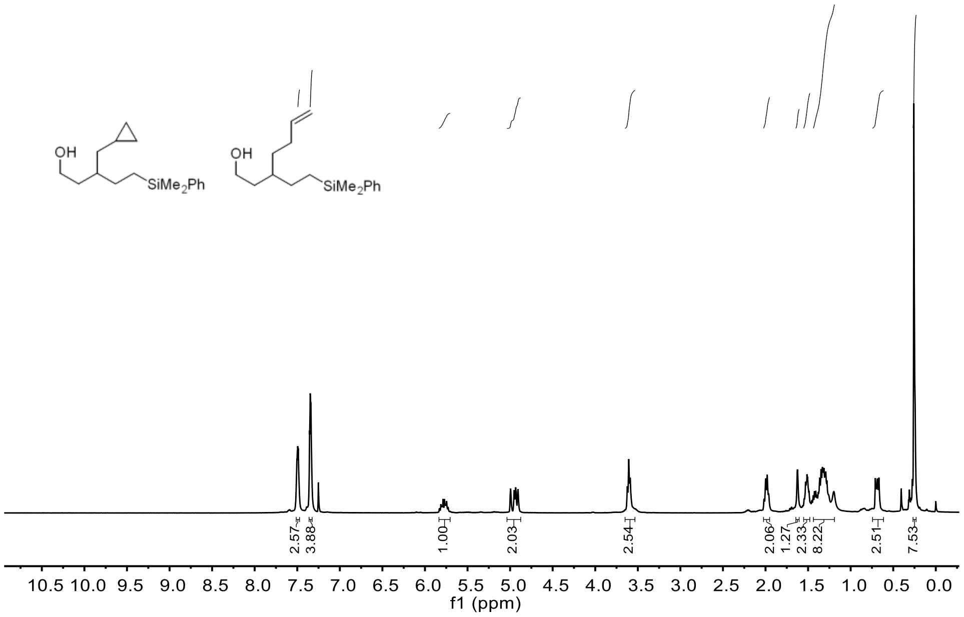

1.8 Identification of product structure

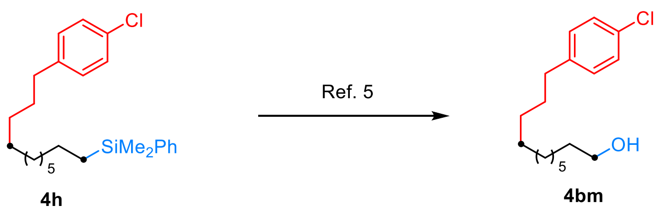

11-(4-chlorophenyl)undecan-1-ol (4bm); ^{1}H NMR (600 MHz, Chloroform-*d*) δ 7.24 - 7.20 (m, 2H), 7.12 - 7.07 (m, 2H), 3.64 (t, J = 6.6 Hz, 2H), 2.64 - 2.49 (m, 2H), 1.56 - 1.52 (m, 3H), 1.38 - 1.23 (m, 16H) ppm; **^{13}C NMR (151 MHz, Chloroform-*d*)** δ 141.5, 131.4, 129.9, 128.4, 63.3, 35.4, 32.9, 31.5, 29.72, 29.69, 29.67, 29.59, 29.56, 29.3, 25.9 ppm; **IR(ATR):** $\tilde{\nu}$ 3347，

2927, 2854, 1718, 1492, 1463, 1092, 1057, 1016, 803, 721 cm^{-1}; **HRMS (ESI)** calculated [M-OH]$^+$ for $C_{17}H_{26}Cl^+$ = 265.1718, found: 265.1725.

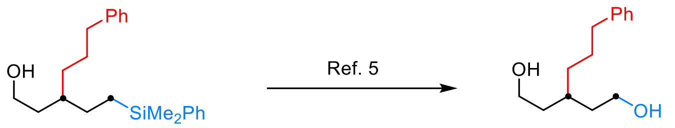

4aa 4bn

3-(3-phenylpropyl)pentane-1,5-diol (4bn); ^{1}H NMR (600 MHz, Chloroform-*d*) δ 7.31 - 7.22 (m, 2H), 7.20 - 7.14 (m, 3H), 3.67 (ddt, *J* = 28.9, 10.6, 6.5 Hz, 4H), 2.60 (t, *J* = 7.7 Hz, 2H), 1.84 (s, 2H), 1.69 - 1.60 (m, 3H), 1.59 - 1.49 (m, 4H), 1.38 - 1.32 (m, 2H) ppm; **^{13}C NMR (151 MHz, Chloroform-*d*)** δ 142.7, 128.5, 128.4, 125.8, 61.0, 36.7, 36.3, 34.2, 31.2, 28.5 ppm; **IR(ATR):** $\tilde{\nu}$ 3332, 3027, 2931, 2858, 1704, 1631, 1496, 1453, 1054, 1010, 909, 748, 699 cm^{-1}; **HRMS (ESI)** calculated [M+H]$^+$ for $C_{14}H_{23}O_2^+$ = 223.1693, found: 223.1690.

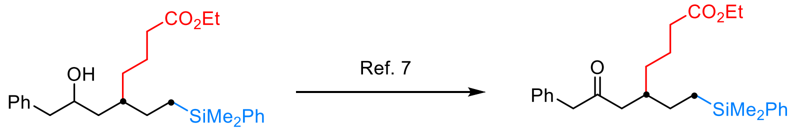

4ag 4bo

ethyl 5-(2-(dimethyl(phenyl)silyl)ethyl)-7-oxo-8-phenyloctanoate (4bo); ^{1}H NMR (600 MHz, Chloroform-*d*) δ 7.47 - 7.43 (m, 2H), 7.37 - 7.28 (m, 5H), 7.25 - 7.23 (m, 1H), 7.18 - 7.14 (m, 2H), 4.10 (q, *J* = 7.1 Hz, 2H), 3.63 (s, 2H), 2.36 (qd, *J* = 16.7, 6.6 Hz, 2H), 2.20 (t, *J* = 7.5 Hz, 2H), 1.94 - 1.81 (m, 1H), 1.50 - 1.42 (m, 2H), 1.27 - 1.21 (m, 5H), 1.21 - 1.13 (m, 2H), 0.57 (dd, *J* = 9.6, 7.8 Hz, 2H), 0.22 (s, 3H), 0.21 (s, 3H) ppm; **^{13}C NMR (151 MHz, Chloroform-*d*)** δ 208.4, 173.8, 139.3, 134.4, 133.7, 129.6, 129.0, 128.9, 127.9, 127.1, 60.4, 50.8, 46.1, 35.9, 34.6, 32.8, 27.5, 22.1, 14.4, 11.9, -3.07, -3.10 ppm; **^{29}Si NMR (119 MHz, Chloroform-*d*)** δ -1.94 ppm; **IR(ATR):** $\tilde{\nu}$ 3067, 2951, 2921, 2851, 1732, 1602, 1496, 1454, 1426, 1371, 1248, 1181, 1113, 1030, 837, 814, 731, 701 cm^{-1}; **HRMS (ESI)** calculated [M+H]$^+$ for $C_{26}H_{37}O_3^+$ = 425.2507, found: 425.2503.

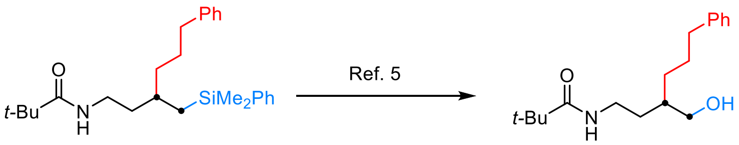

4au 4bp

N-(3-(hydroxymethyl)-6-phenylhexyl)-pivalamide (4bp); ^{1}H NMR (600 MHz, Chloroform-*d*) δ 7.29 - 7.23 (m, 2H), 7.20 - 7.14 (m, 3H), 6.12 (t, *J* = 6.0 Hz, 1H), 3.76 (ddd, *J* = 10.7, 7.0, 5.1 Hz, 1H), 3.66 (ddd, *J* = 10.8, 6.8, 5.3 Hz, 1H), 3.26 - 3.17 (m, 2H), 2.60 (t, *J* = 7.6 Hz, 2H), 1.73 - 1.63 (m, 2H), 1.63 - 1.55 (m, 1H), 1.47 - 1.40 (m, 1H), 1.38 - 1.27 (m, 3H), 1.16 (s, 9H) ppm; **^{13}C NMR (151 MHz, Chloroform-*d*)** δ 179.0, 142.4, 128.5, 128.4, 125.9, 60.9, 43.1, 38.8, 36.11, 36.05, 34.7, 32.4, 28.6, 27.7 ppm; **IR(ATR):** $\tilde{\nu}$ 3341, 3062, 3026, 2930, 2860, 1636, 1539, 1480, 1454, 1366, 1298, 1212, 1054, 910, 733, 699 cm^{-1}; **HRMS (ESI)** calculated $[M+H]^+$ for $C_{18}H_{30}O_2N^+$ = 292.2271, found: 292.2267.

1.9 NMR Spectra

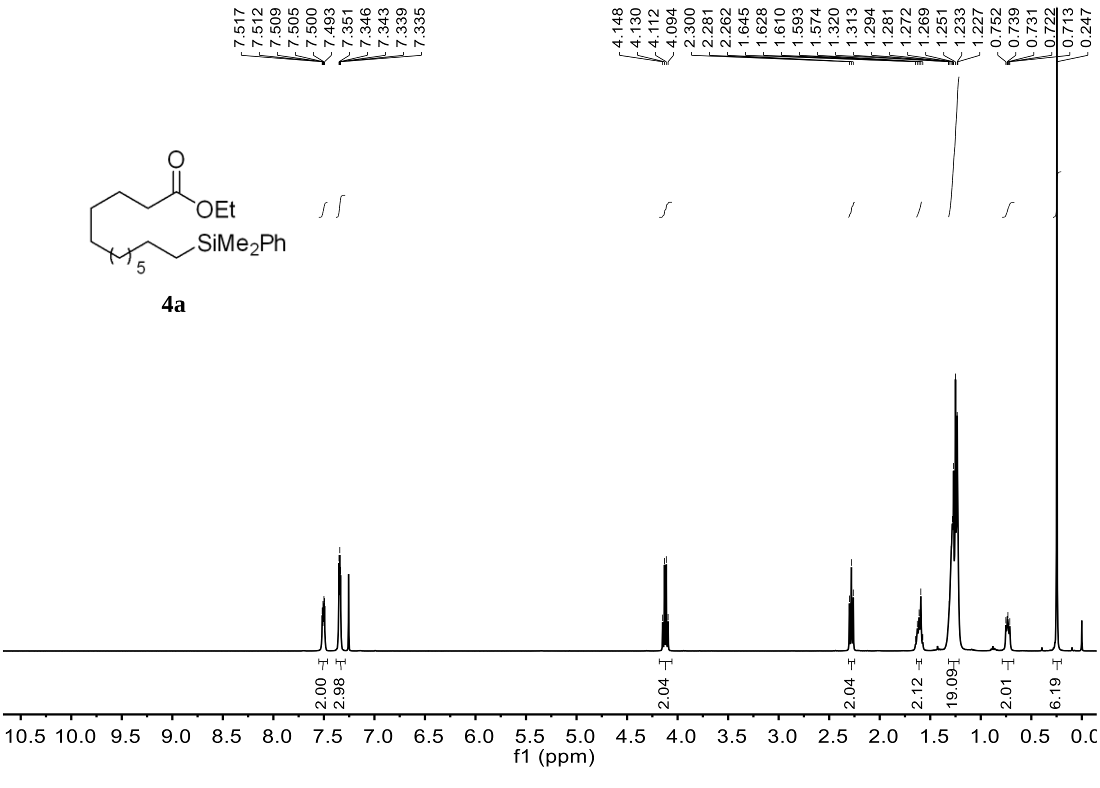

^{1}H NMR Spectrum of **4a**

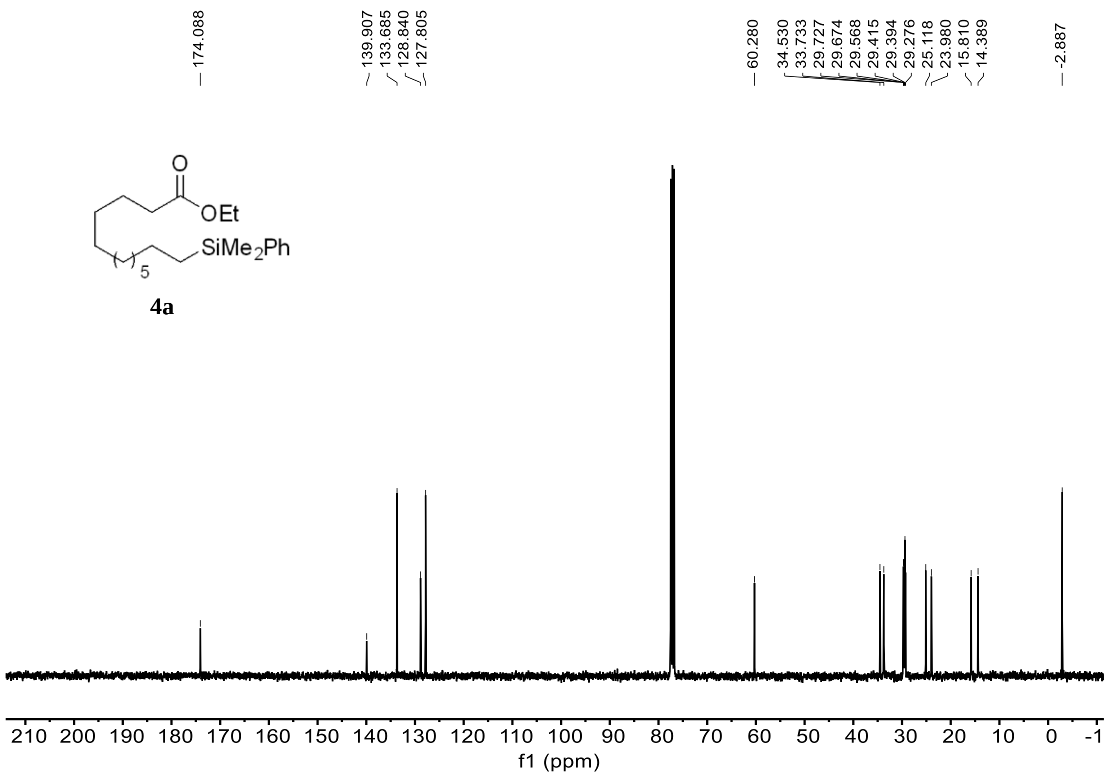

^{13}C NMR Spectrum of **4a**

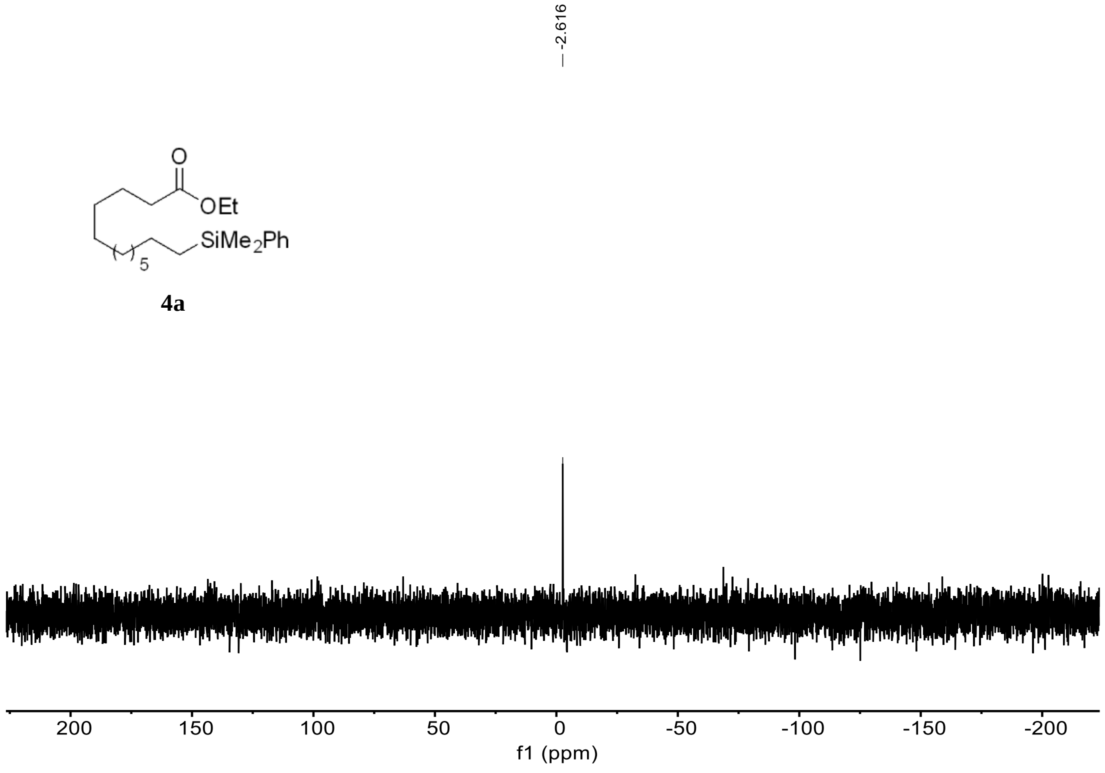

^{29}Si NMR Spectrum of **4a**

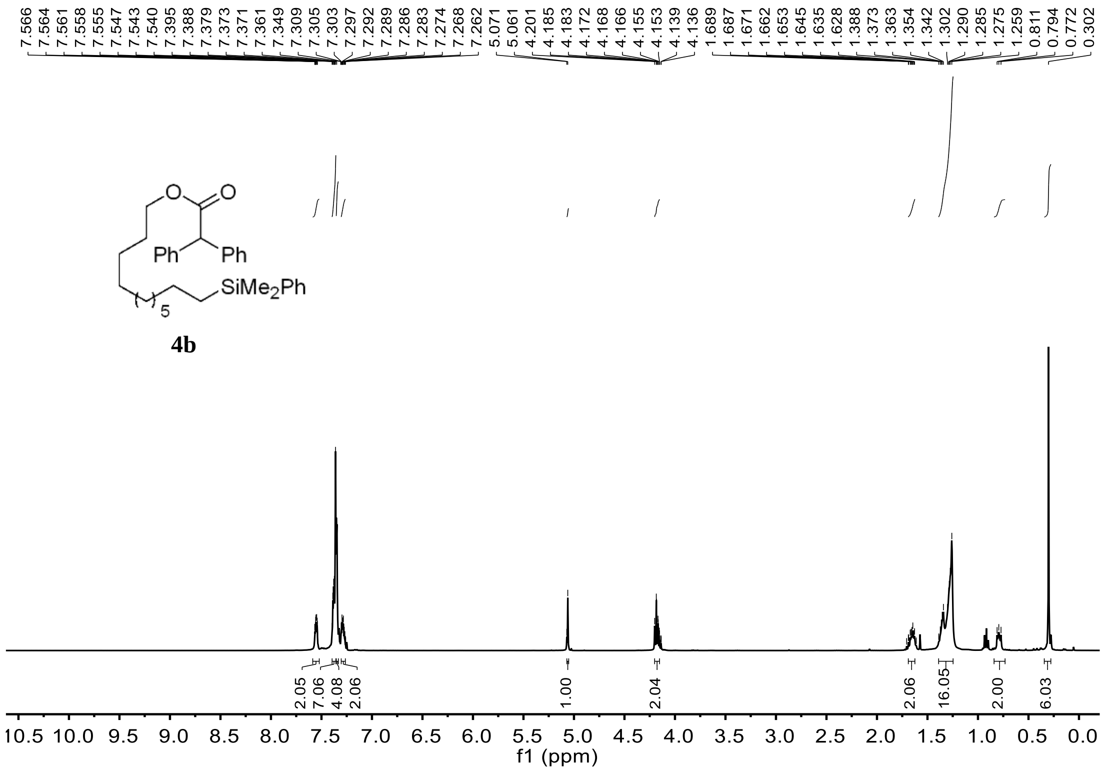

^{1}H NMR Spectrum of **4b**

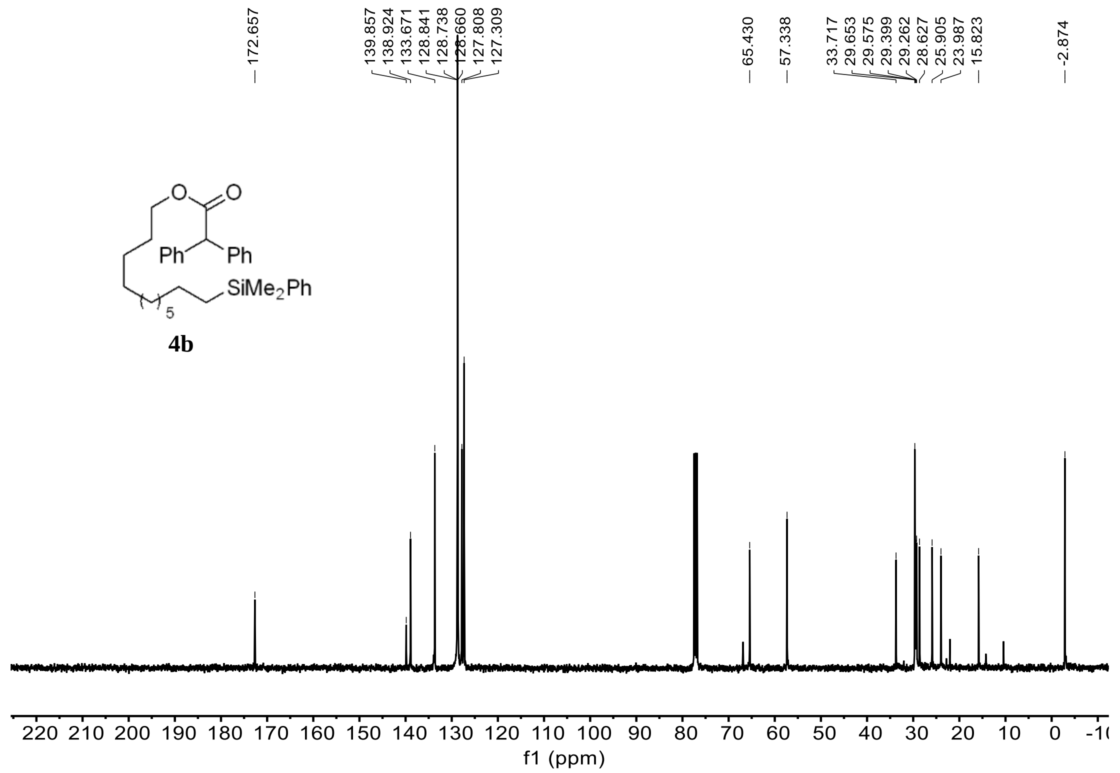

^{13}C NMR Spectrum of **4b**

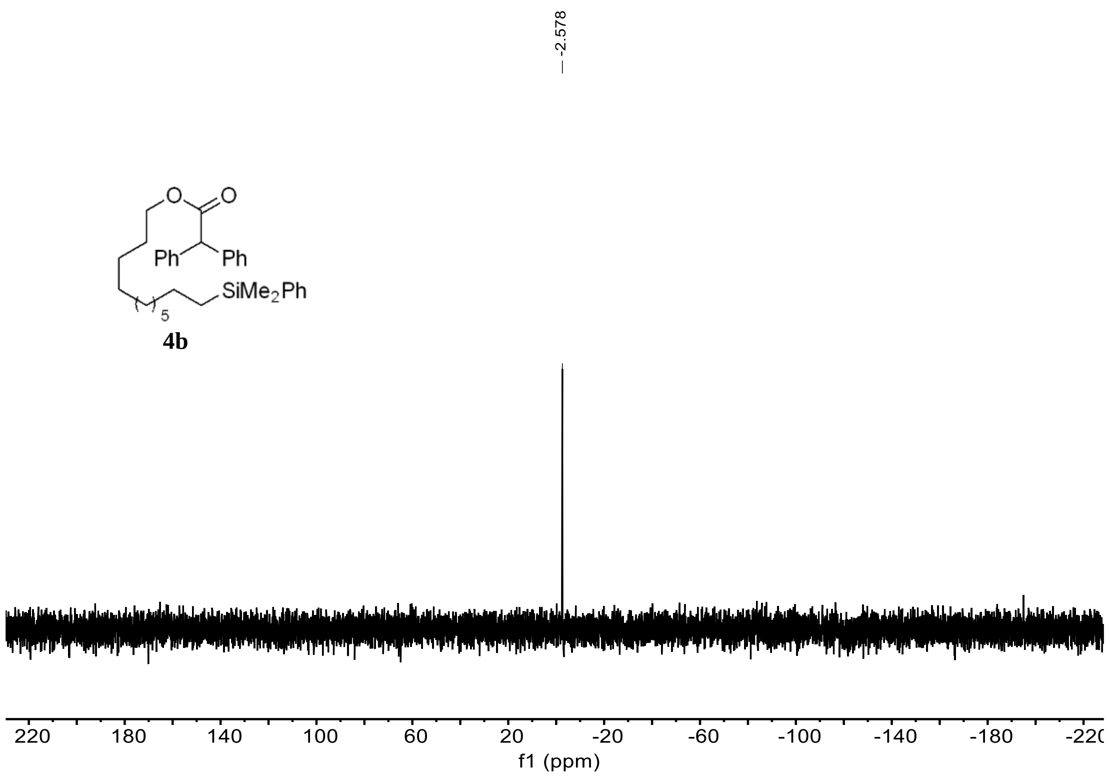

^{29}Si NMR Spectrum of **4b**

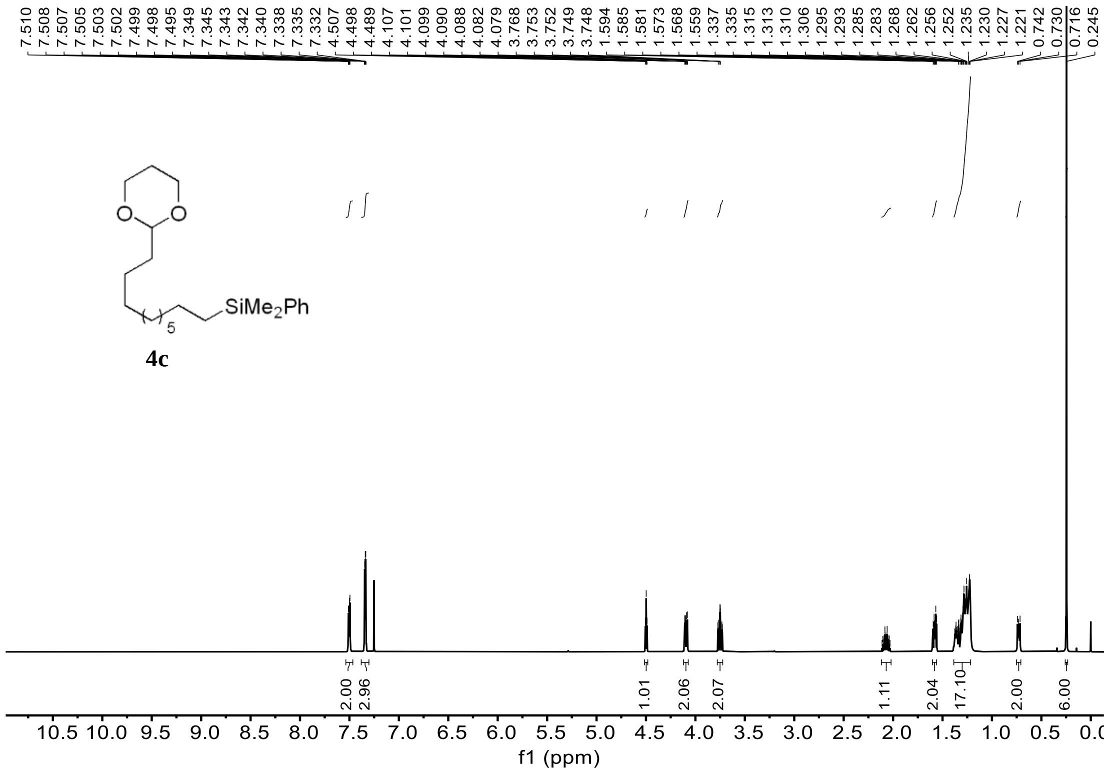

^{1}H NMR Spectrum of **4c**

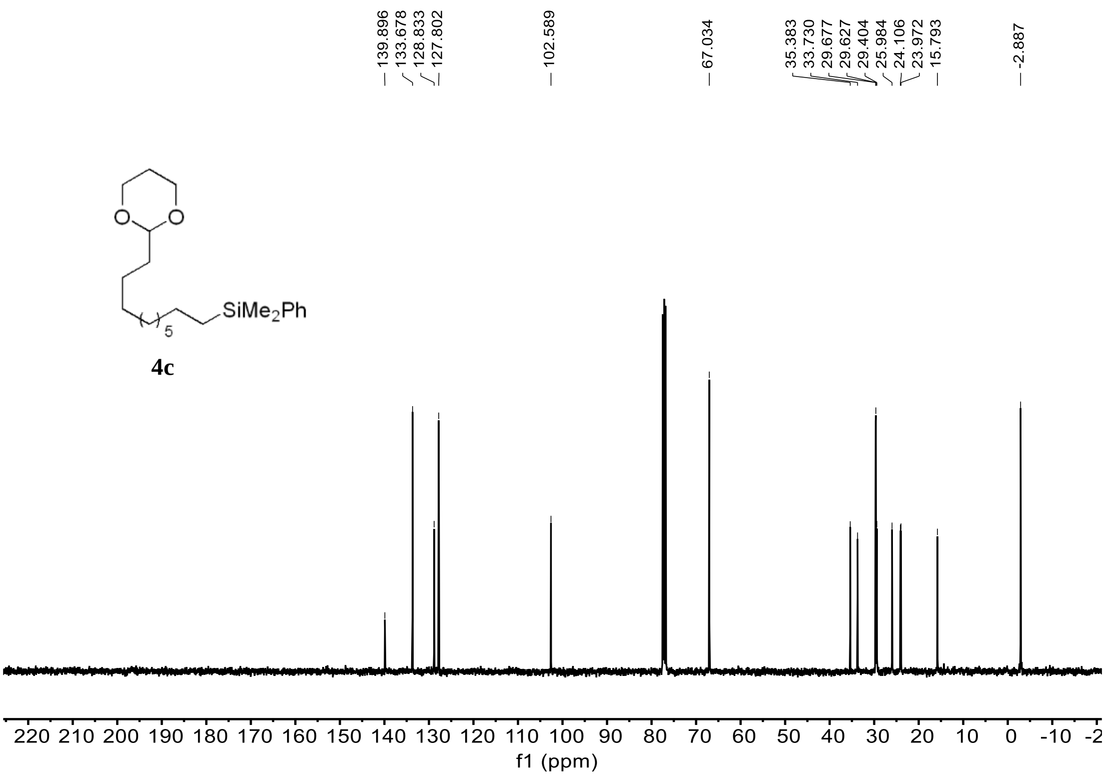

^{13}C NMR Spectrum of **4c**

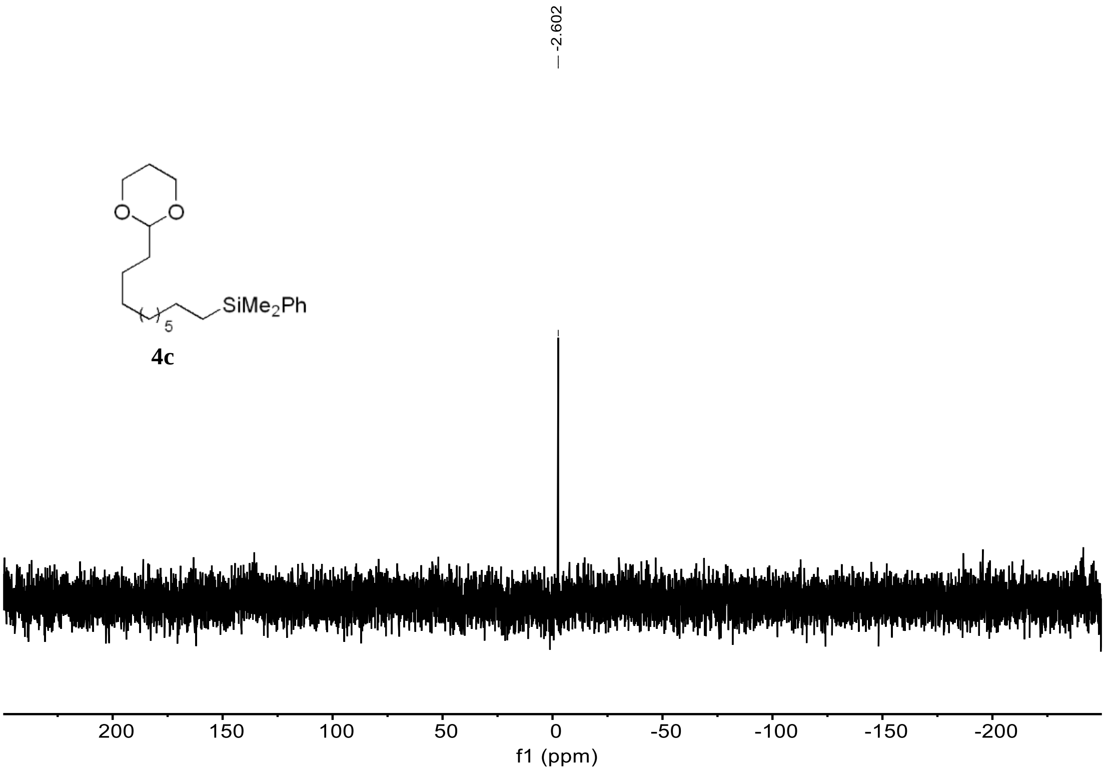

^{29}Si NMR Spectrum of **4c**

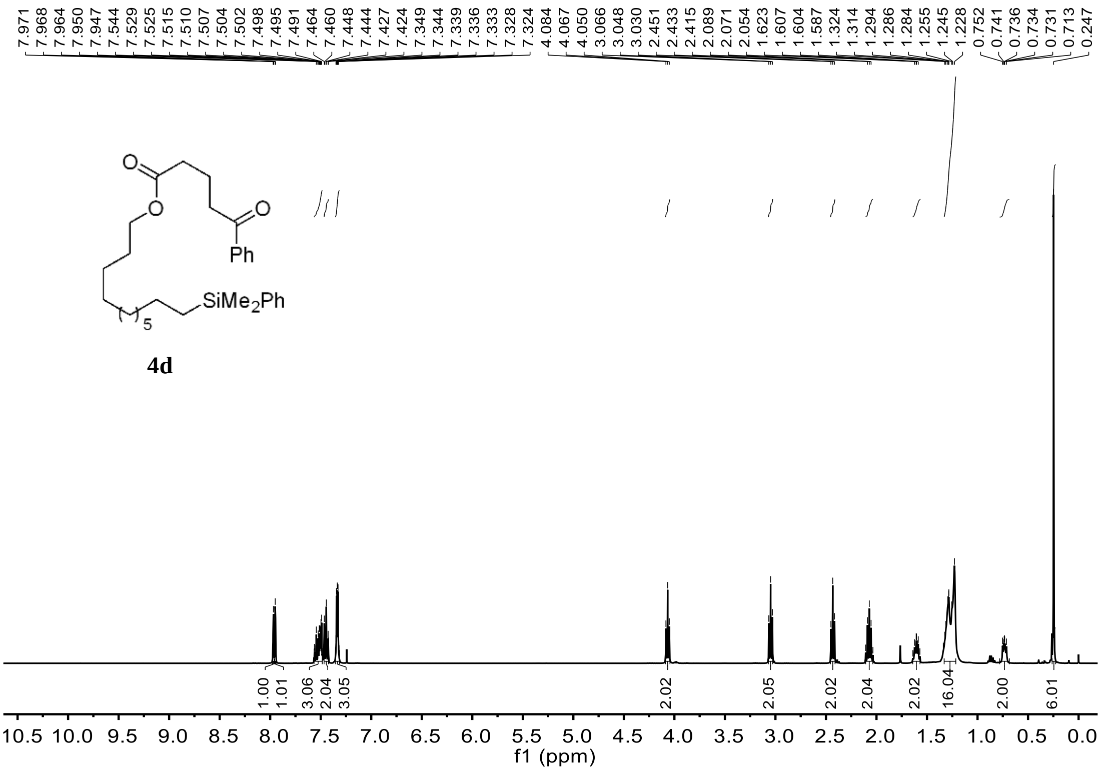

^{1}H NMR Spectrum of **4d**

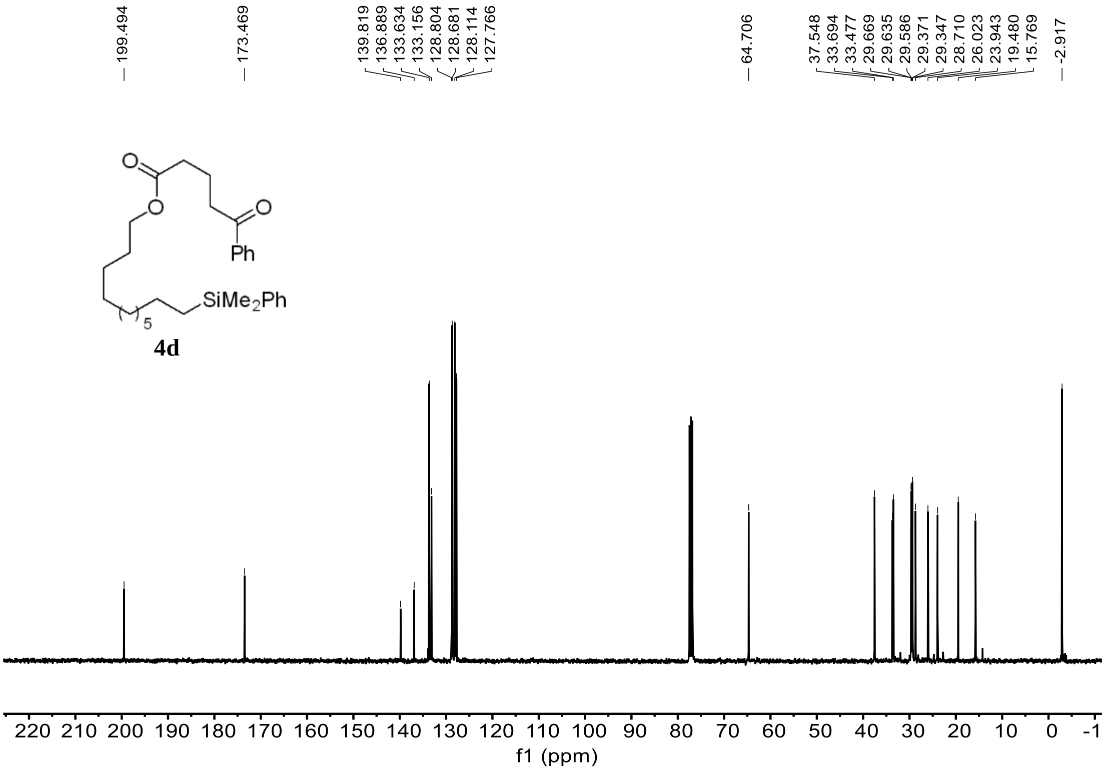

^{13}C NMR Spectrum of **4d**

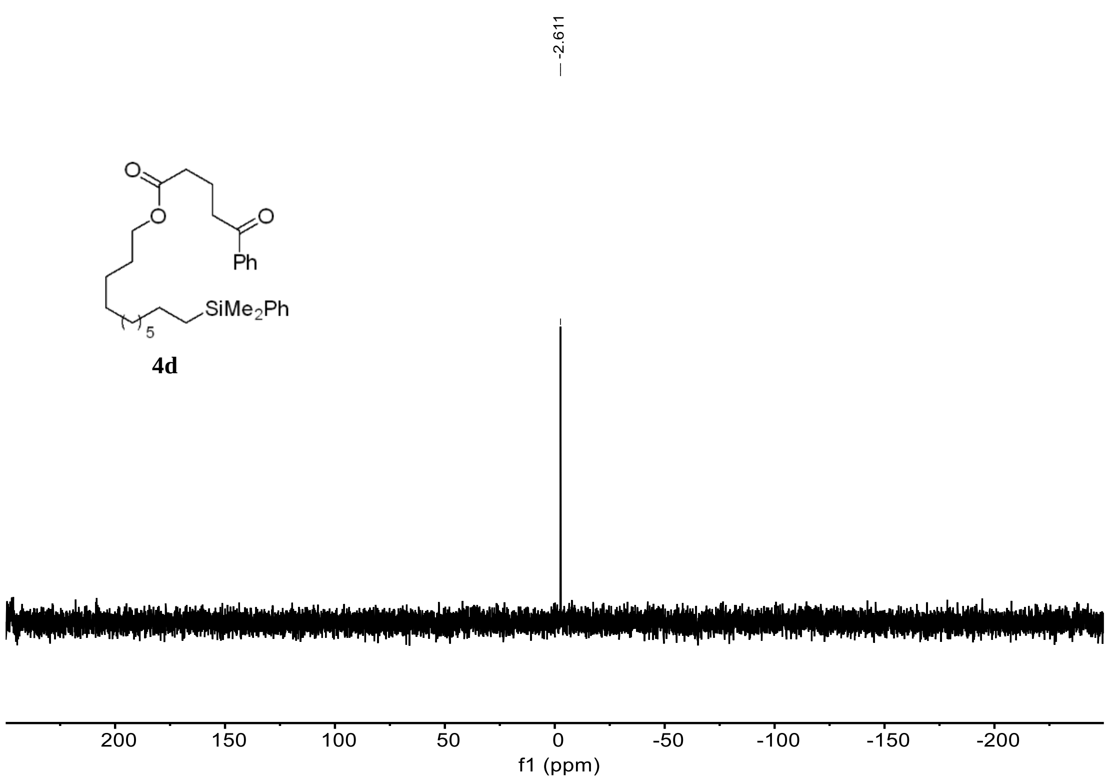

4d

^{29}Si NMR Spectrum of **4d**

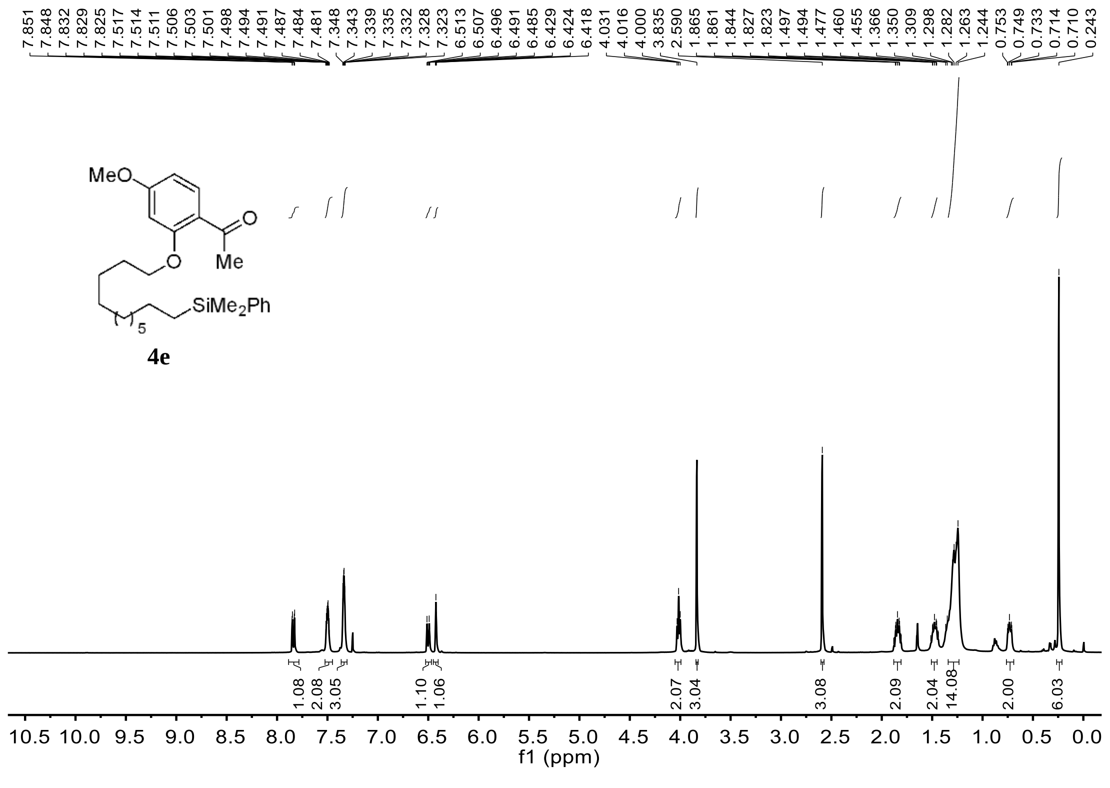

4e

^{1}H NMR Spectrum of **4e**

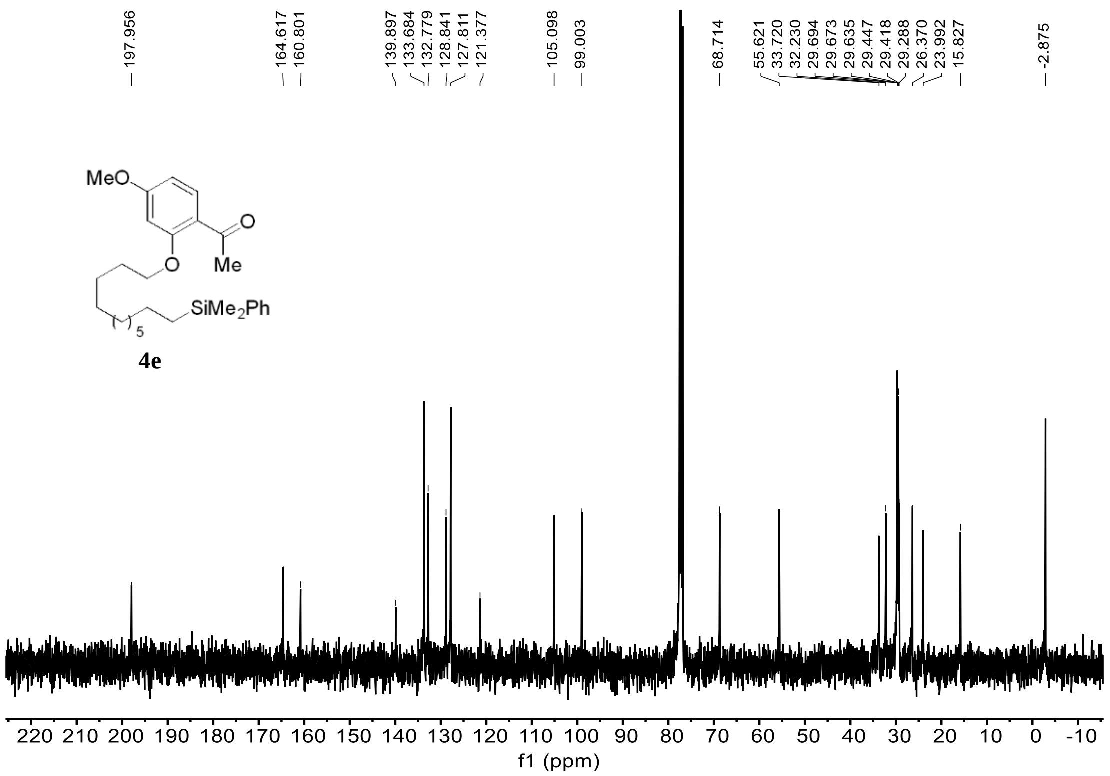

^{13}C NMR Spectrum of **4e**

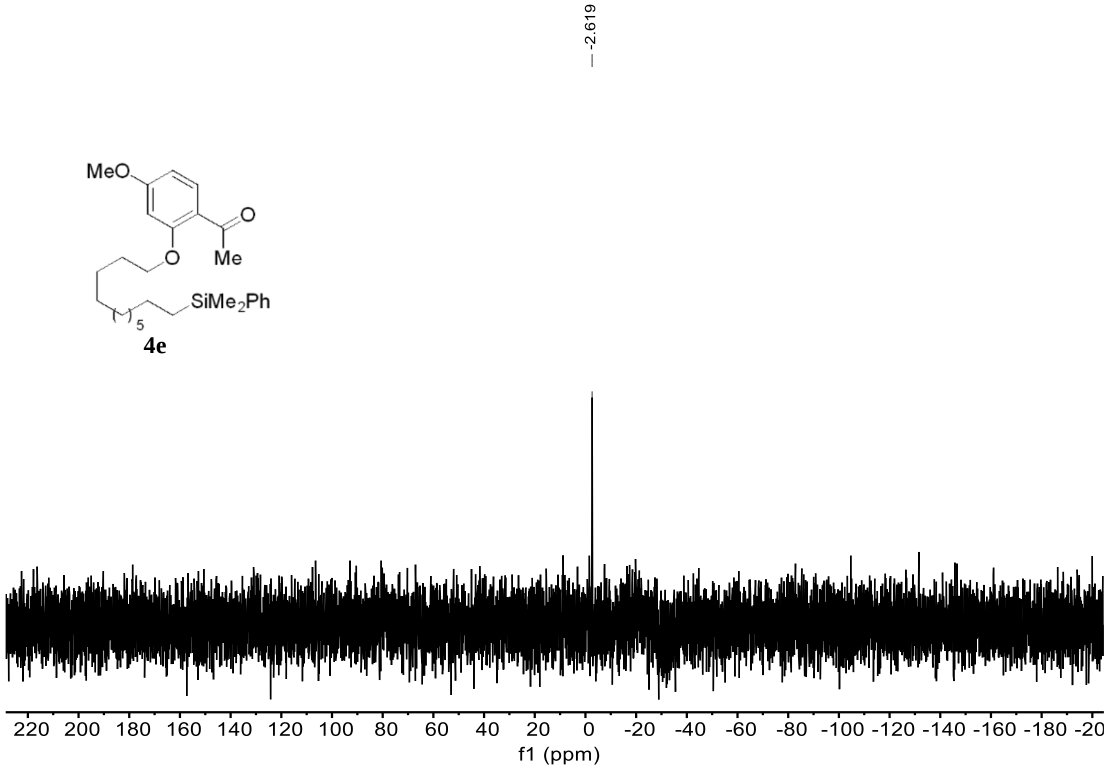

^{29}Si NMR Spectrum of **4e**

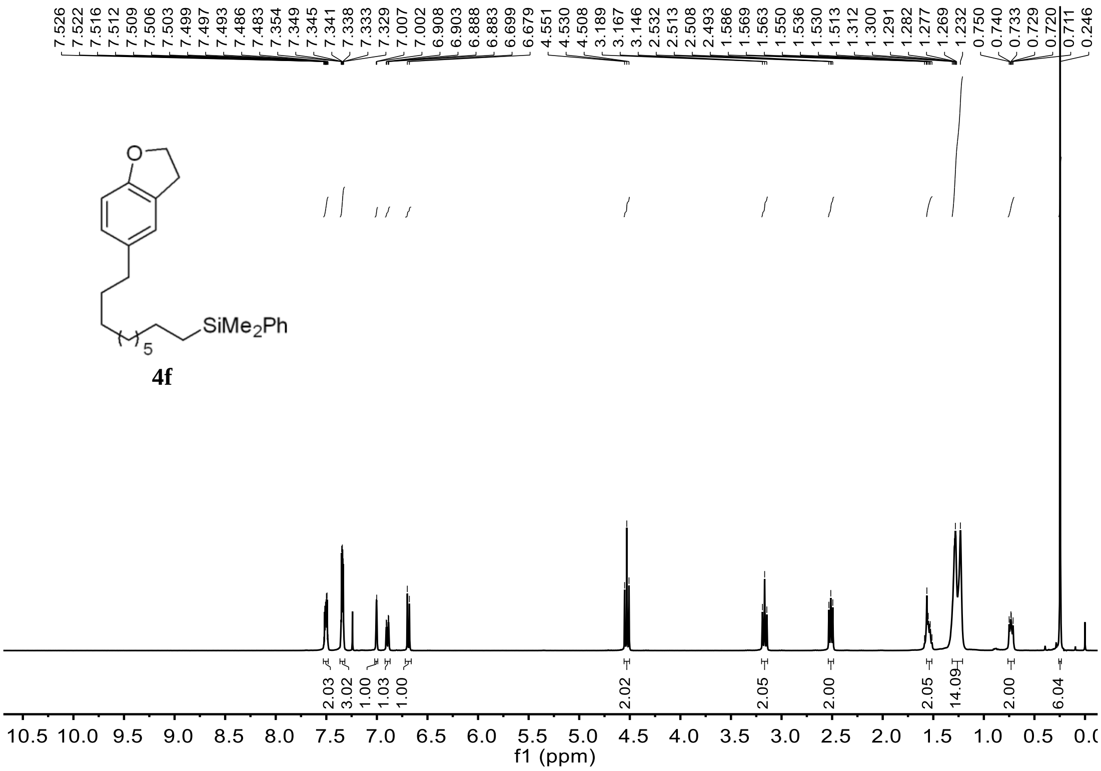

^{1}H NMR Spectrum of **4f**

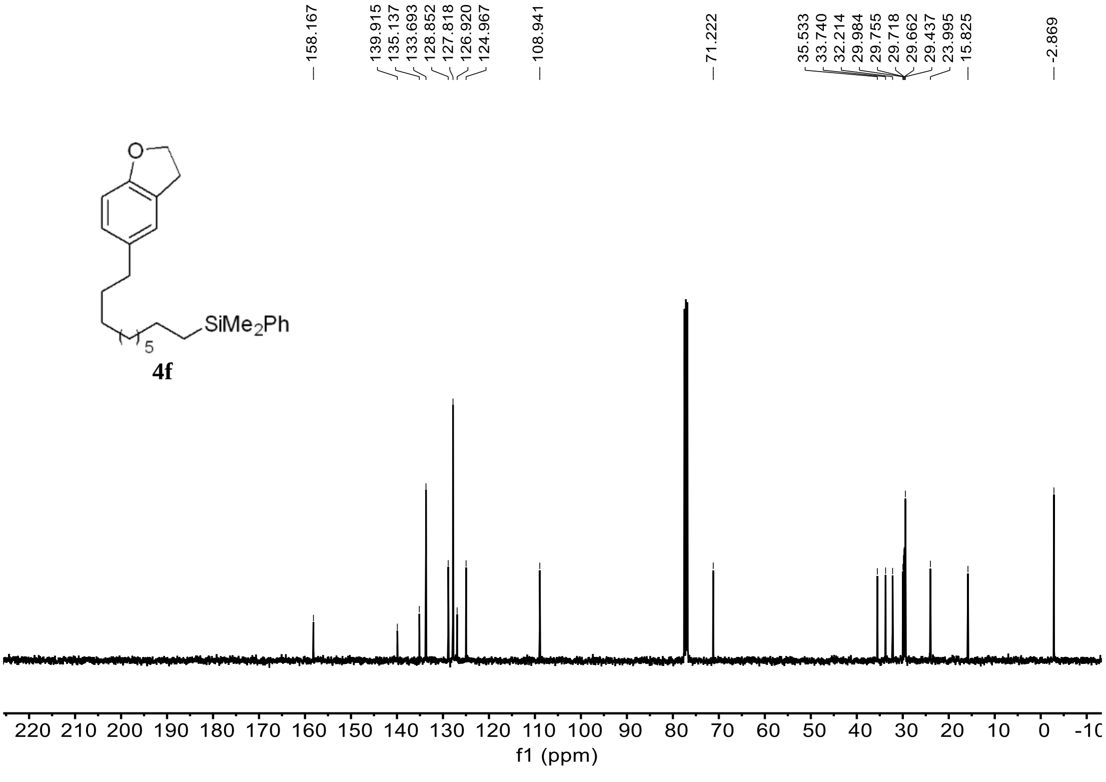

^{13}C NMR Spectrum of **4f**

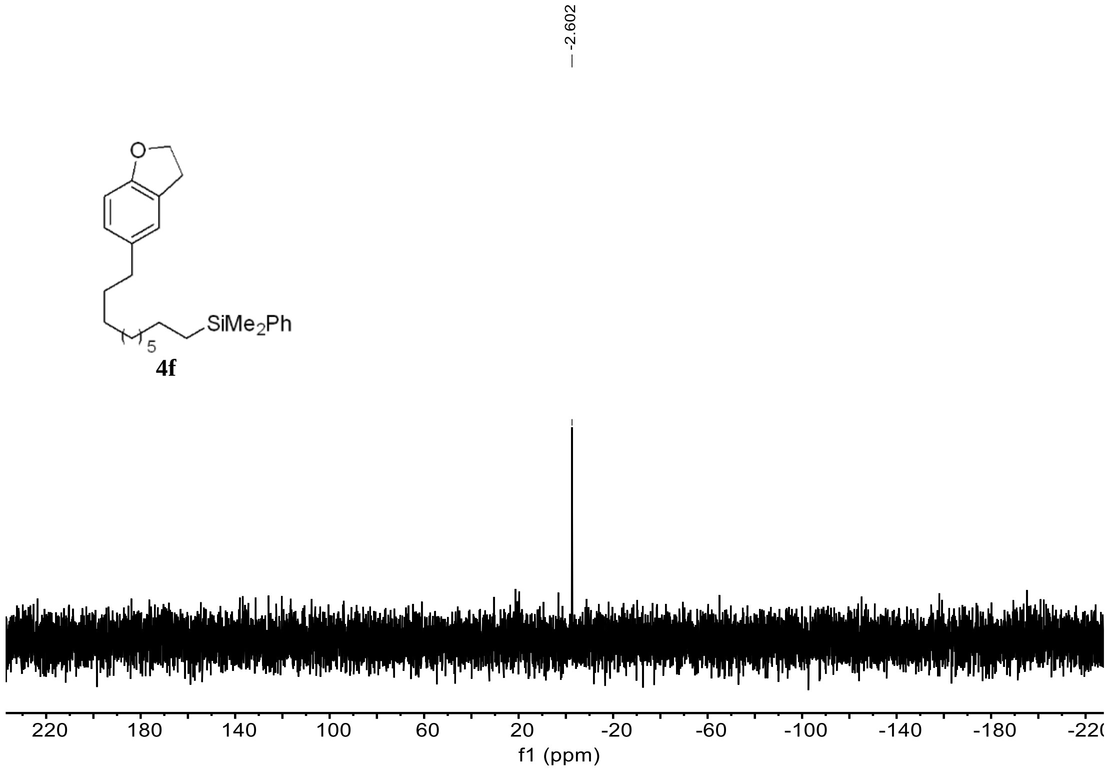

^{29}Si NMR Spectrum of **4f**

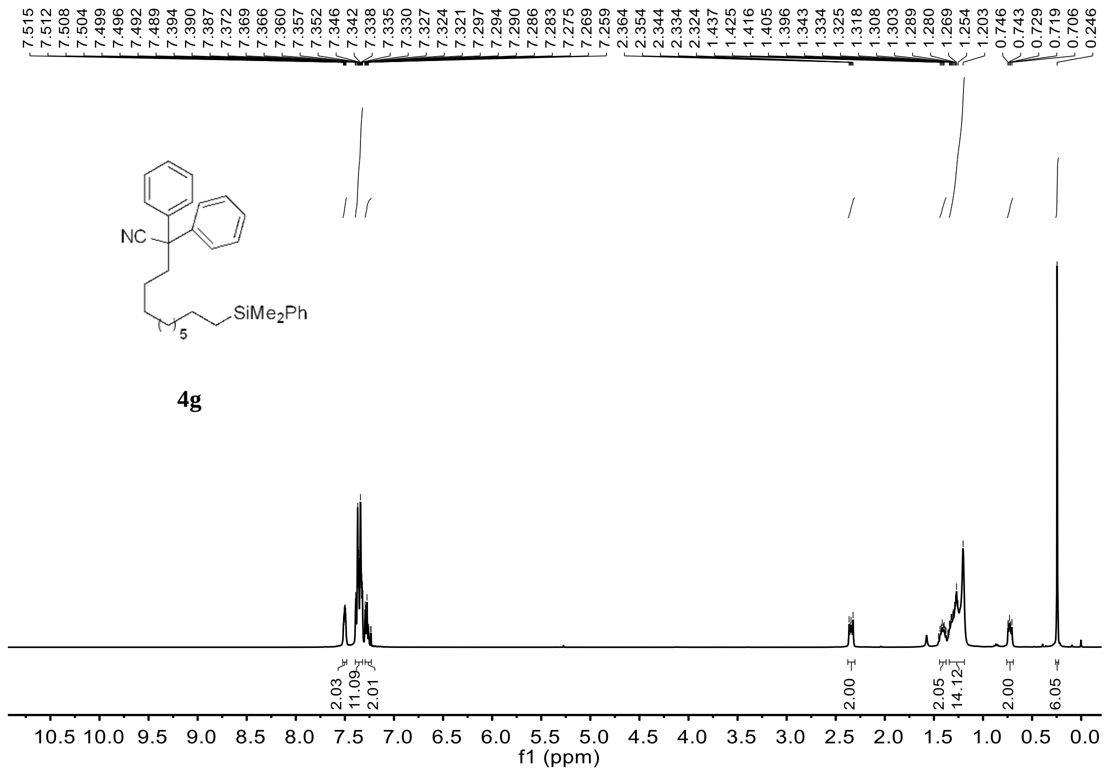

^{1}H NMR Spectrum of **4g**

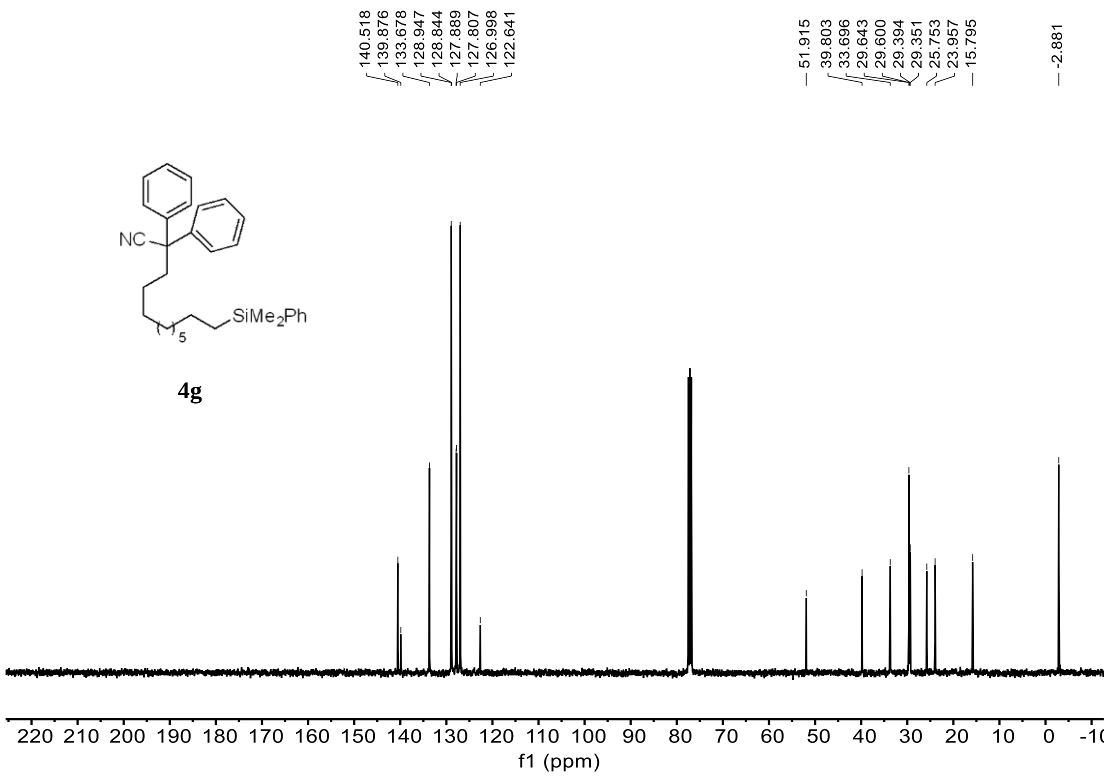

^{13}C NMR Spectrum of **4g**

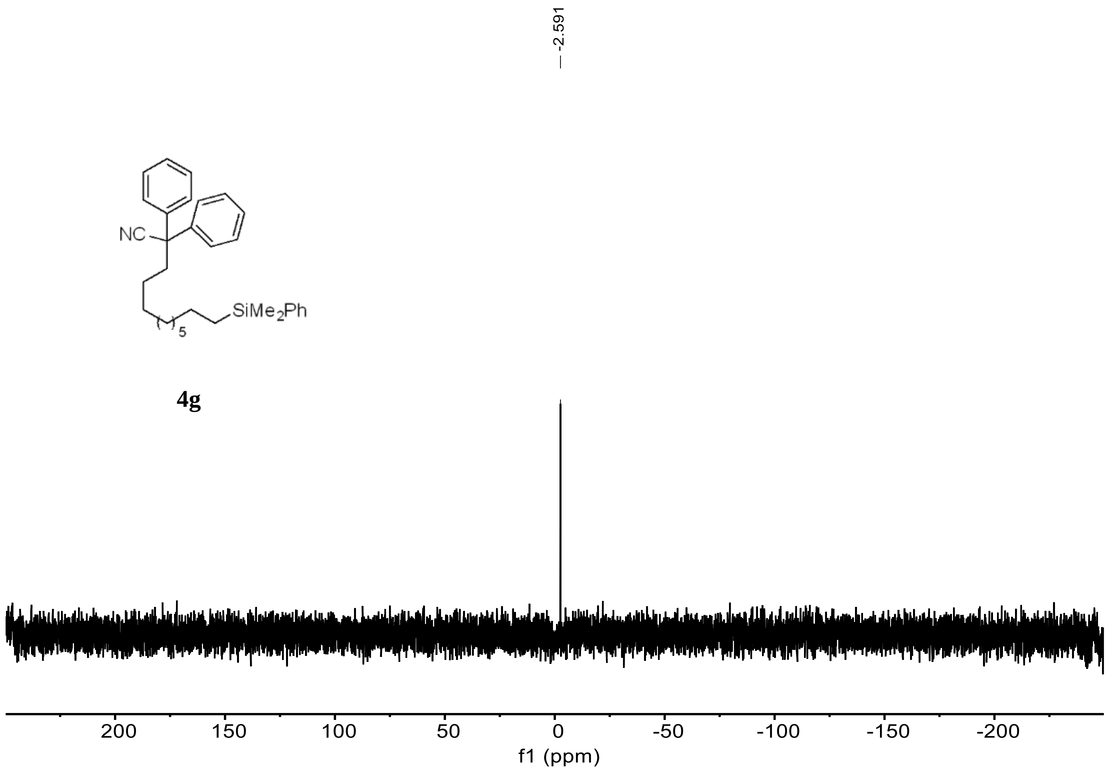

^{29}Si NMR Spectrum of **4g**

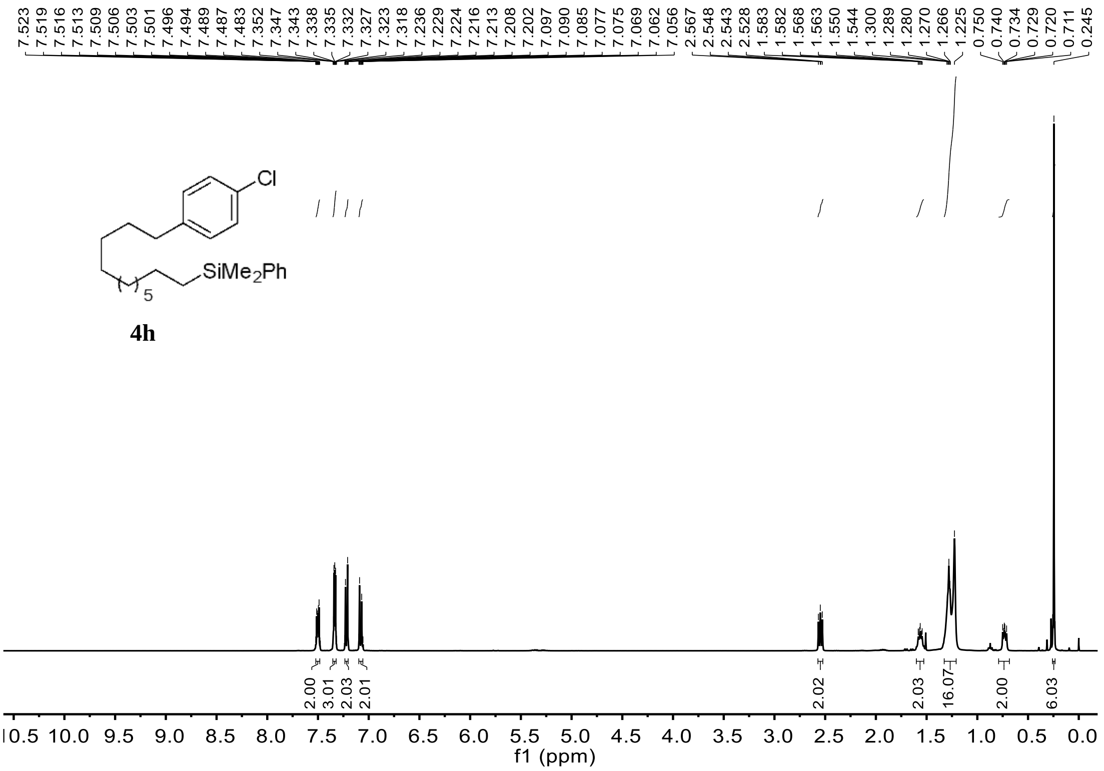

^{1}H NMR Spectrum of **4h**

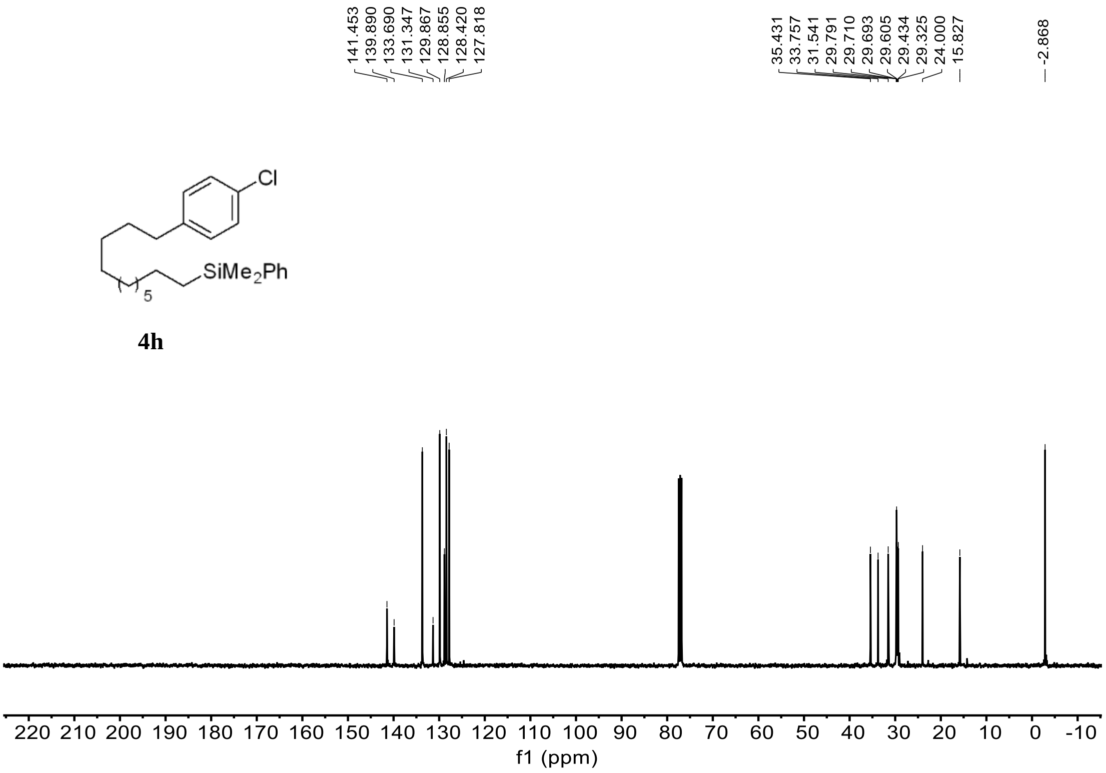

^{13}C NMR Spectrum of **4h**

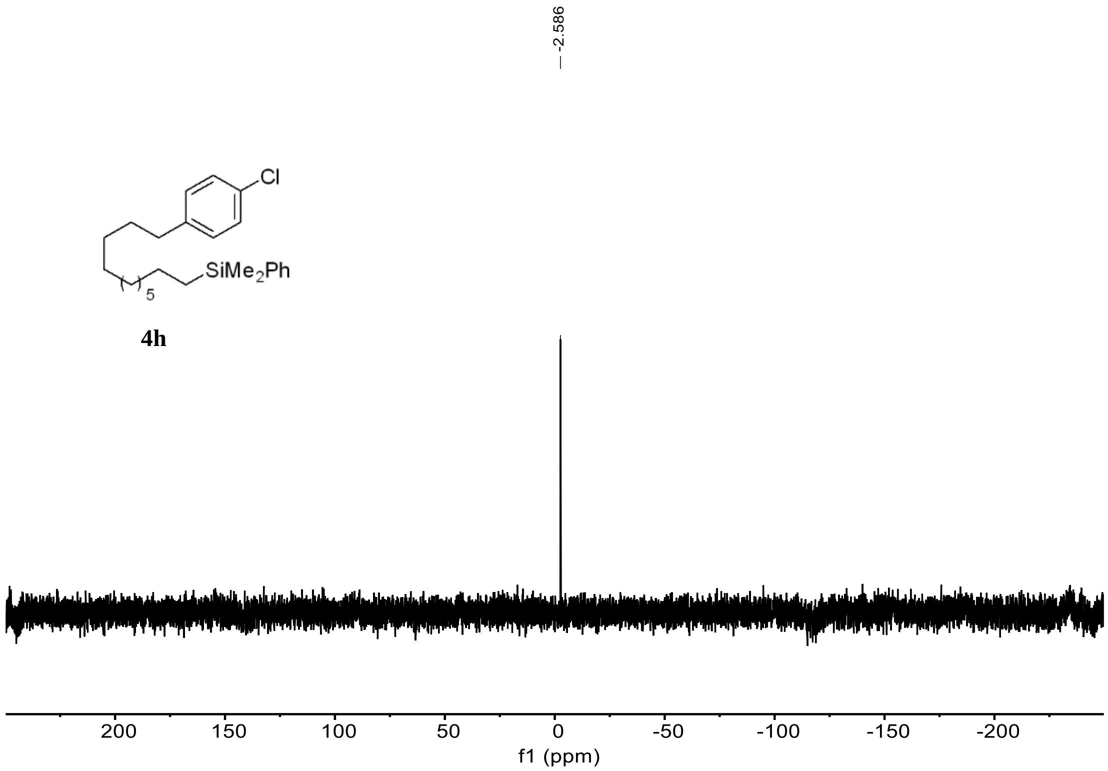

^{29}Si NMR Spectrum of **4h**

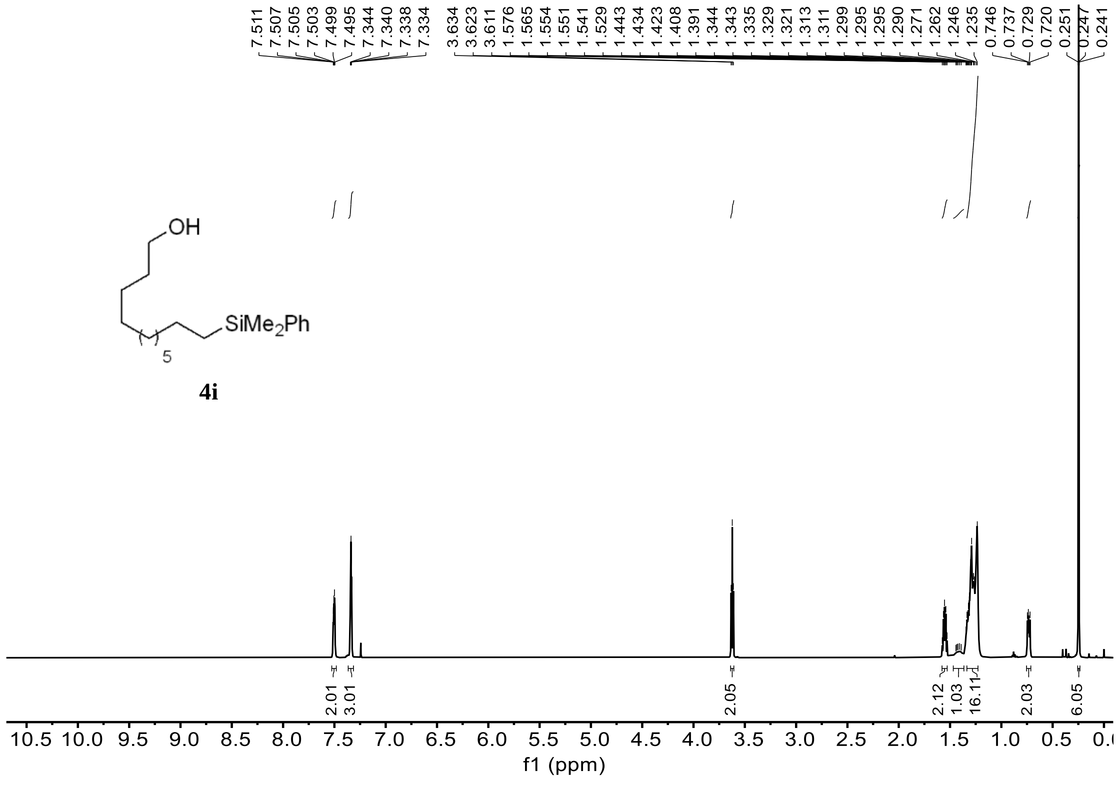

^{1}H NMR Spectrum of **4i**

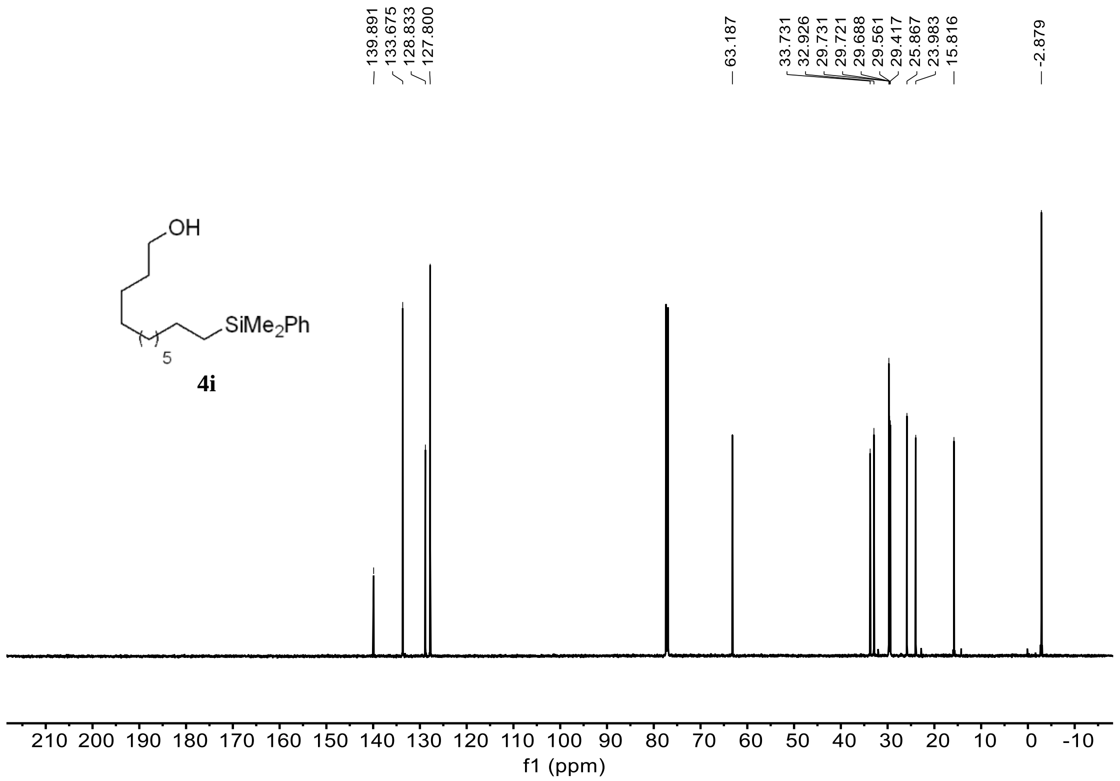

^{13}C NMR Spectrum of **4i**

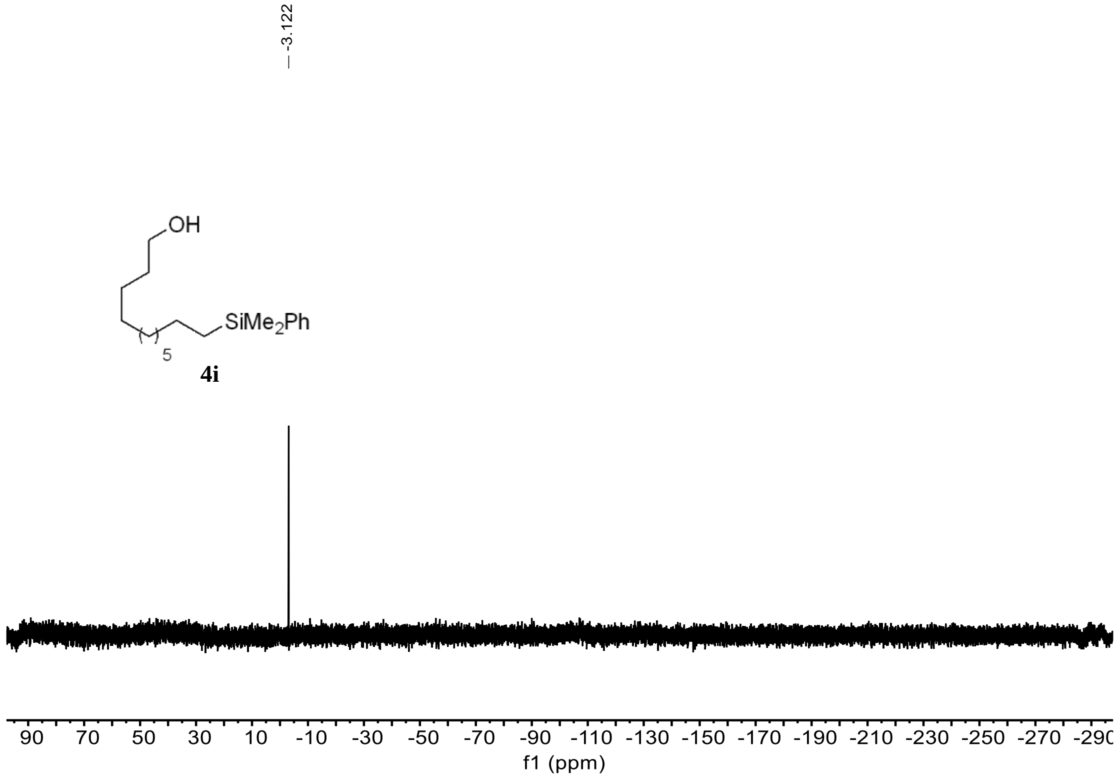

^{29}Si NMR Spectrum of **4i**

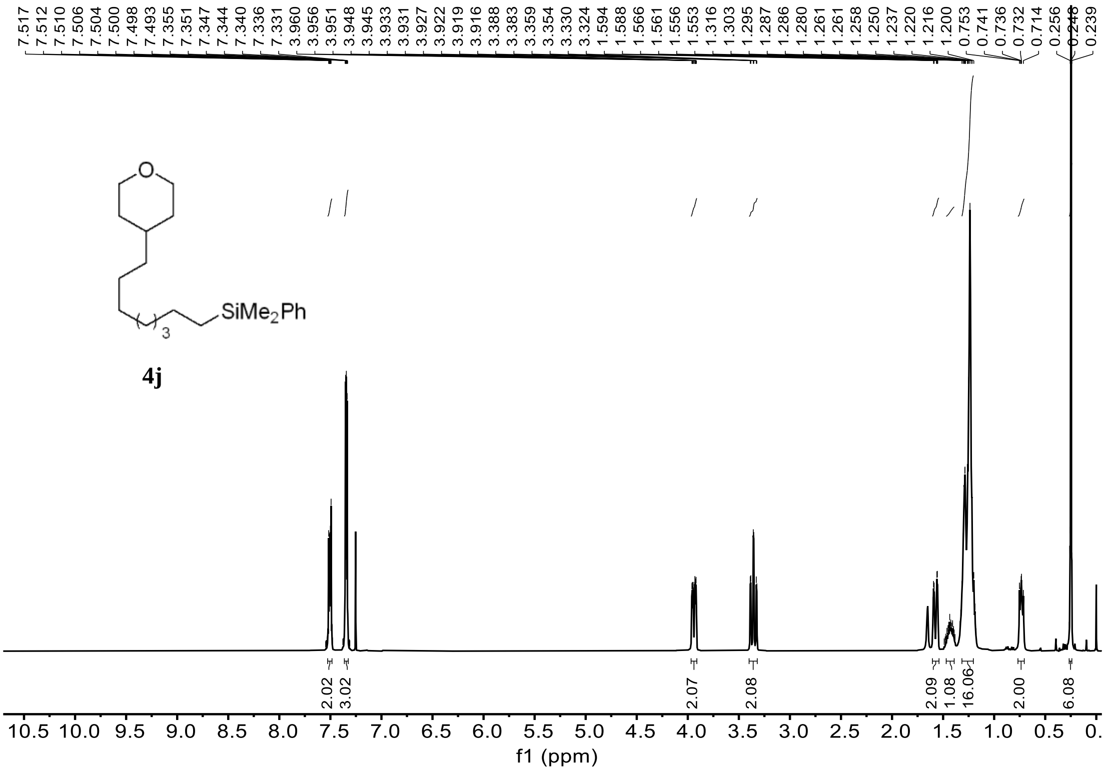

^{1}H NMR Spectrum of **4j**

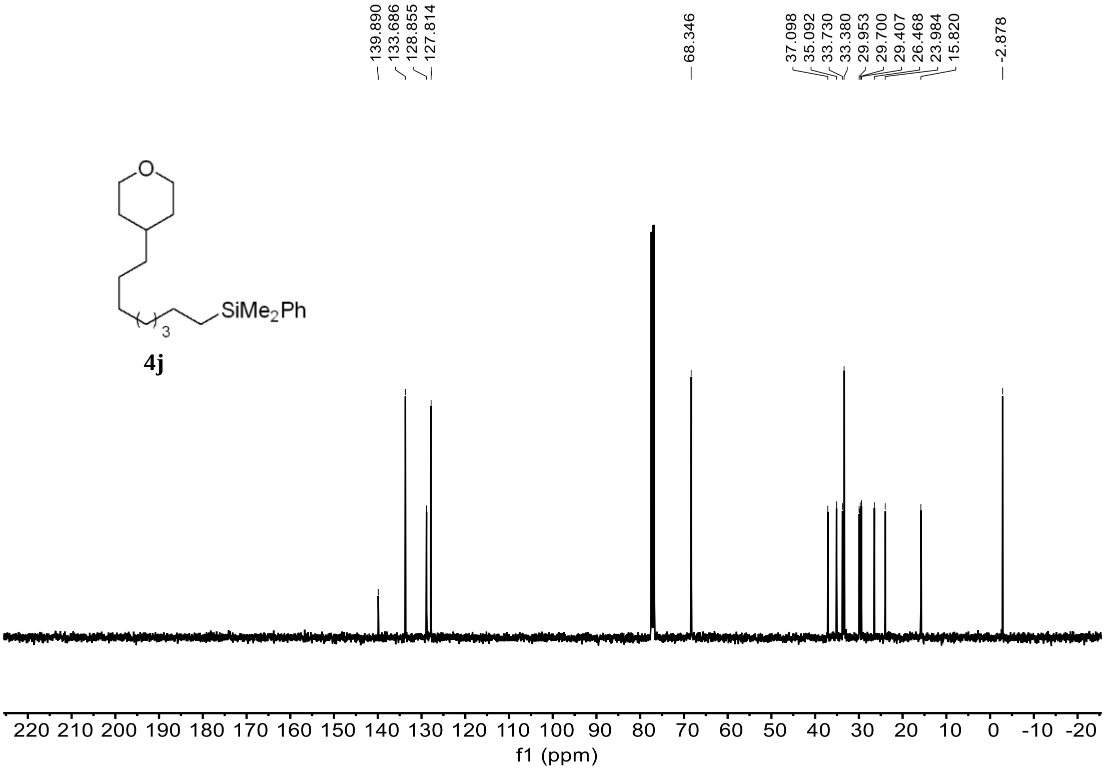

^{13}C NMR Spectrum of **4j**

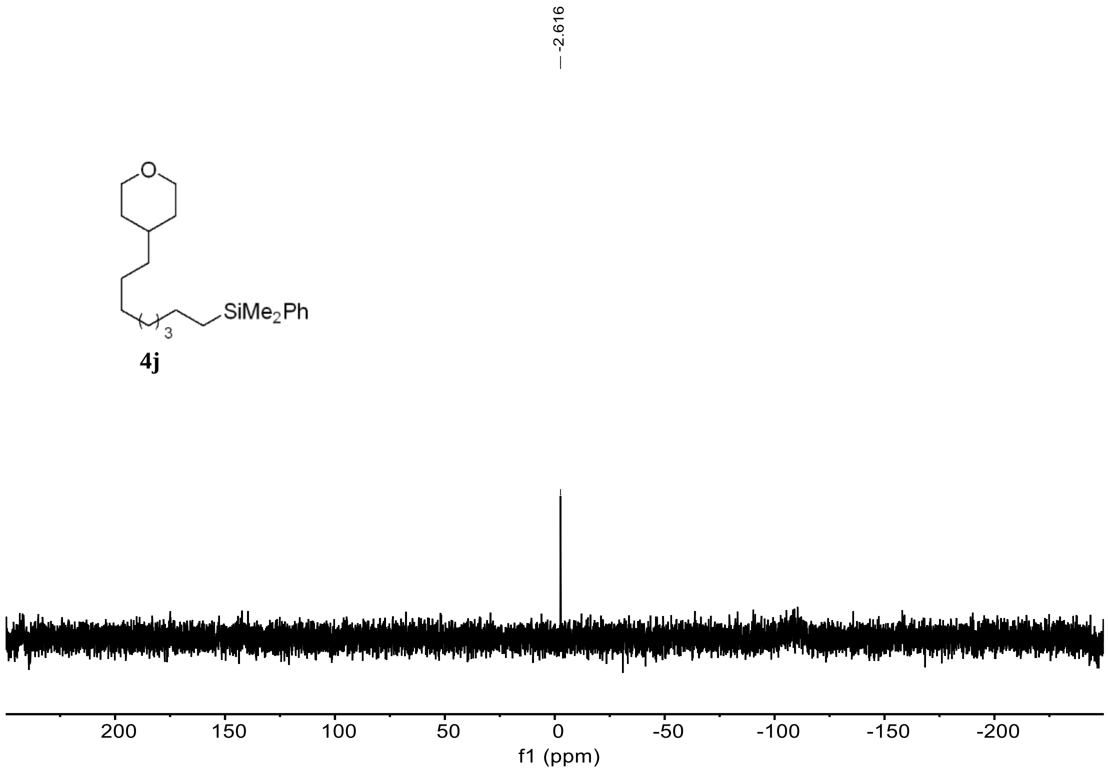

^{29}Si NMR Spectrum of **4j**

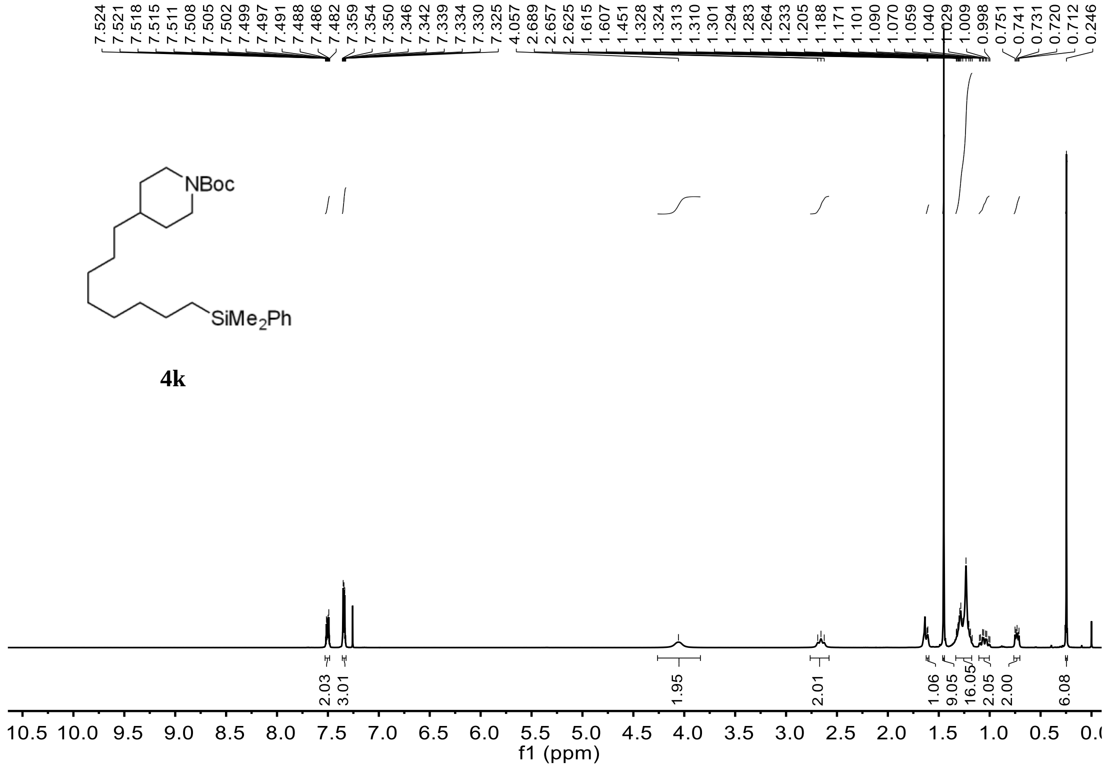

^{1}H NMR Spectrum of **4k**

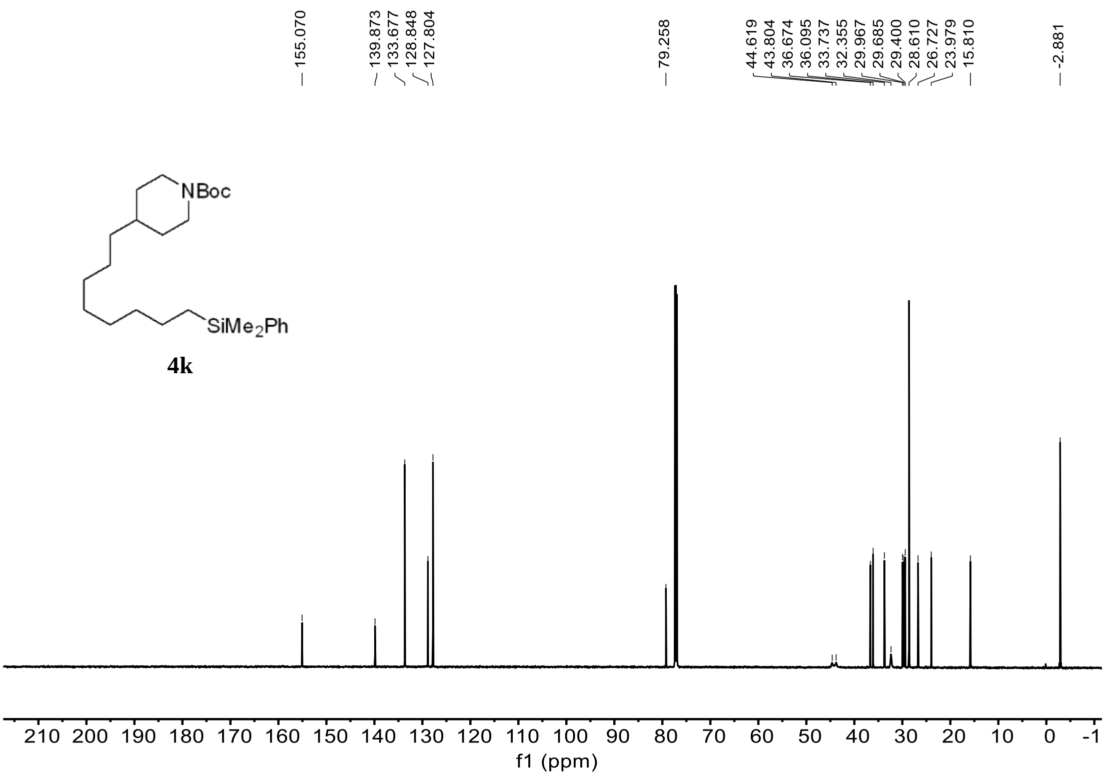

^{13}C NMR Spectrum of **4k**

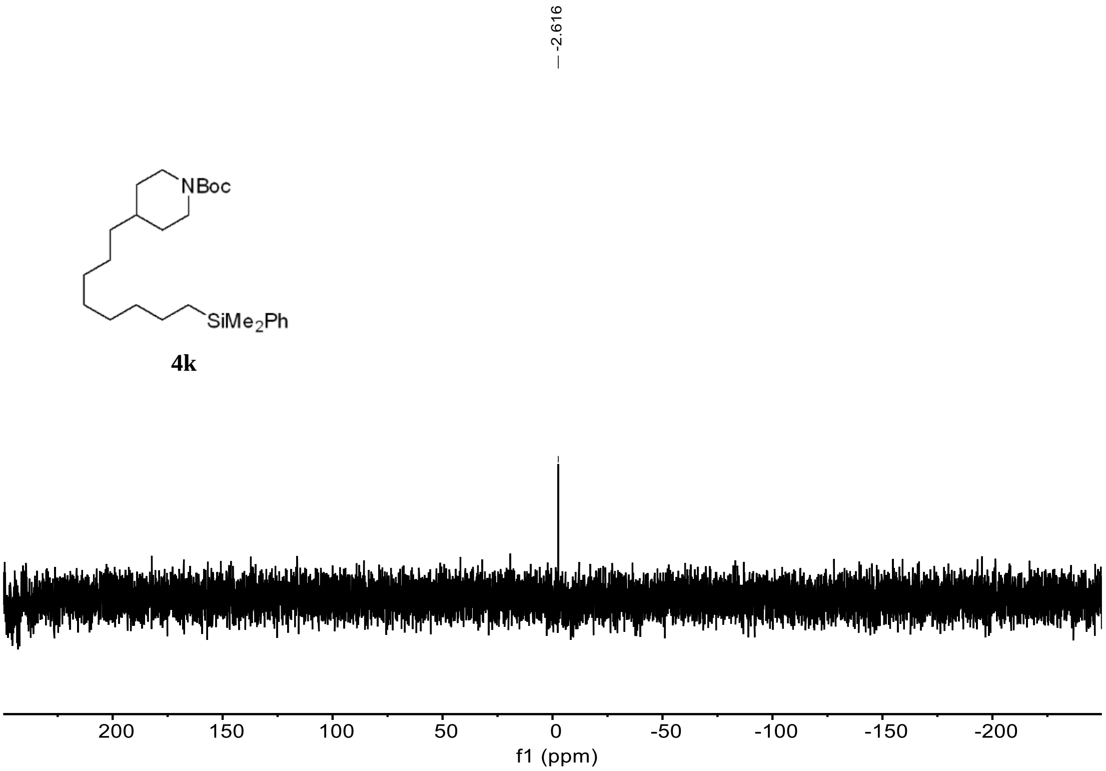

^{29}Si NMR Spectrum of **4k**

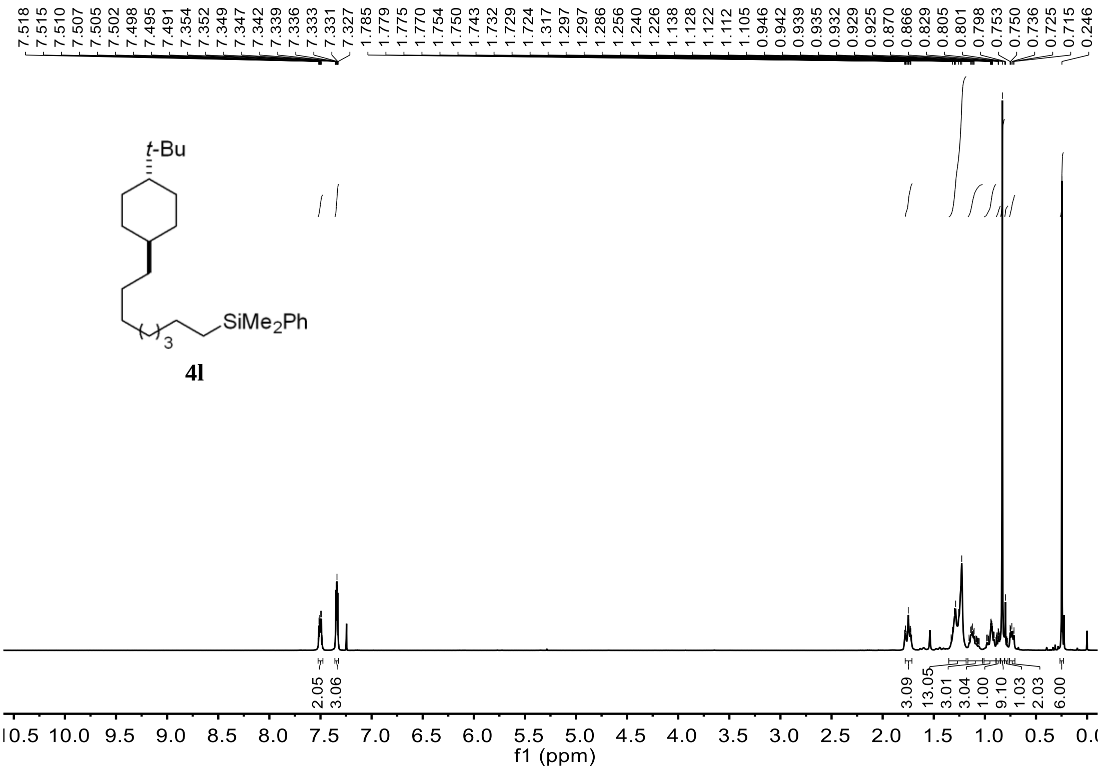

^{1}H NMR Spectrum of **4l**

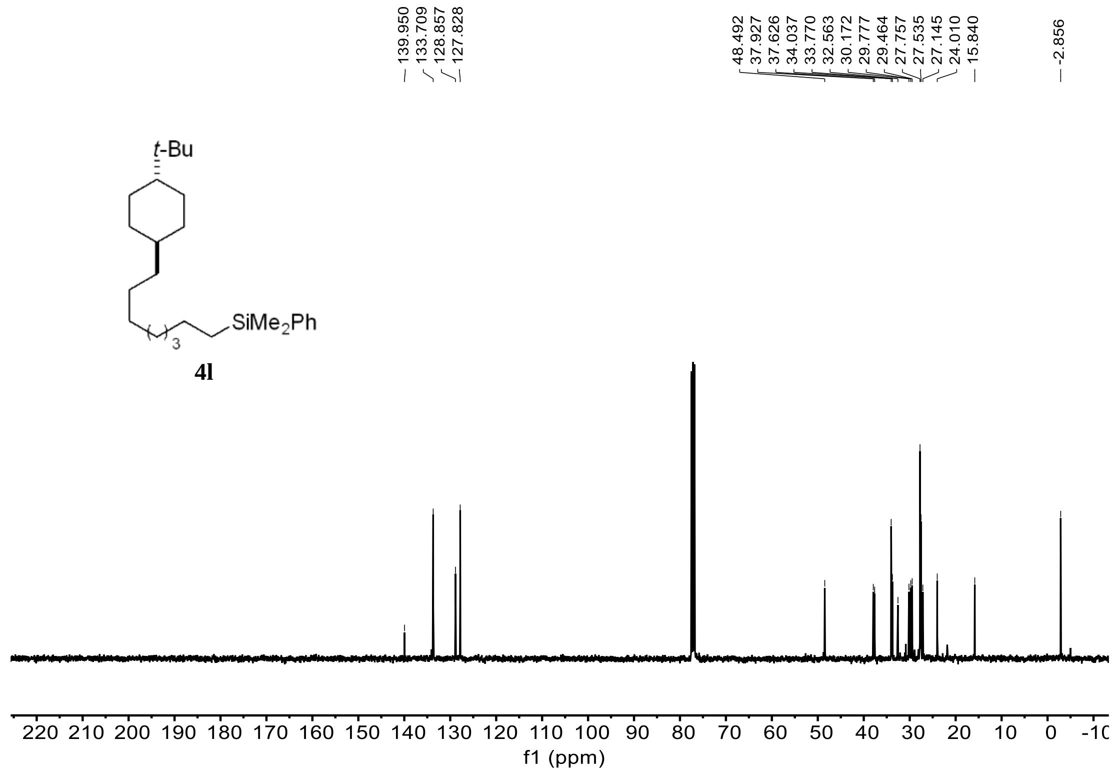

^{13}C NMR Spectrum of **4l**

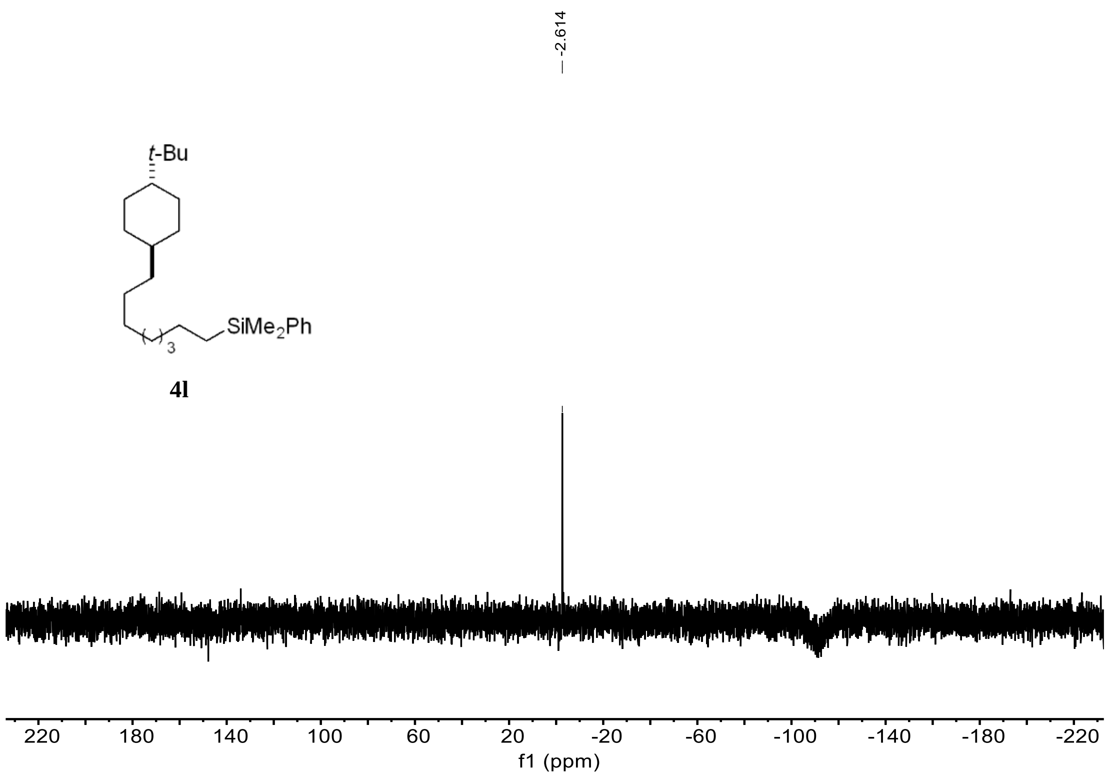

^{29}Si NMR Spectrum of **4l**

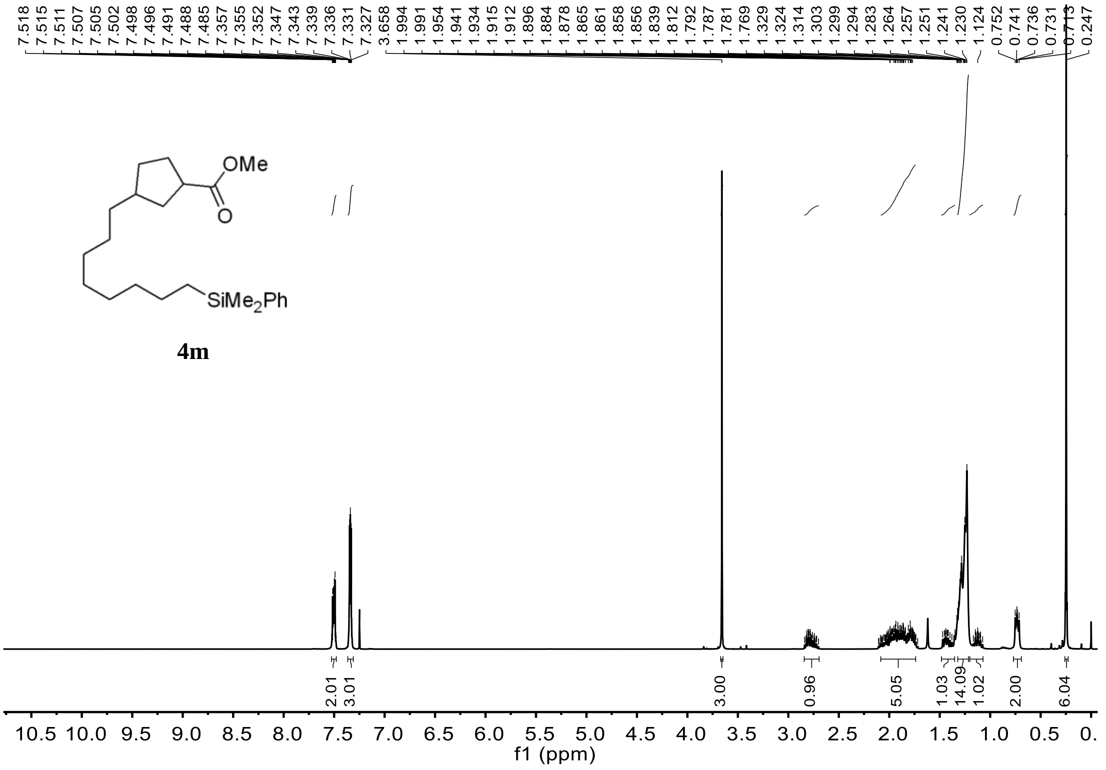

^{1}H NMR Spectrum of **4m**

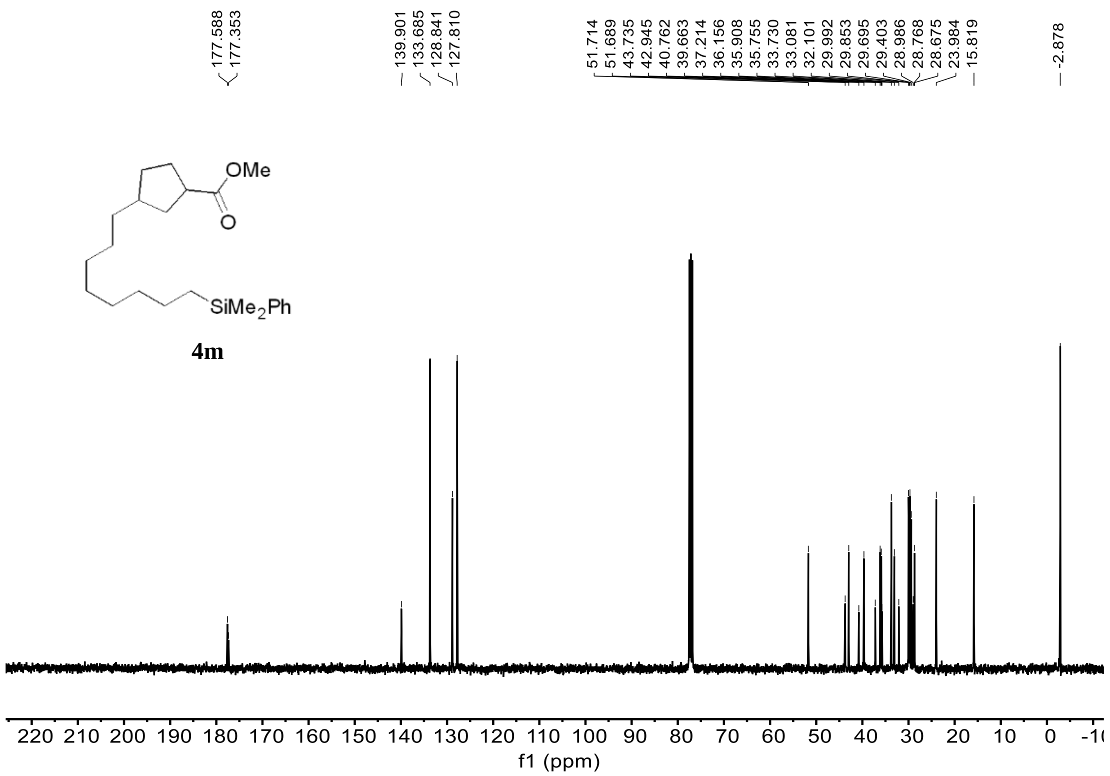

^{13}C NMR Spectrum of **4m**

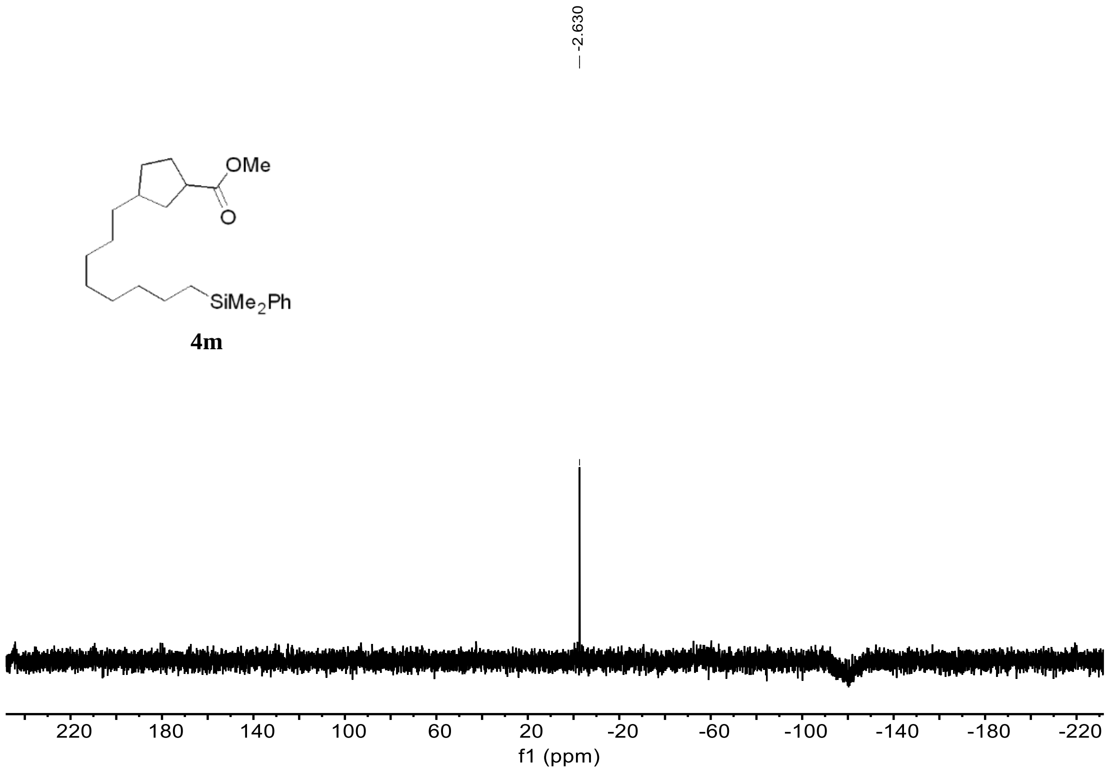

^{29}Si NMR Spectrum of **4m**

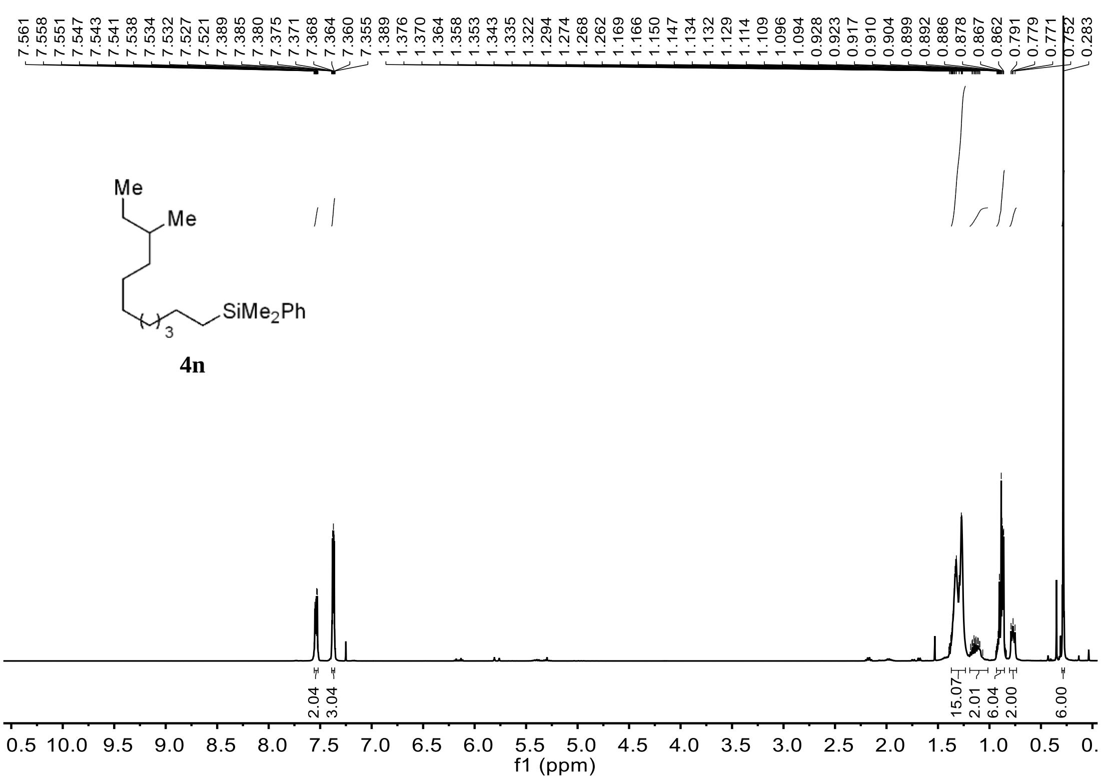

^{1}H NMR Spectrum of **4n**

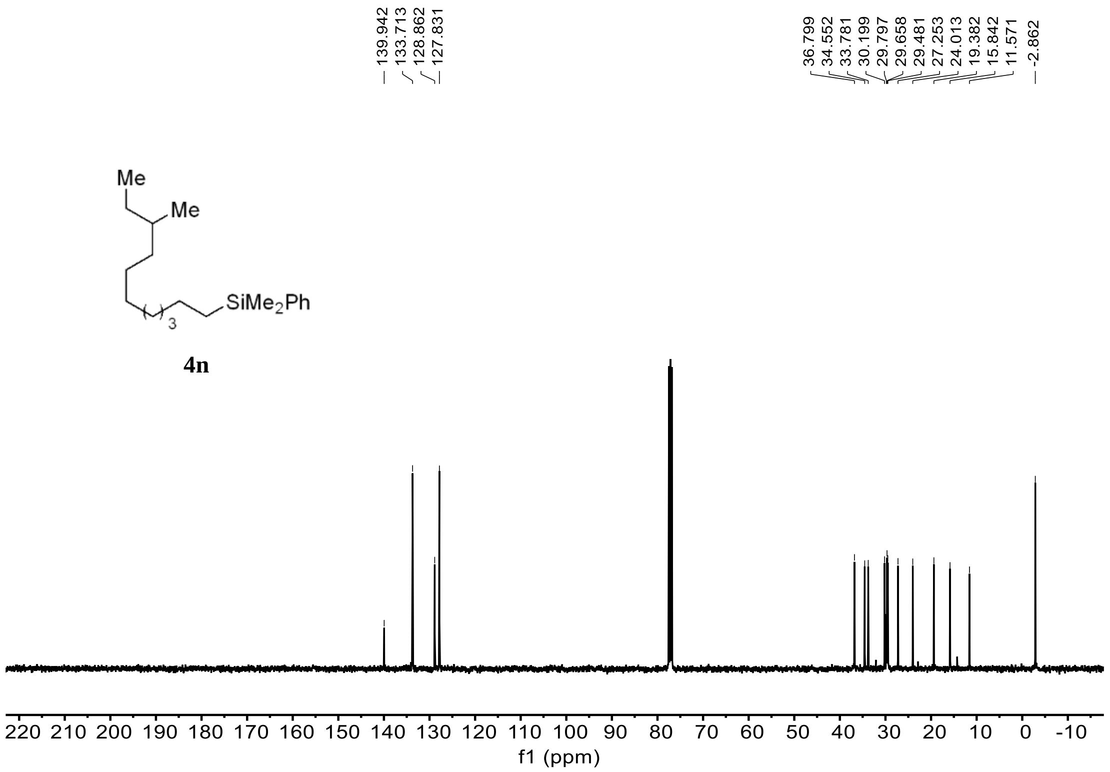

^{13}C NMR Spectrum of **4n**

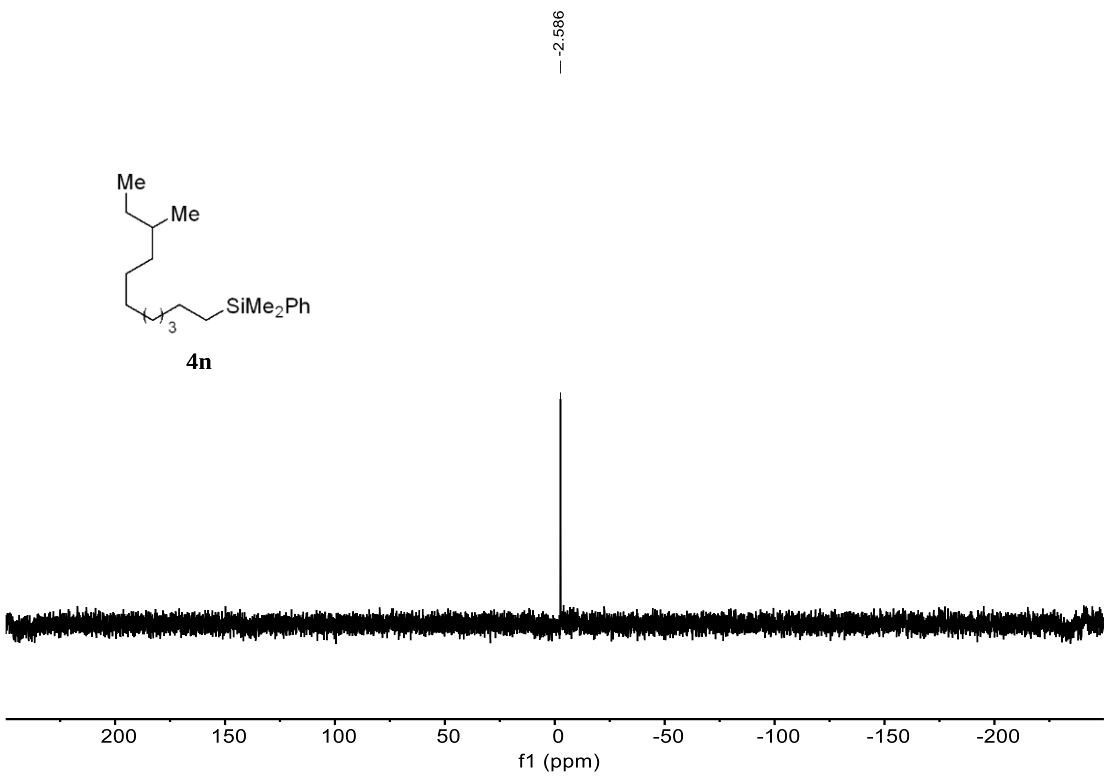

^{29}Si NMR Spectrum of **4n**

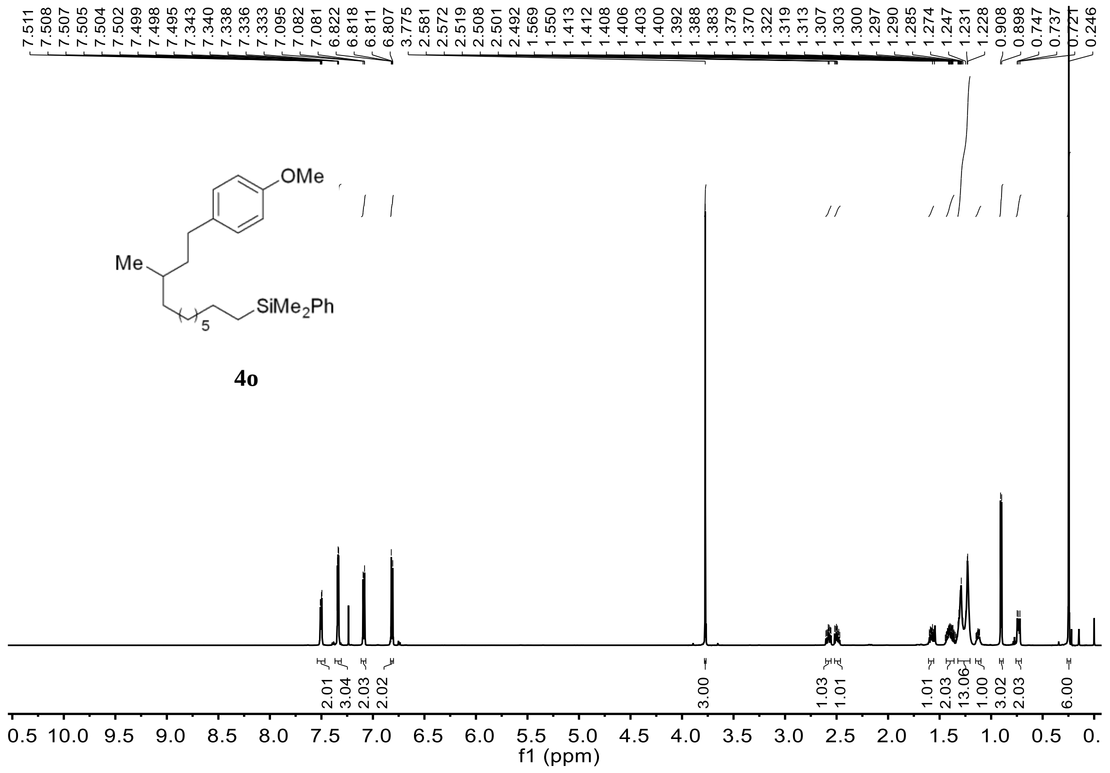

^{1}H NMR Spectrum of **4o**

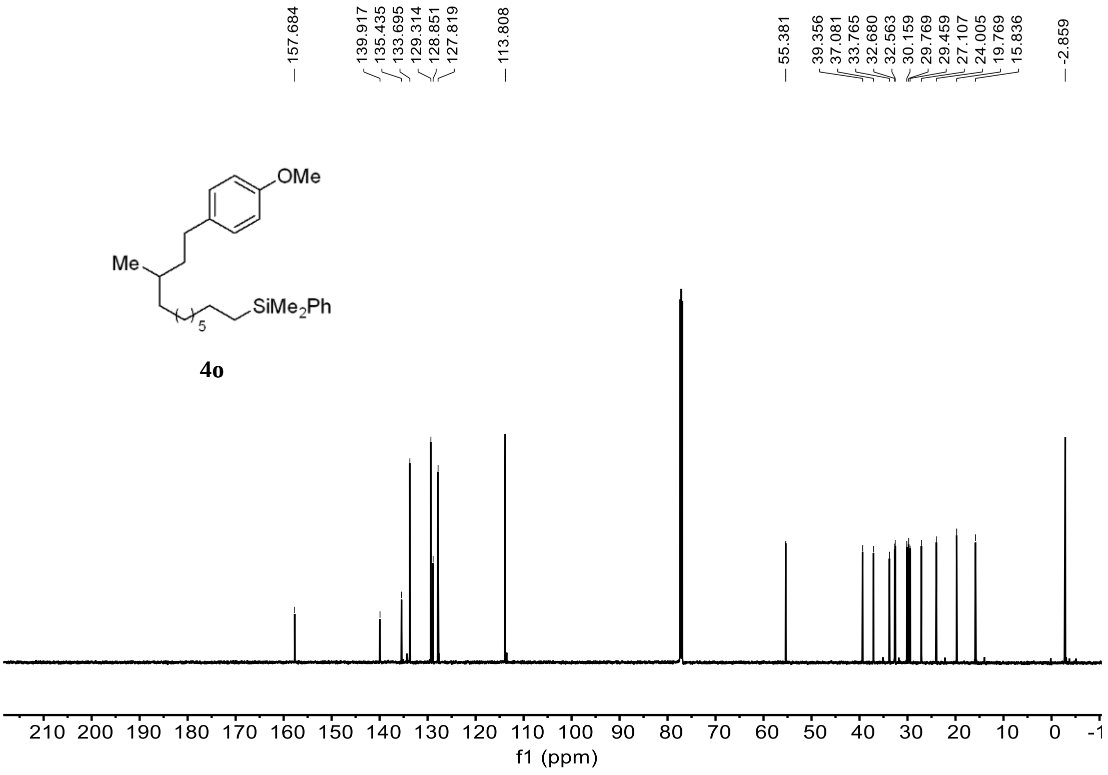

^{13}C NMR Spectrum of **4o**

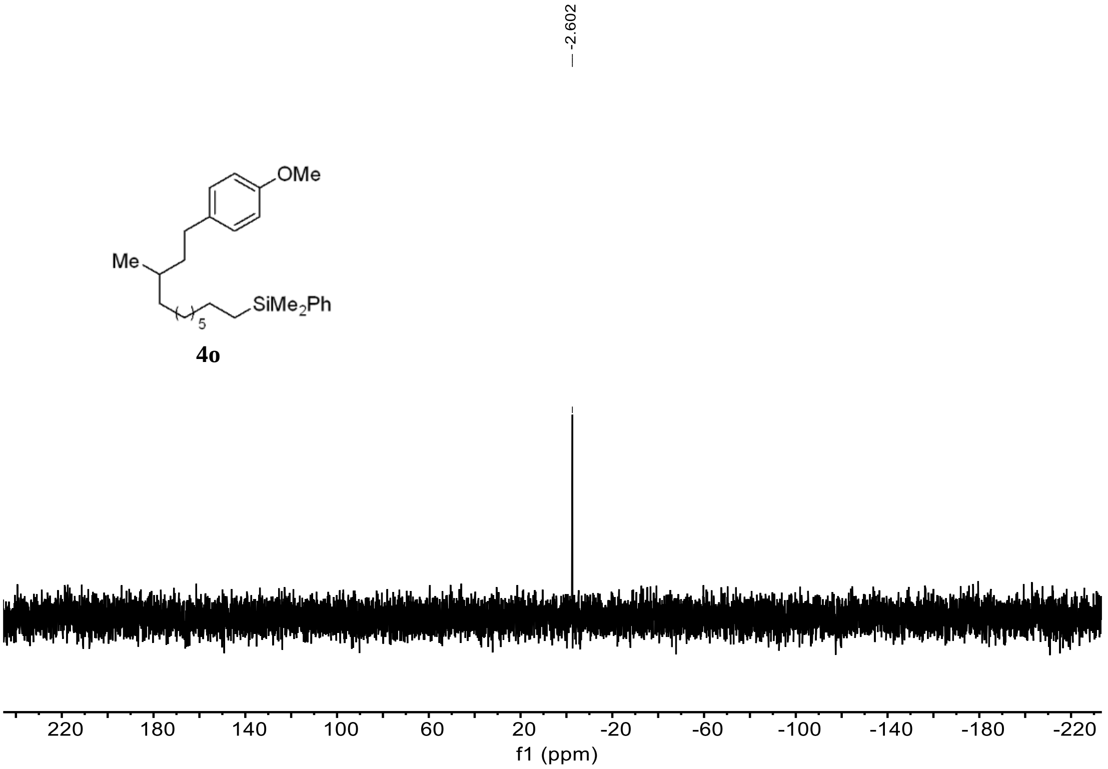

^{29}Si NMR Spectrum of **4o**

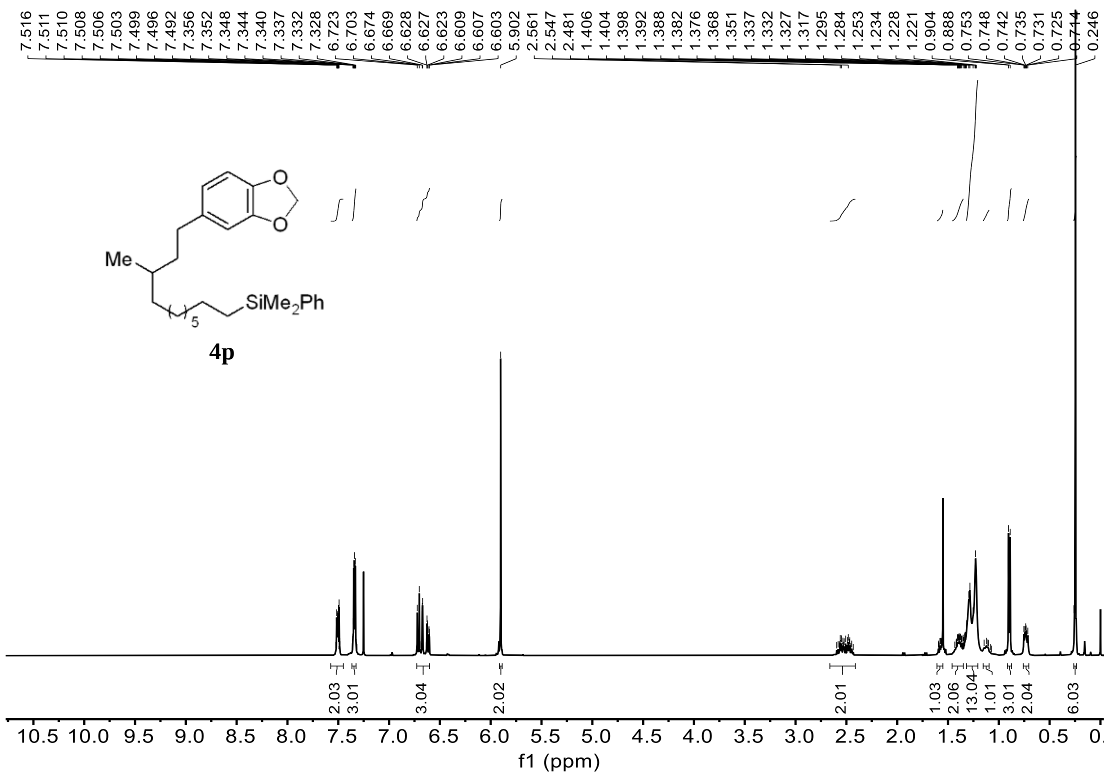

^{1}H NMR Spectrum of 4**p**

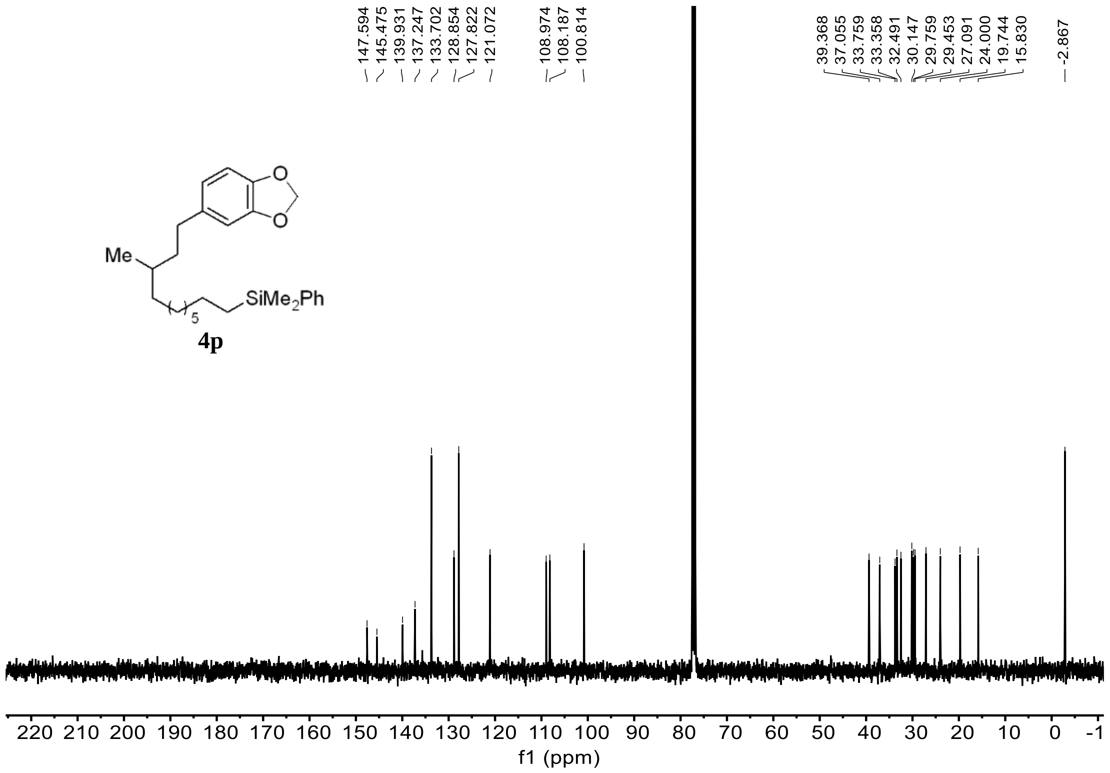

^{13}C NMR Spectrum of **4p**

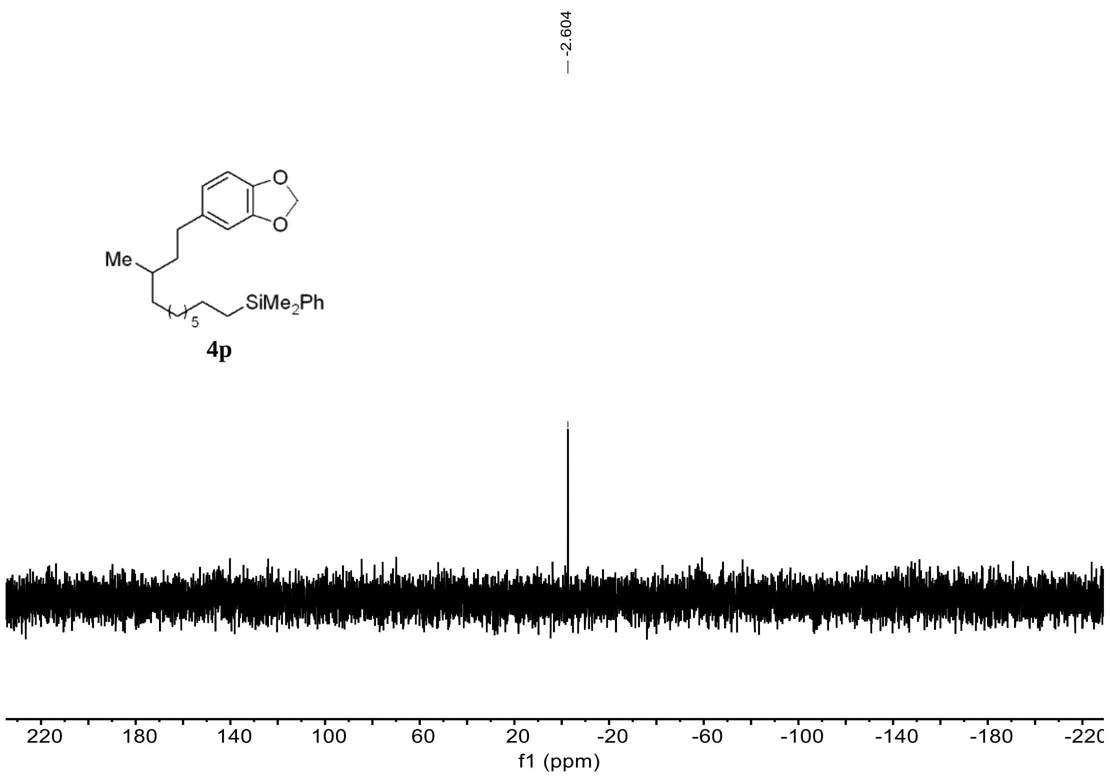

^{29}Si NMR Spectrum of **4p**

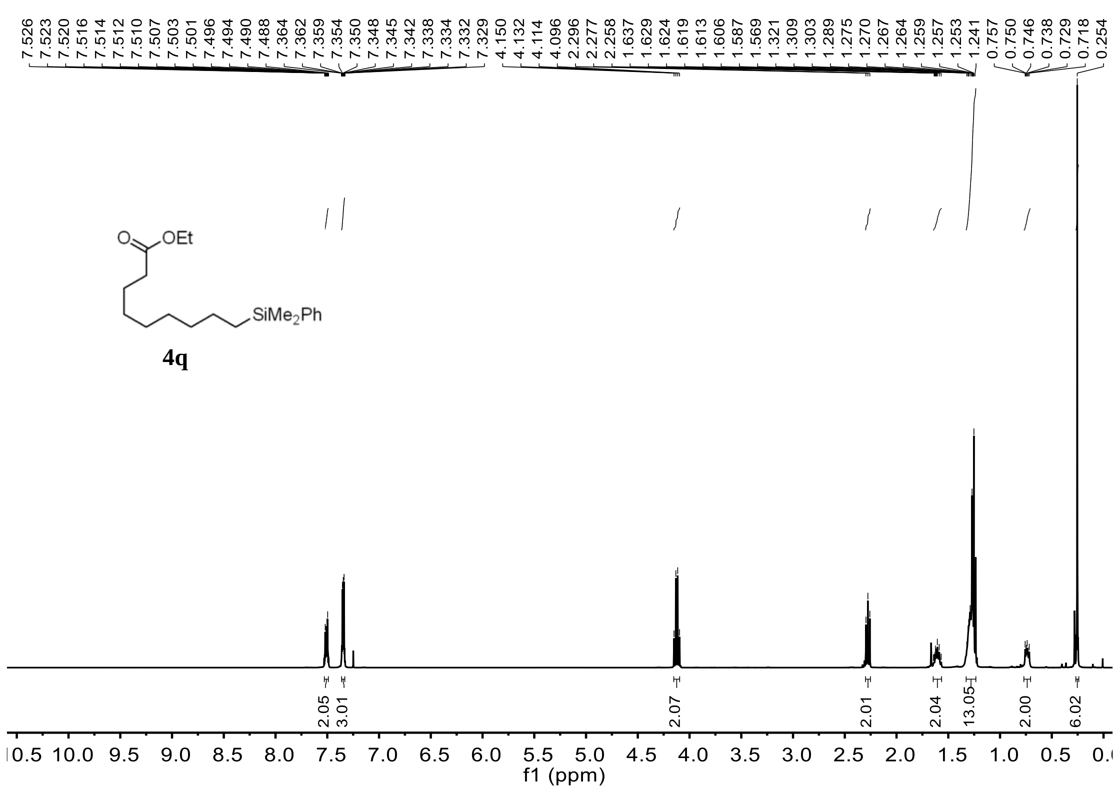

^{1}H NMR Spectrum of **4q**

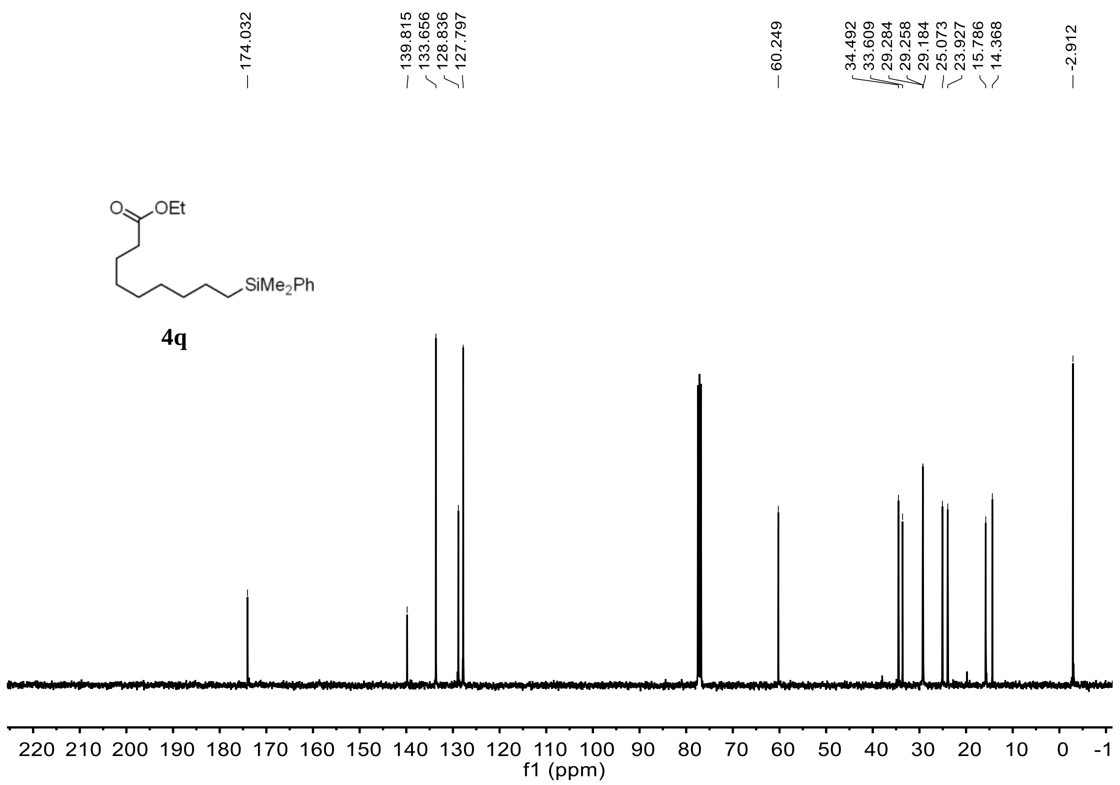

^{13}C NMR Spectrum of **4q**

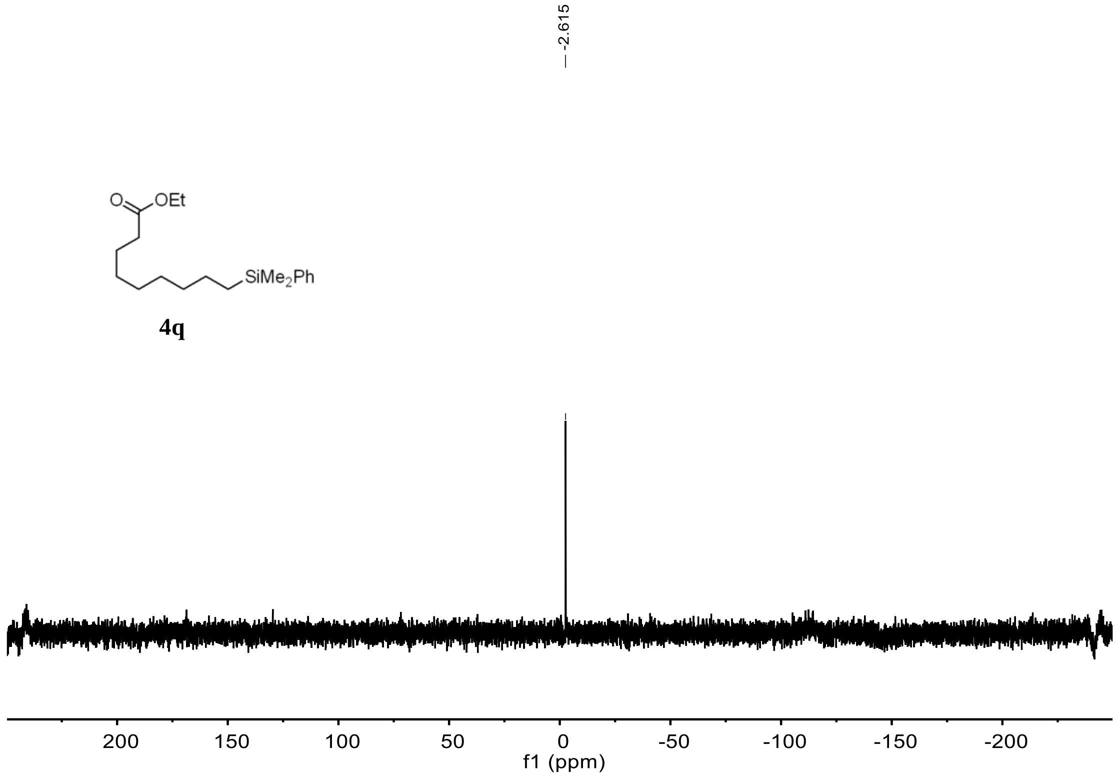

^{29}Si NMR Spectrum of **4q**

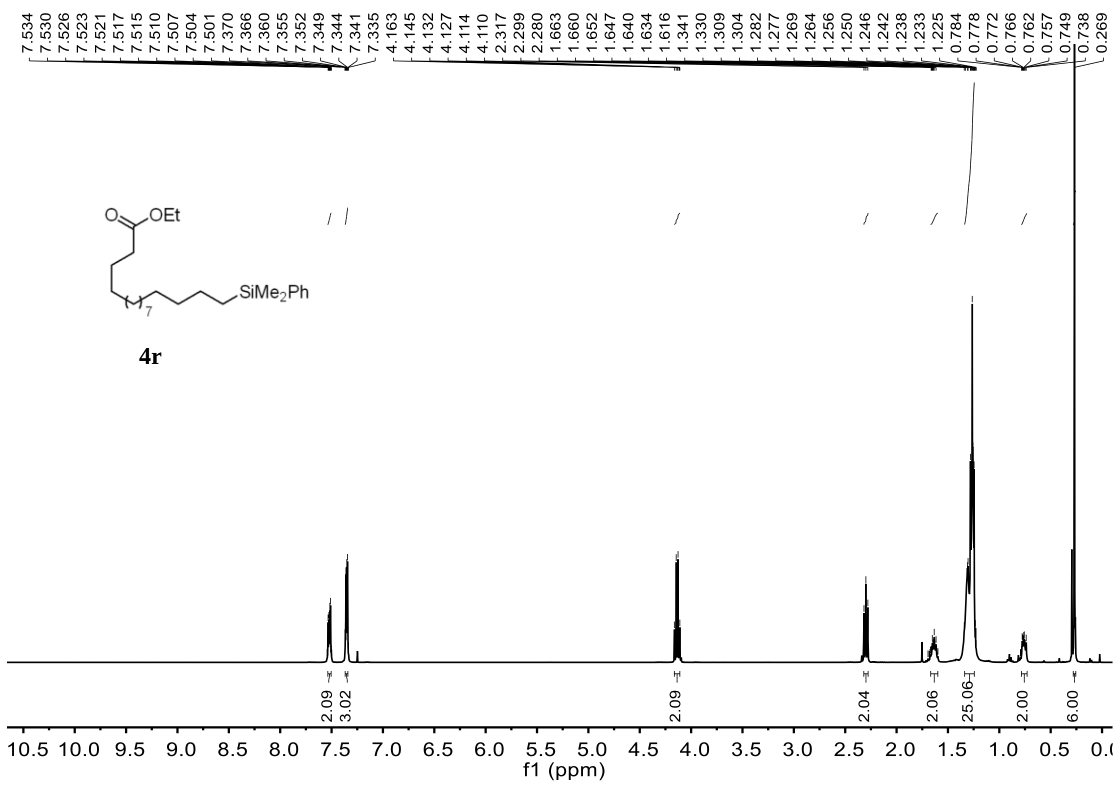

^{1}H NMR Spectrum of **4r**

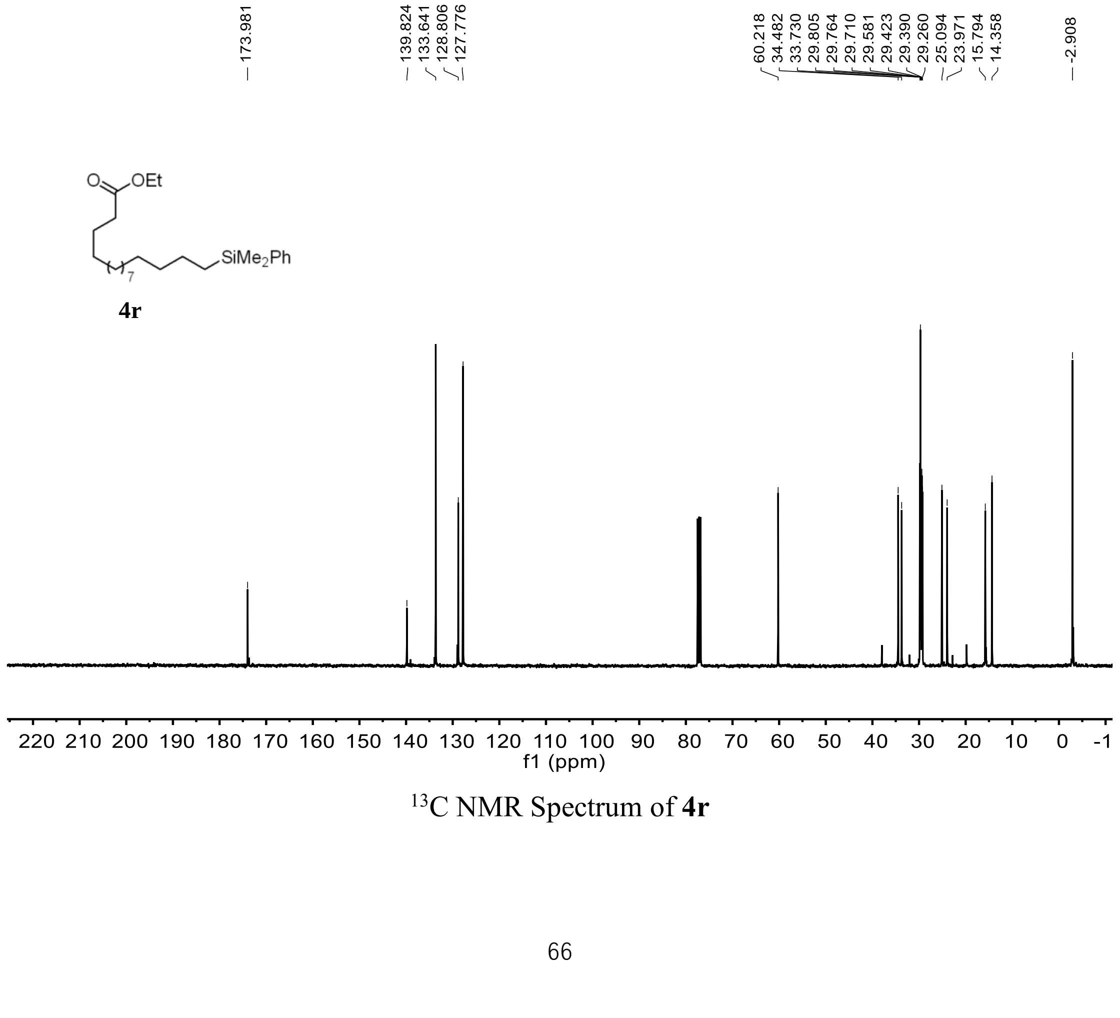

^{13}C NMR Spectrum of **4r**

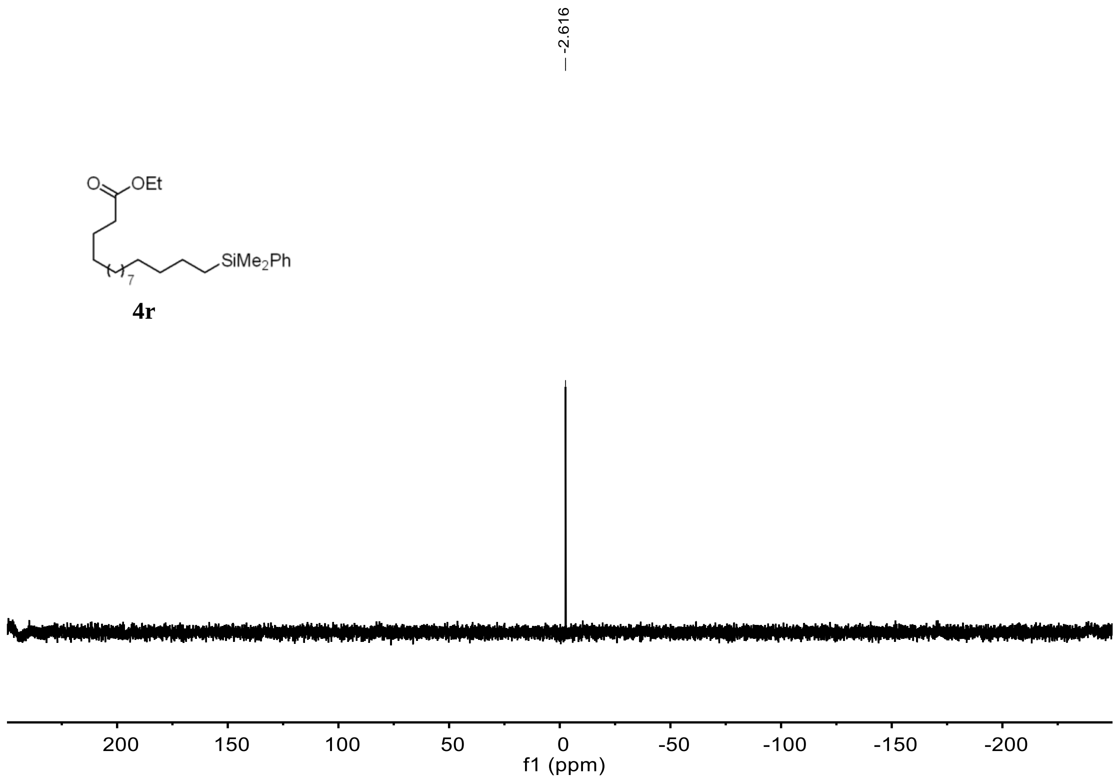

^{29}Si NMR Spectrum of **4r**

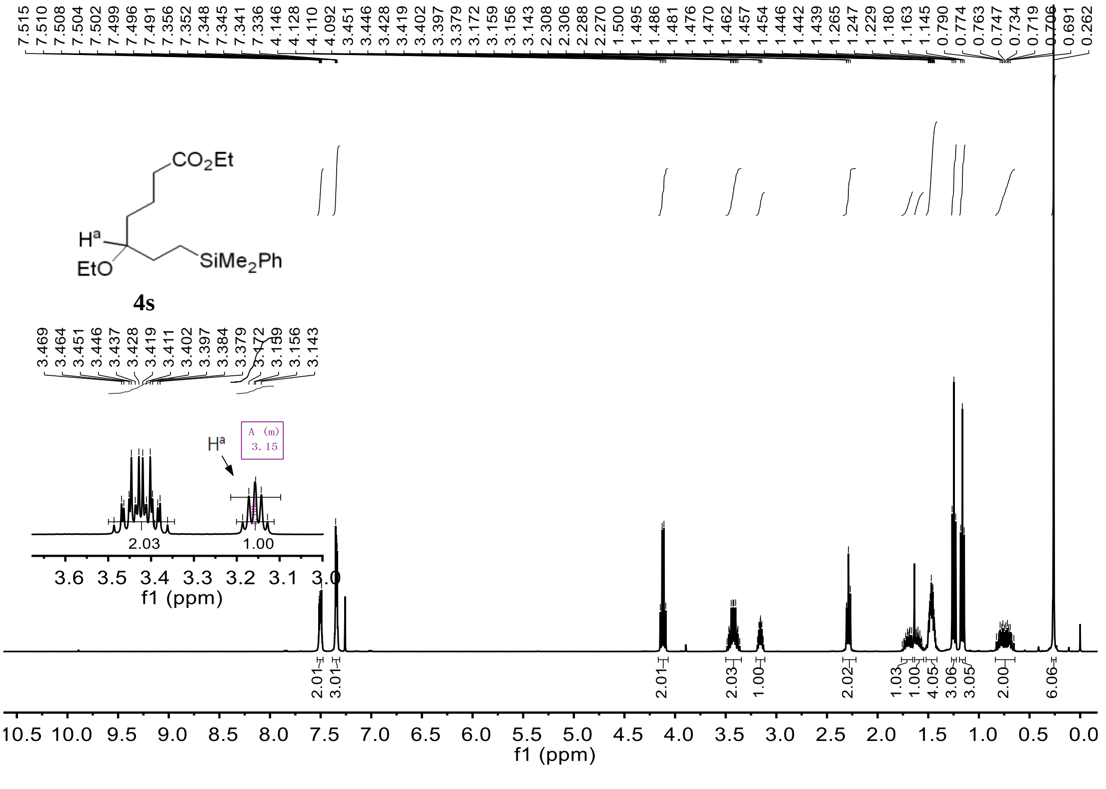

^{1}H NMR Spectrum of **4s**

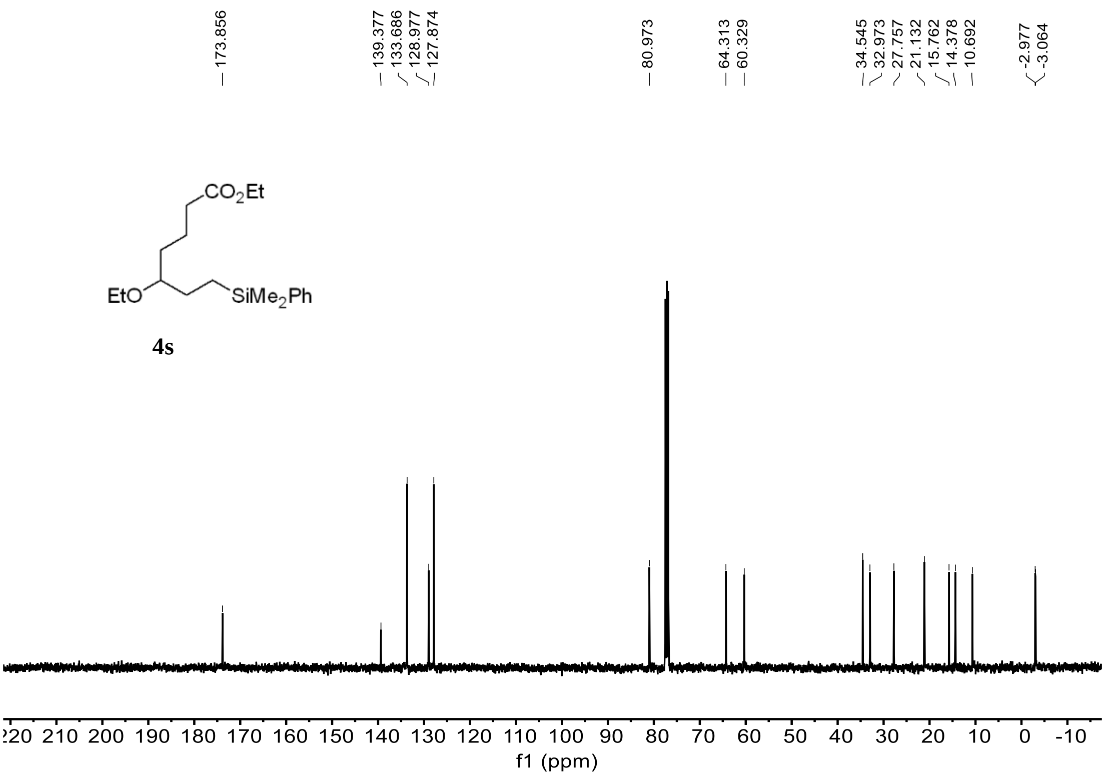

^{13}C NMR Spectrum of **4s**

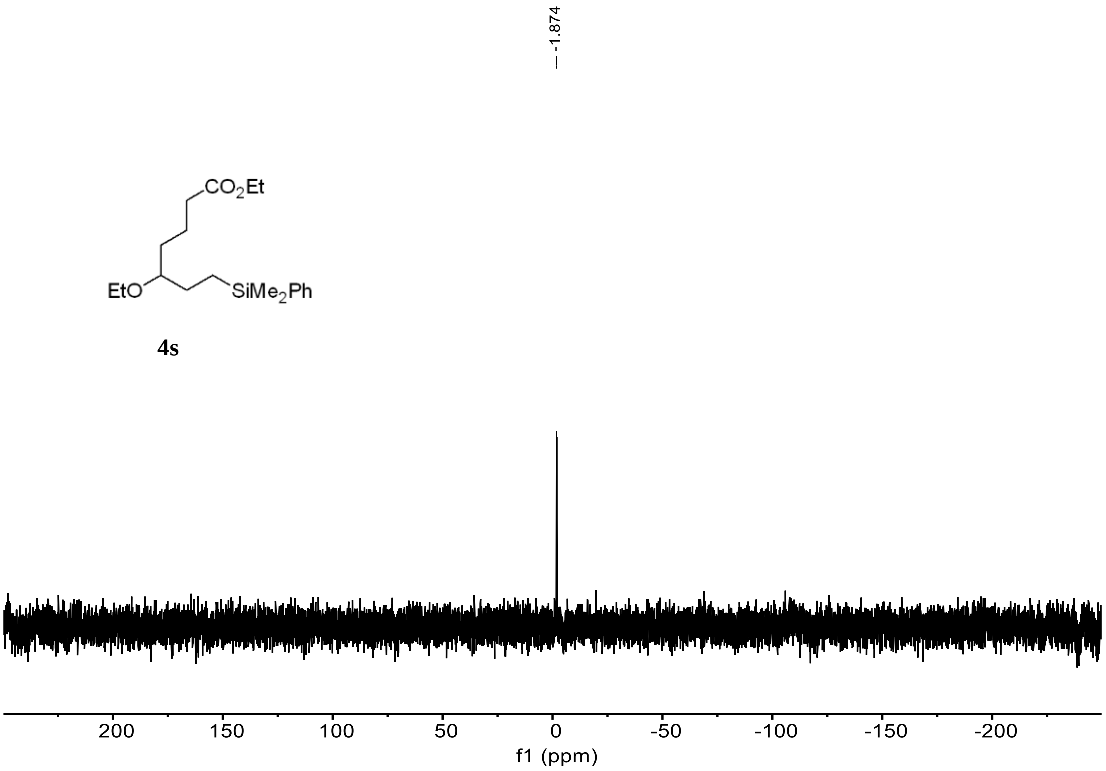

^{29}Si NMR Spectrum of **4s**

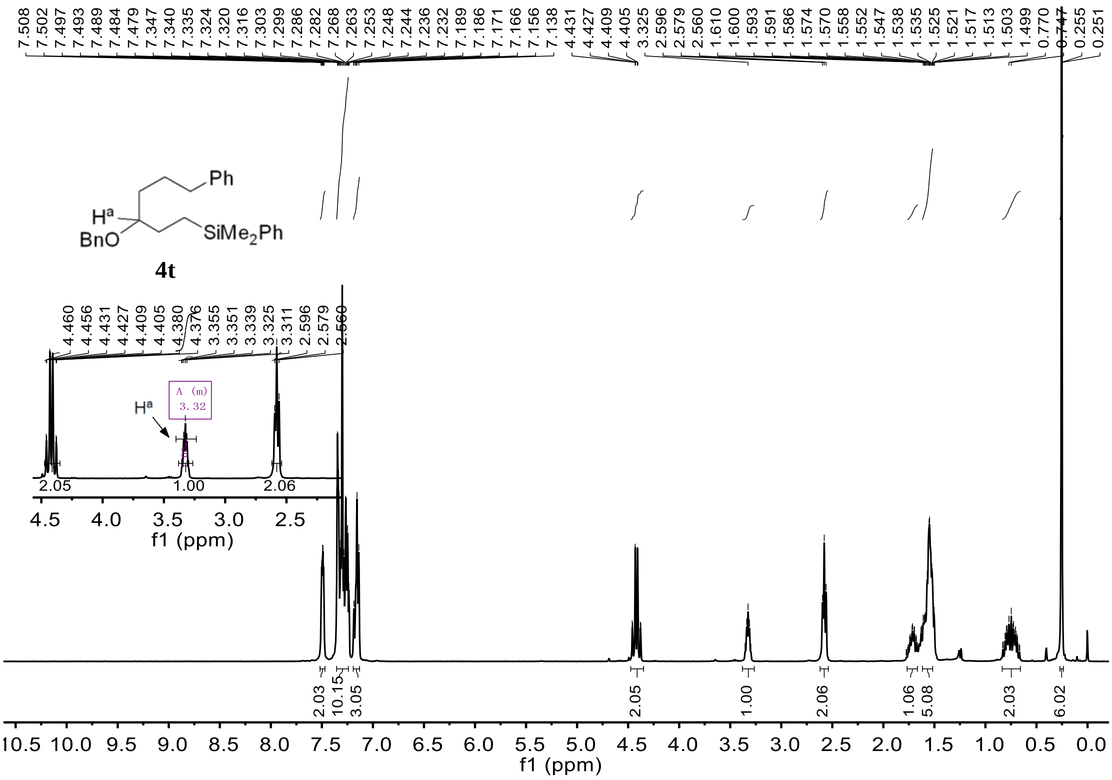

^{1}H NMR Spectrum of **4t**

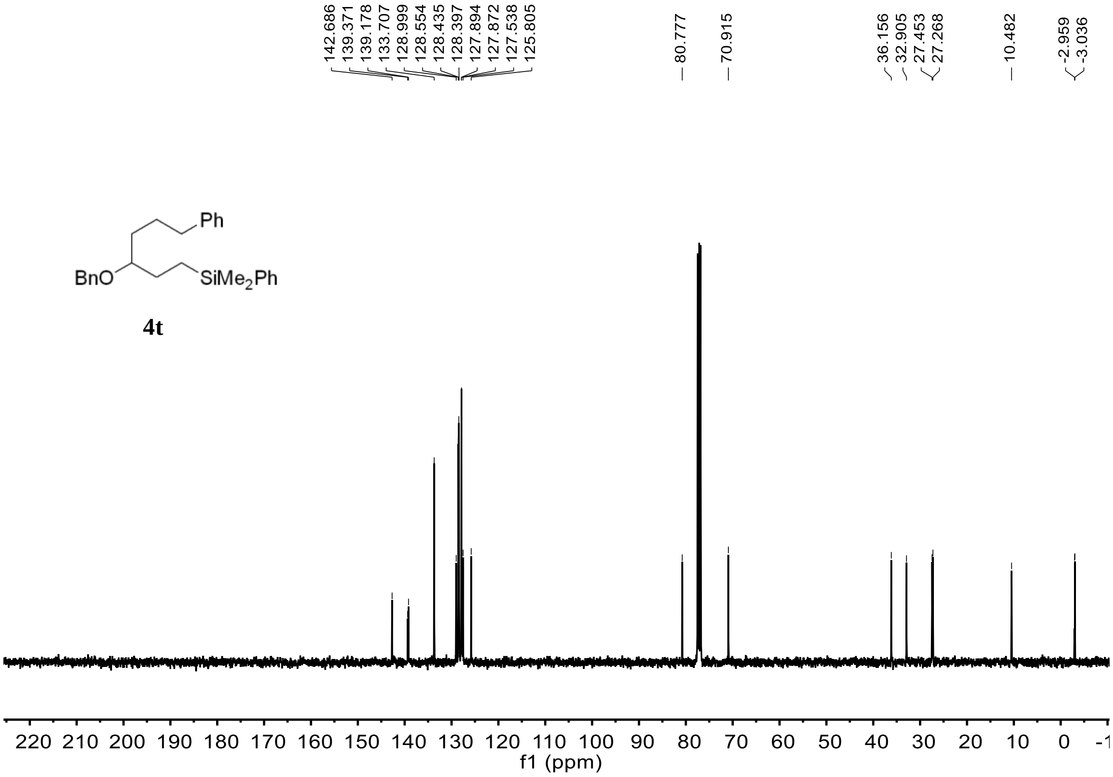

^{13}C NMR Spectrum of **4t**

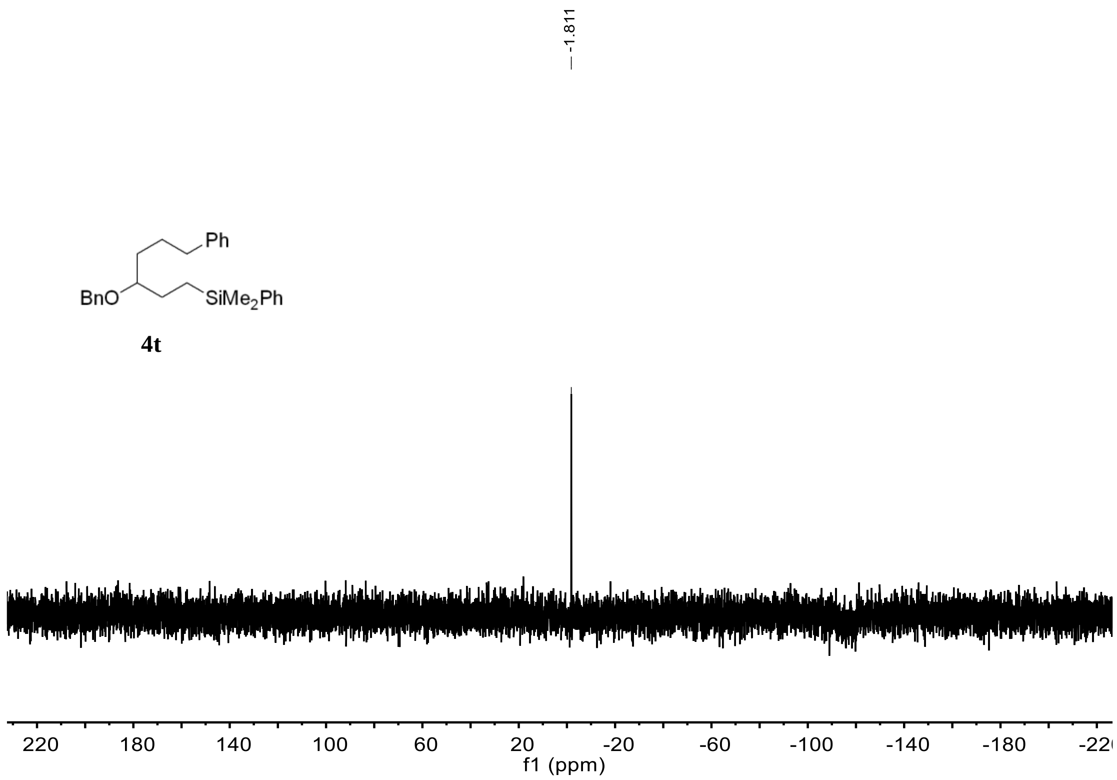

^{29}Si NMR Spectrum of **4t**

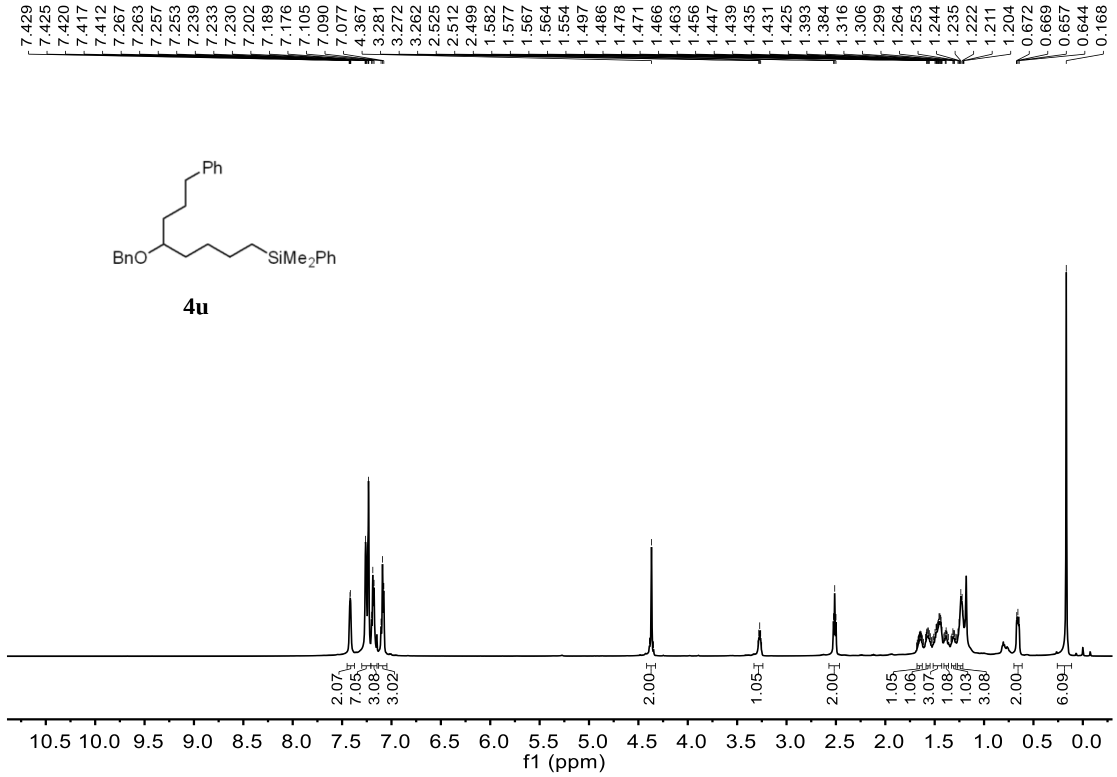

^{1}H NMR Spectrum of **4u**

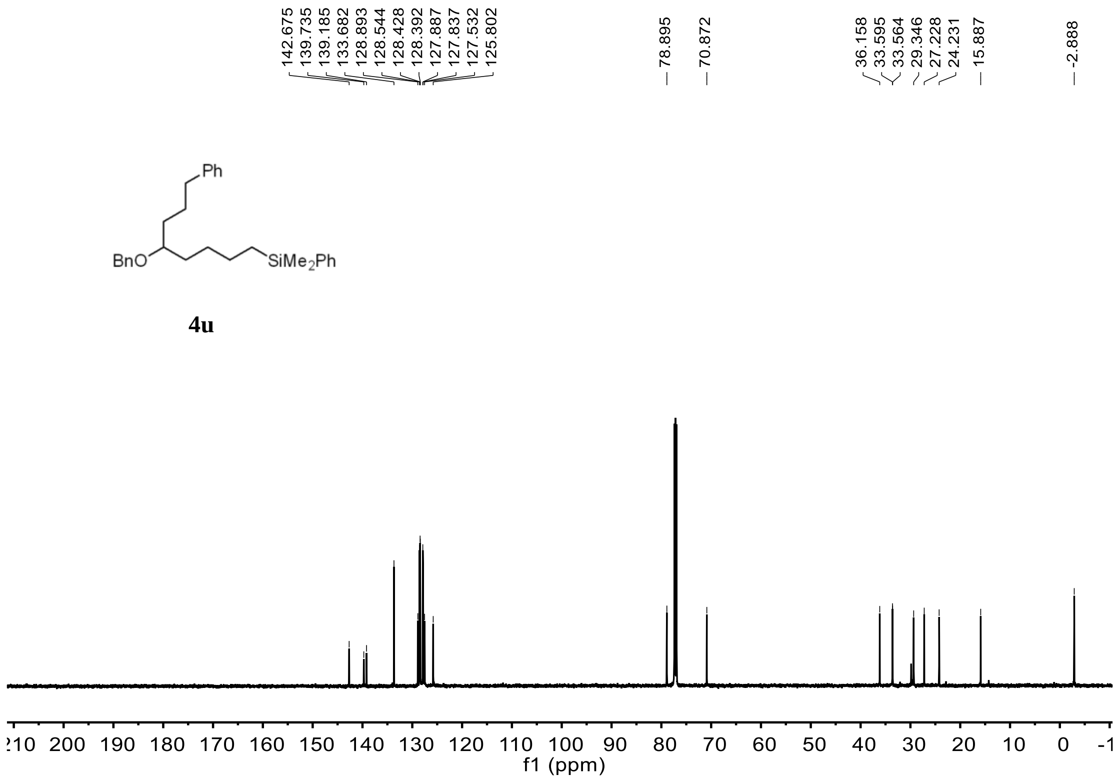

^{13}C NMR Spectrum of **4u**

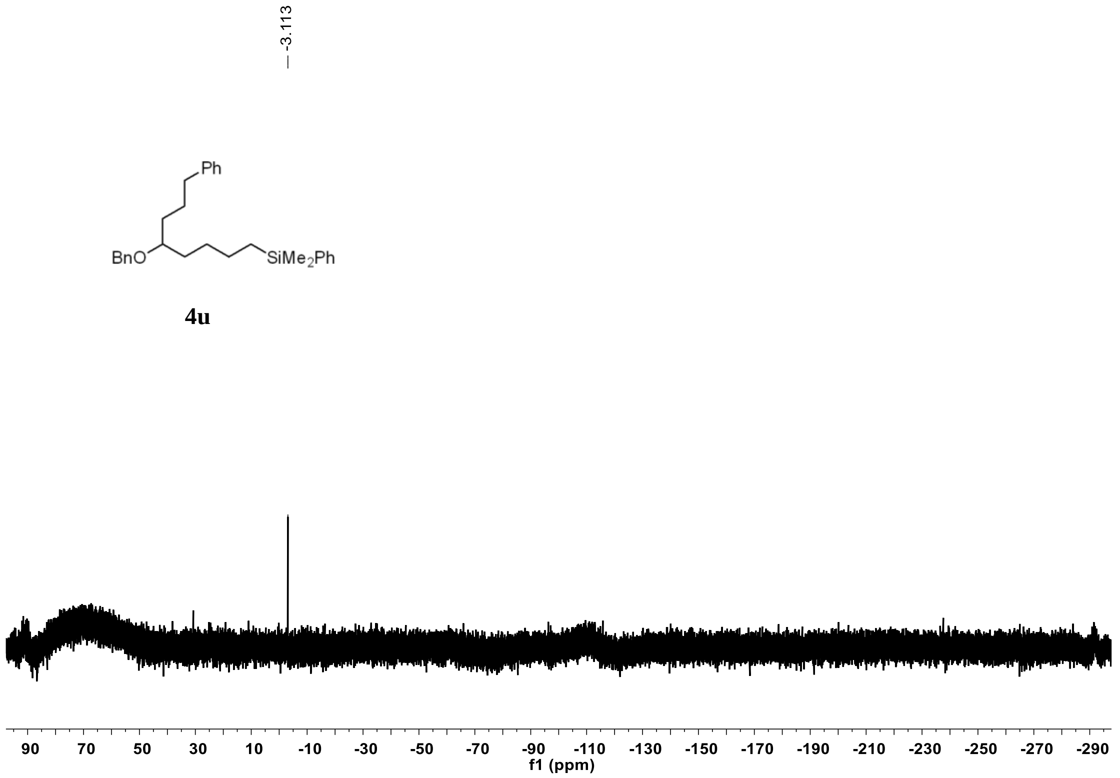

^{29}Si NMR Spectrum of **4u**

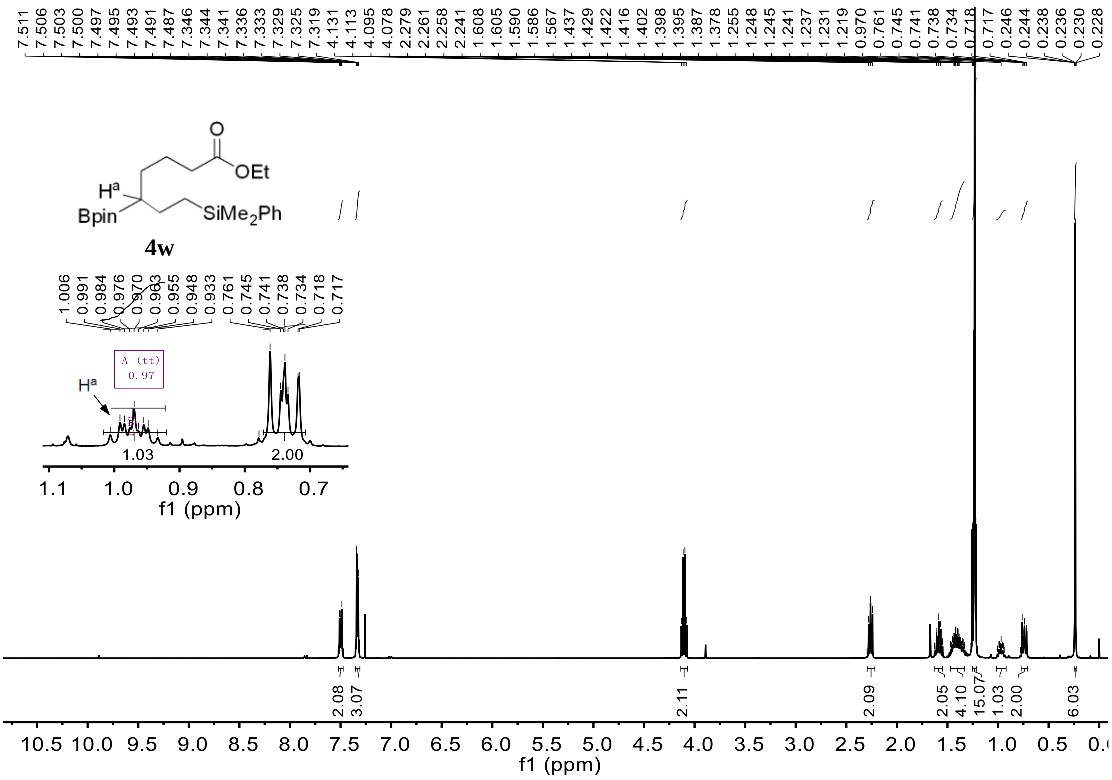

^{1}H NMR Spectrum of **4w**

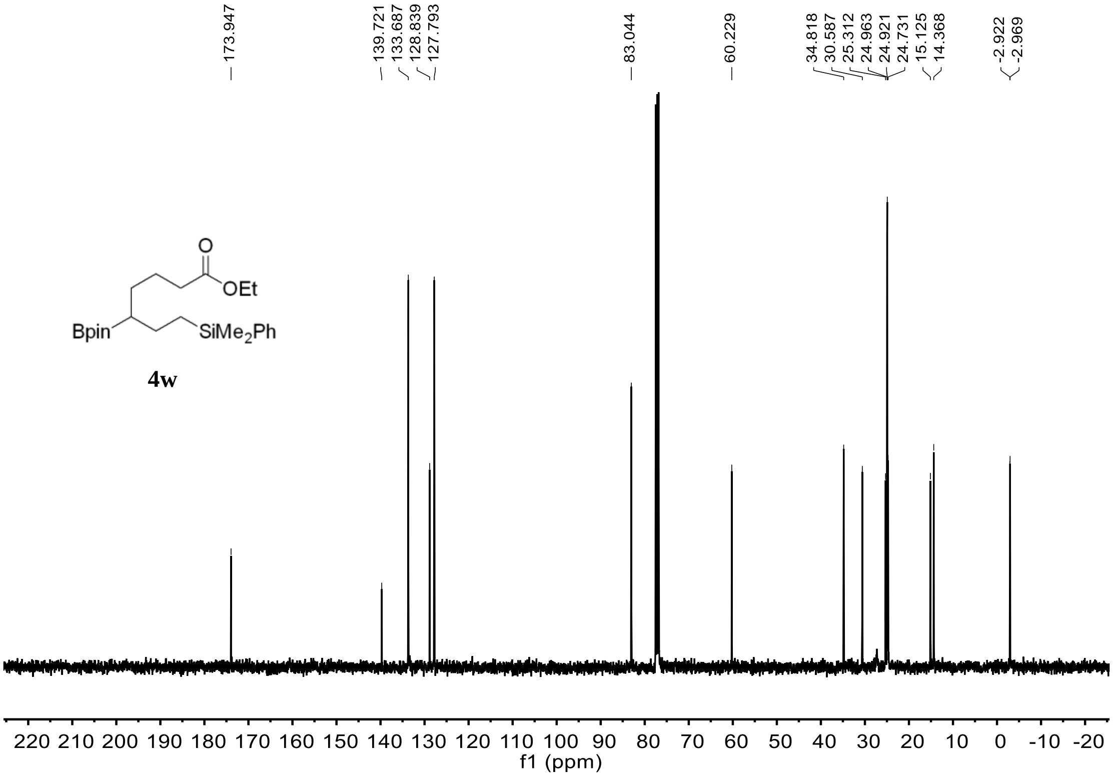

^{13}C NMR Spectrum of **4w**

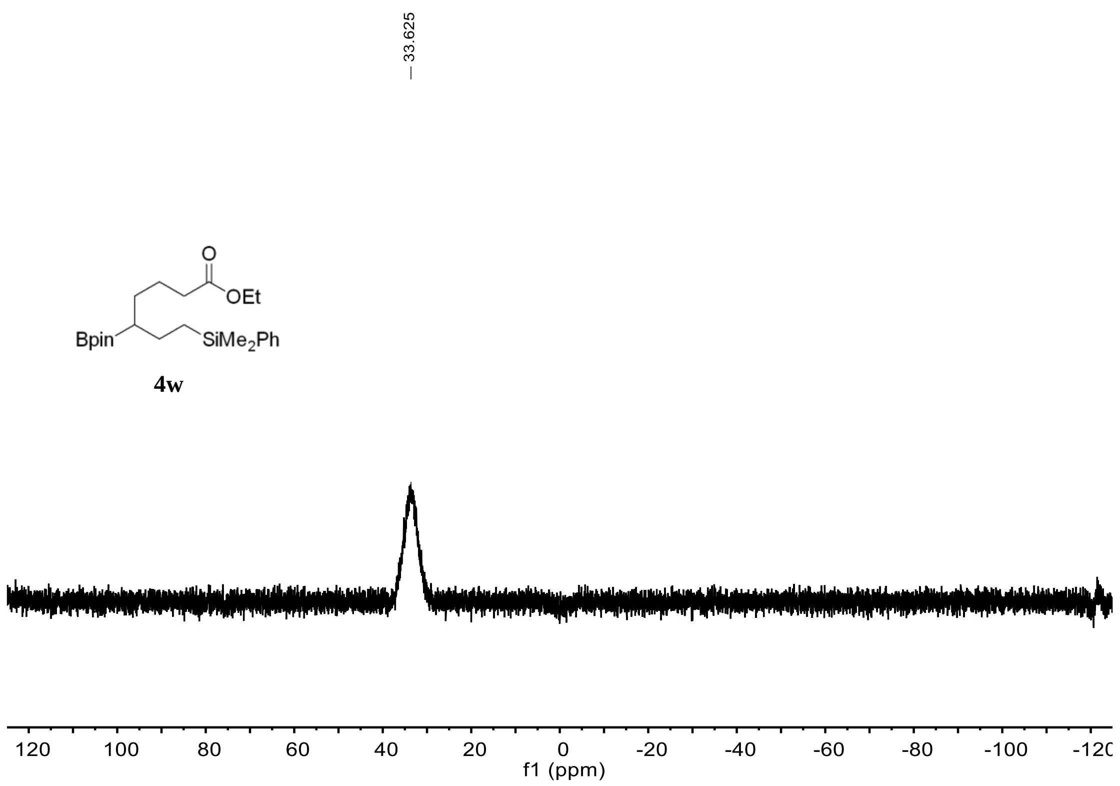

^{11}B NMR Spectrum of **4w**

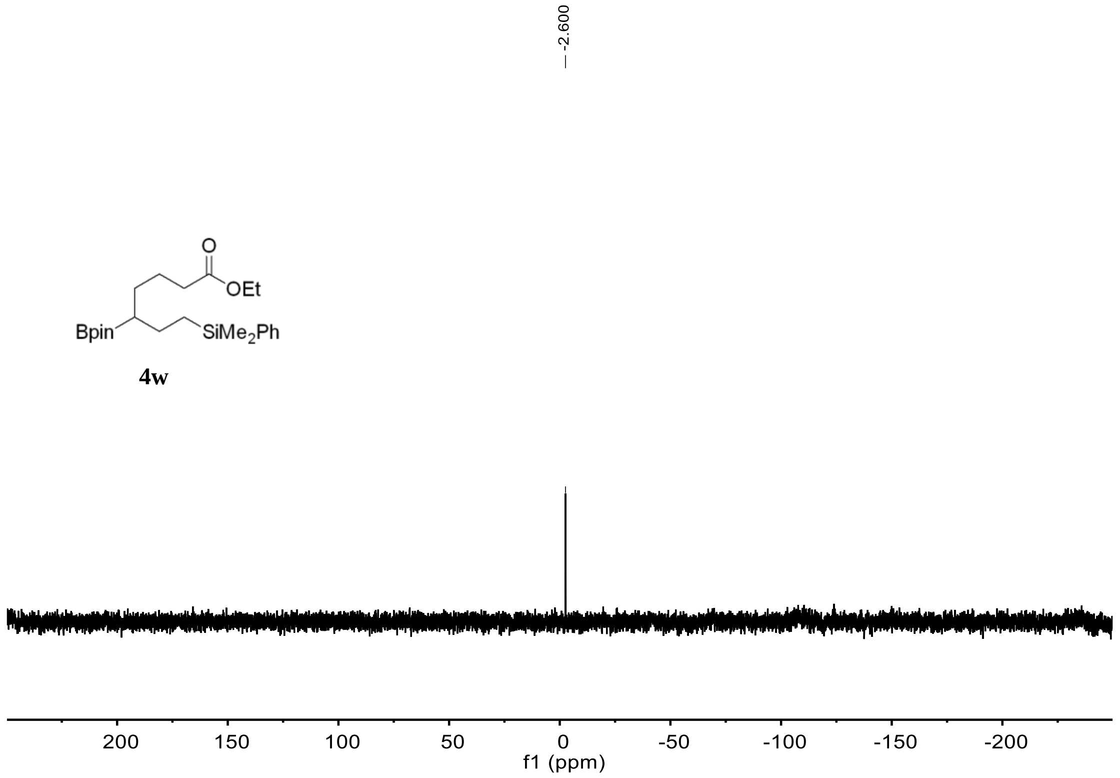

^{29}Si NMR Spectrum of **4w**

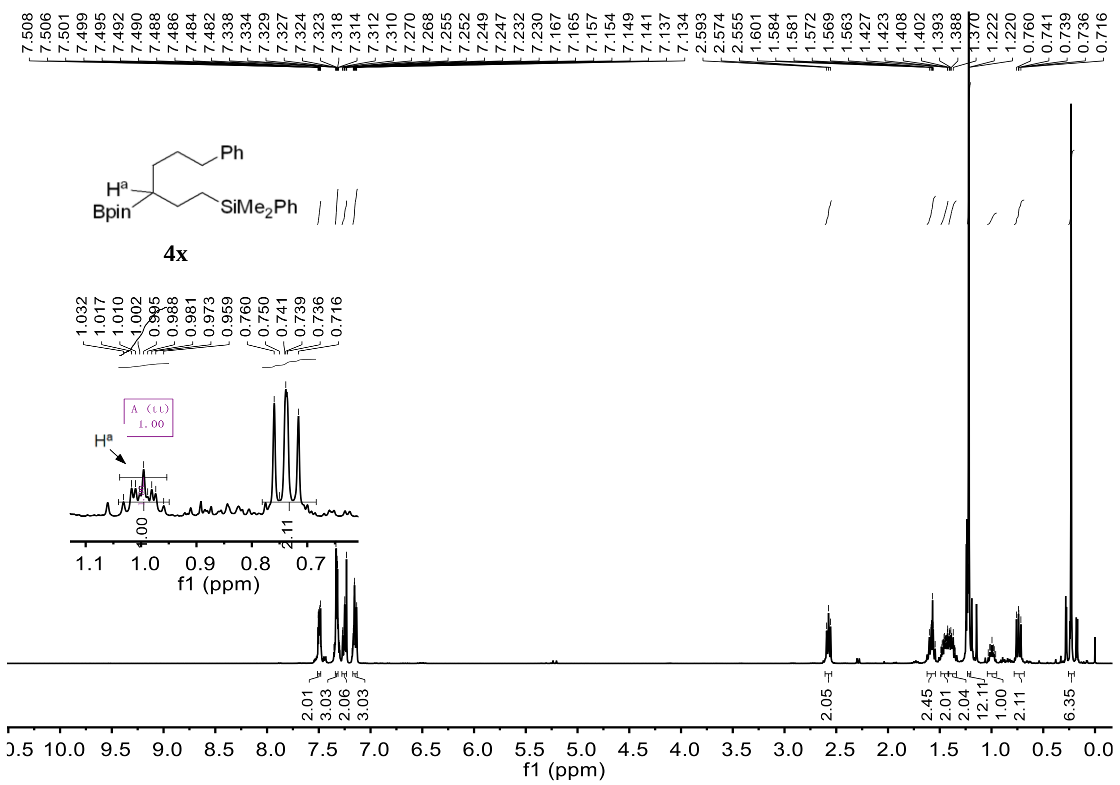

^{1}H NMR Spectrum of **4x**

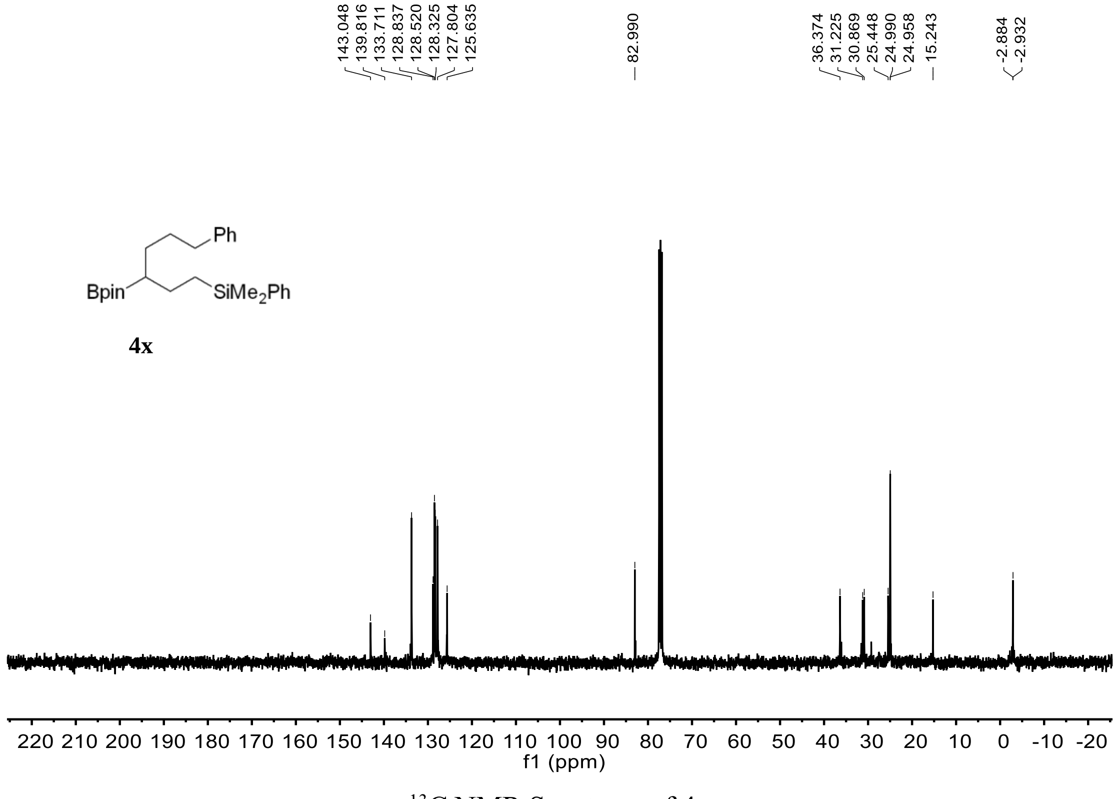

^{13}C NMR Spectrum of **4x**

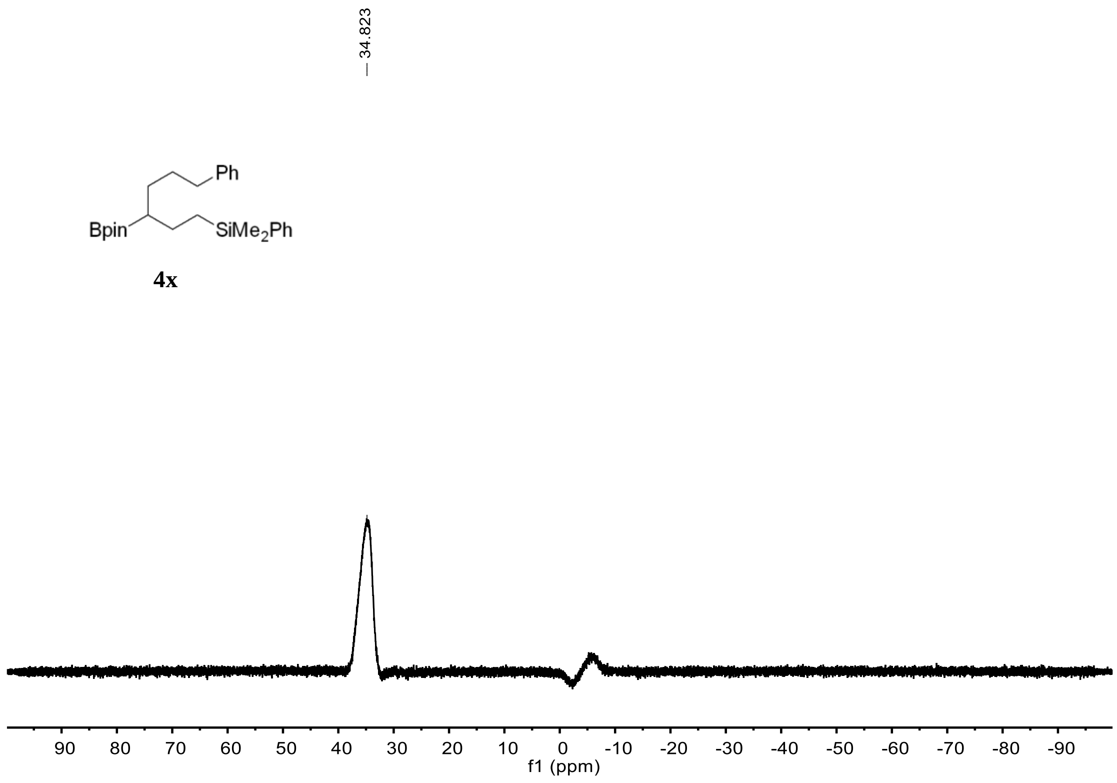

^{11}B NMR Spectrum of **4x**

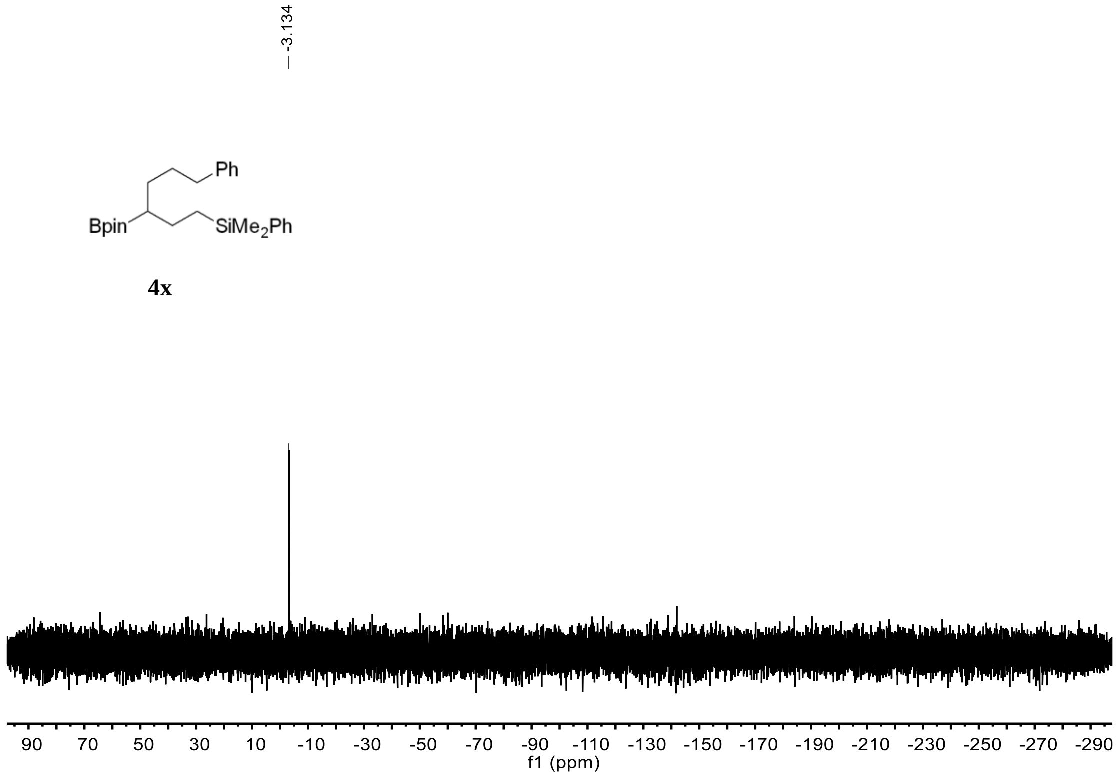

^{29}Si NMR Spectrum of **4x**

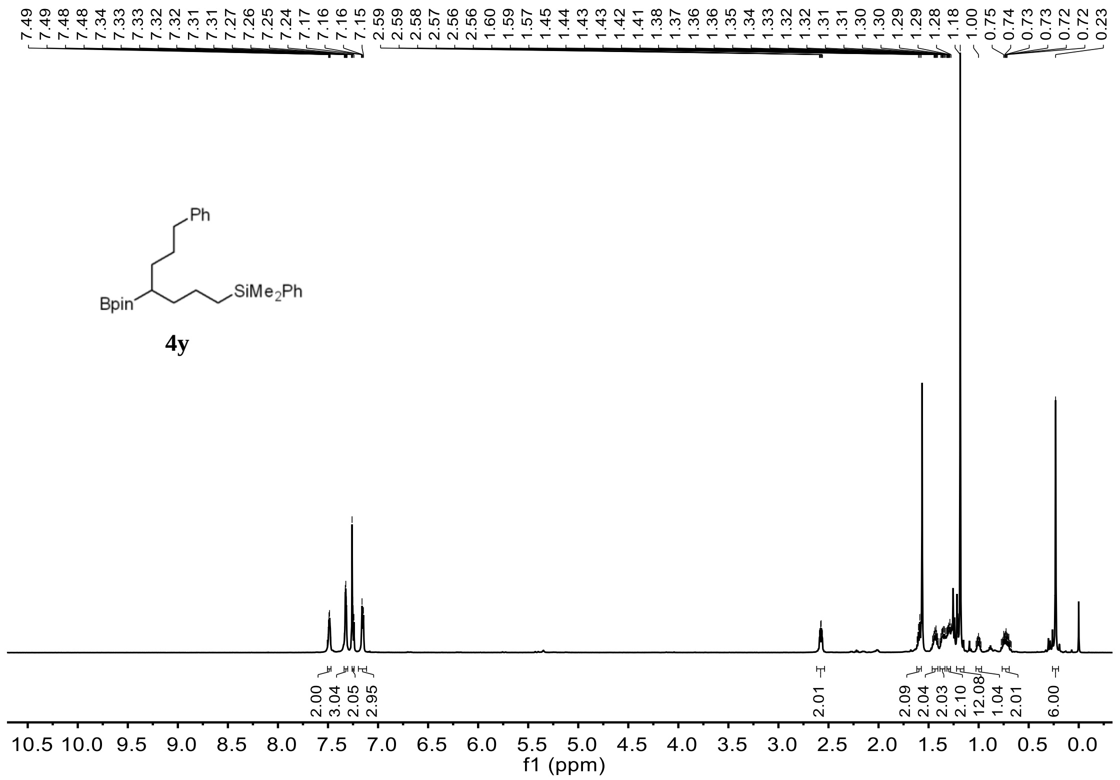

^{1}H NMR Spectrum of **4y**

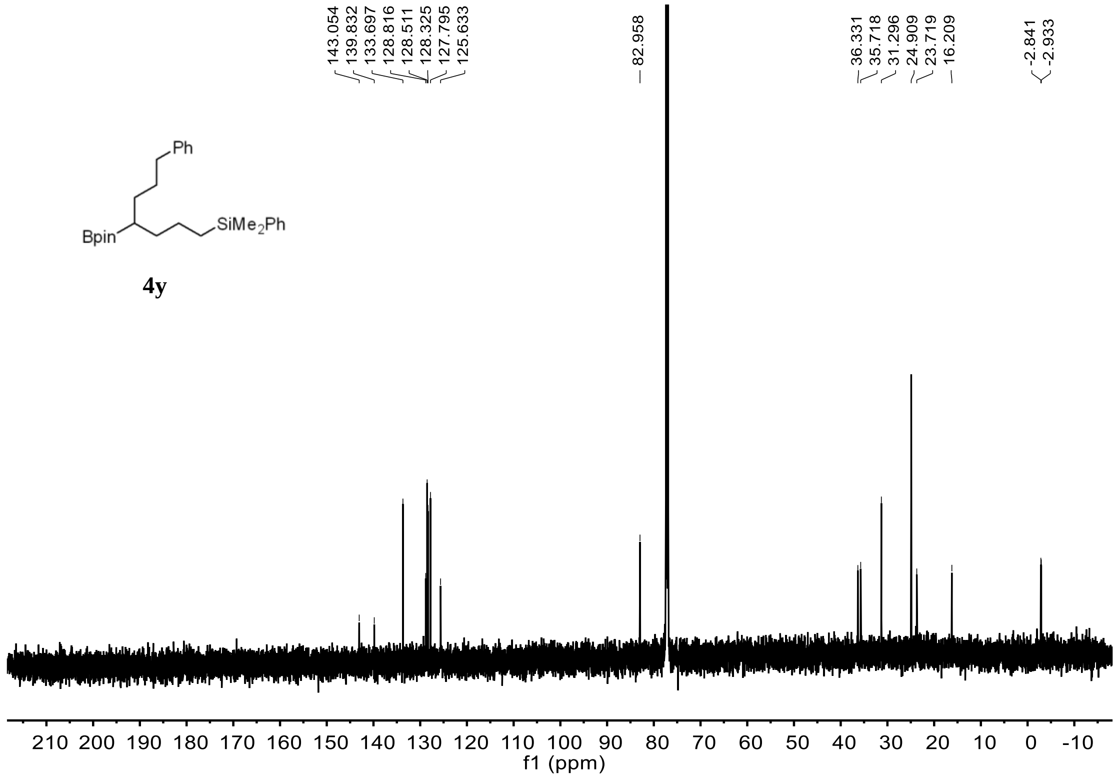

^{13}C NMR Spectrum of **4y**

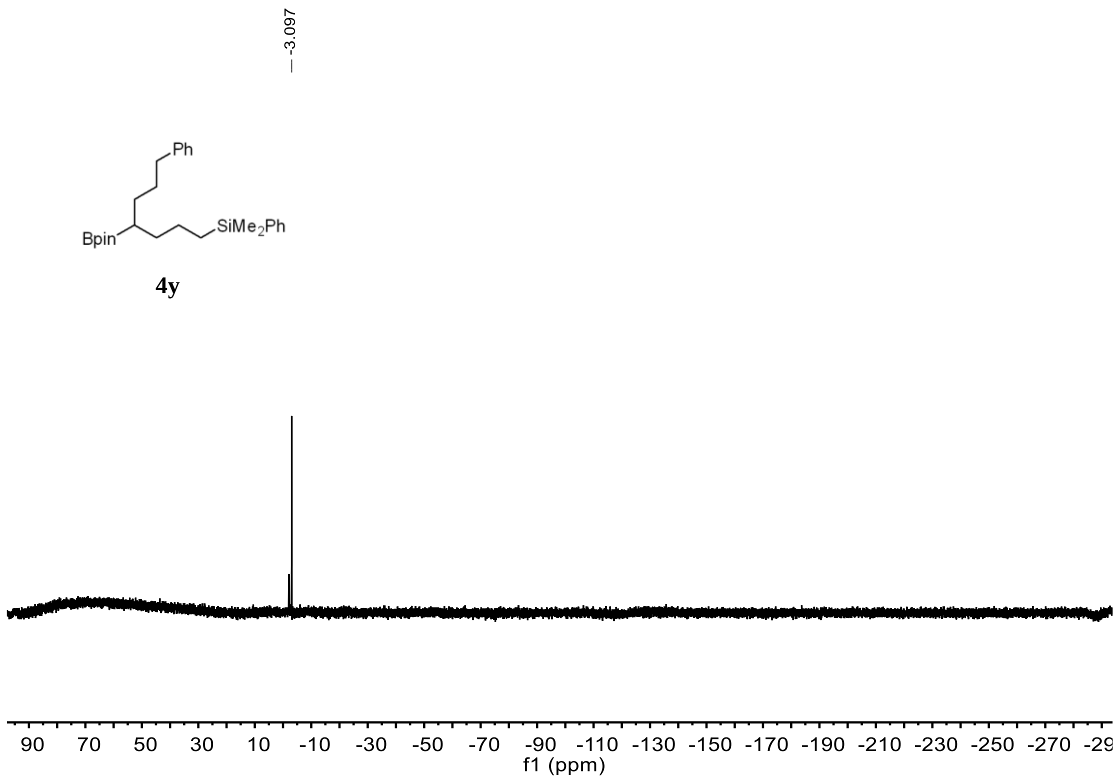

^{29}Si NMR Spectrum of **4y**

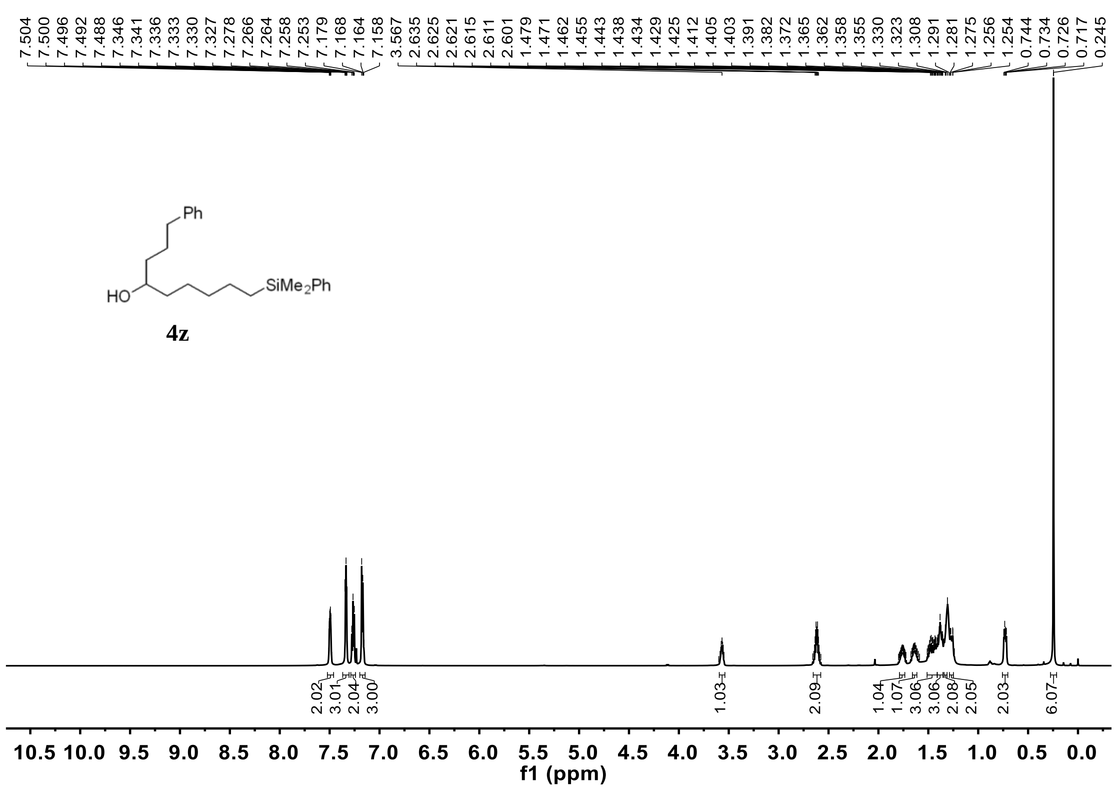

^{1}H NMR Spectrum of **4z**

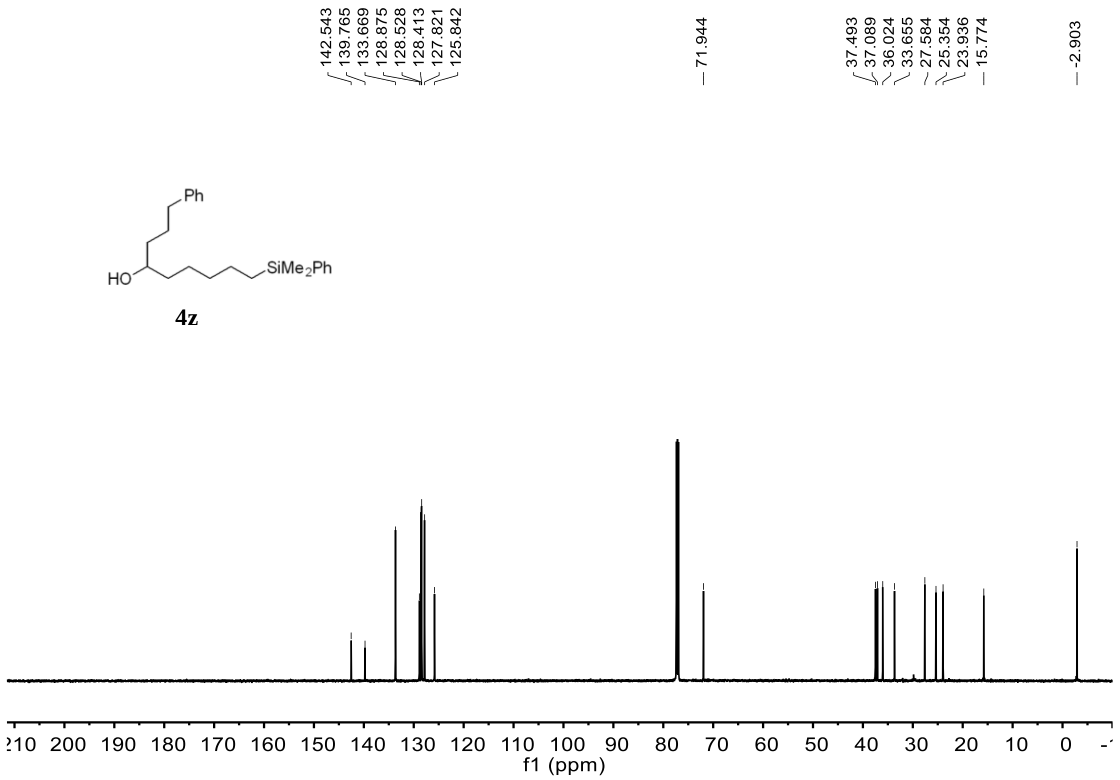

^{13}C NMR Spectrum of **4z**

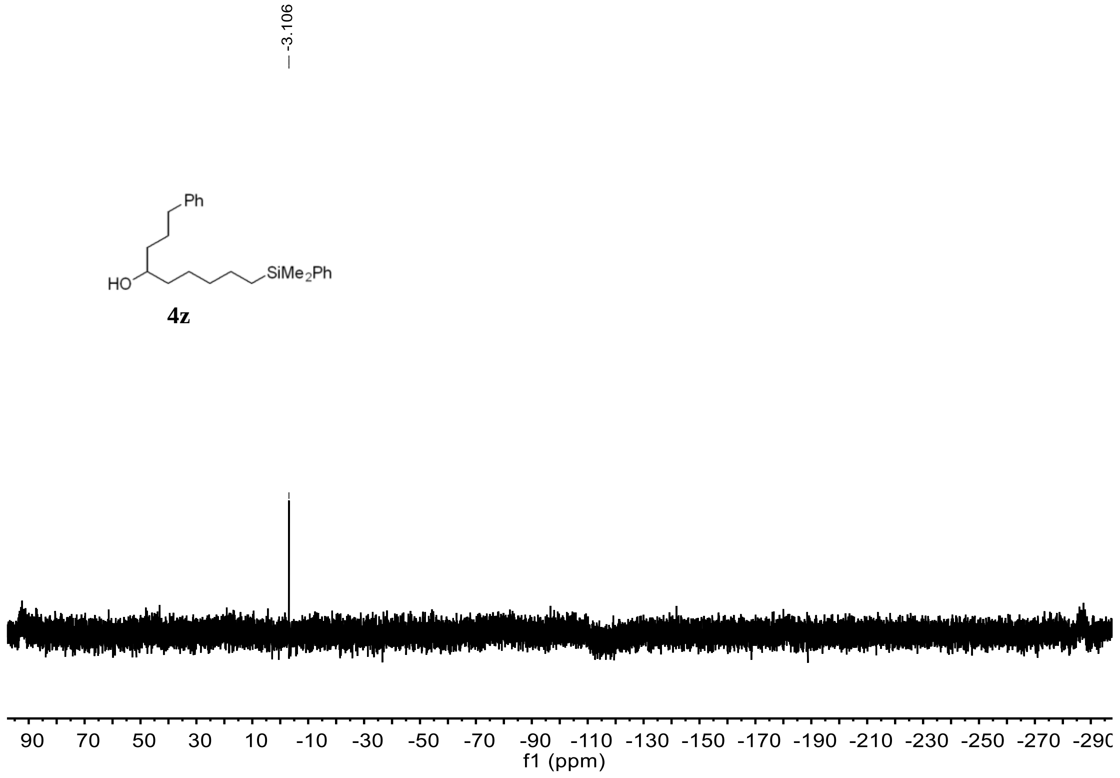

^{29}Si NMR Spectrum of **4z**

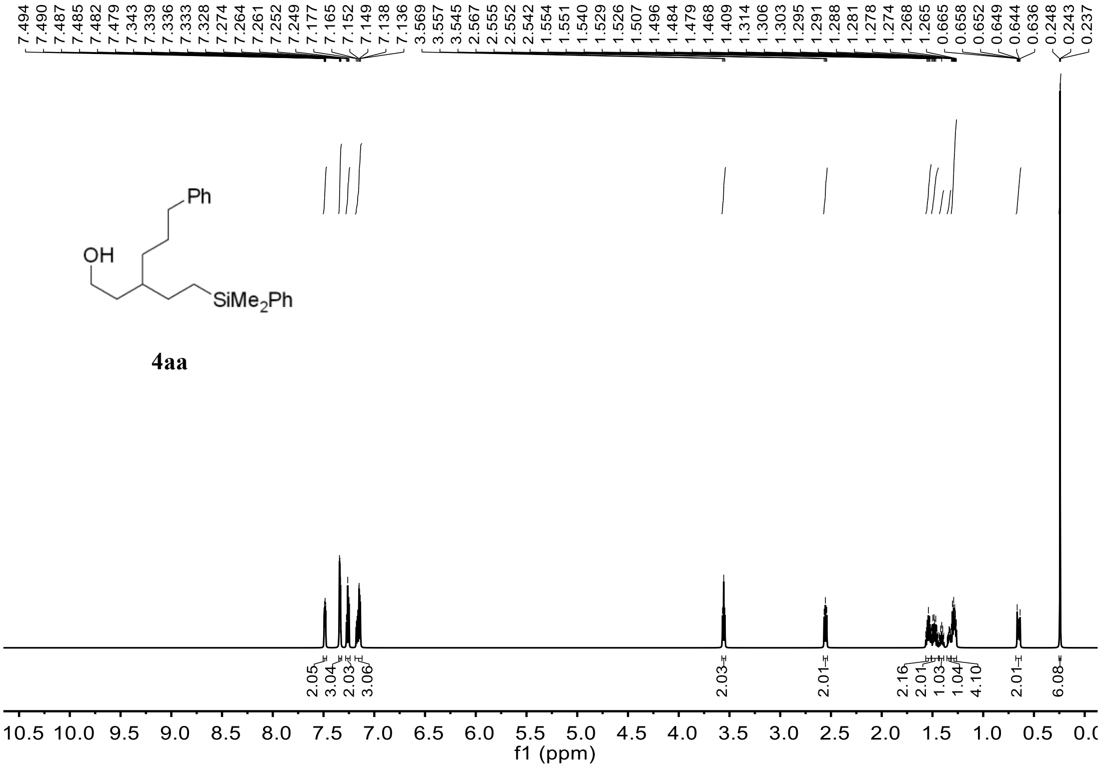

^{1}H NMR Spectrum of **4aa**

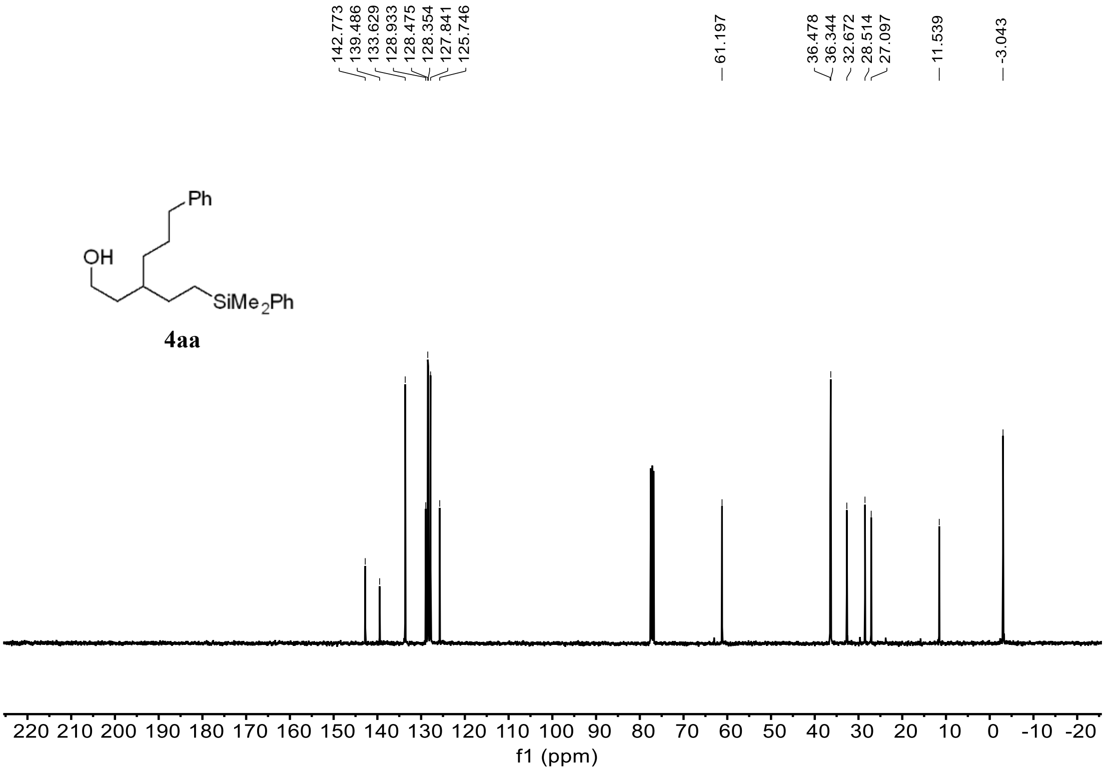

^{13}C NMR Spectrum of **4aa**

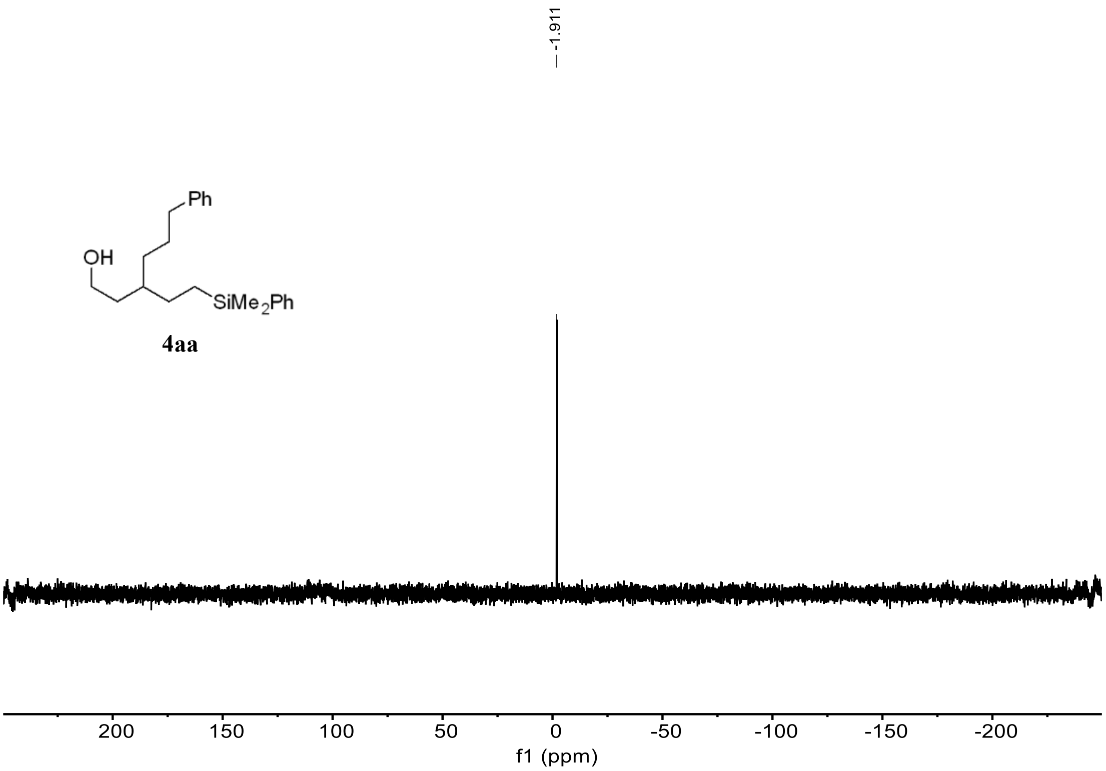

^{29}Si NMR Spectrum of **4aa**

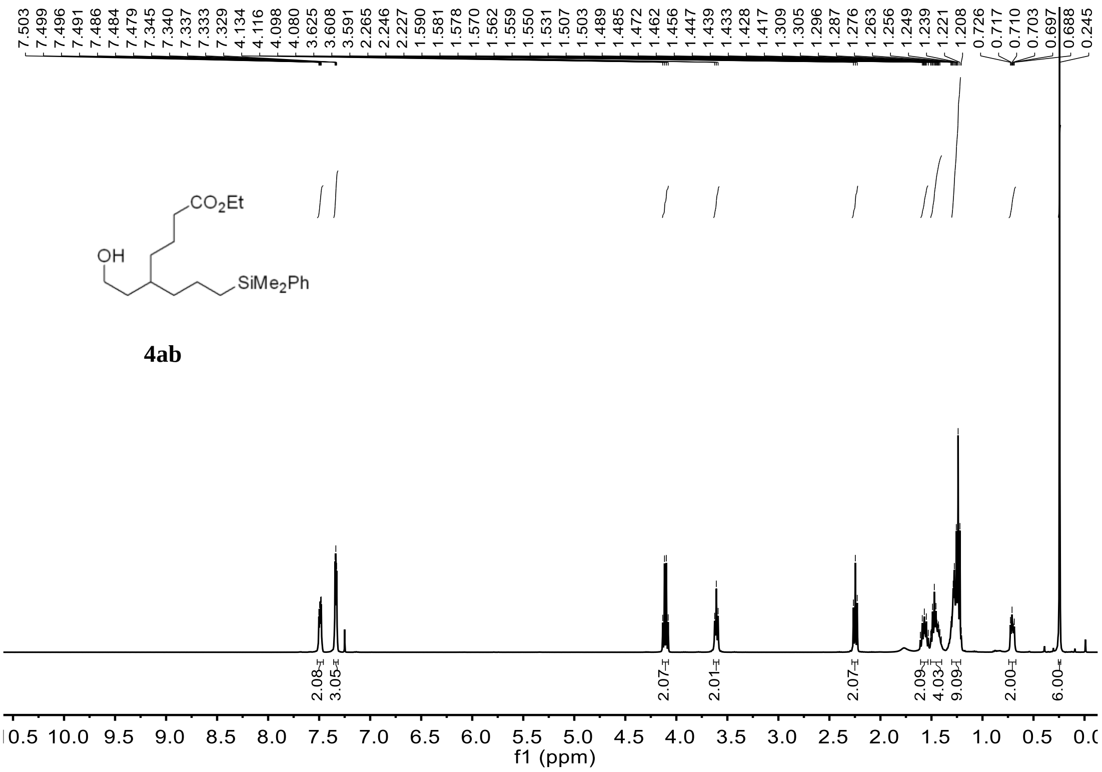

^{1}H NMR Spectrum of **4ab**

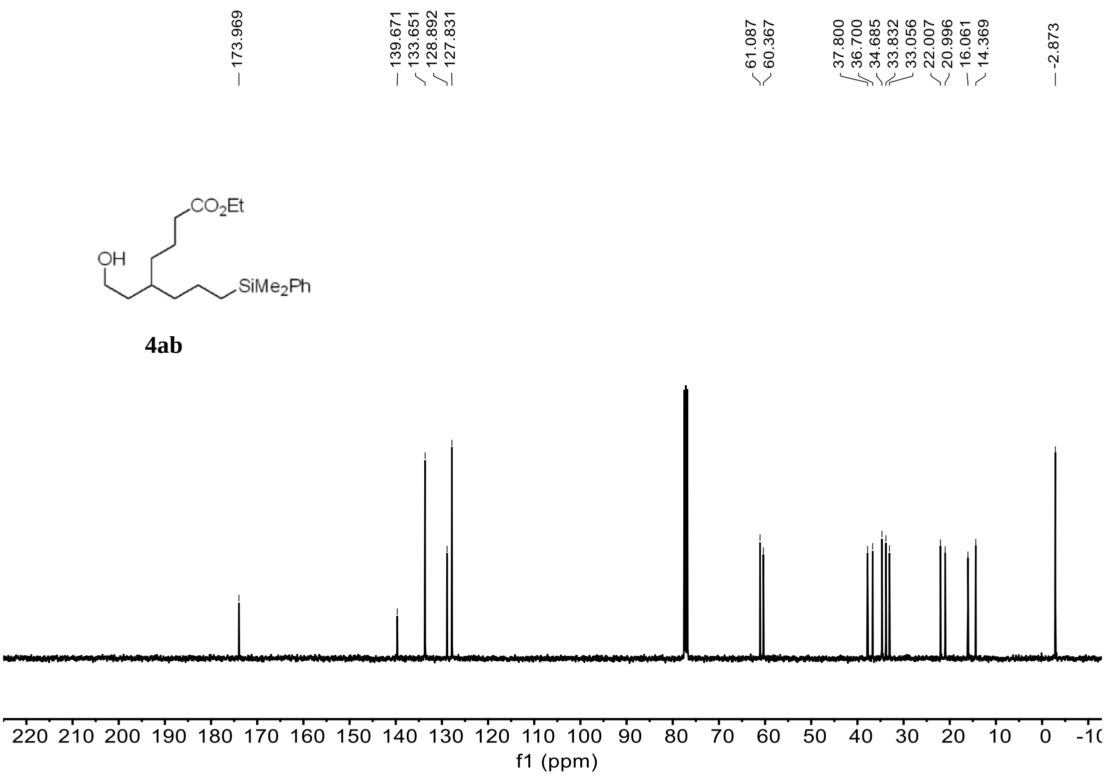

^{13}C NMR Spectrum of **4ab**

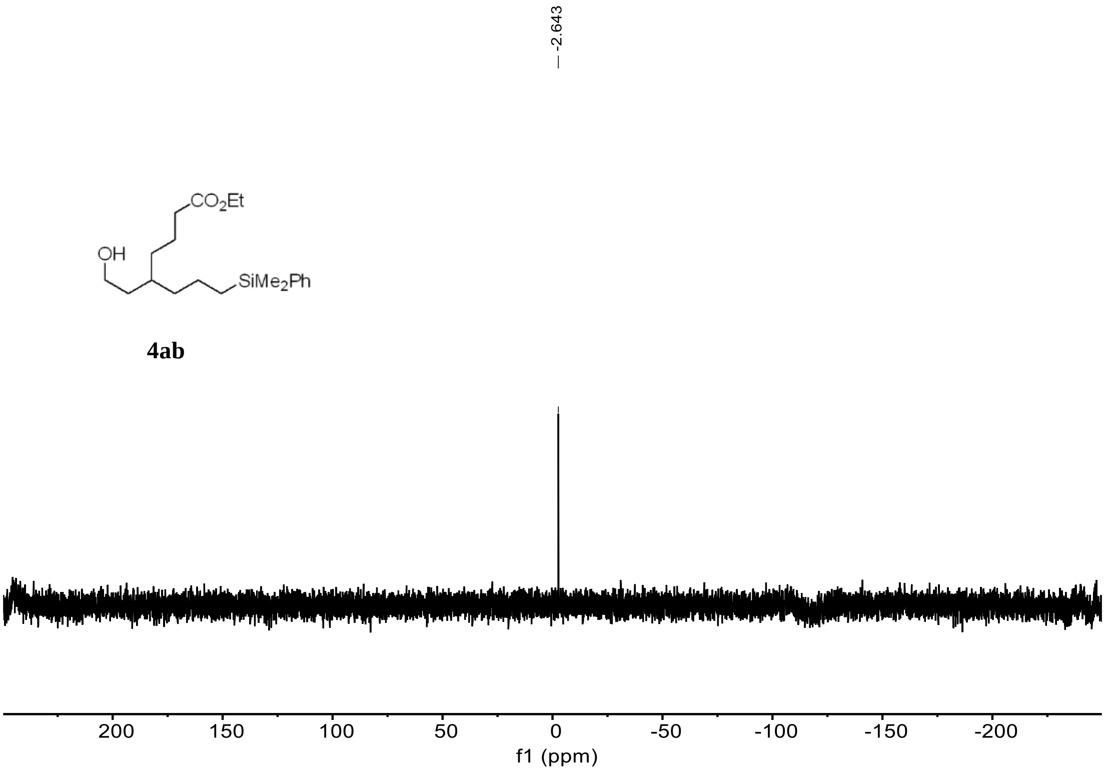

^{29}Si NMR Spectrum of **4ab**

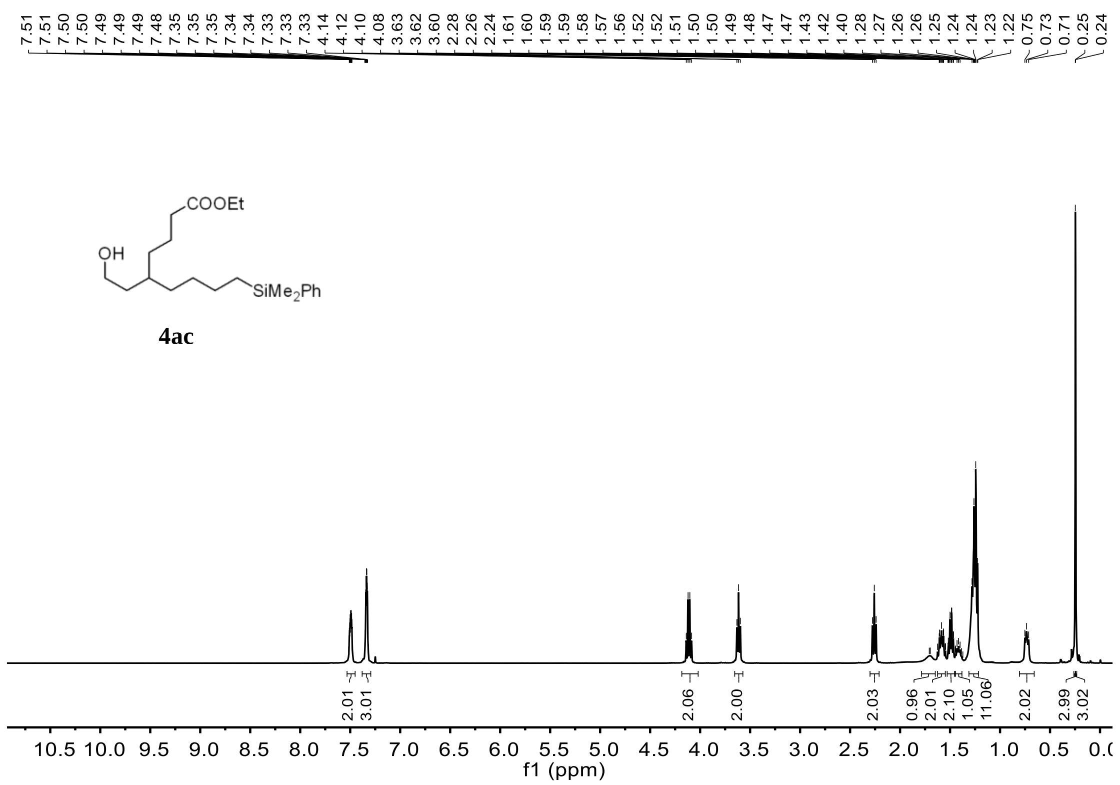

^{1}H NMR Spectrum of **4ac**

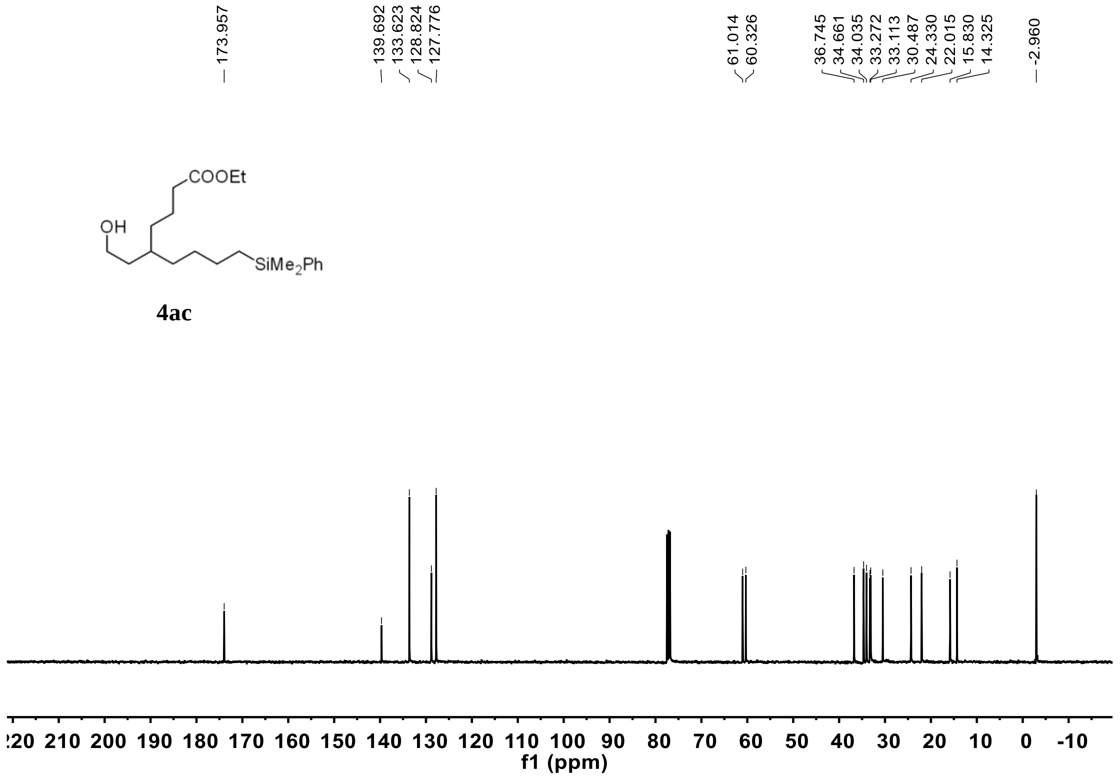

^{13}C NMR Spectrum of **4ac**

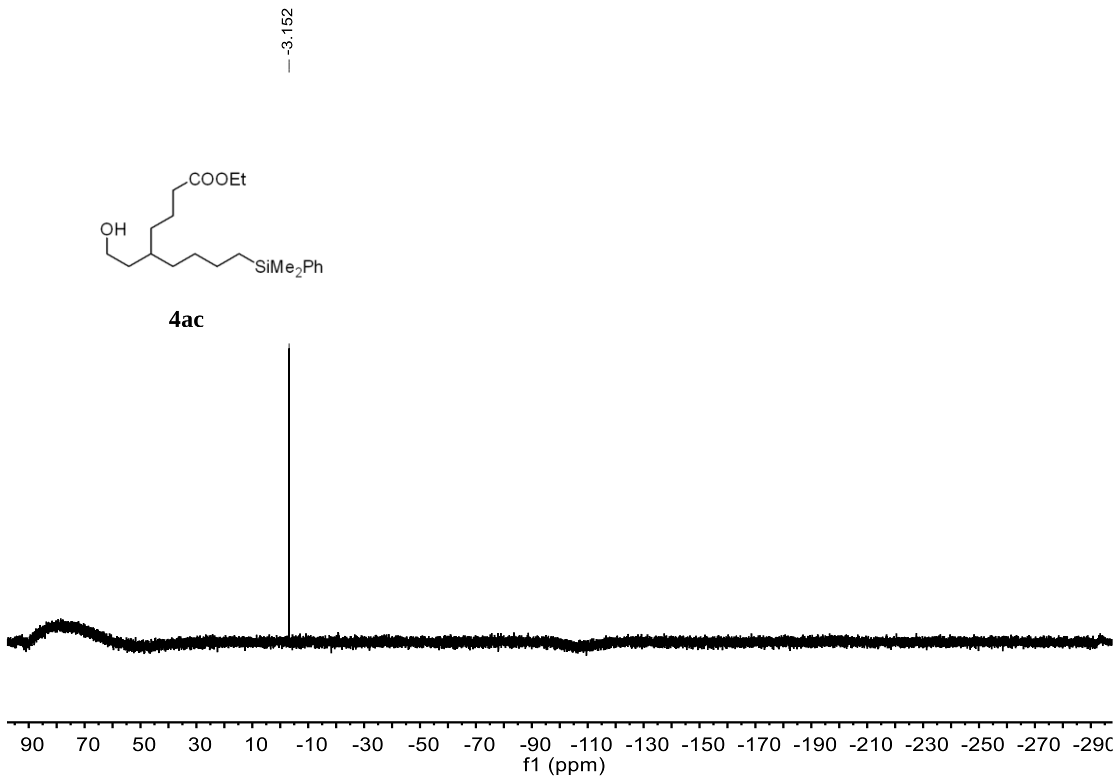

4ac

^{29}Si NMR Spectrum of **4ac**

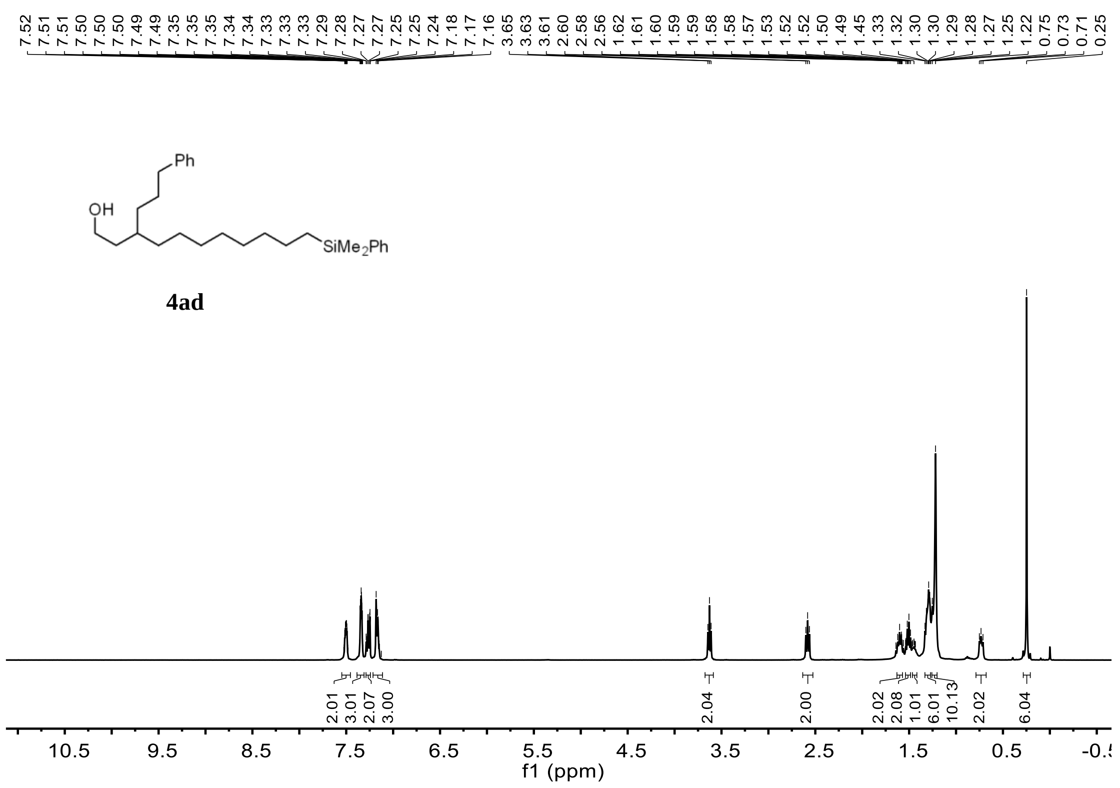

4ad

^{1}H NMR Spectrum of **4ad**

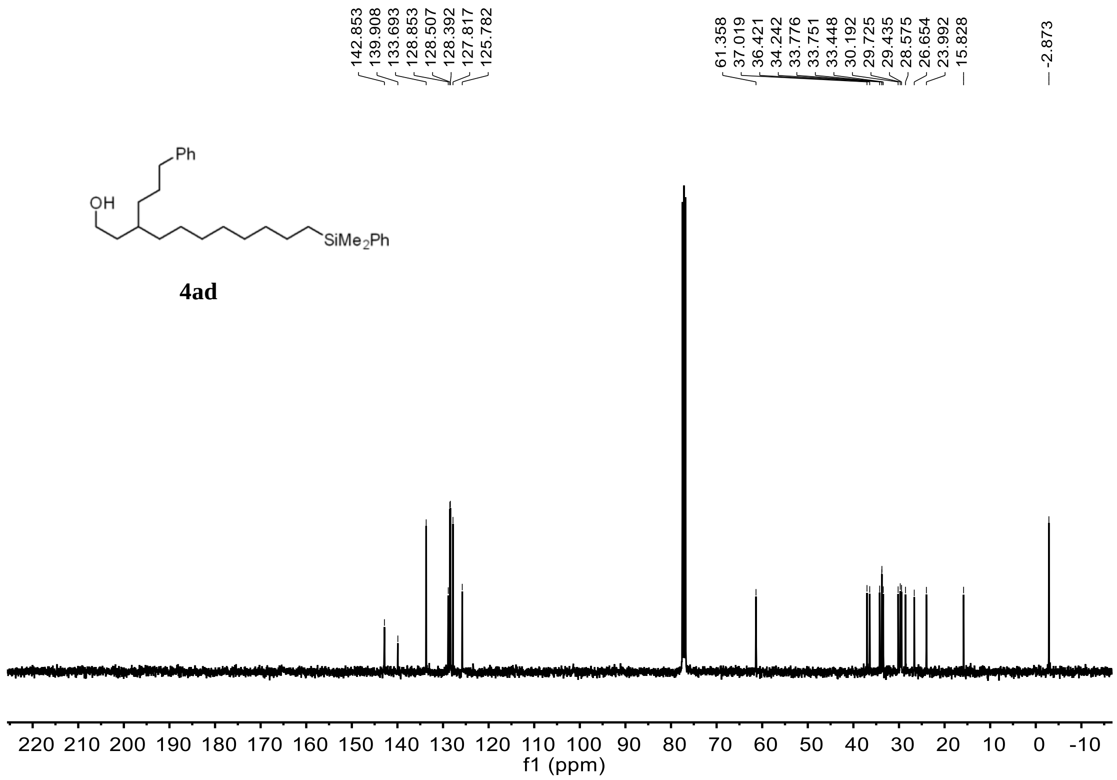

^{13}C NMR Spectrum of **4ad**

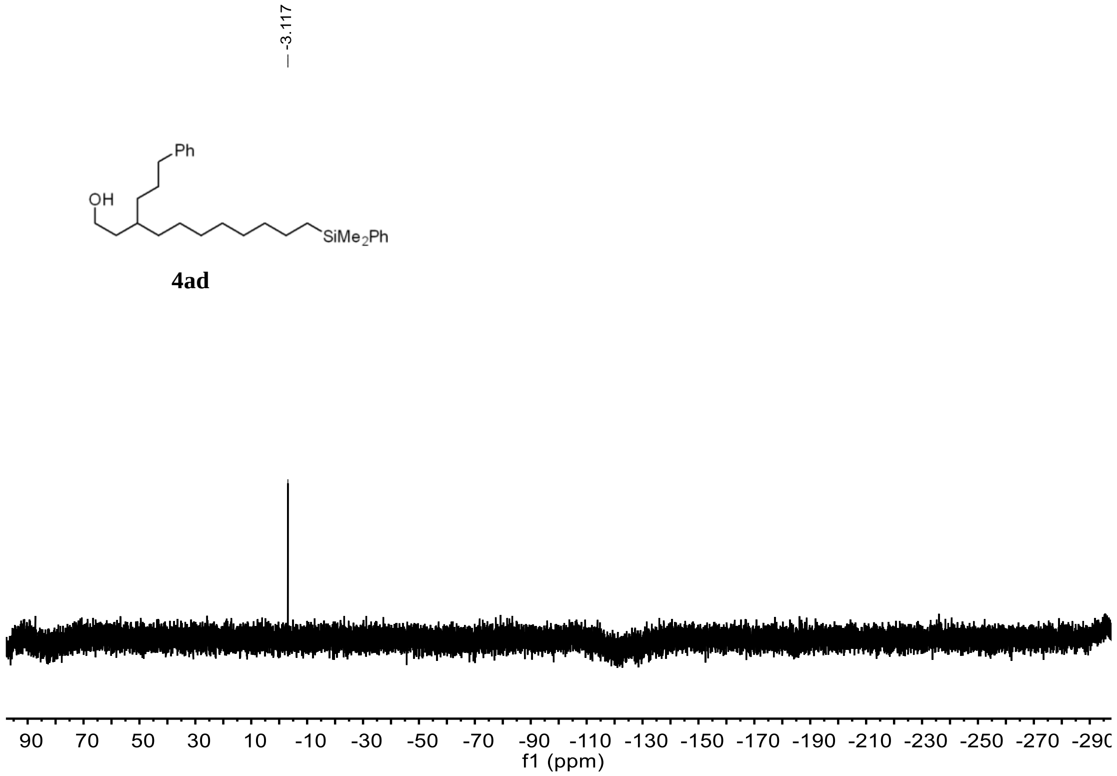

^{29}Si NMR Spectrum of **4ad**

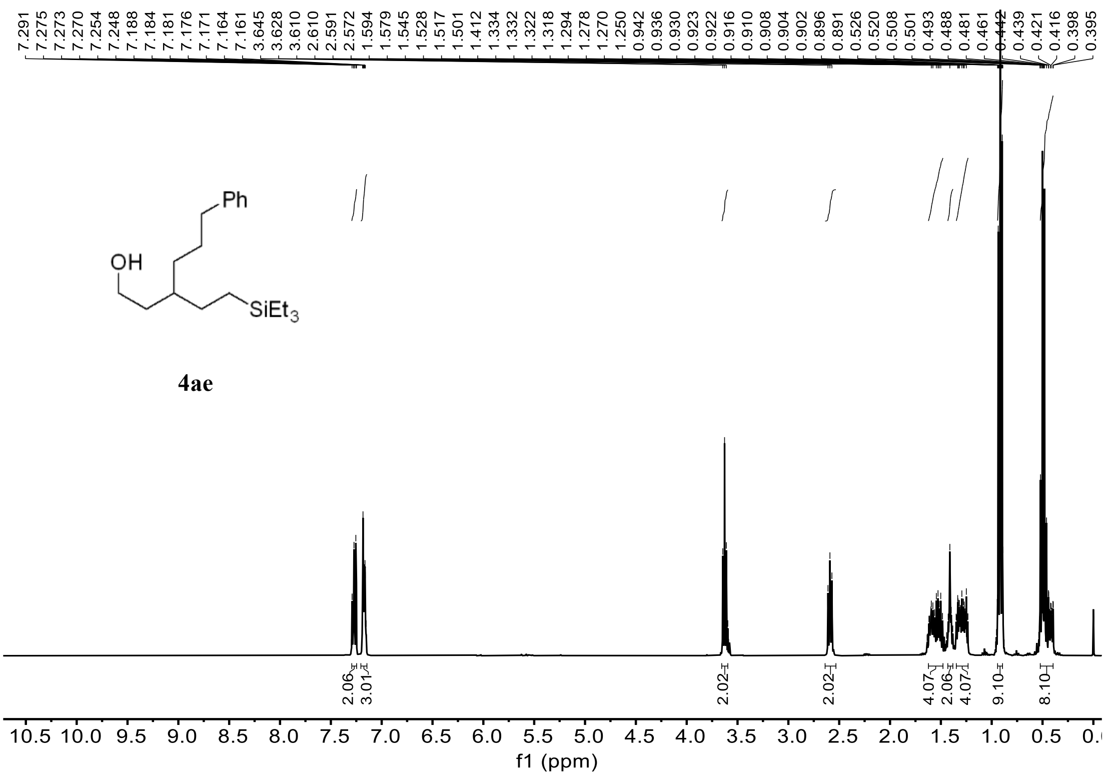

^{1}H NMR Spectrum of **4ae**

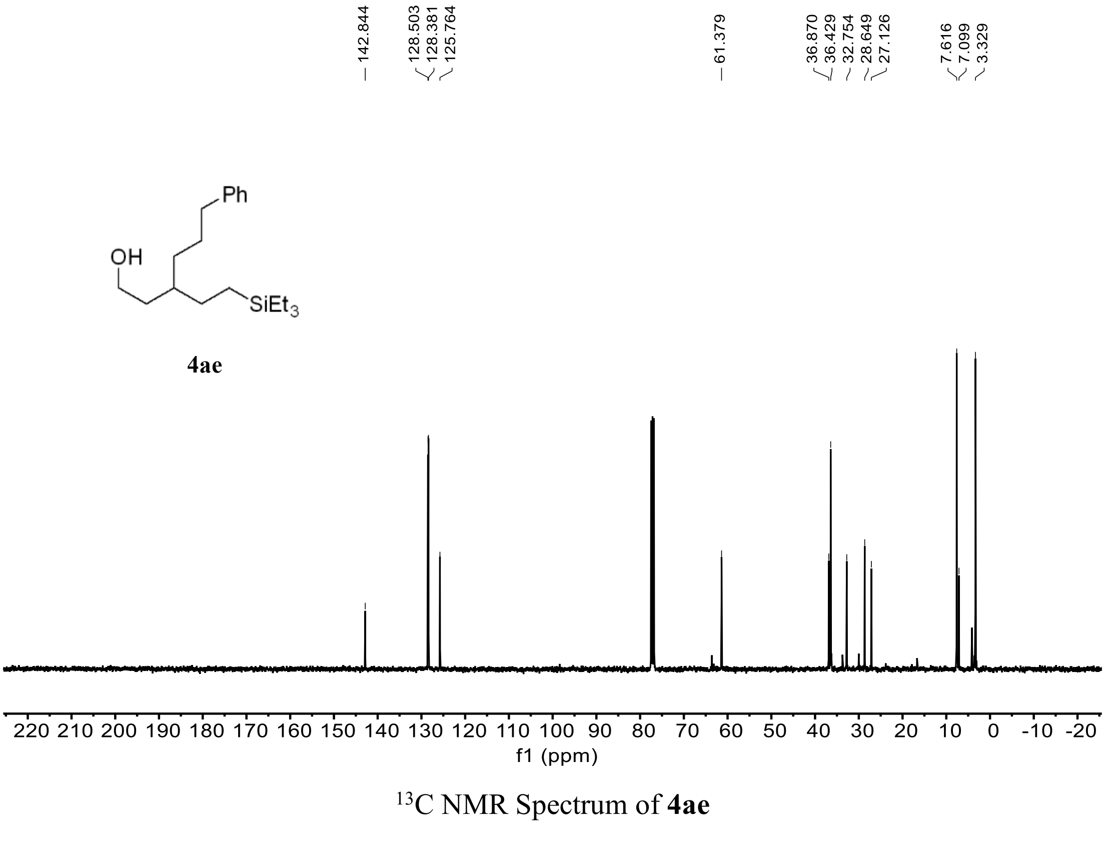

^{13}C NMR Spectrum of **4ae**

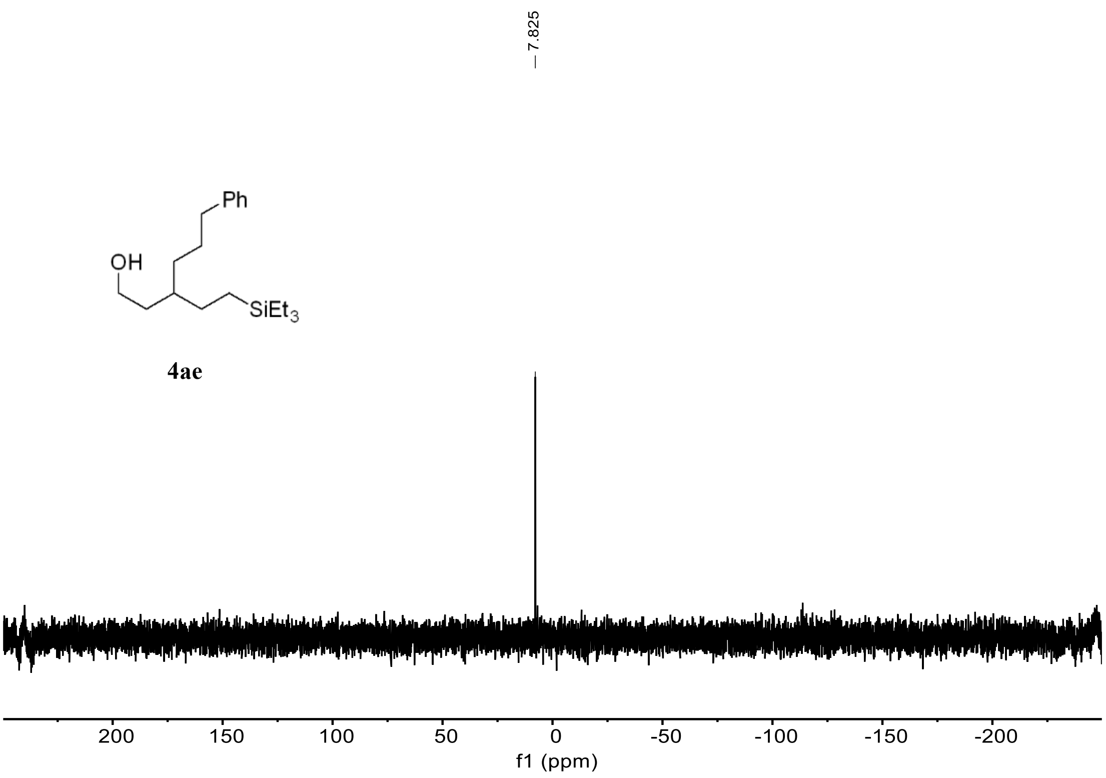

^{29}Si NMR Spectrum of **4ae**

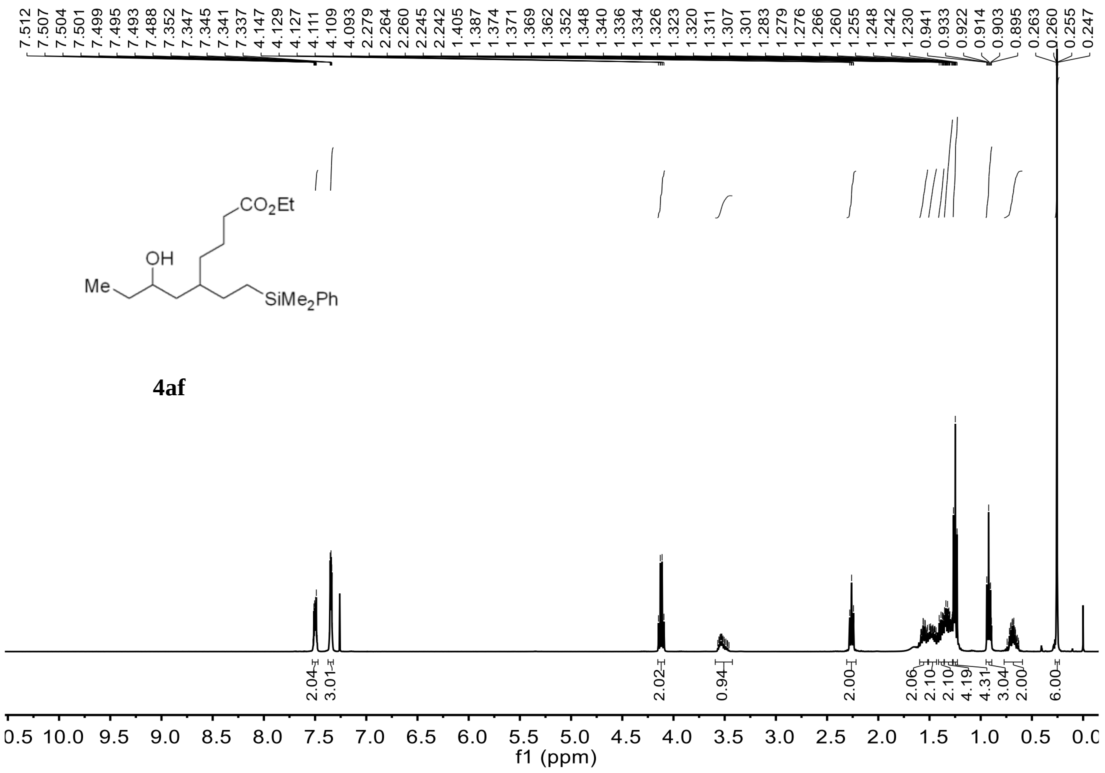

^{1}H NMR Spectrum of **4af**

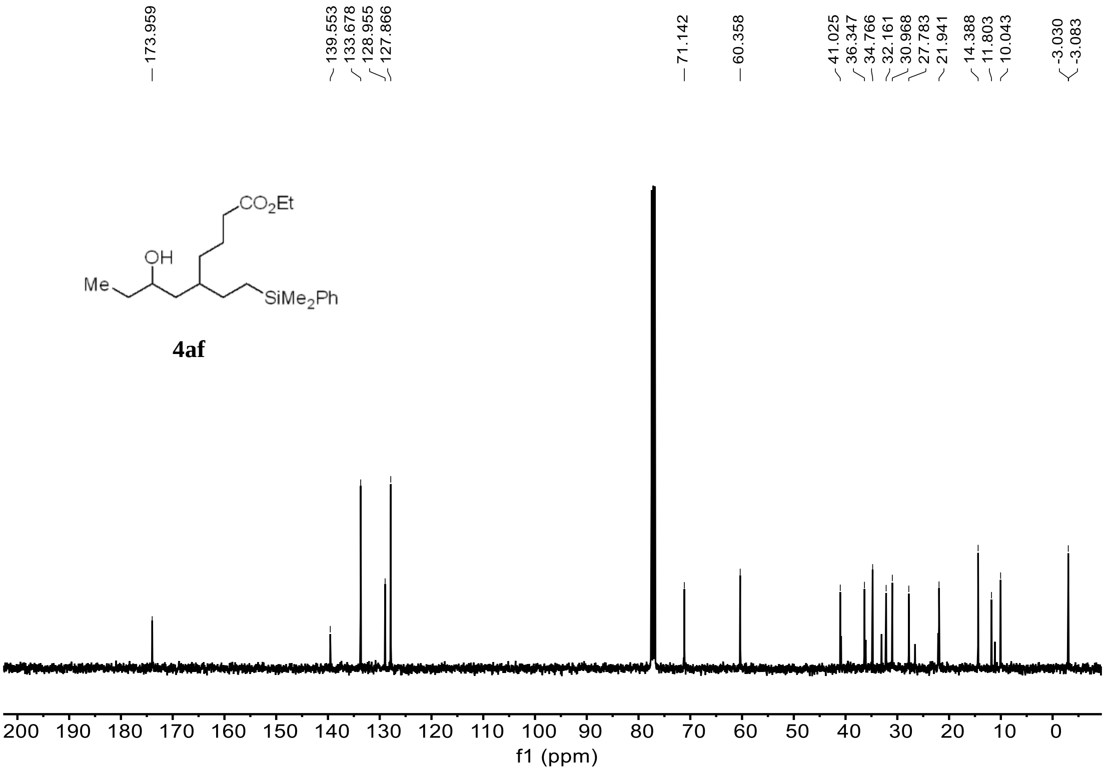

^{13}C NMR Spectrum of **4af**

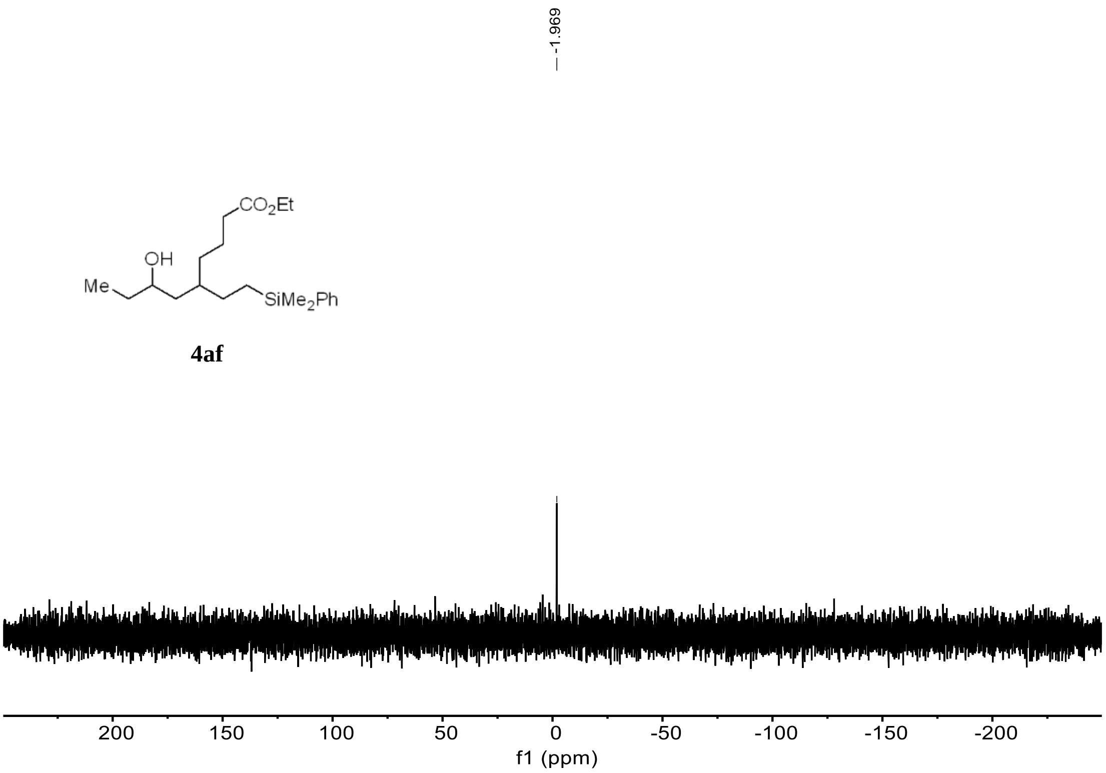

^{29}Si NMR Spectrum of **4af**

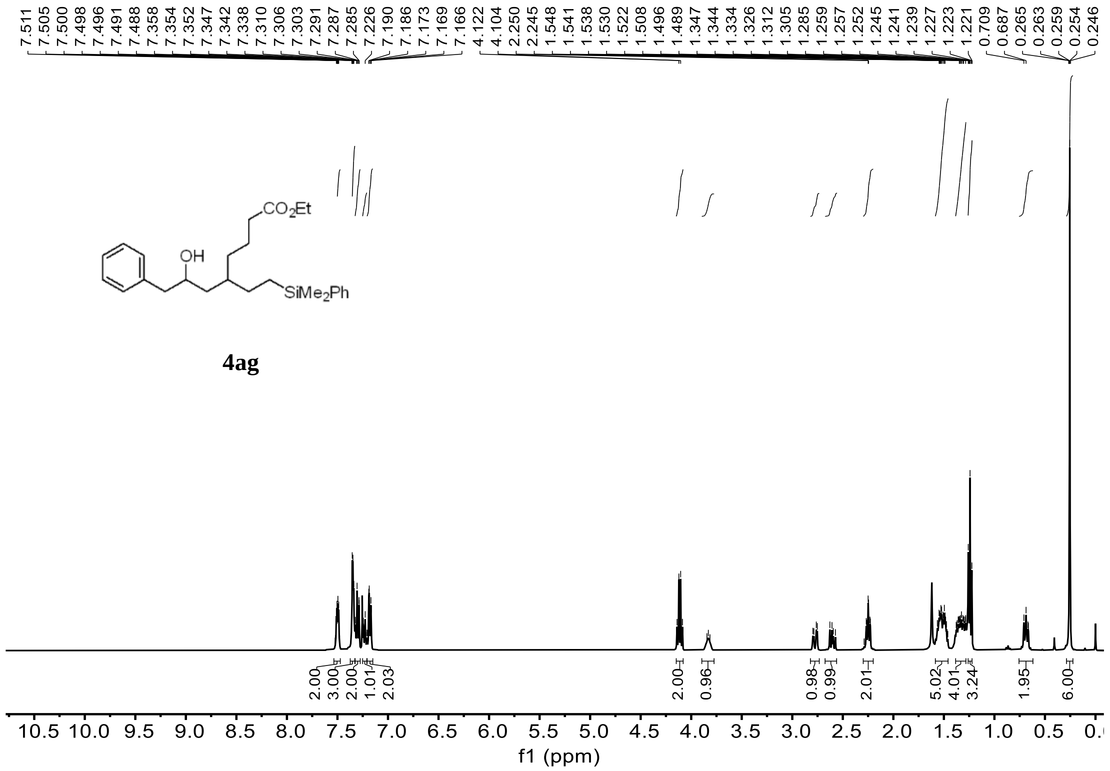

^{1}H NMR Spectrum of **4ag**

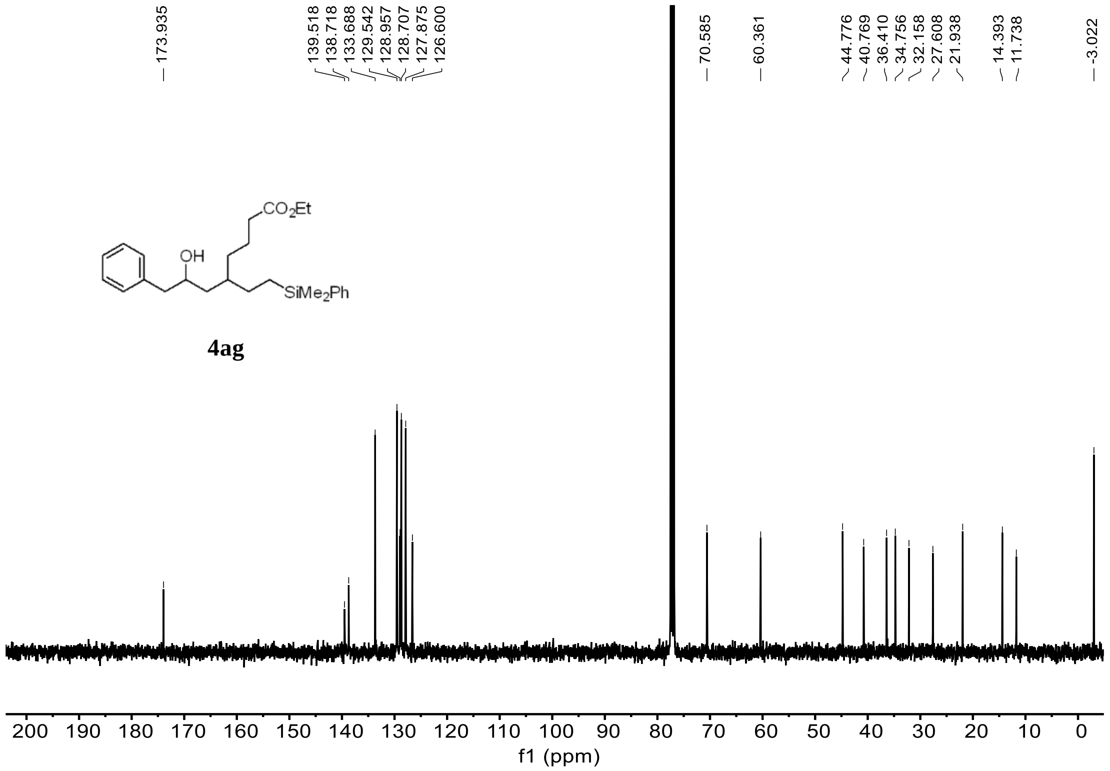

^{13}C NMR Spectrum of **4ag**

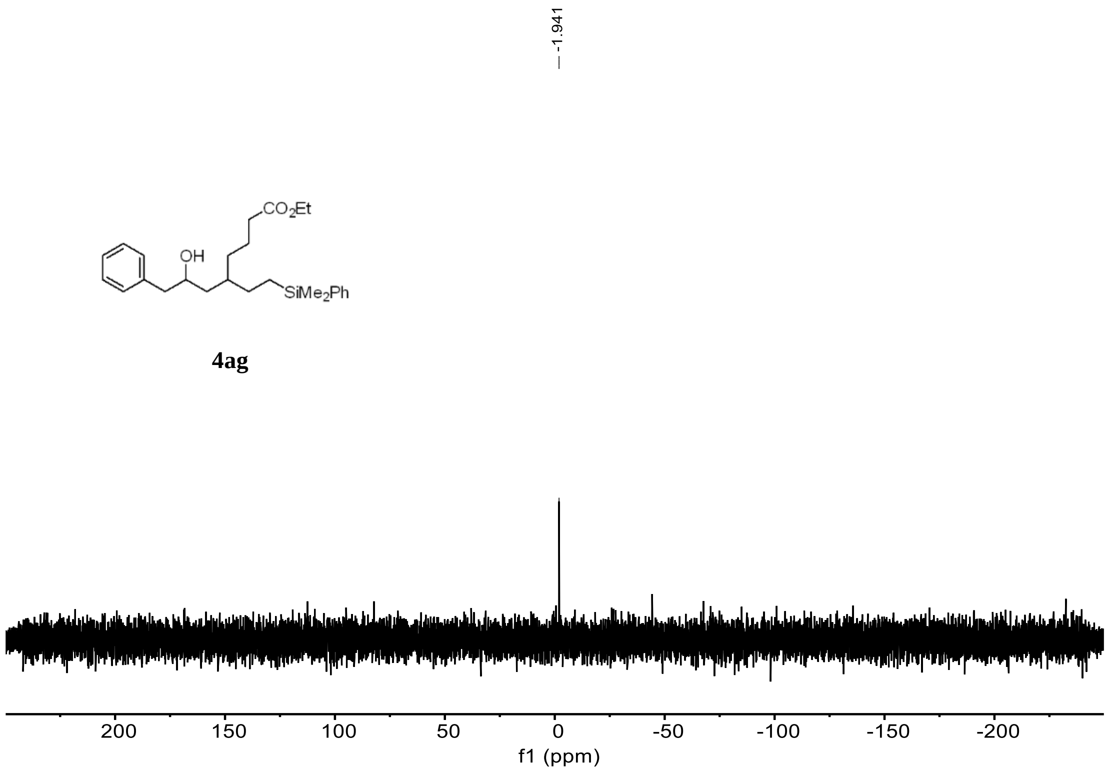

^{29}Si NMR Spectrum of **4ag**

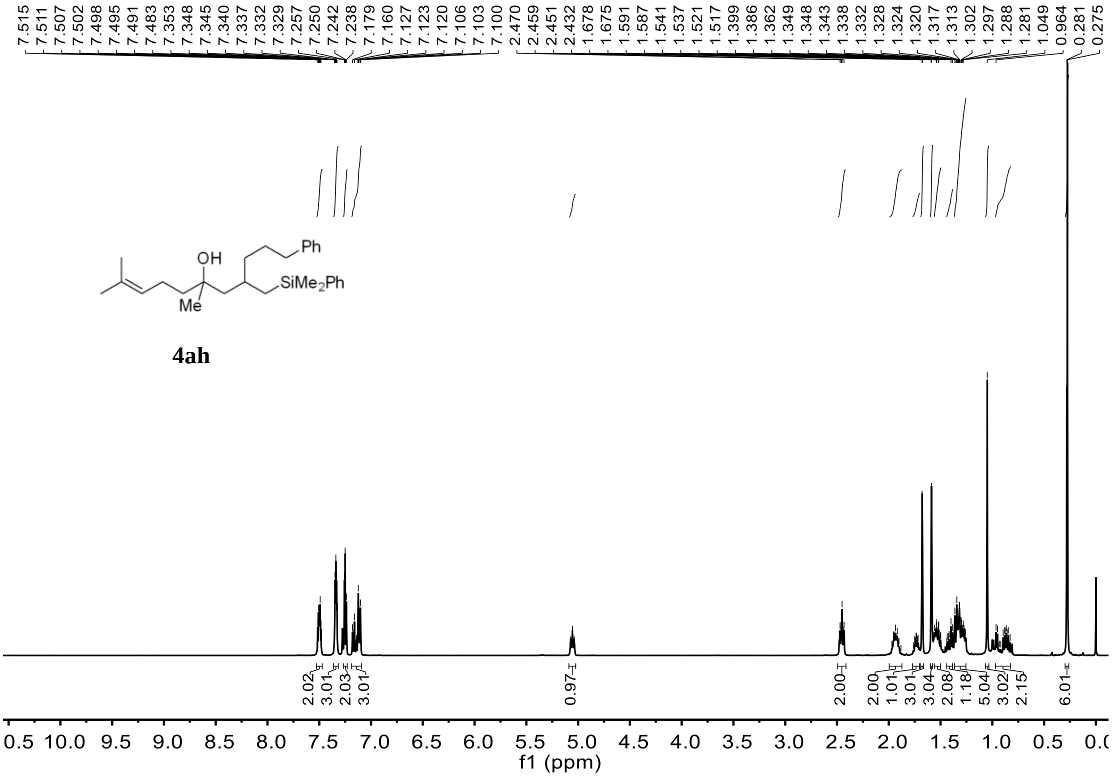

^{1}H NMR Spectrum of **4ah**

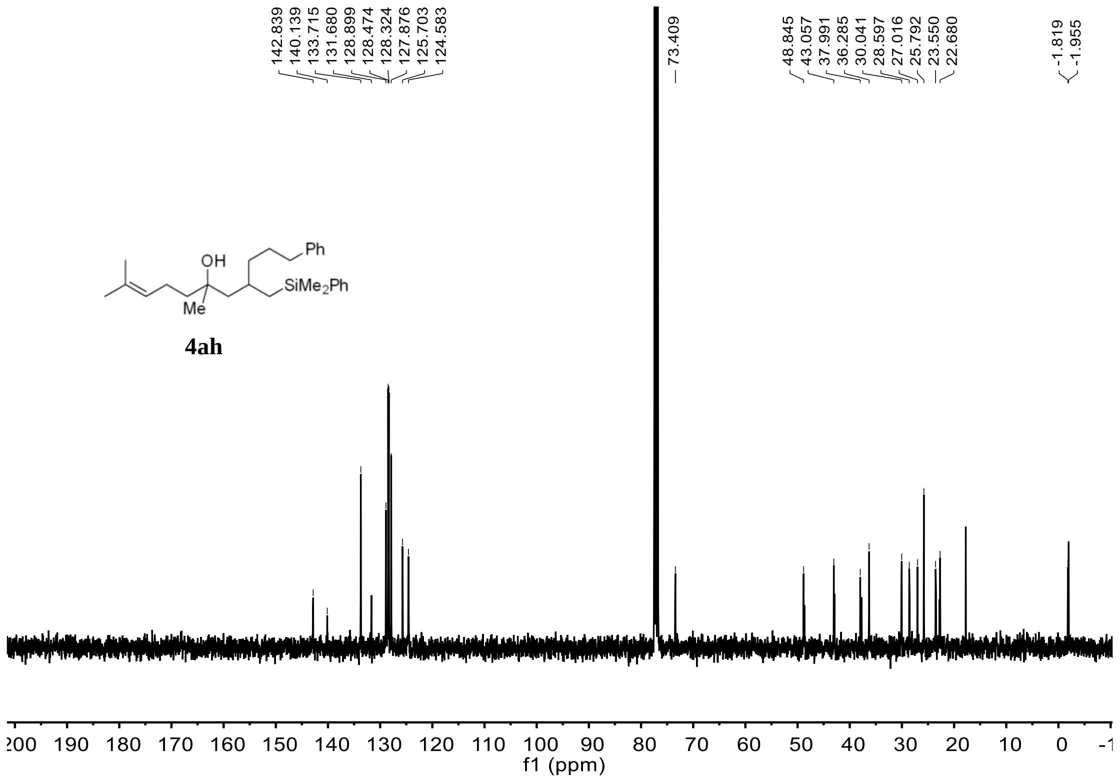

^{13}C NMR Spectrum of **4ah**

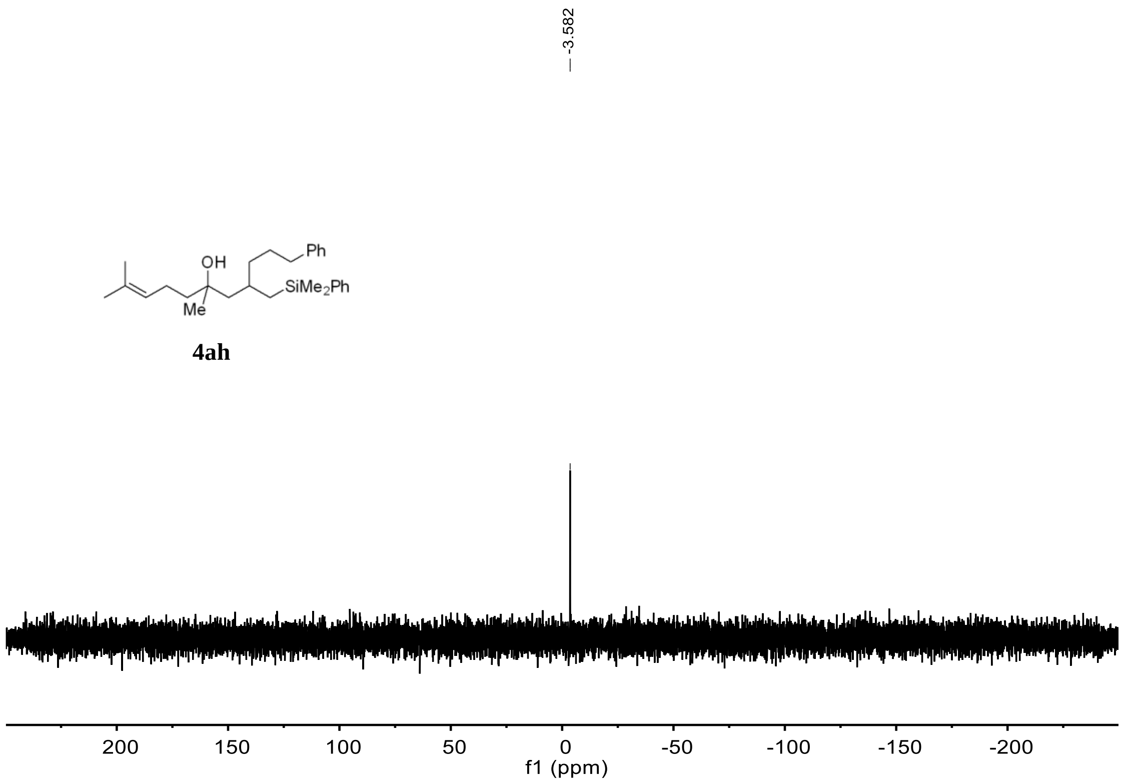

^{29}Si NMR Spectrum of **4ah**

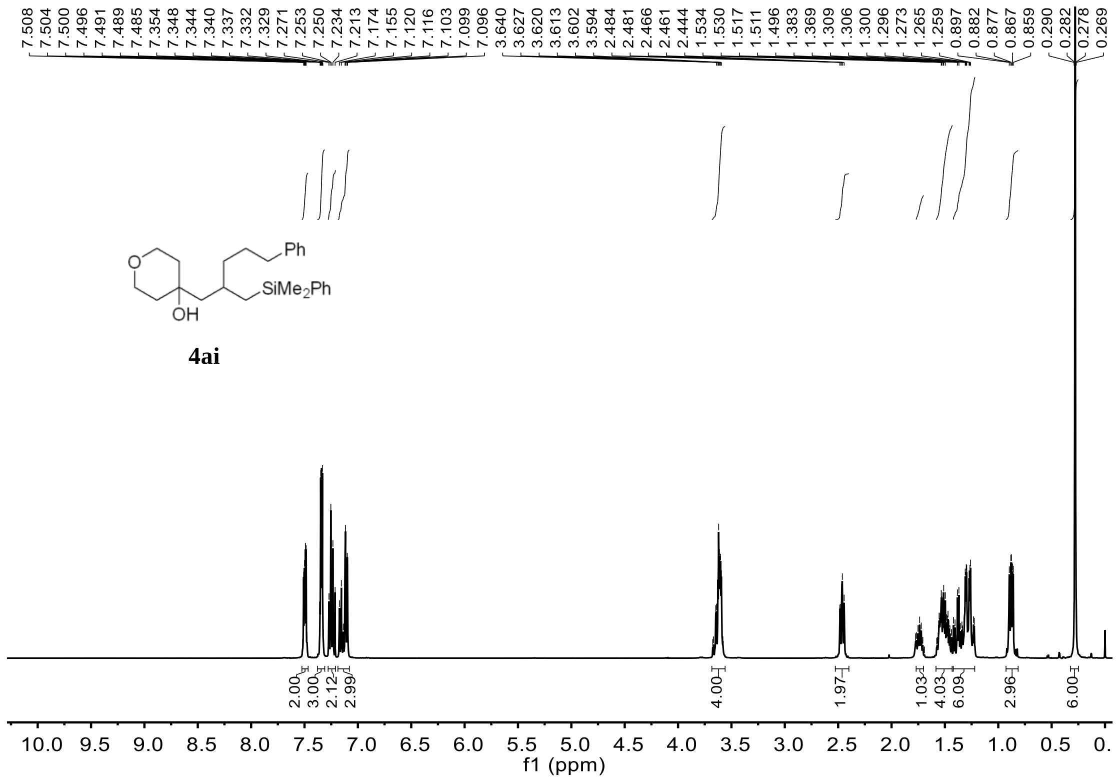

^{1}H NMR Spectrum of **4ai**

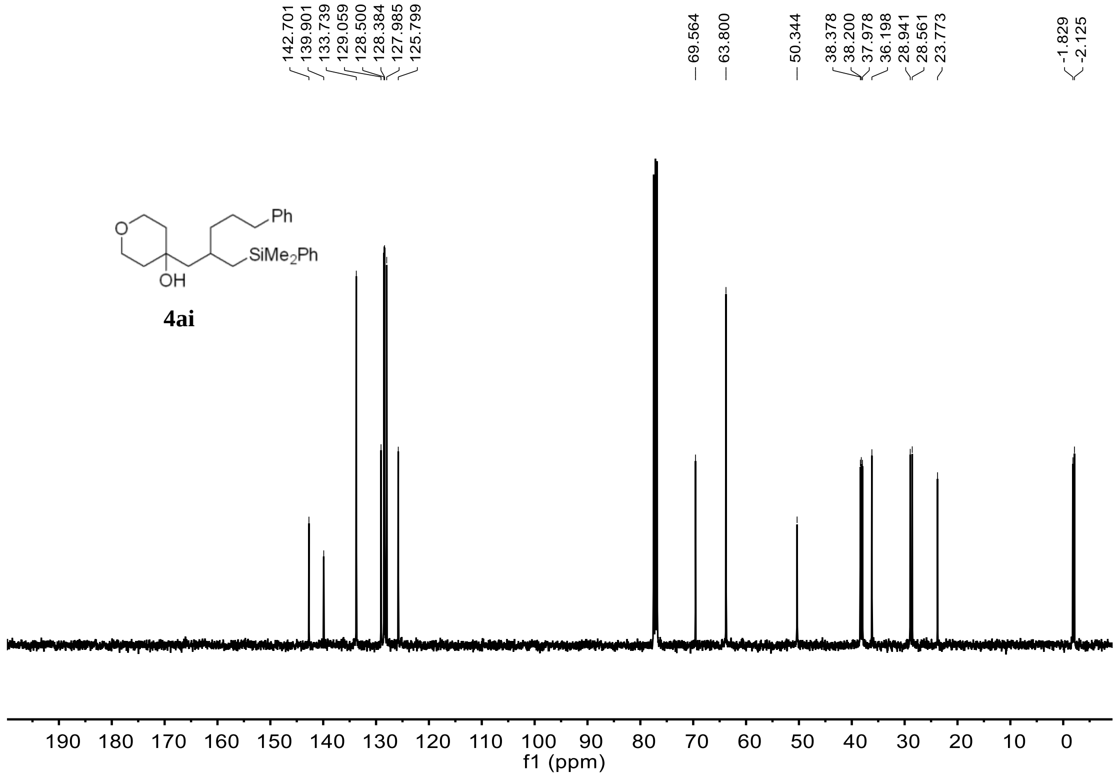

^{13}C NMR Spectrum of **4ai**

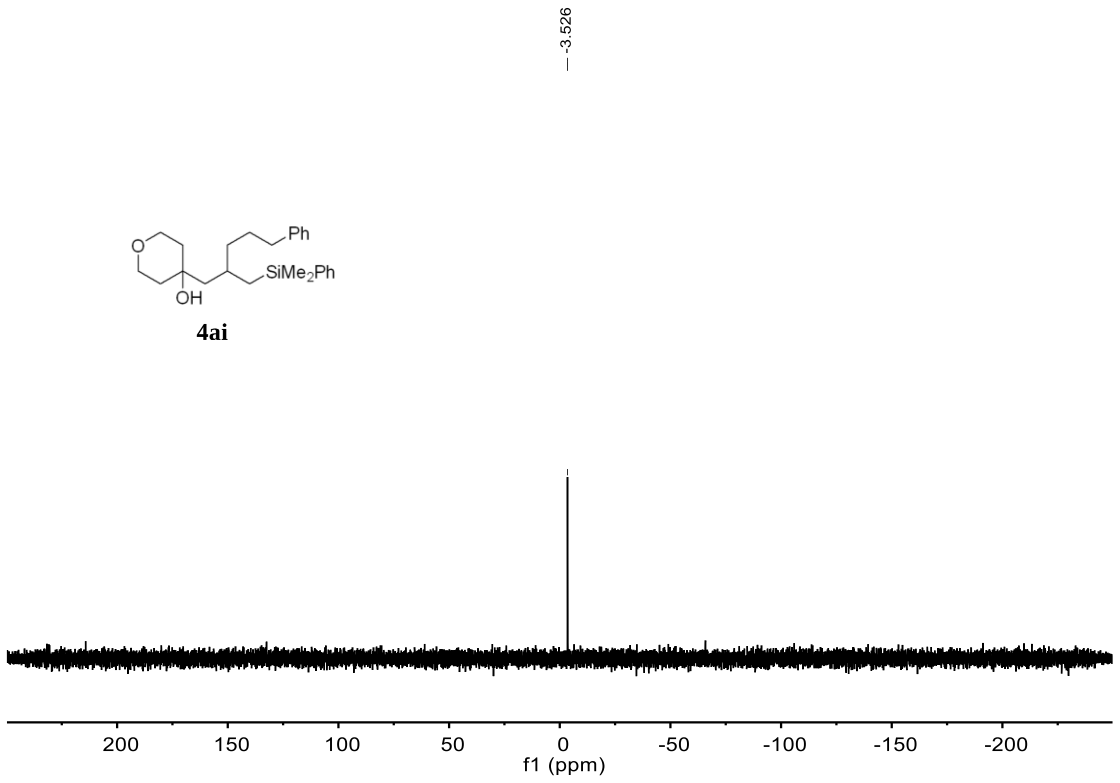

^{29}Si NMR Spectrum of **4ai**

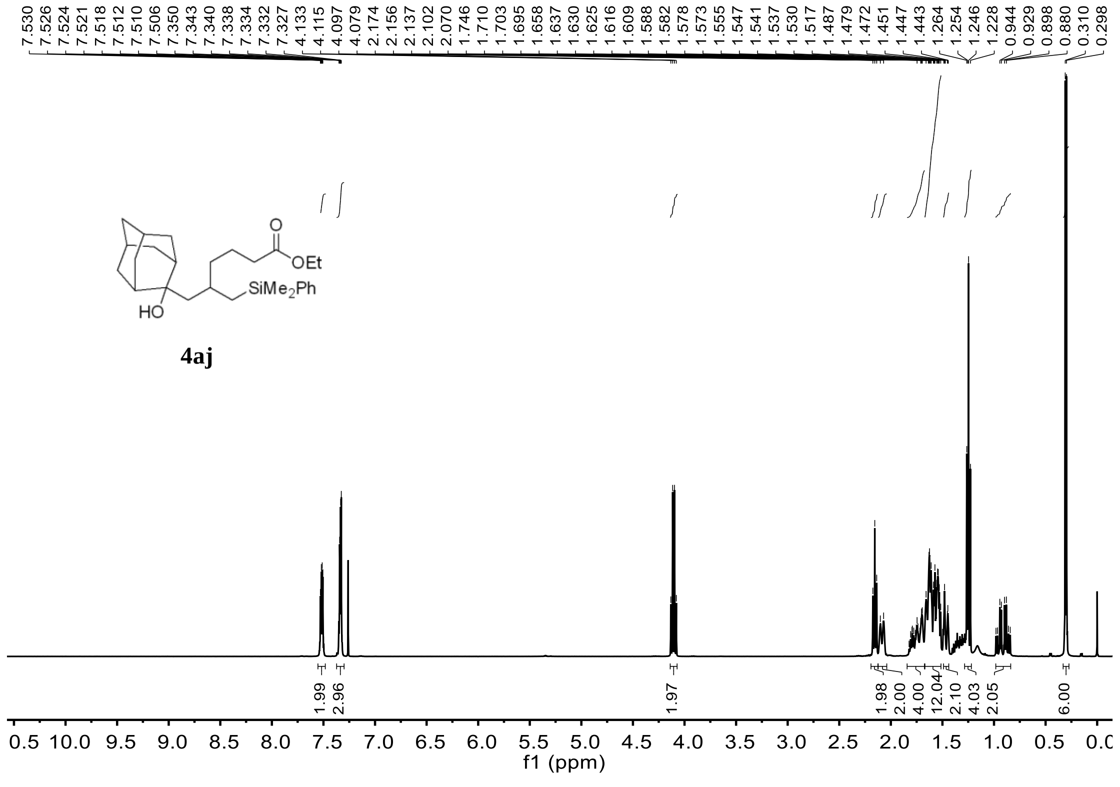

^{1}H NMR Spectrum of **4aj**

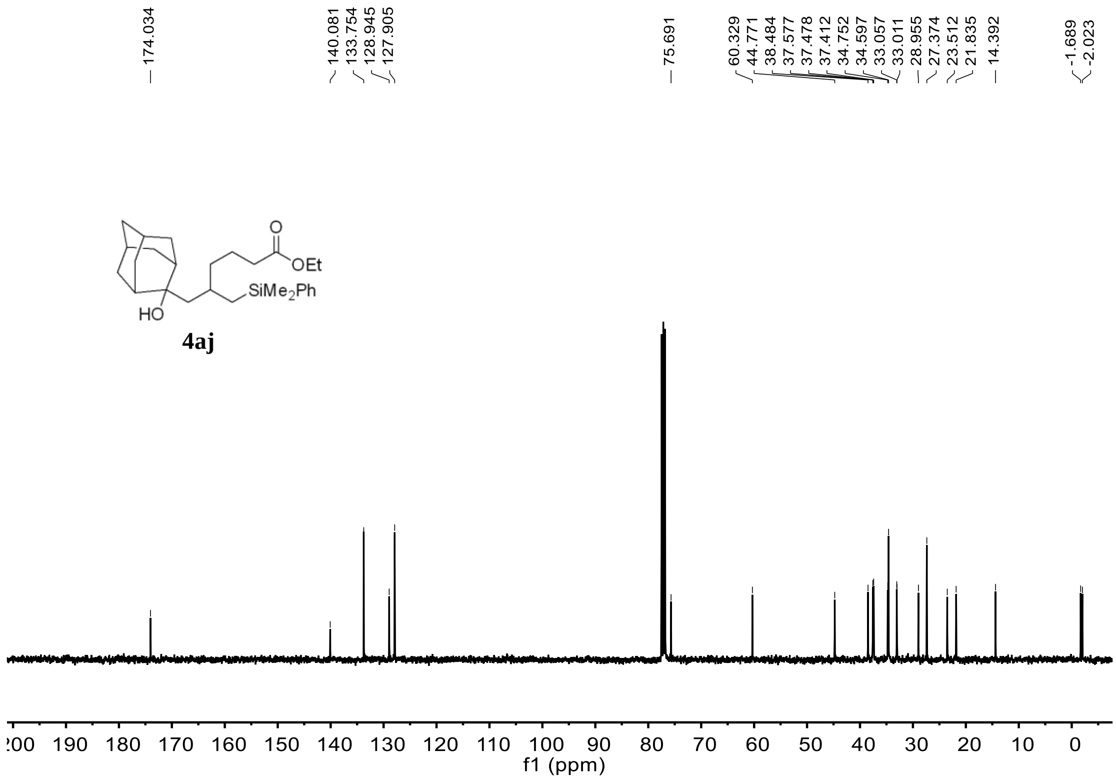

^{13}C NMR Spectrum of **4aj**

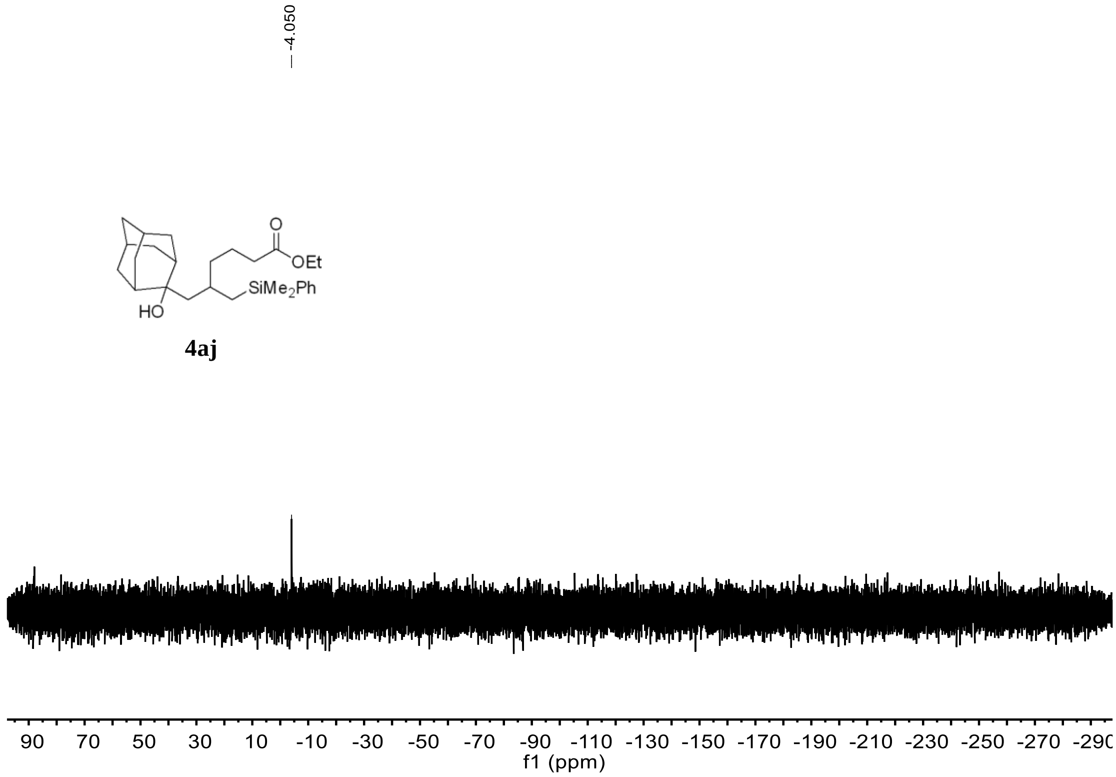

^{29}Si NMR Spectrum of **4aj**

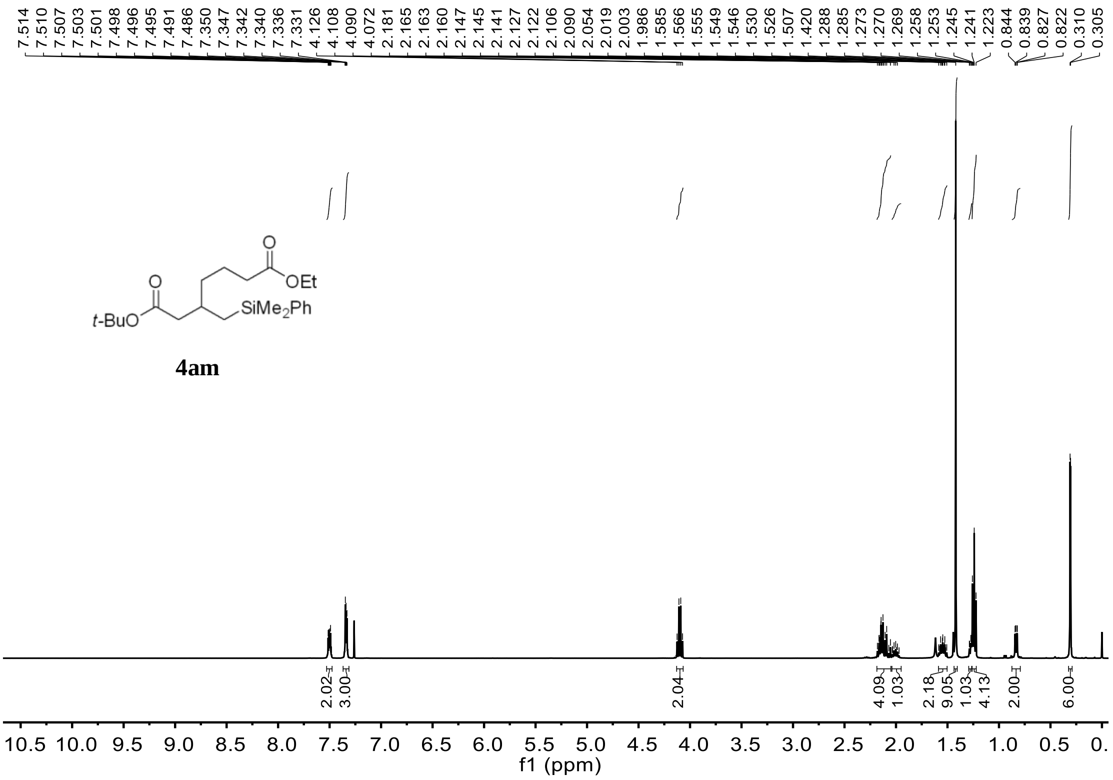

^{1}H NMR Spectrum of **4am**

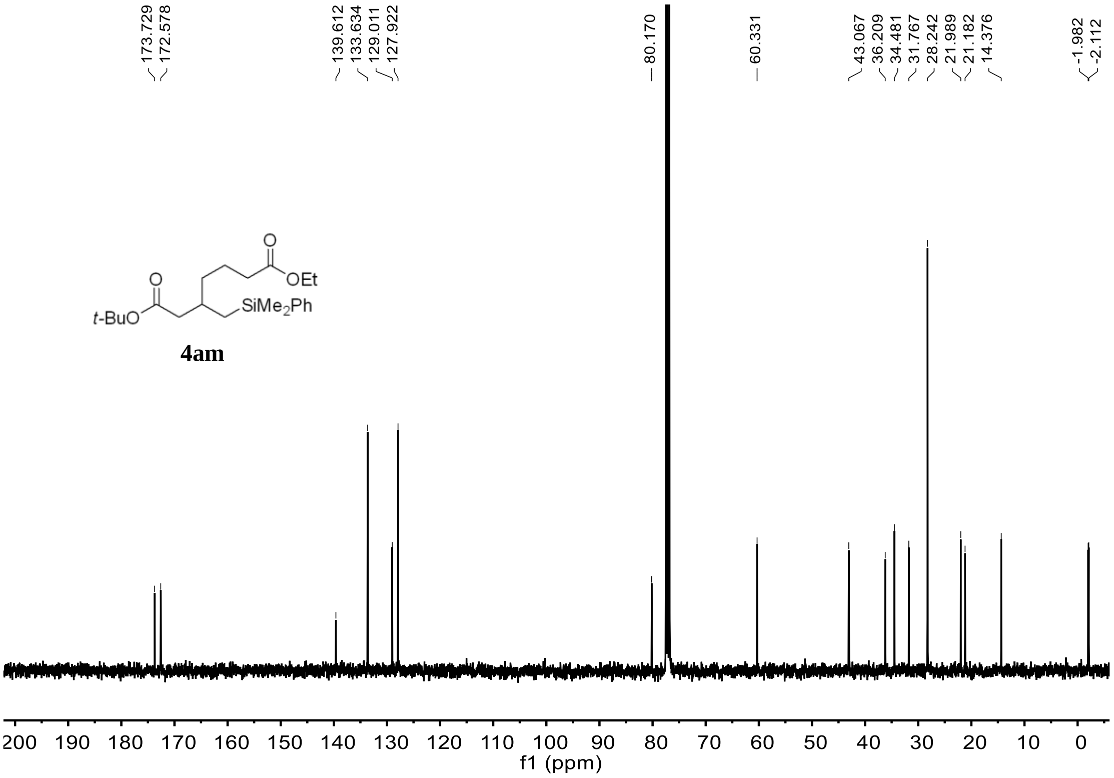

^{13}C NMR Spectrum of **4am**

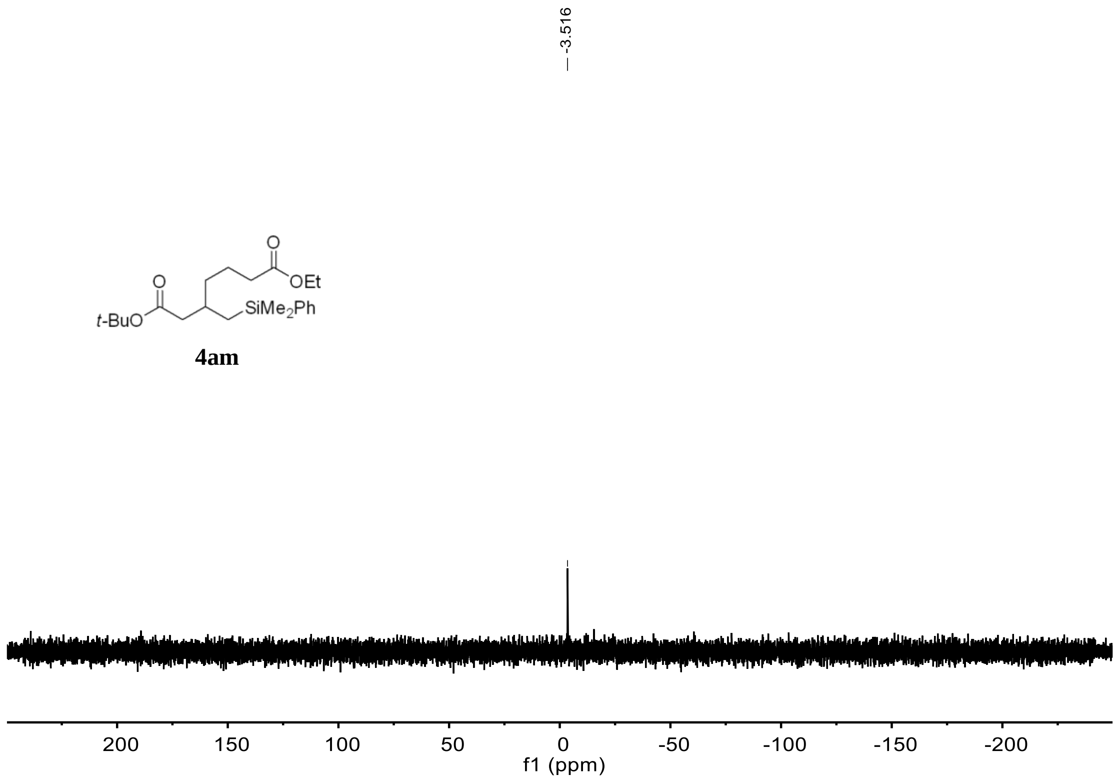

^{29}Si NMR Spectrum of **4am**

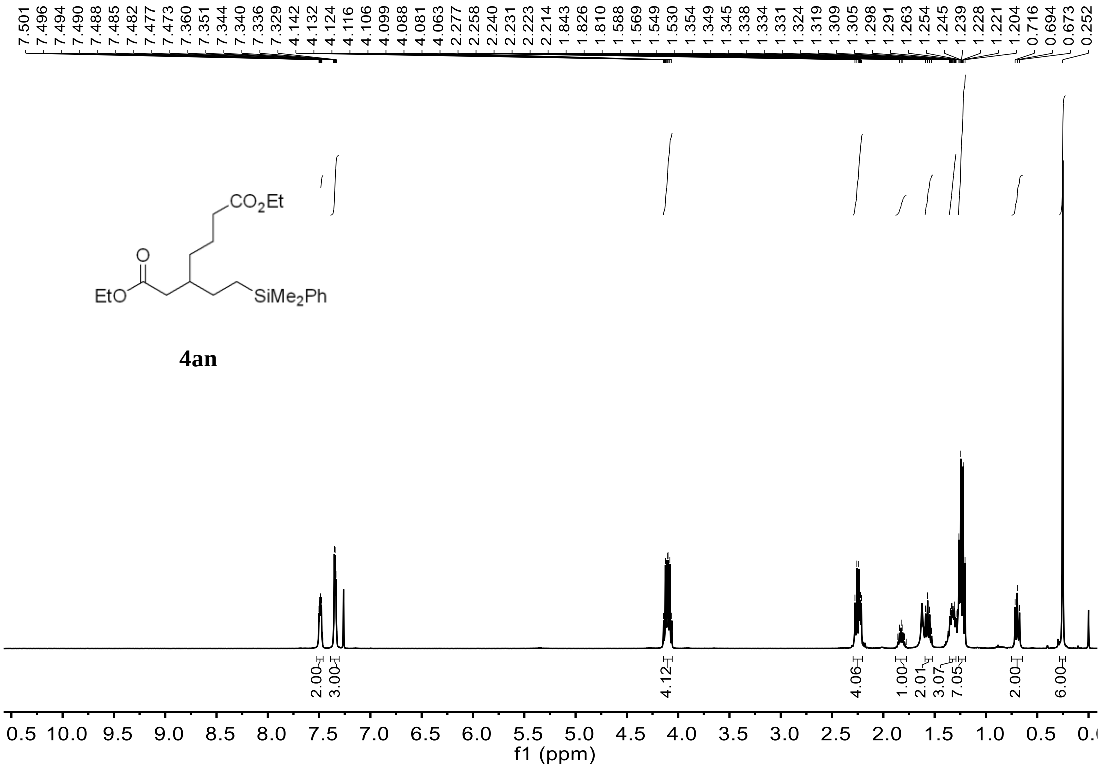

^{1}H NMR Spectrum of **4an**

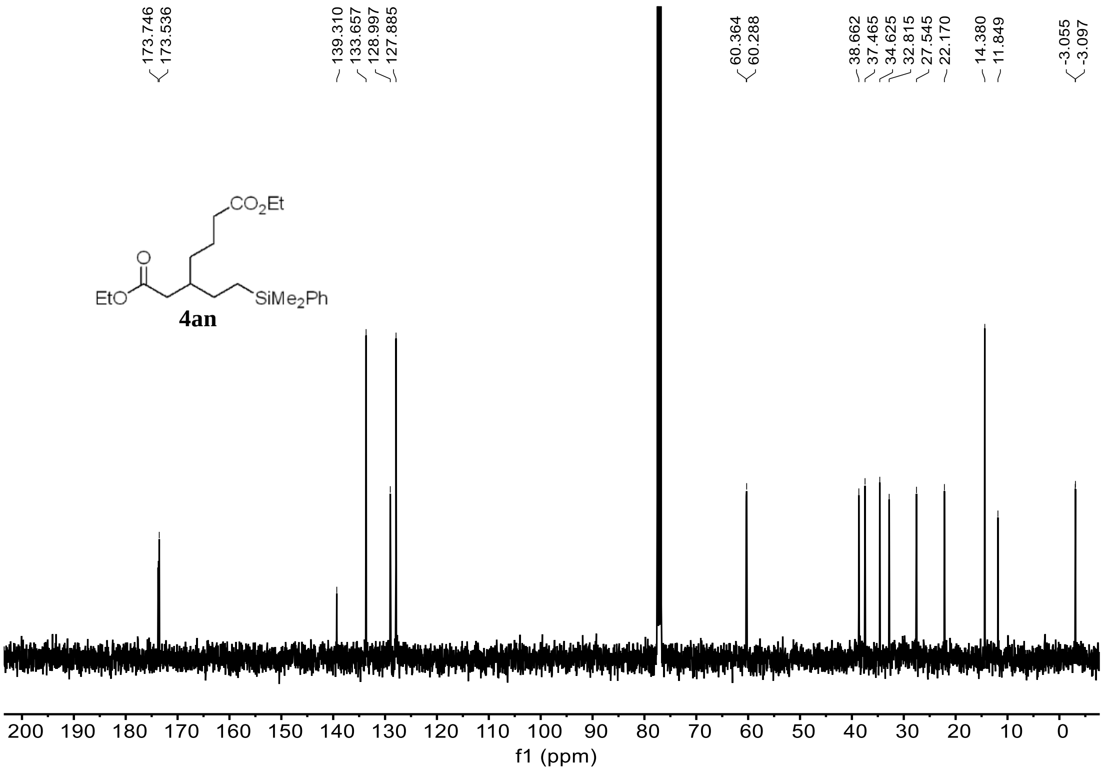

^{13}C NMR Spectrum of **4an**

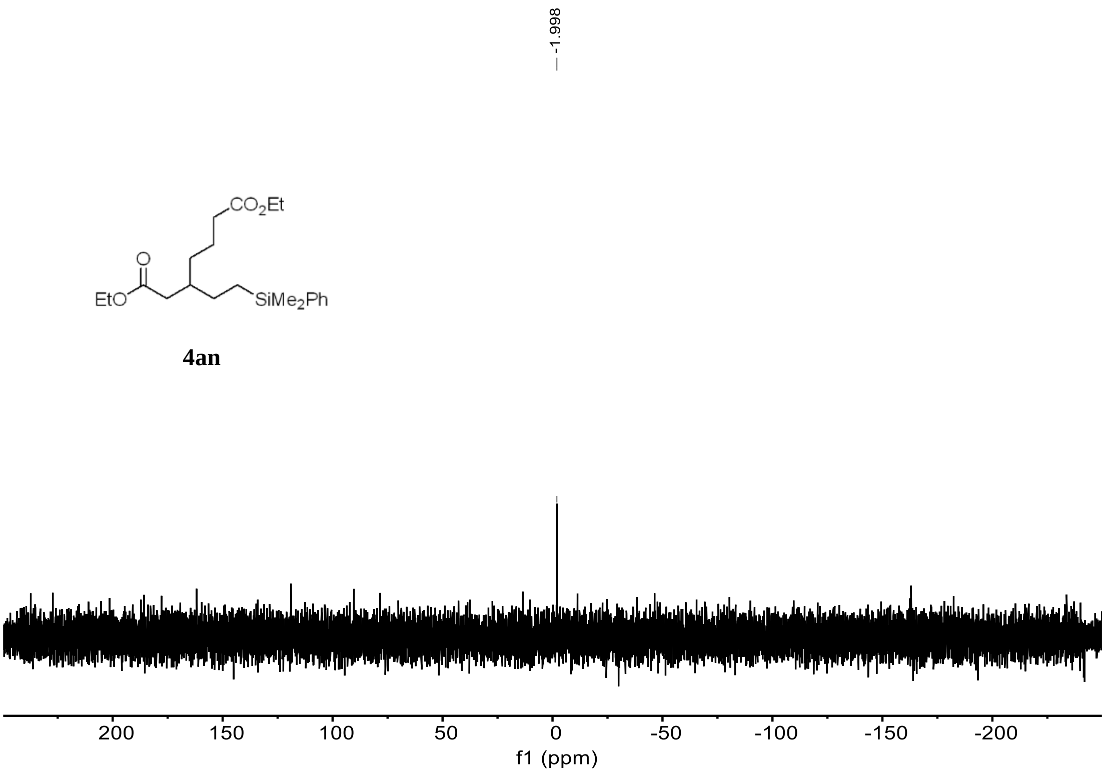

^{29}Si NMR Spectrum of **4an**

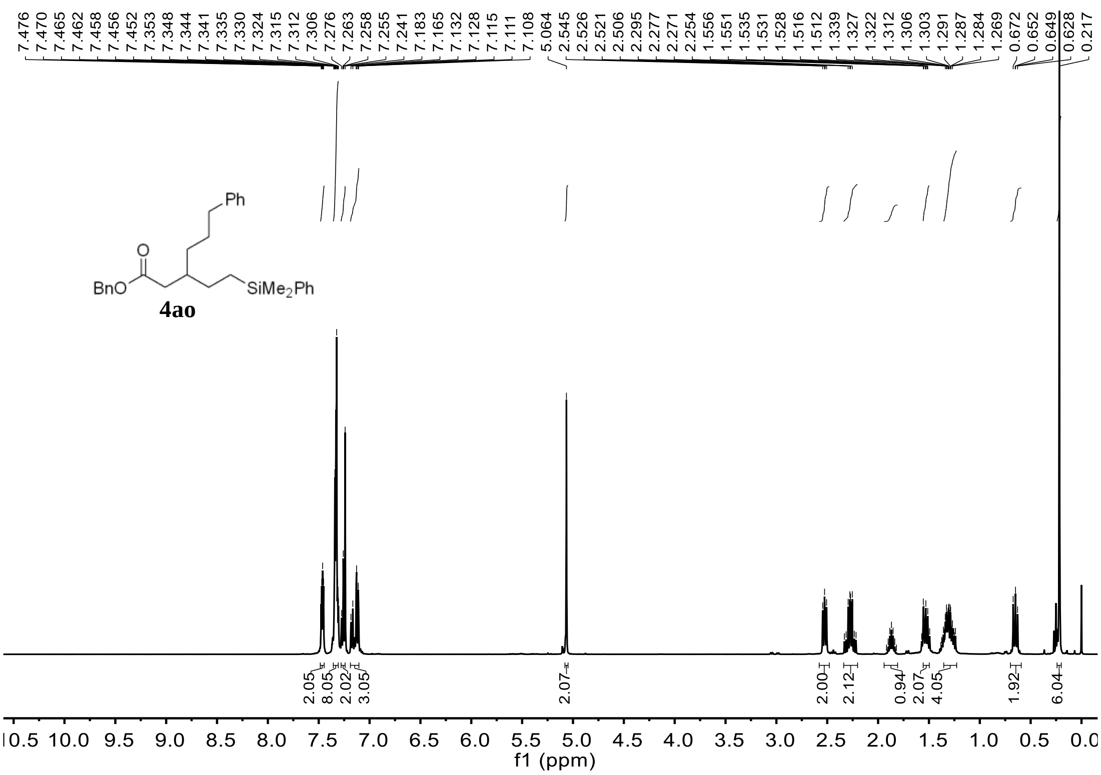

^{1}H NMR Spectrum of **4ao**

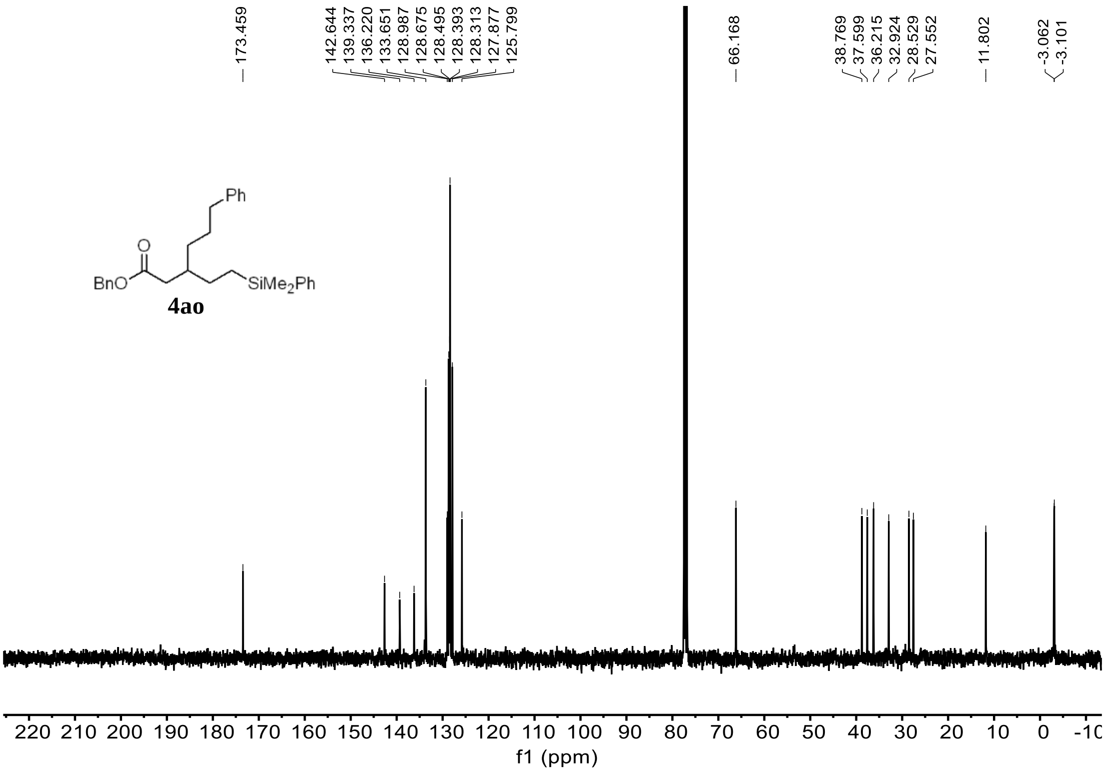

^{13}C NMR Spectrum of **4ao**

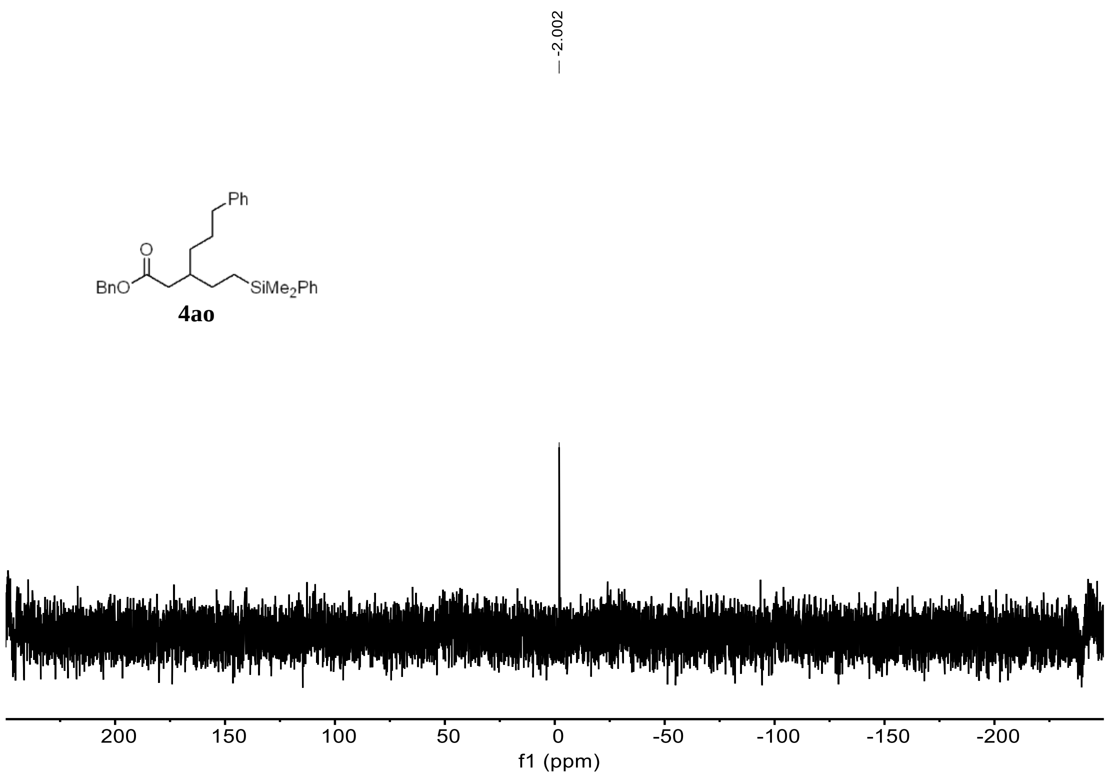

^{29}Si NMR Spectrum of **4ao**

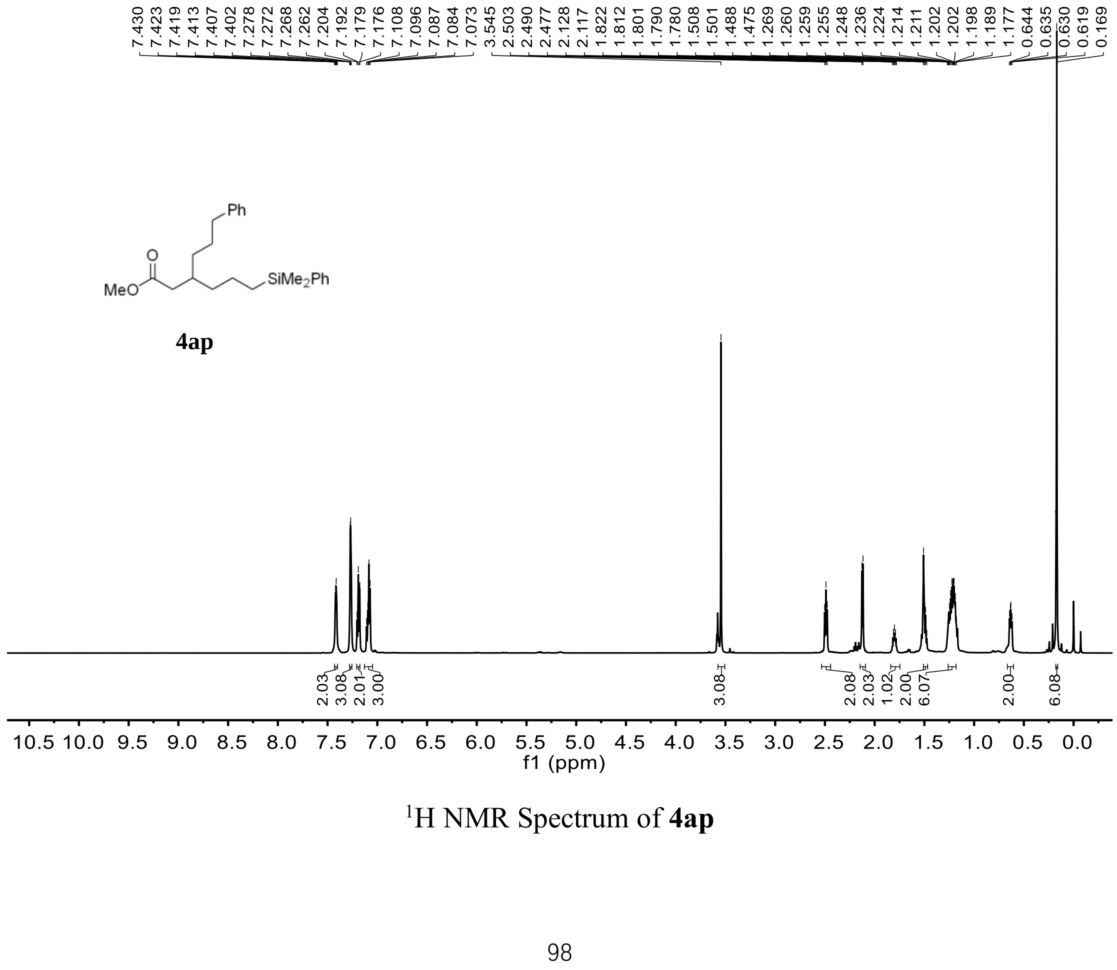

^{1}H NMR Spectrum of **4ap**

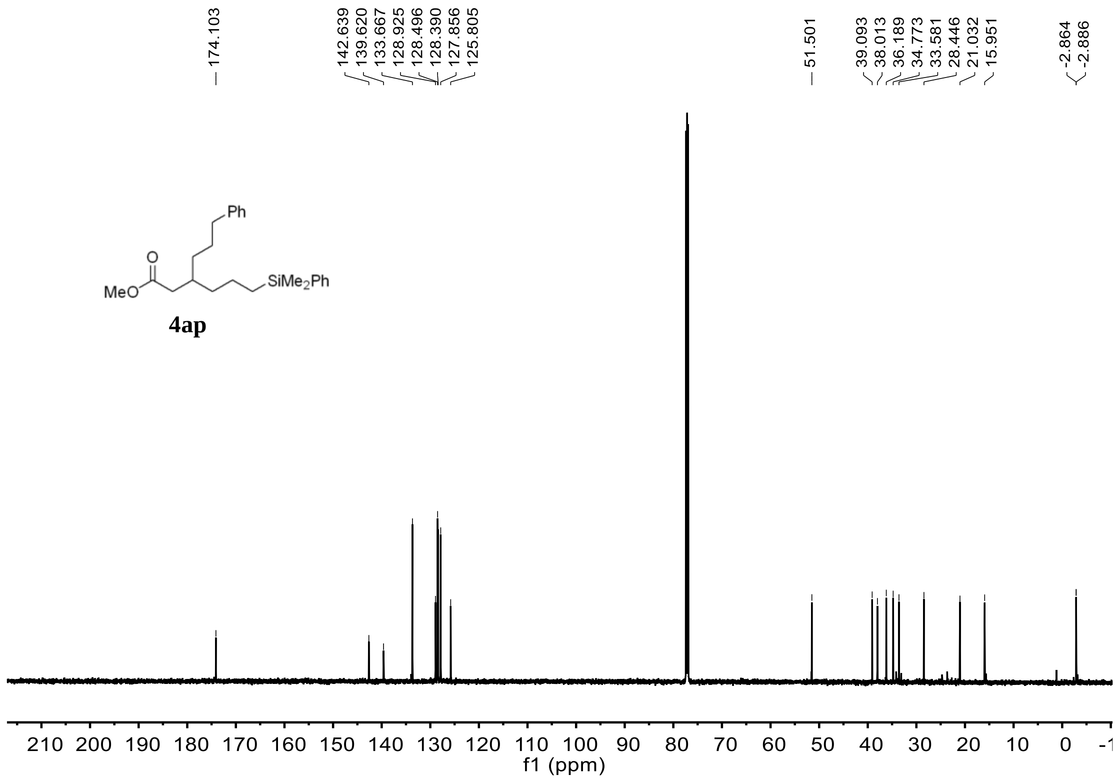

^{13}C NMR Spectrum of **4ap**

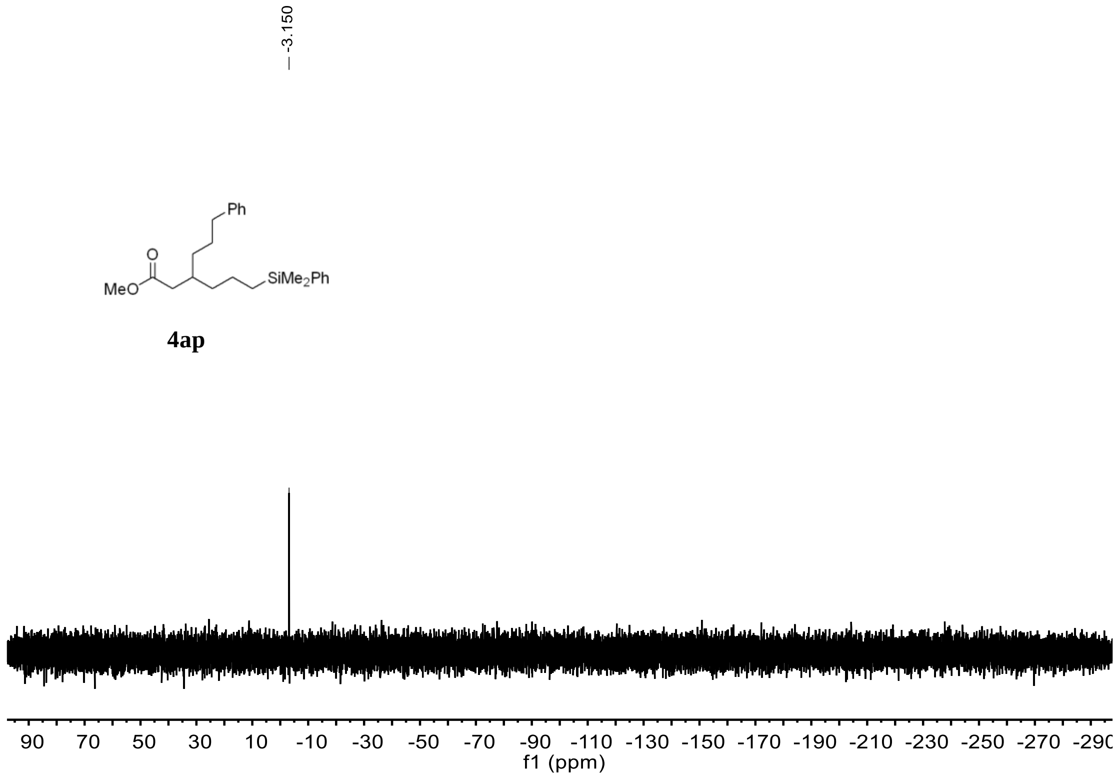

^{29}Si NMR Spectrum of **4ap**

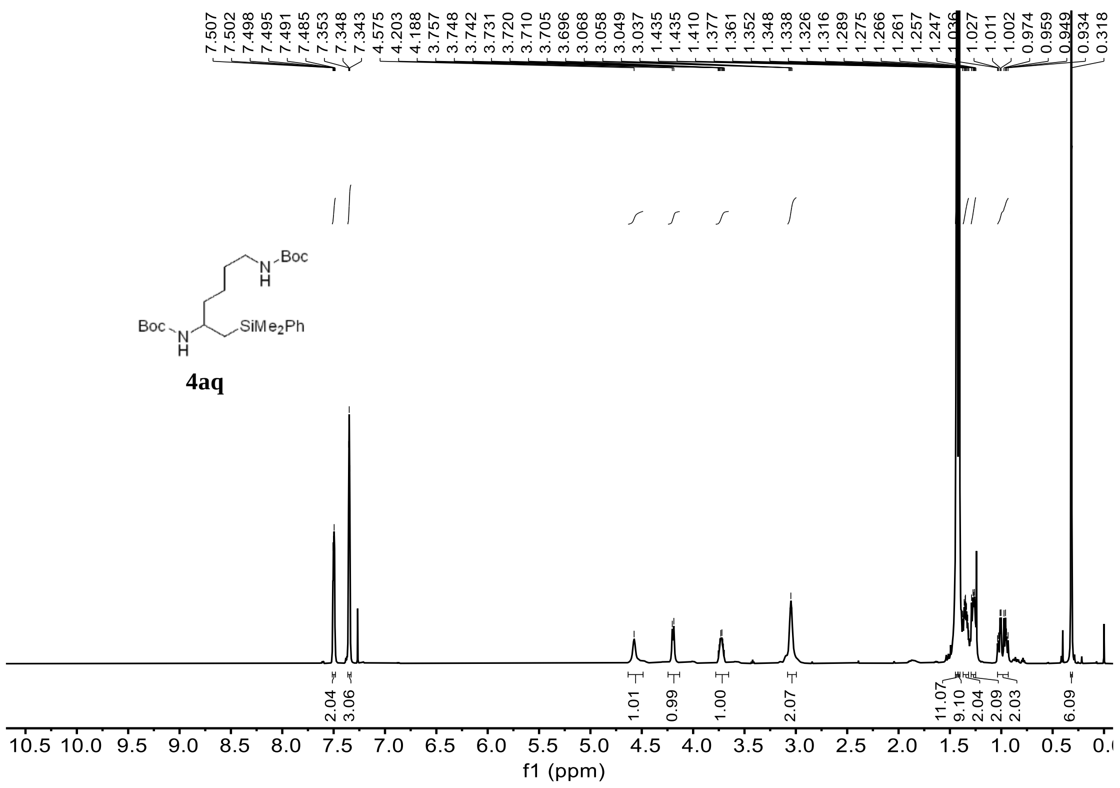

^{1}H NMR Spectrum of **4aq**

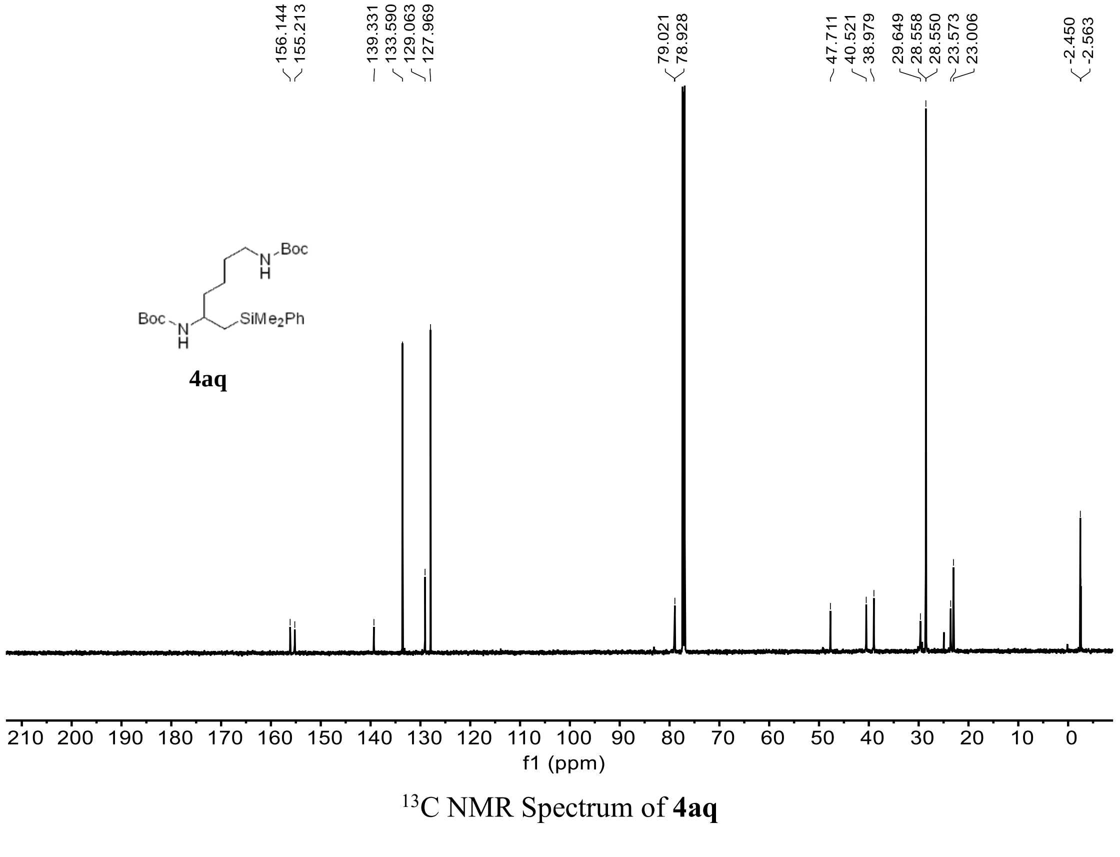

^{13}C NMR Spectrum of **4aq**

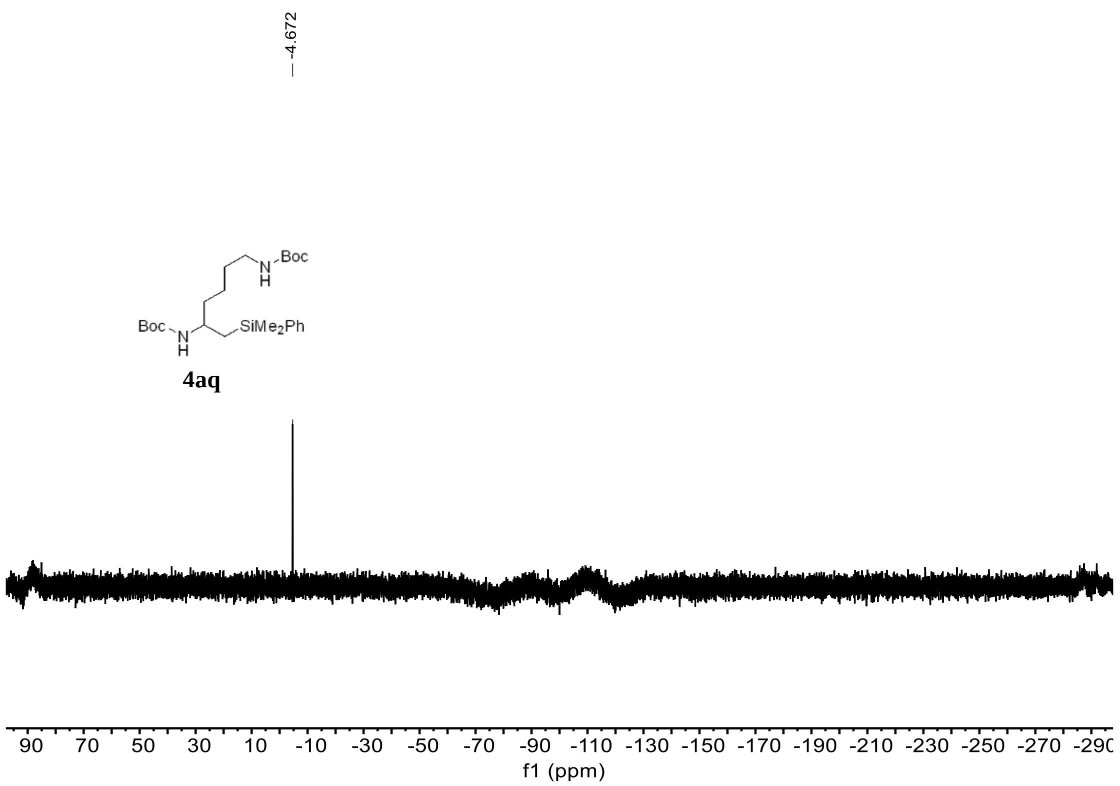

^{29}Si NMR Spectrum of **4aq**

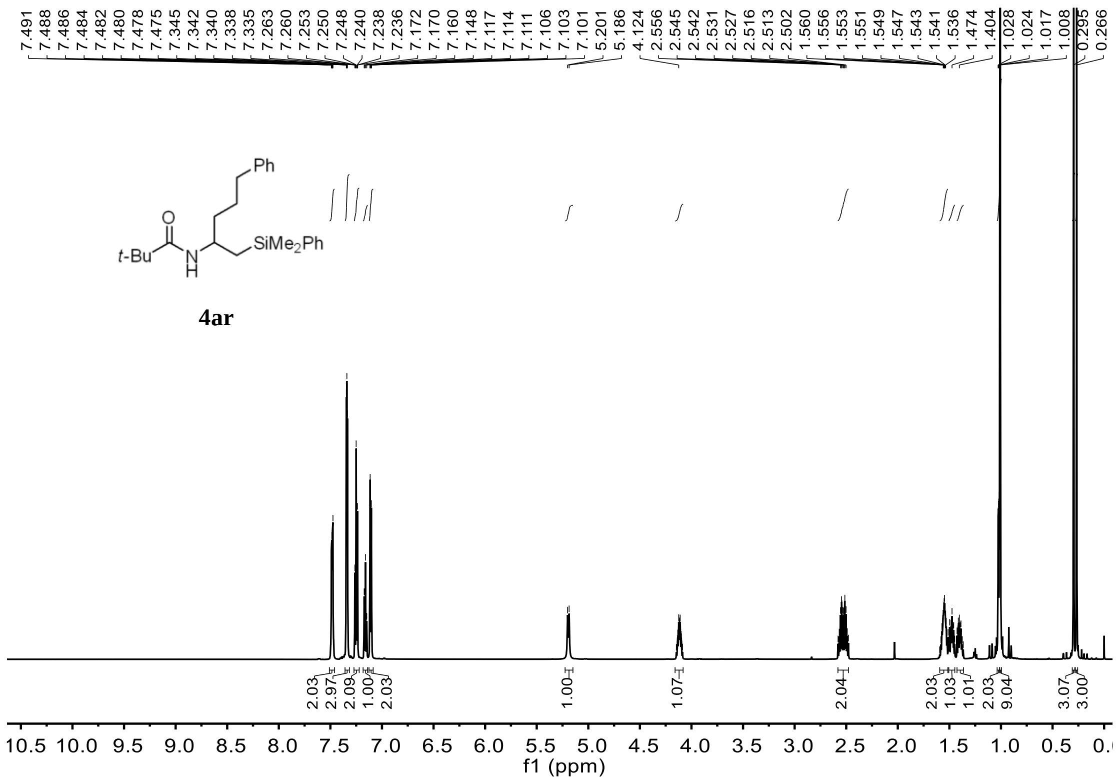

^{1}H NMR Spectrum of **4ar**

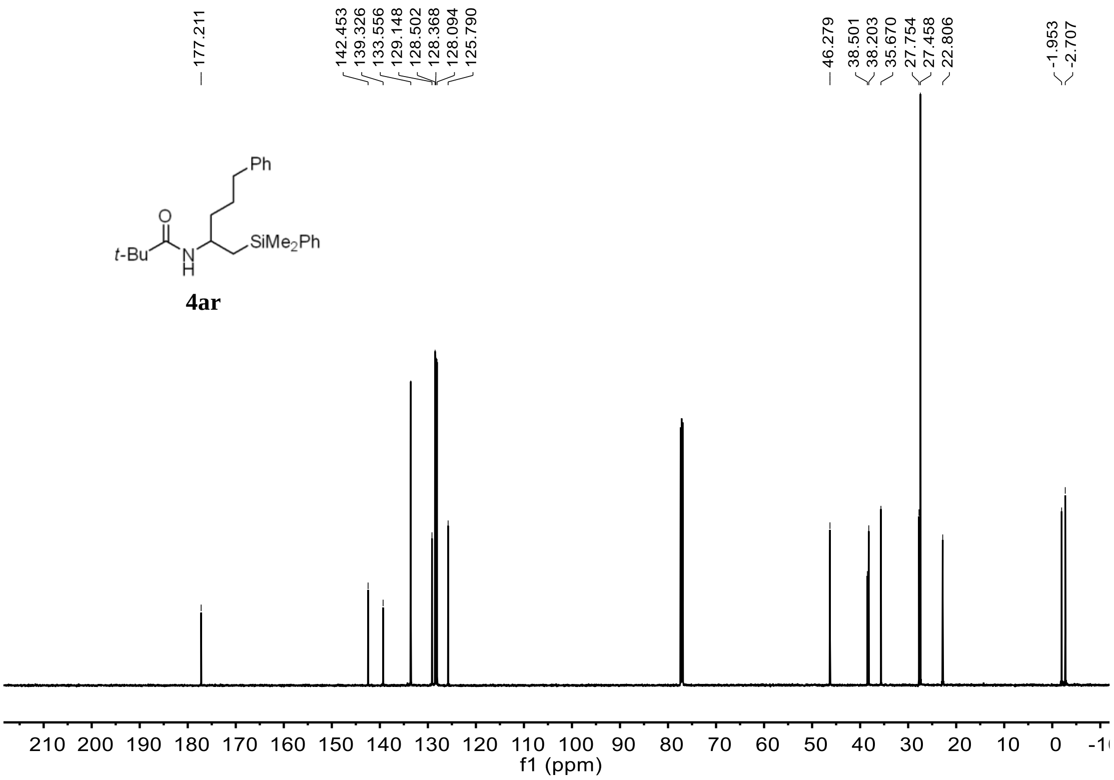

^{13}C NMR Spectrum of **4ar**

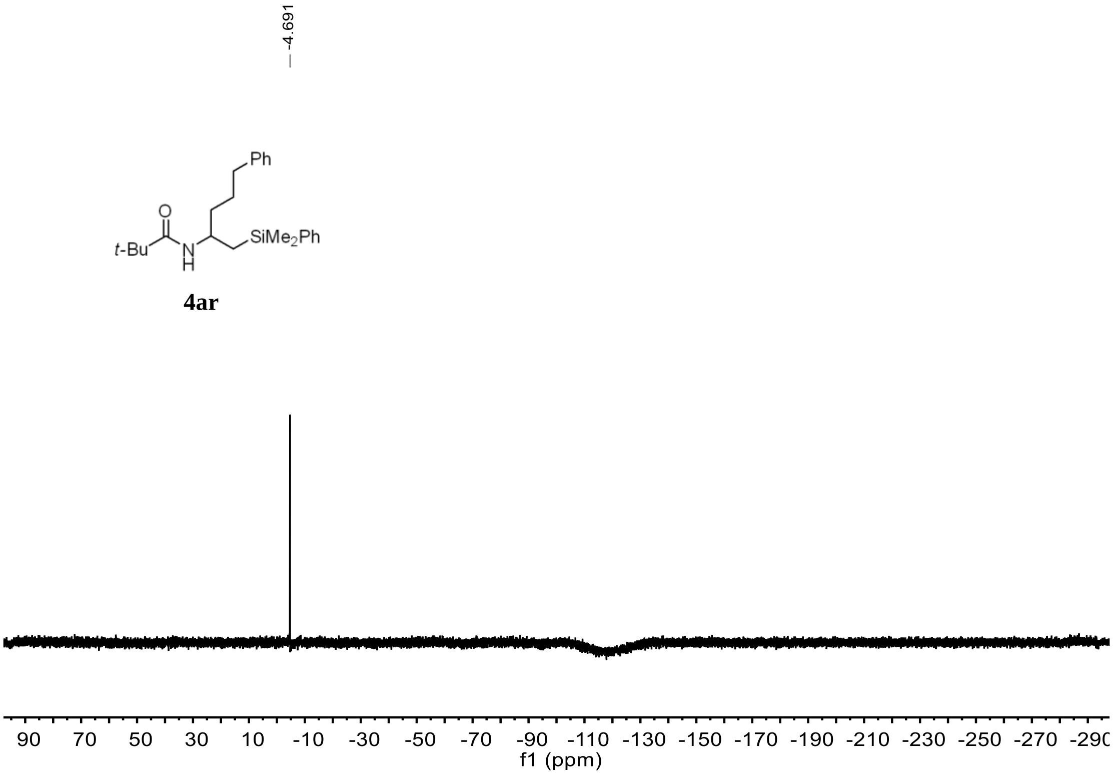

^{29}Si NMR Spectrum of **4ar**

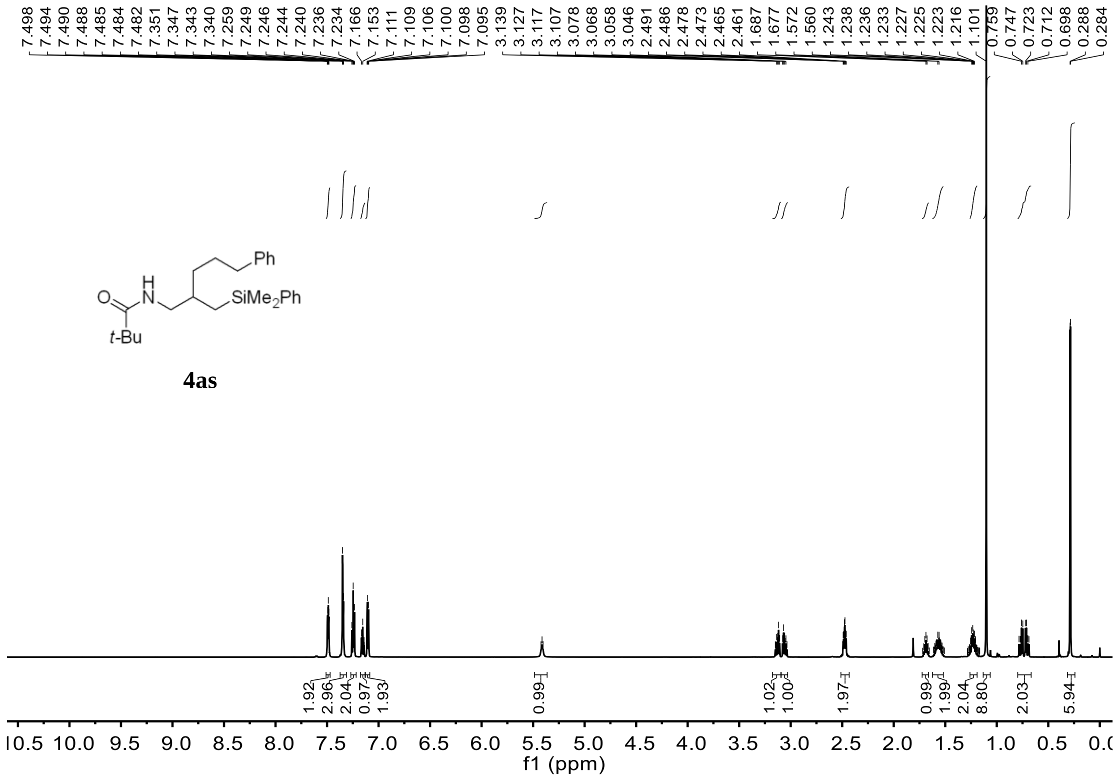

^{1}H NMR Spectrum of **4as**

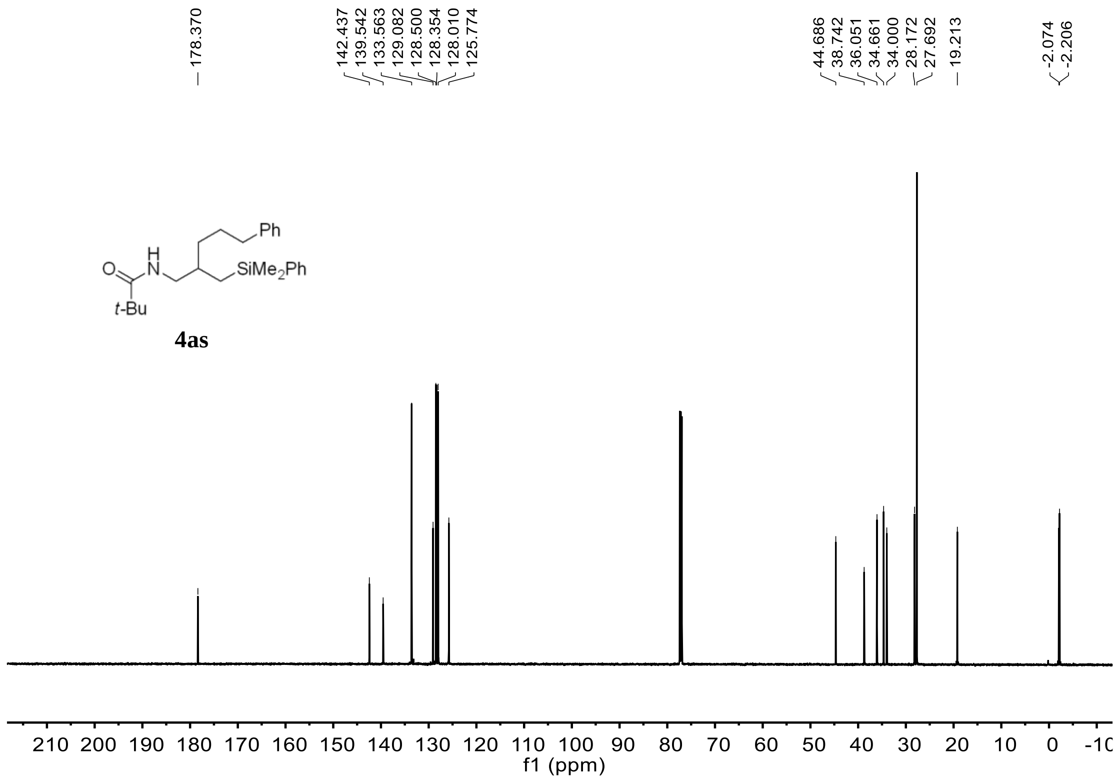

^{13}C NMR Spectrum of **4as**

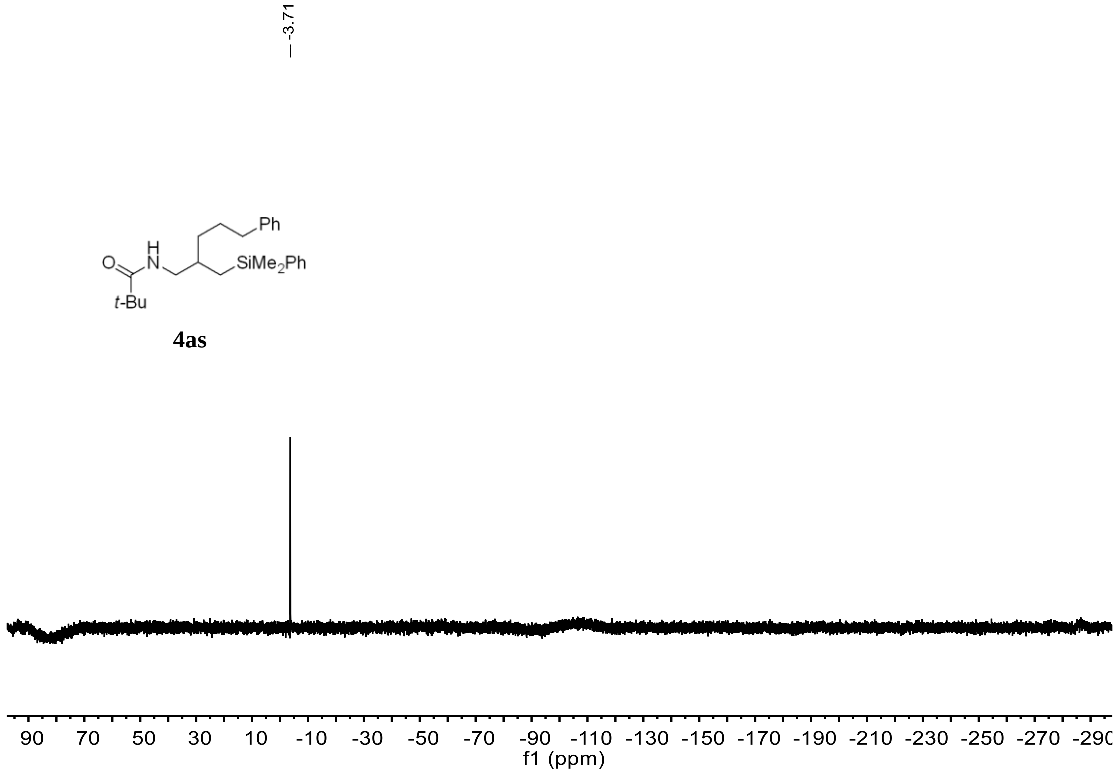

^{29}Si NMR Spectrum of **4as**

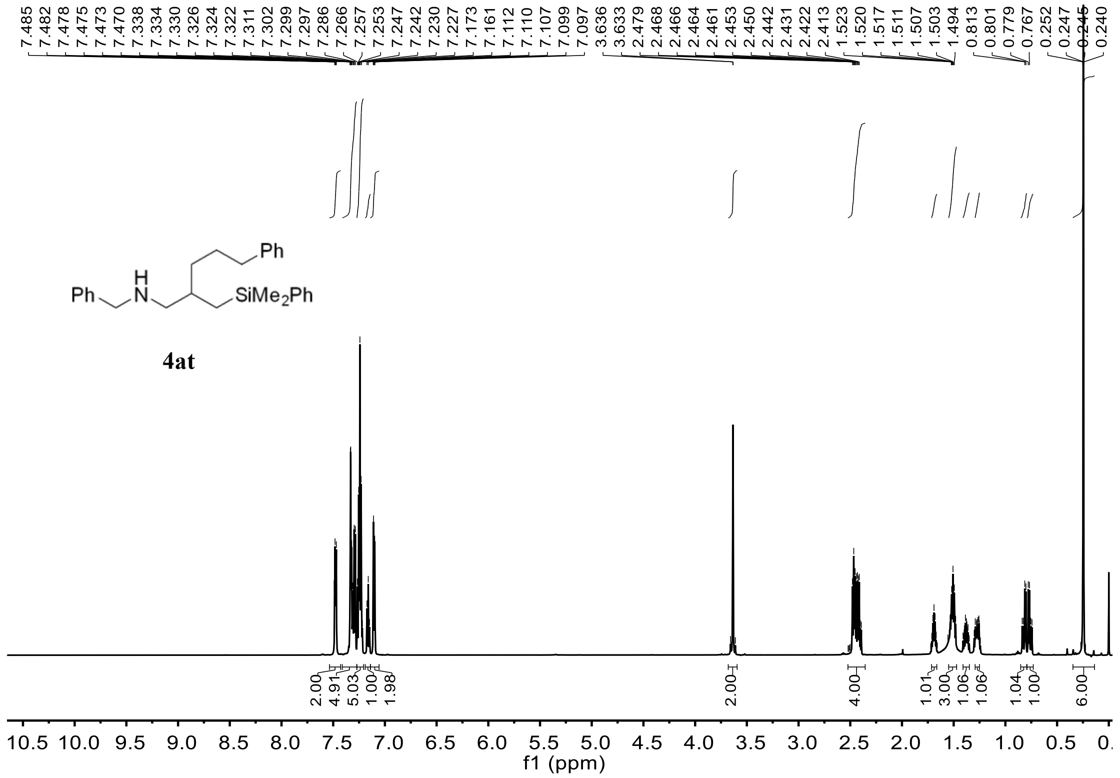

^{1}H NMR Spectrum of **4at**

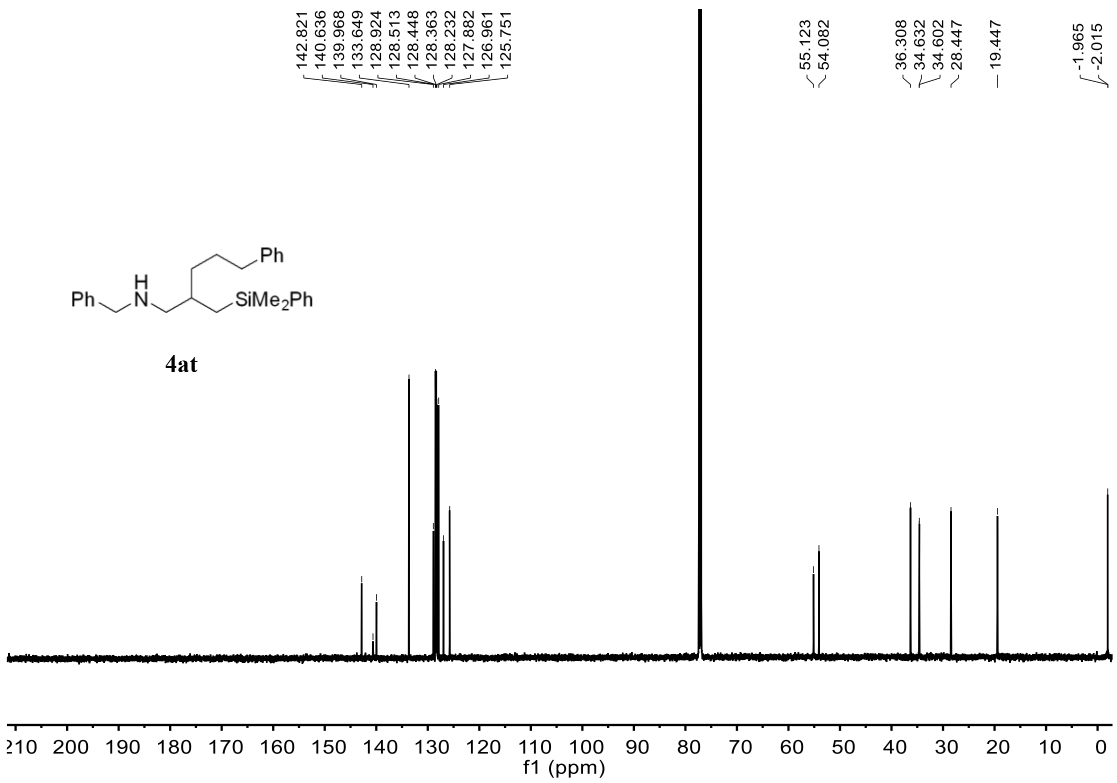

^{13}C NMR Spectrum of **4at**

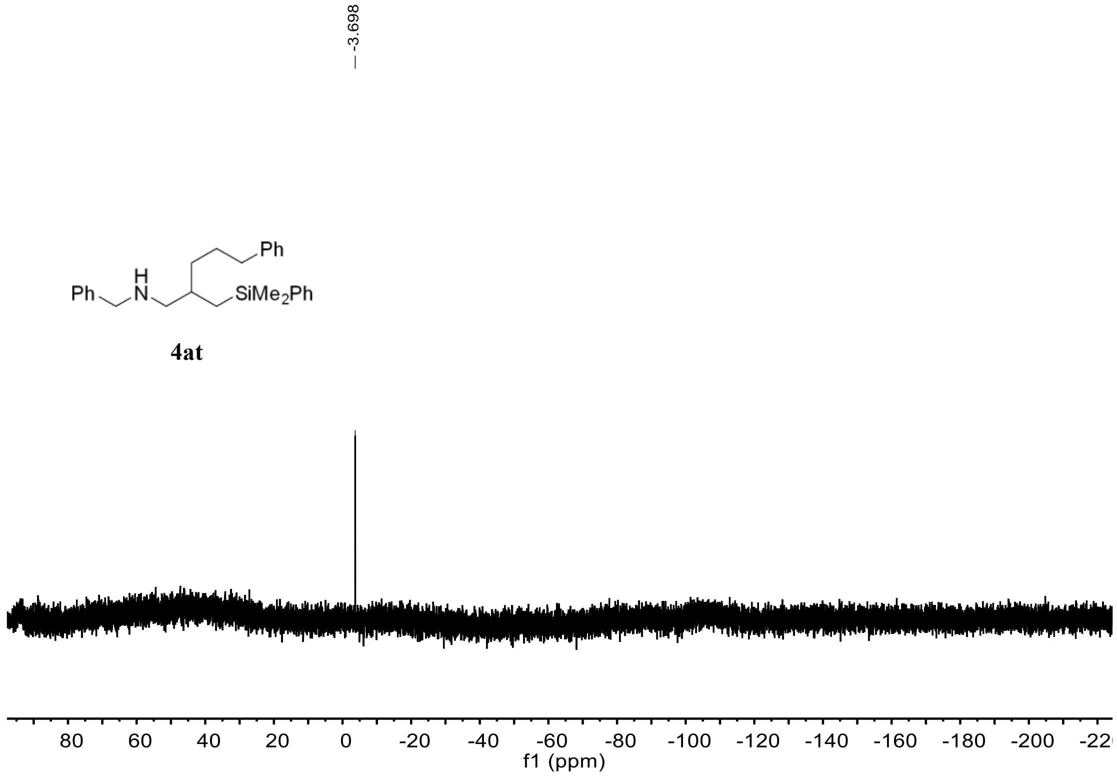

^{29}Si NMR Spectrum of **4at**

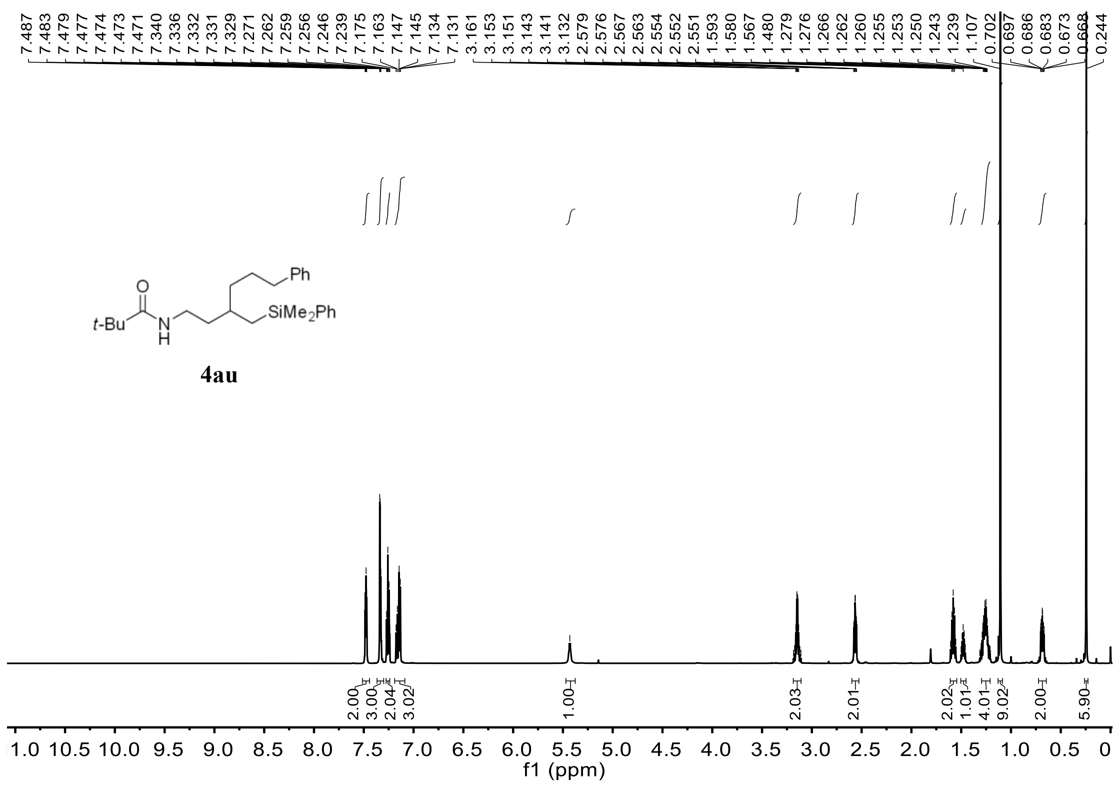

^{1}H NMR Spectrum of **4au**

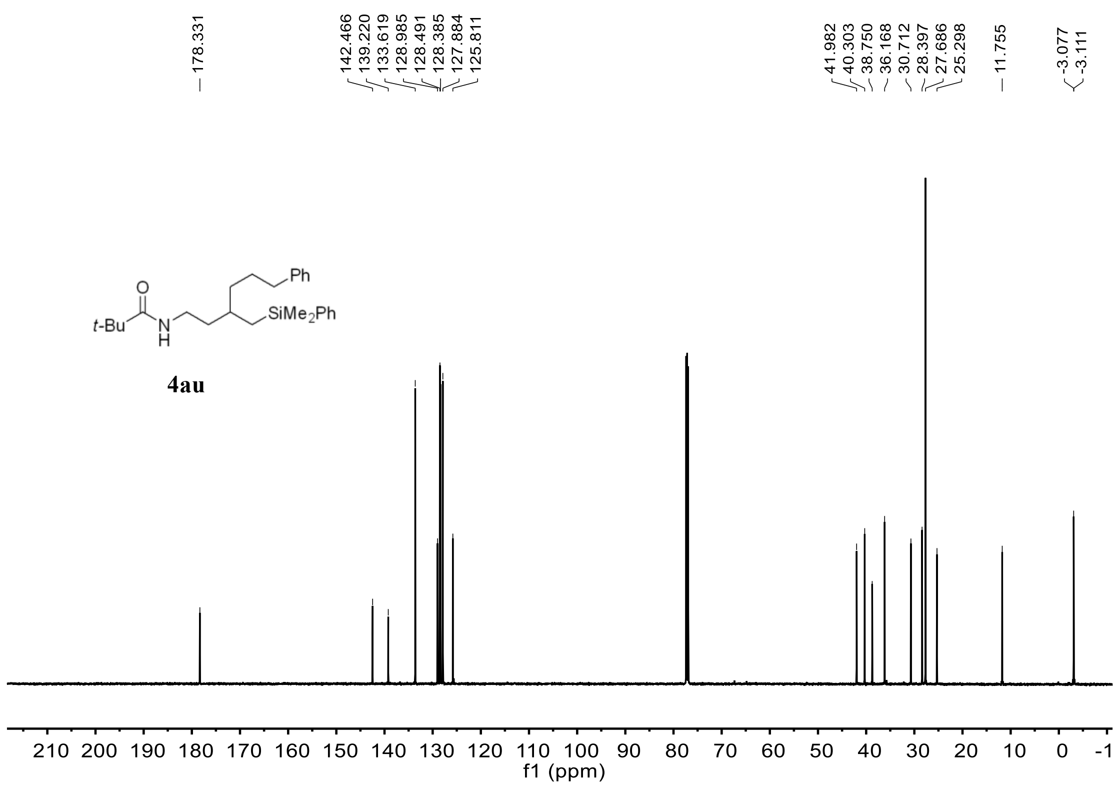

^{13}C NMR Spectrum of **4au**

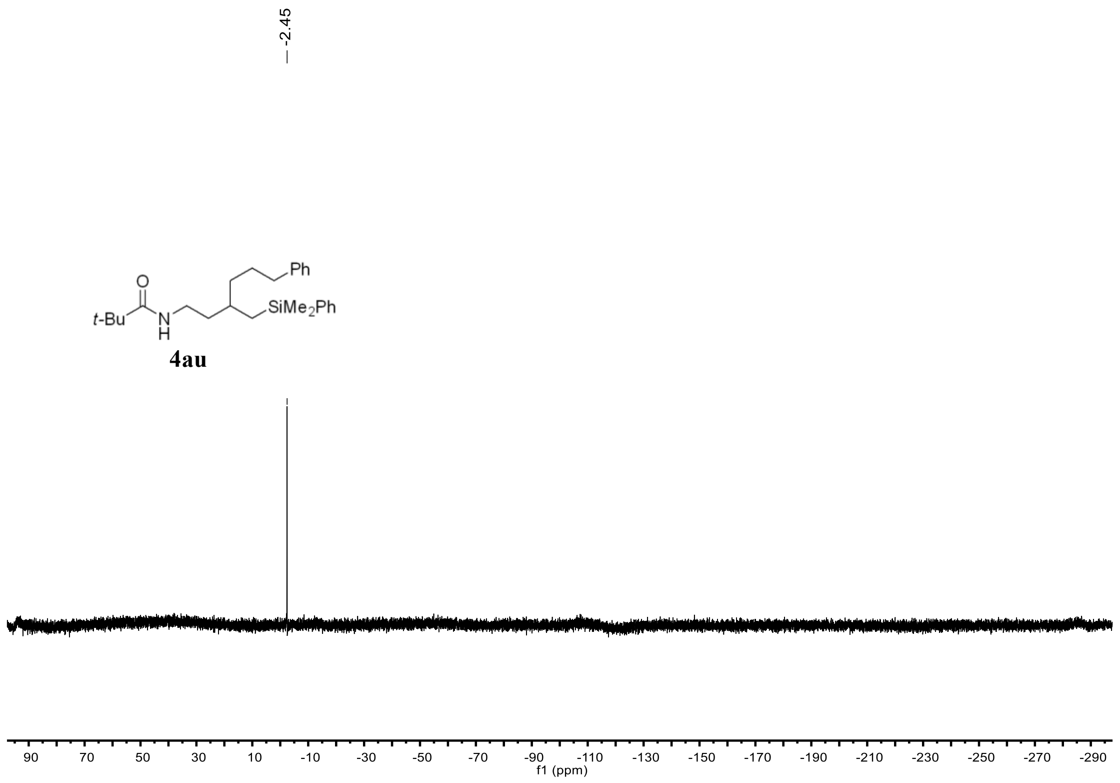

^{29}Si NMR Spectrum of **4au**

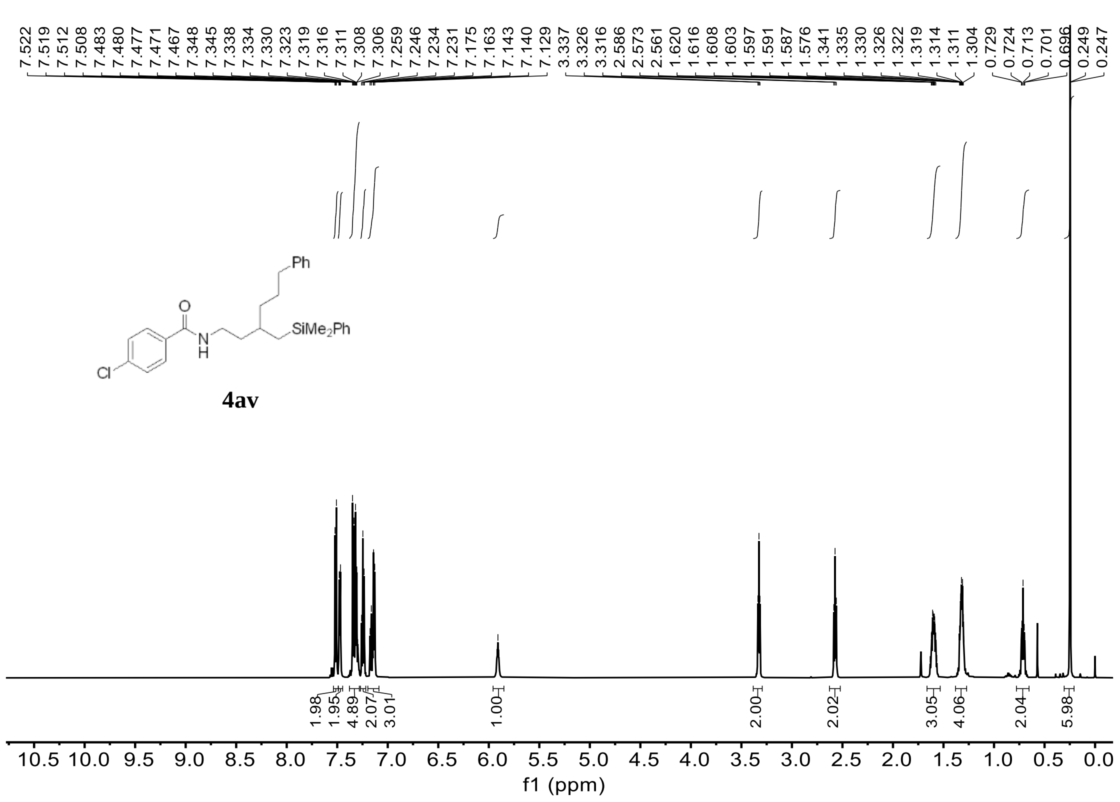

^{1}H NMR Spectrum of **4av**

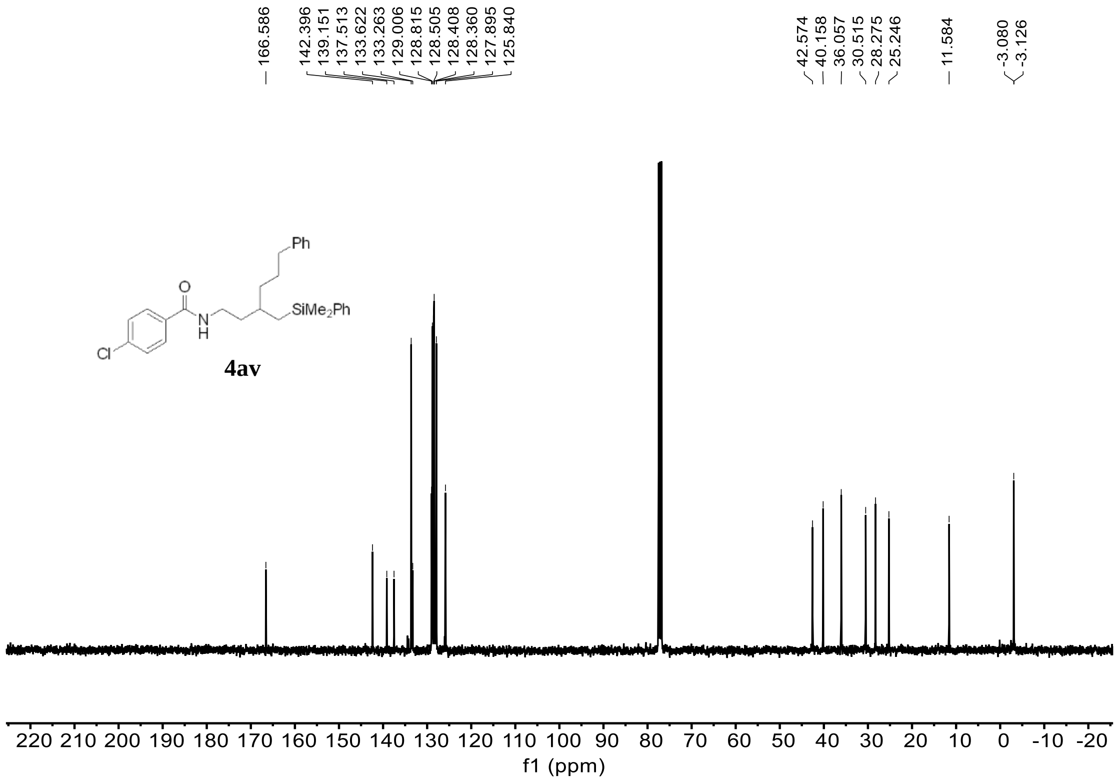

^{13}C NMR Spectrum of **4av**

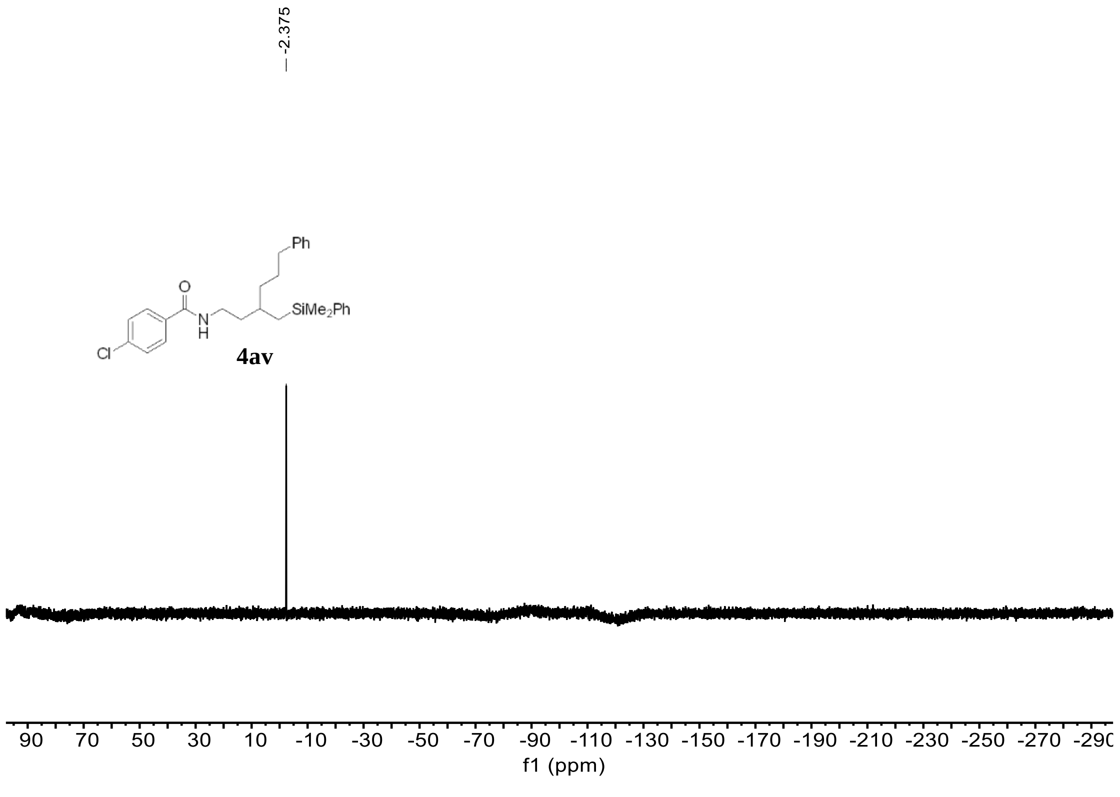

^{29}Si NMR Spectrum of **4av**

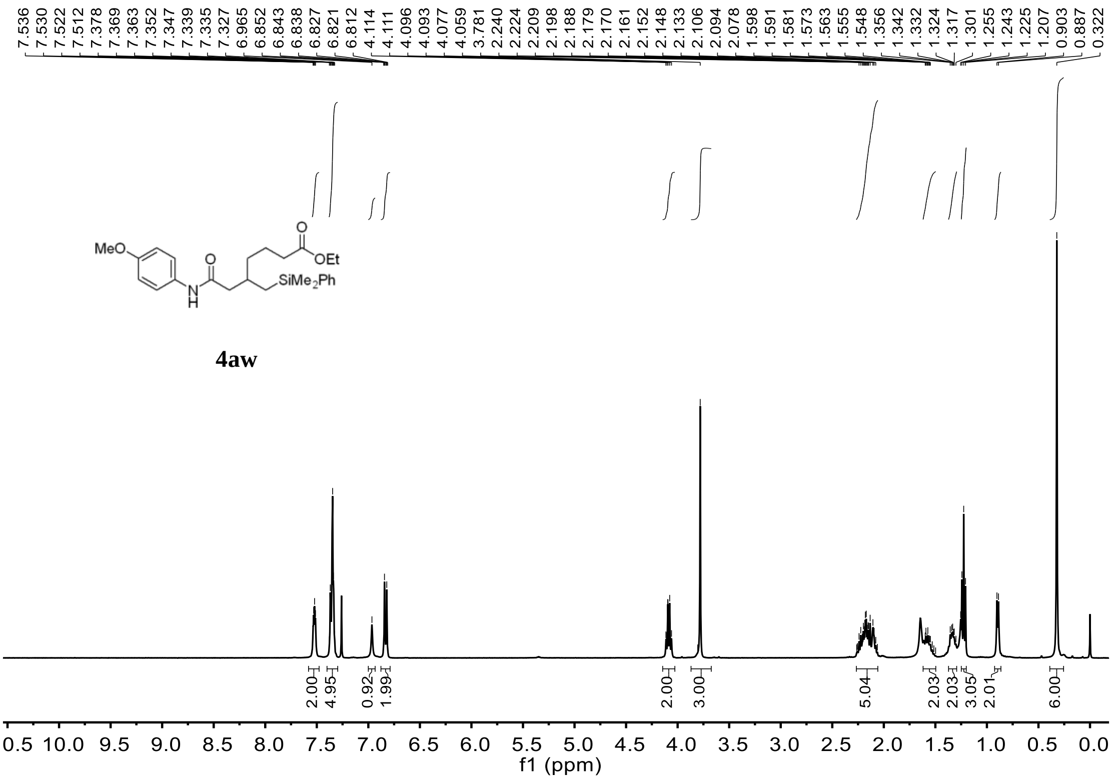

^{1}H NMR Spectrum of **4aw**

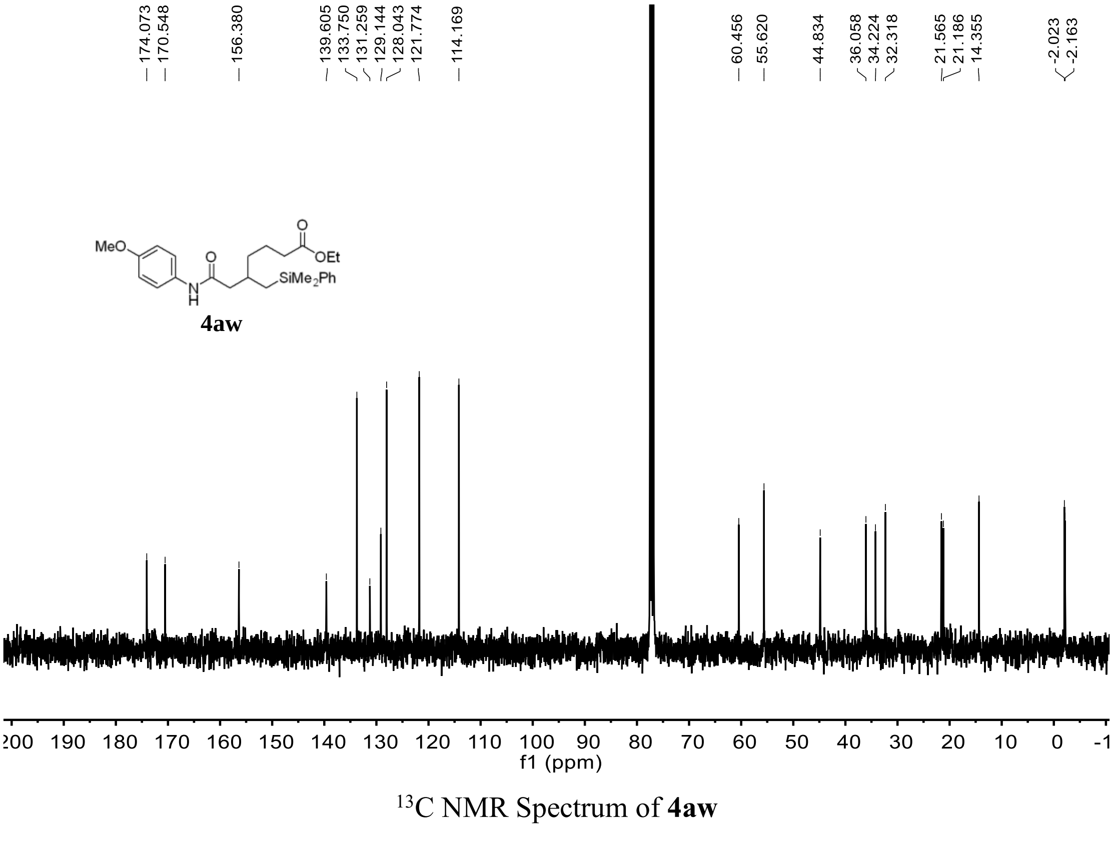

^{13}C NMR Spectrum of **4aw**

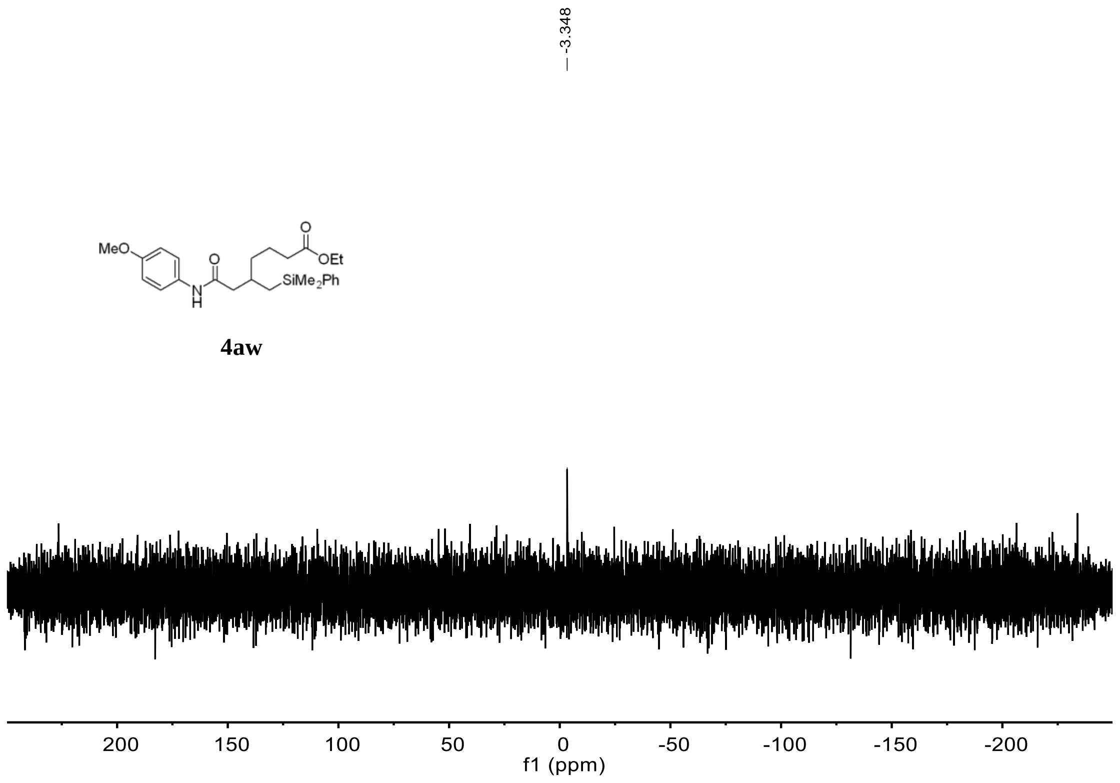

^{29}Si NMR Spectrum of **4aw**

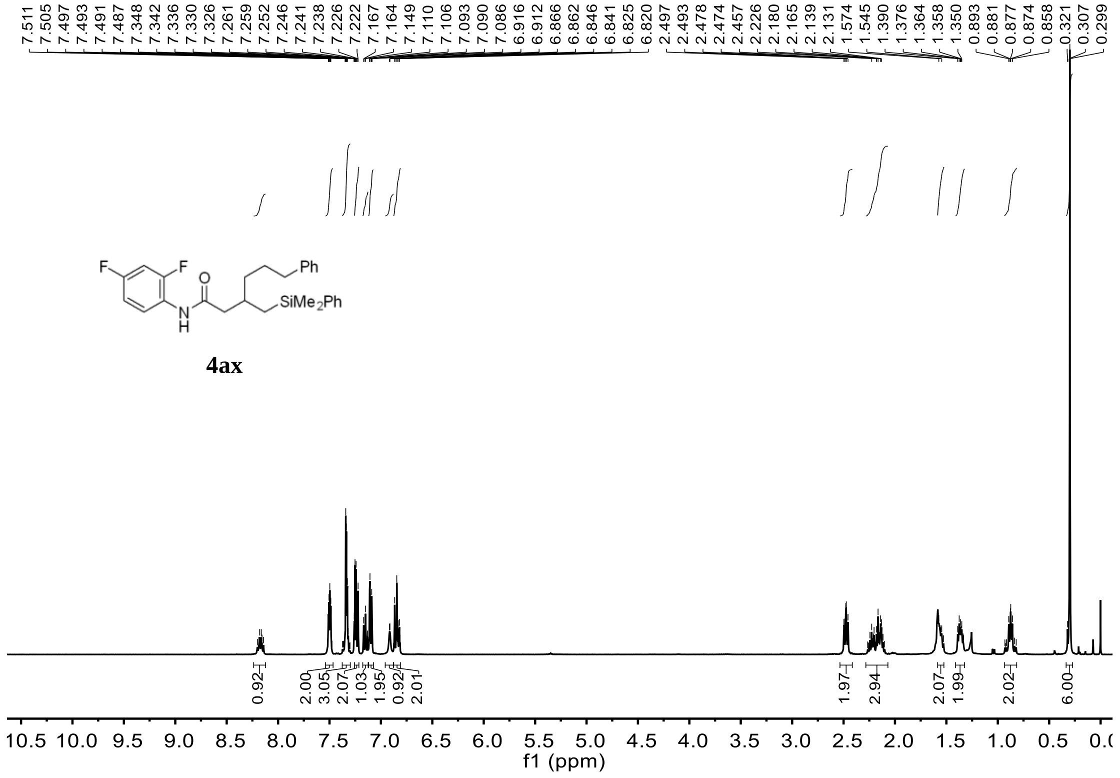

^{1}H NMR Spectrum of **4ax**

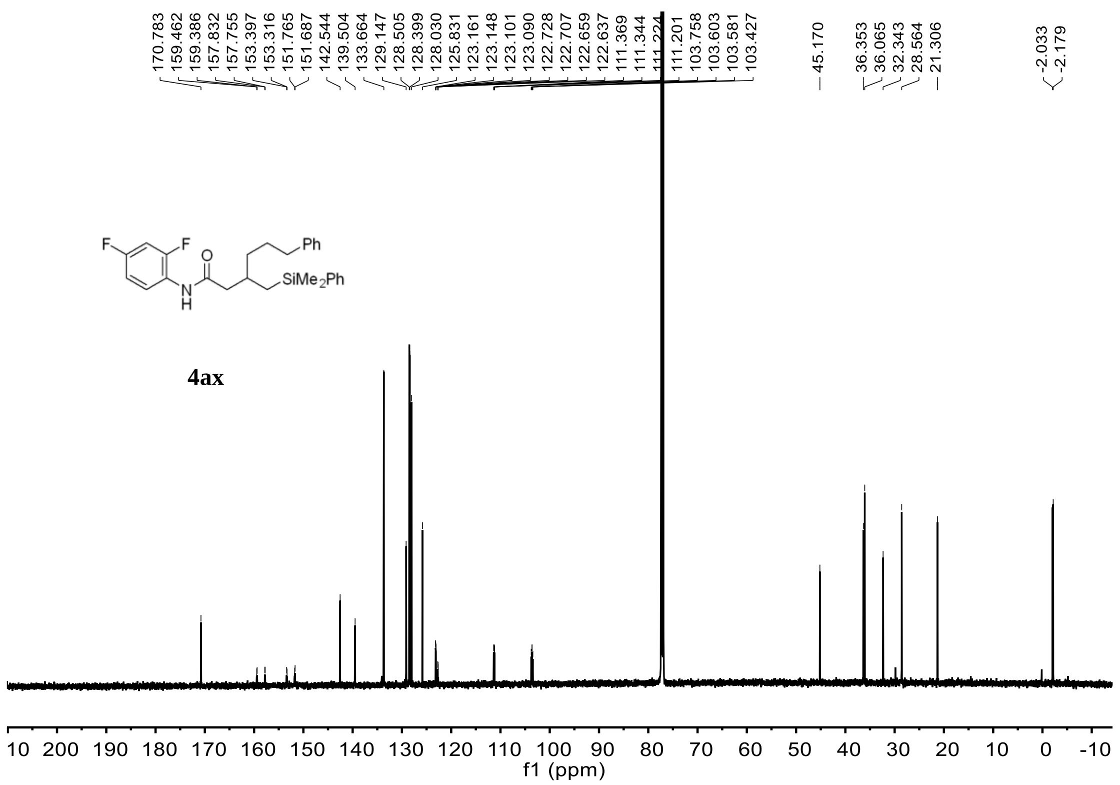

^{13}C NMR Spectrum of **4ax**

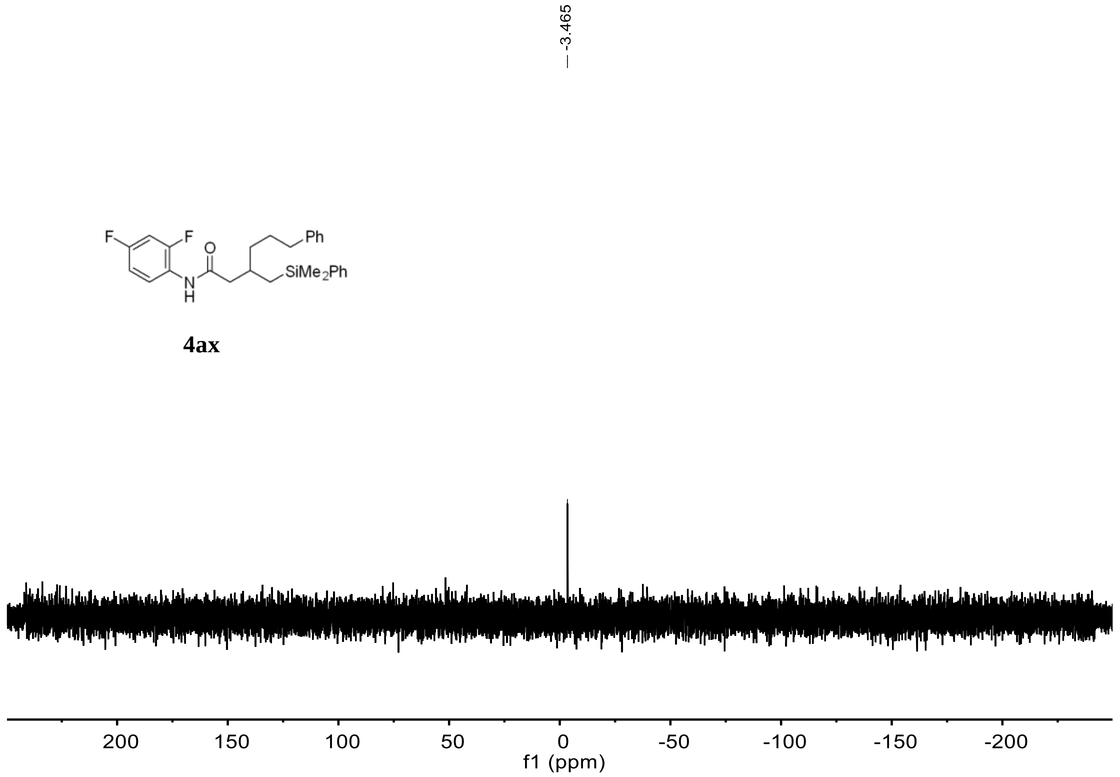

^{29}Si NMR Spectrum of **4ax**

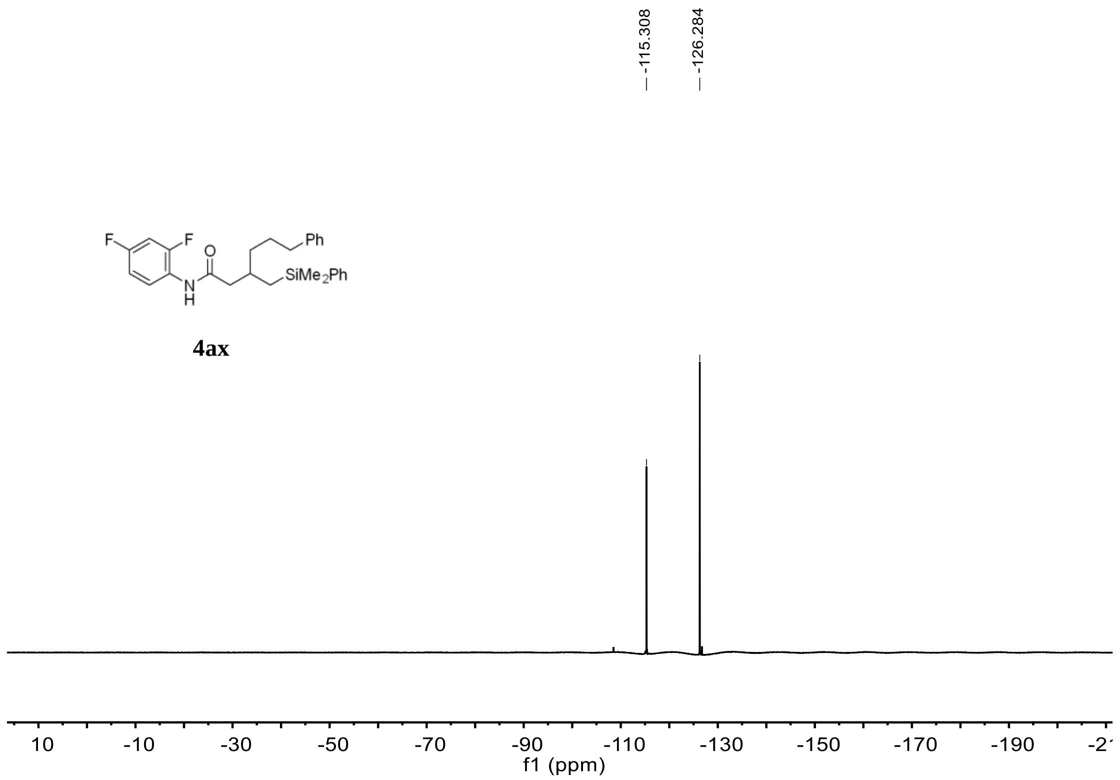

^{19}F NMR Spectrum of **4ax**

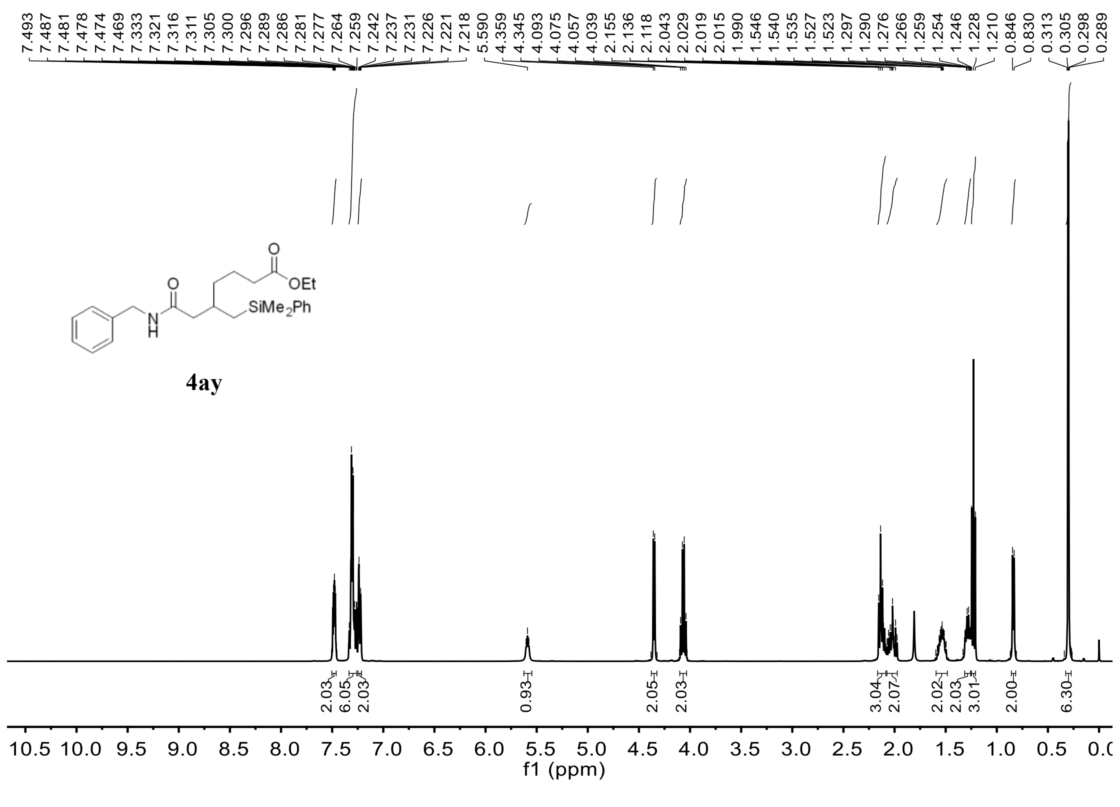

^{1}H NMR Spectrum of **4ay**

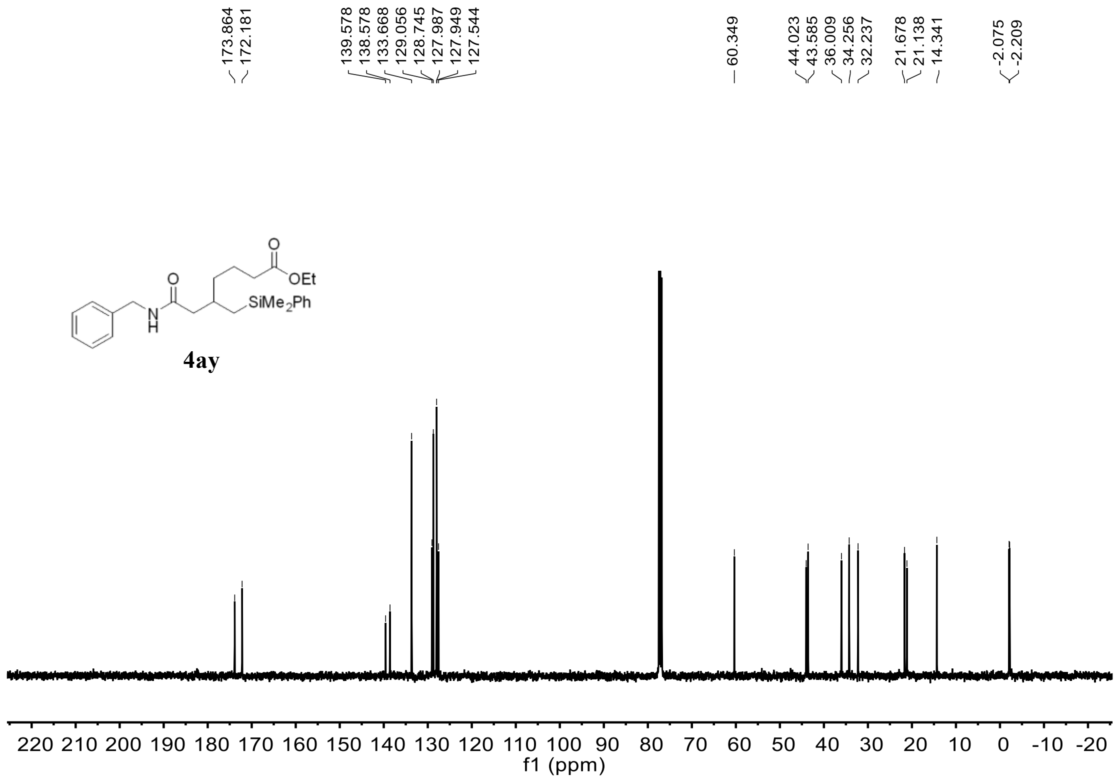

^{13}C NMR Spectrum of **4ay**

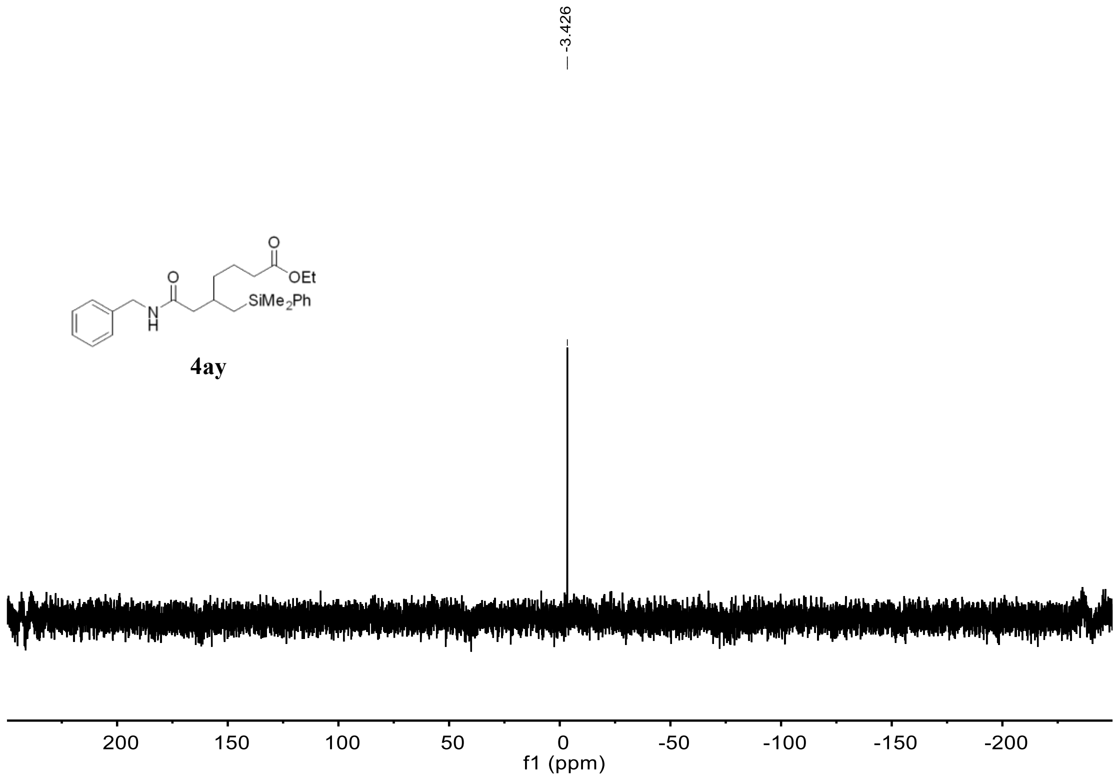

^{29}Si NMR Spectrum of **4ay**

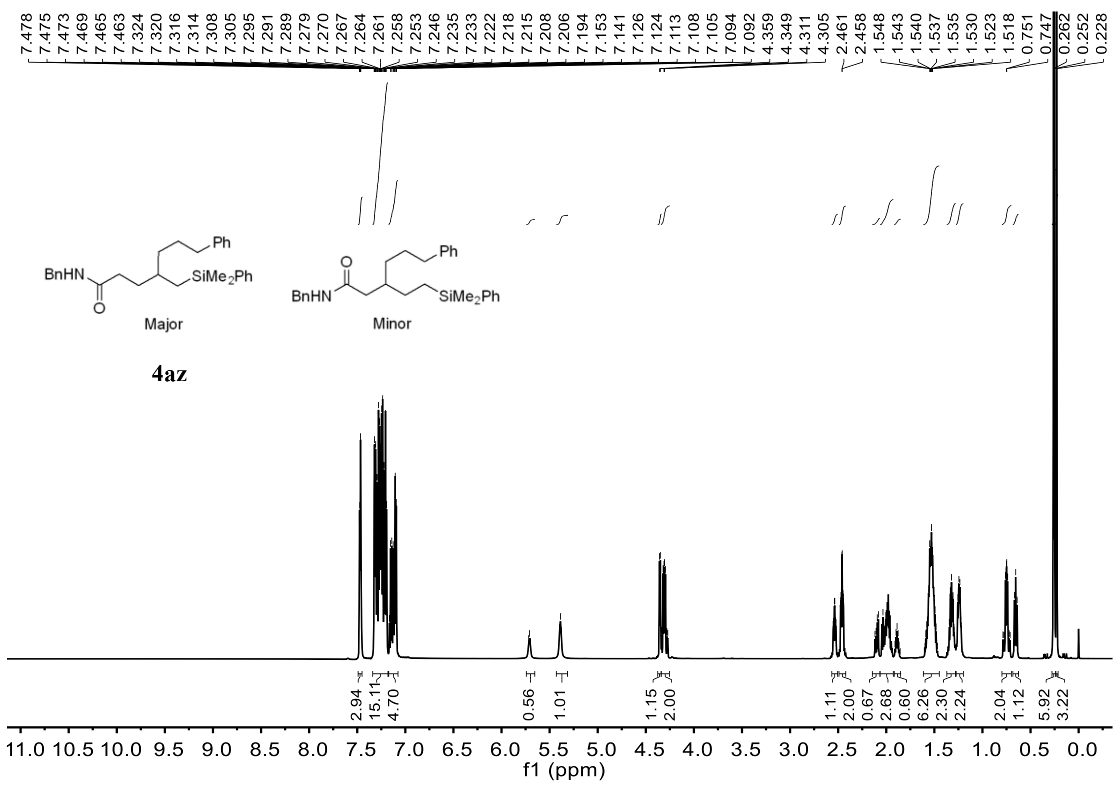

^{1}H NMR Spectrum of **4az**

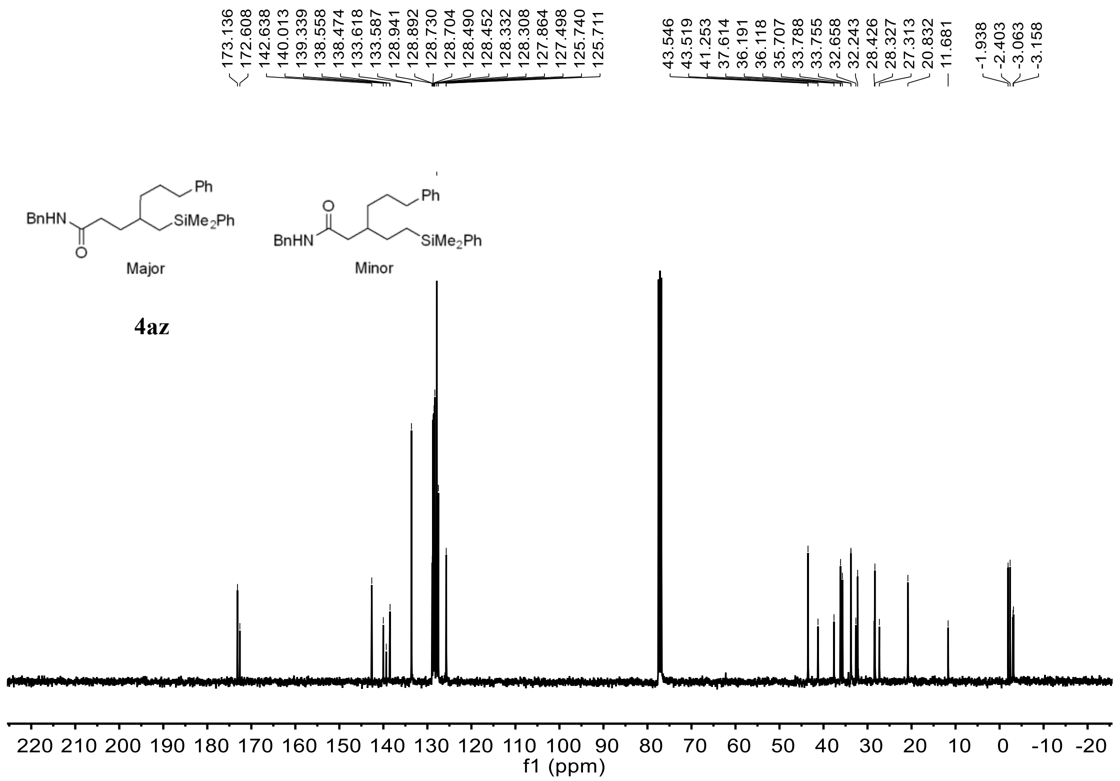

^{13}C NMR Spectrum of **4az**

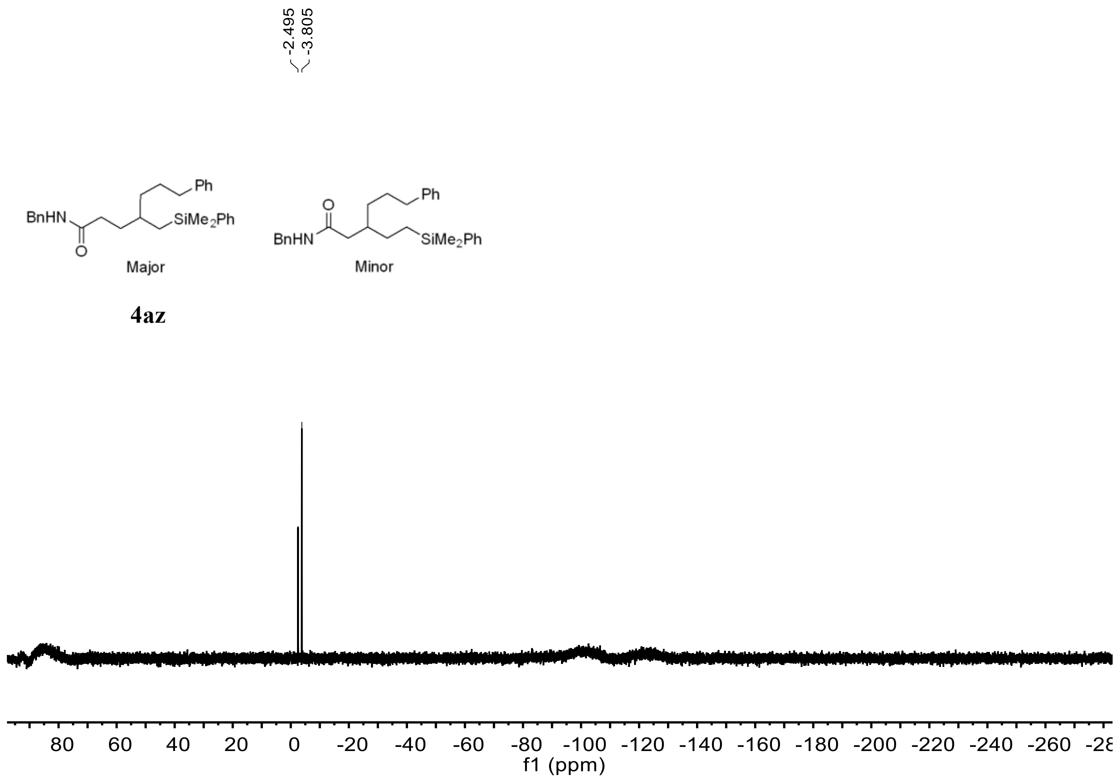

^{29}Si NMR Spectrum of **4az**

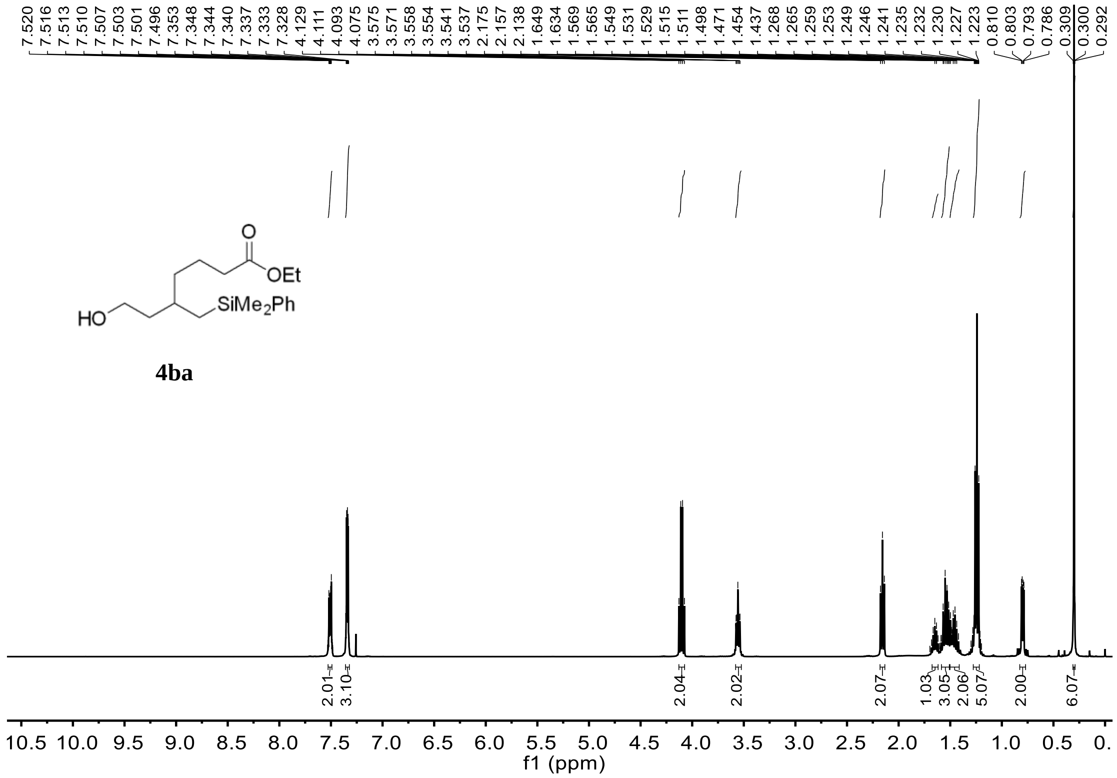

^{1}H NMR Spectrum of **4ba**

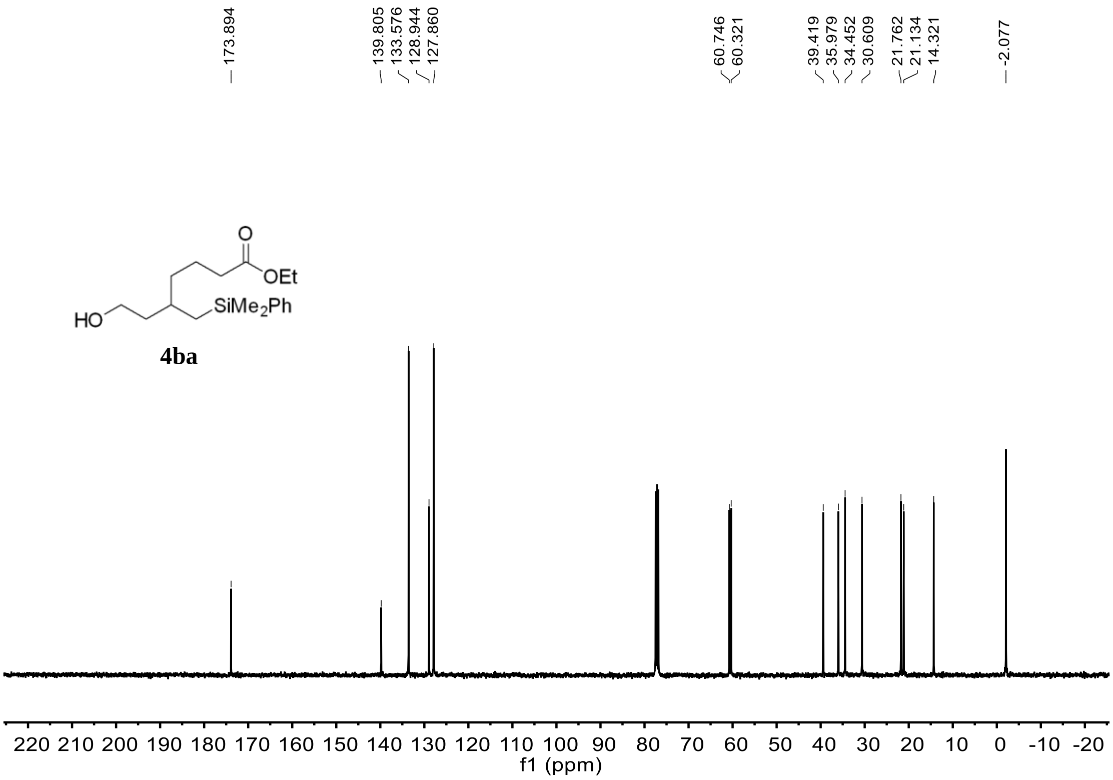

^{13}C NMR Spectrum of **4ar**

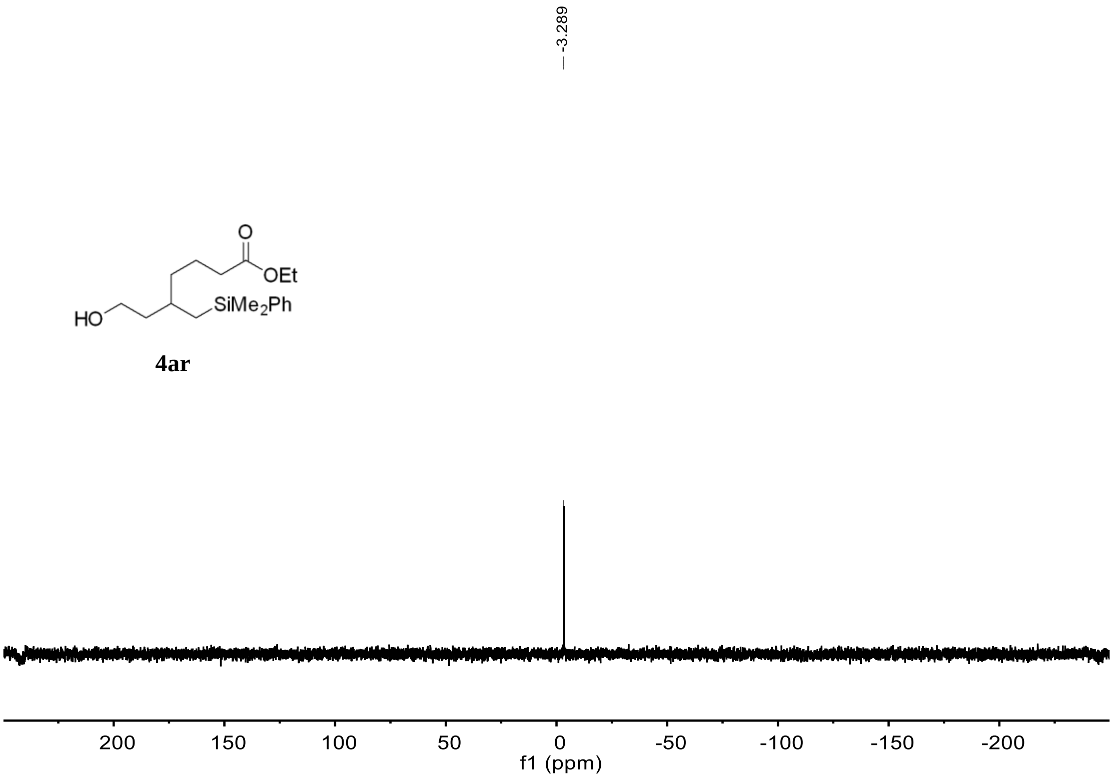

^{29}Si NMR Spectrum of **4ba**

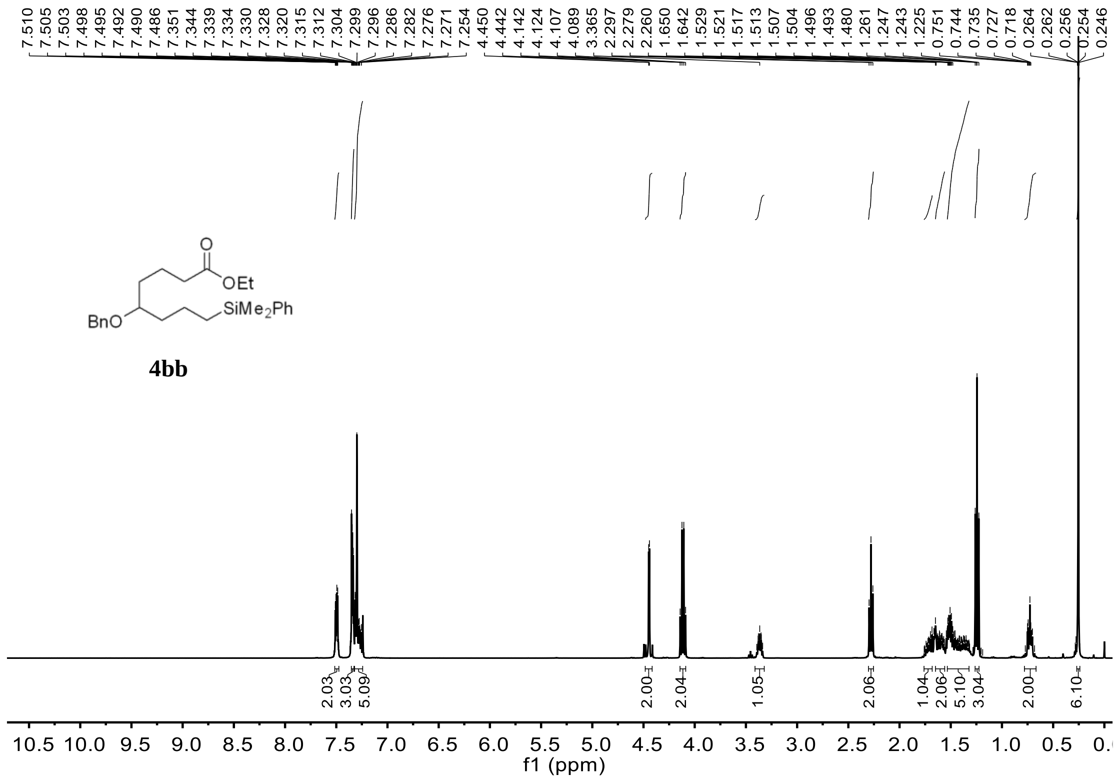

^{1}H NMR Spectrum of **4bb**

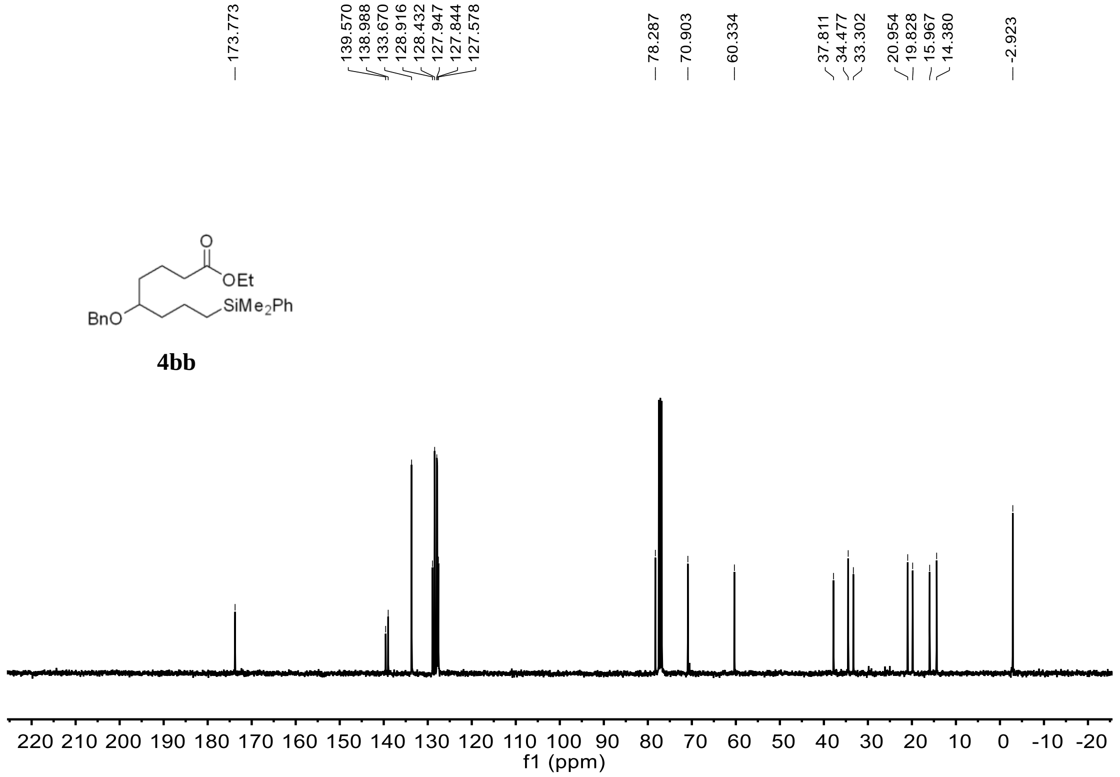

^{13}C NMR Spectrum of **4bb**

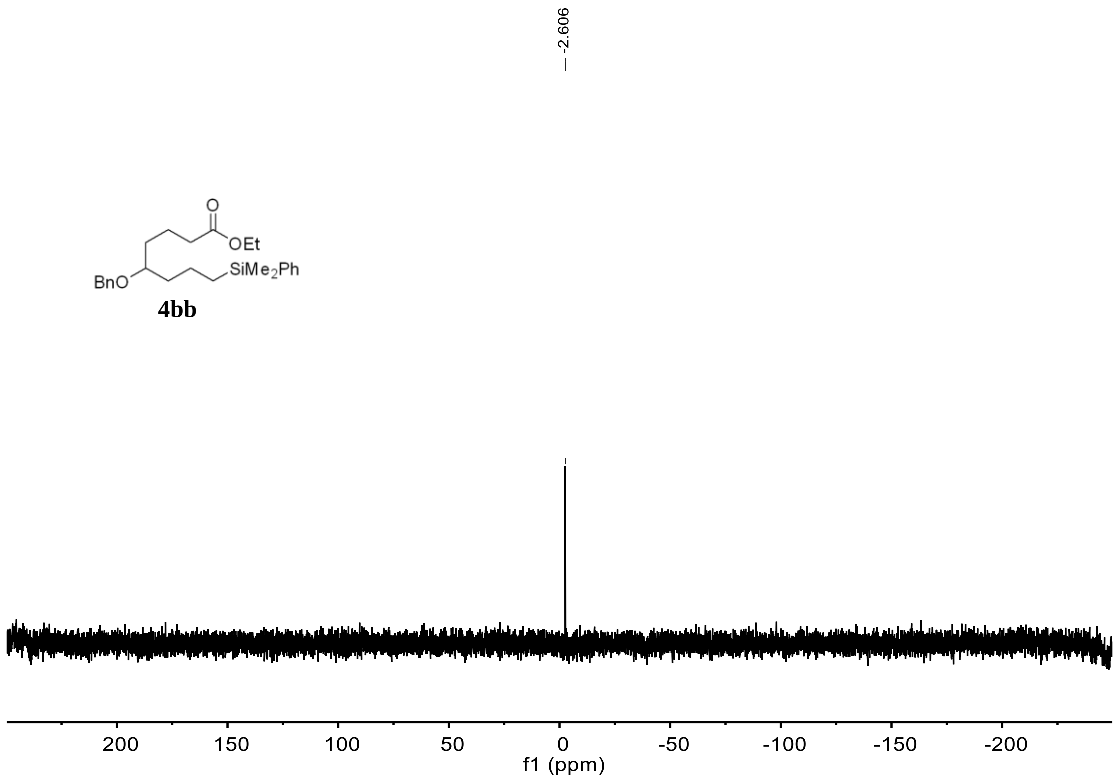

^{29}Si NMR Spectrum of **4bb**

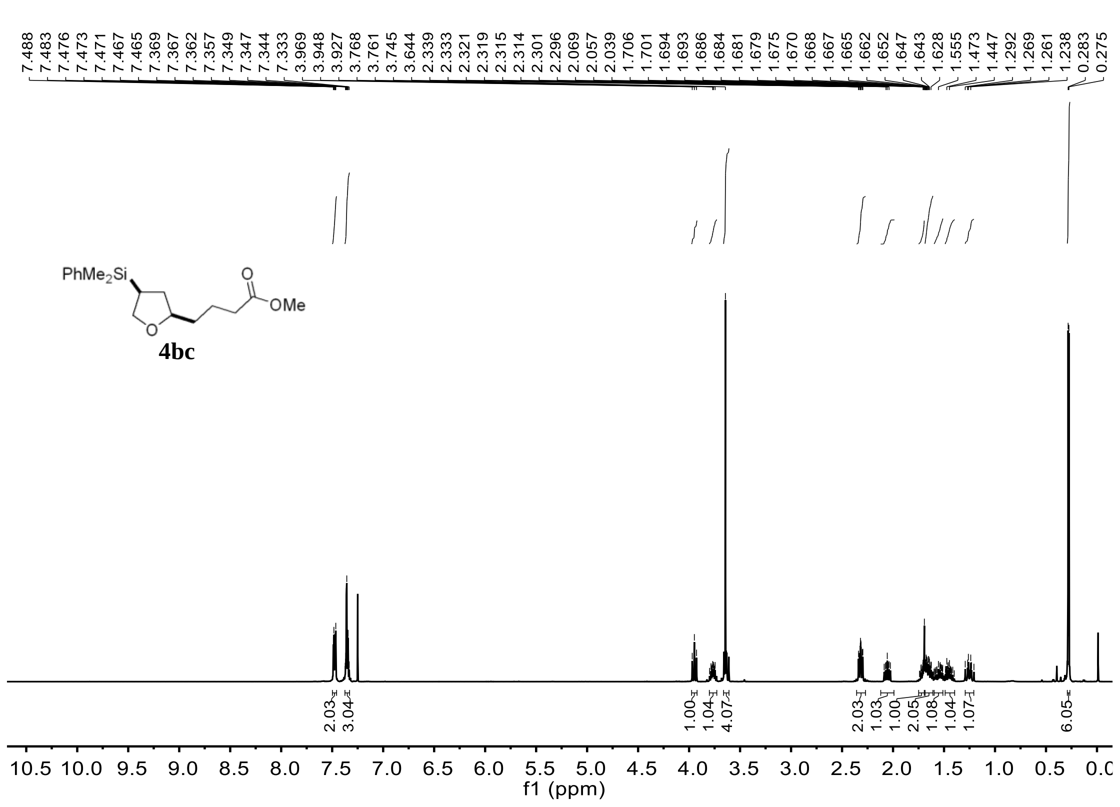

^{1}H NMR Spectrum of **4bc**

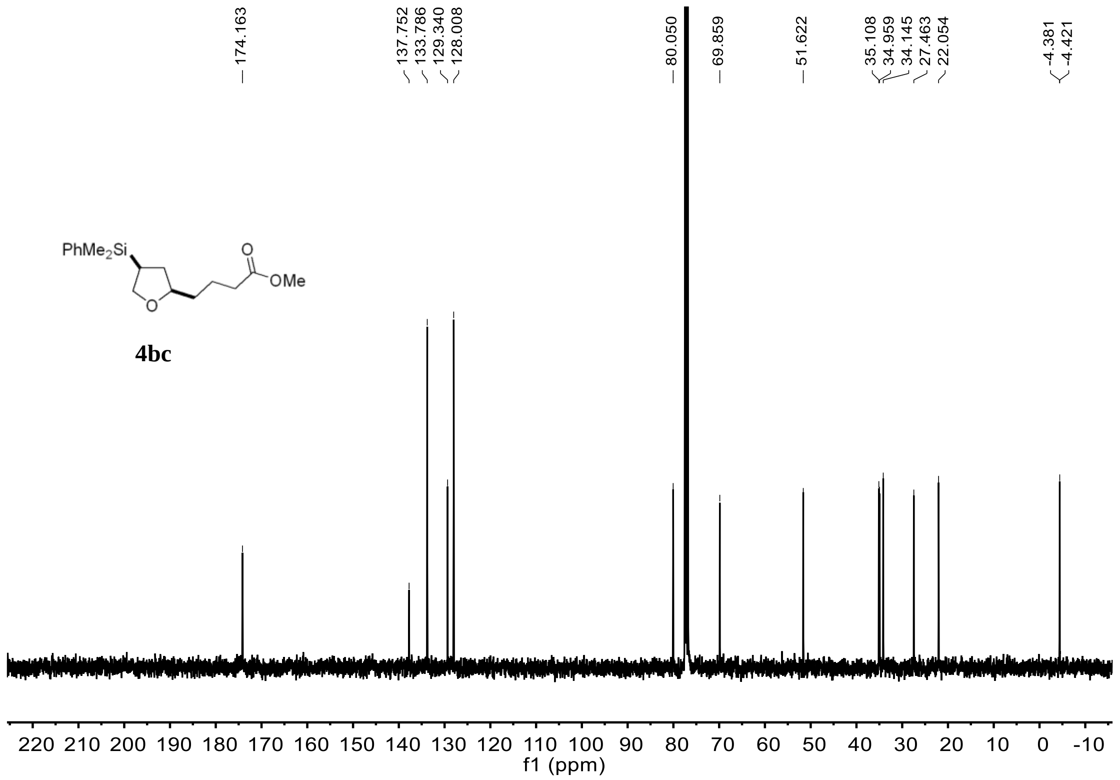

^{13}C NMR Spectrum of **4bc**

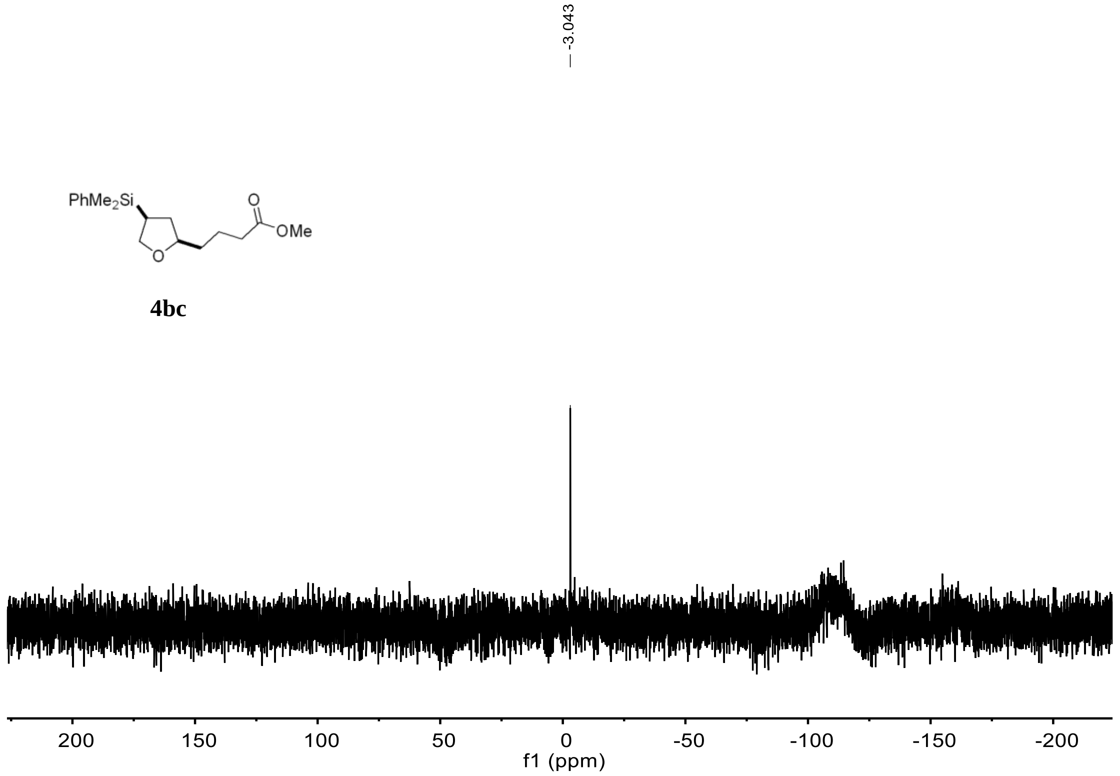

^{29}Si NMR Spectrum of **4bc**

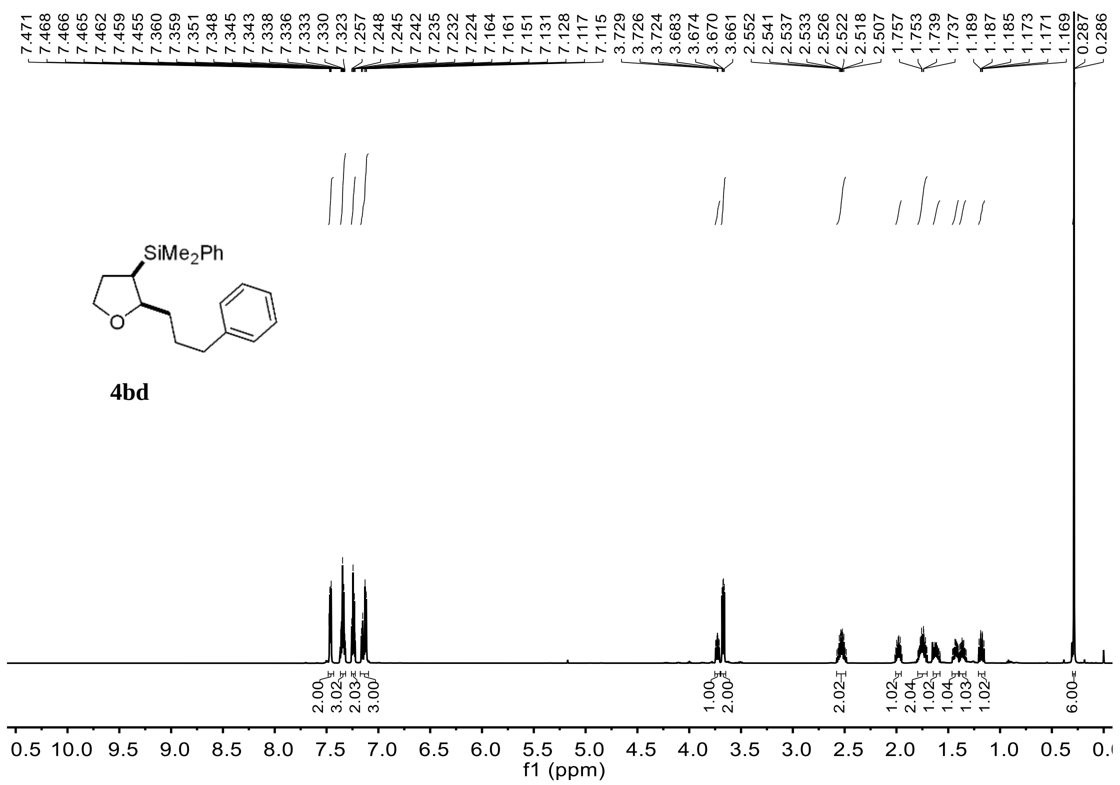

^{1}H NMR Spectrum of **4bd**

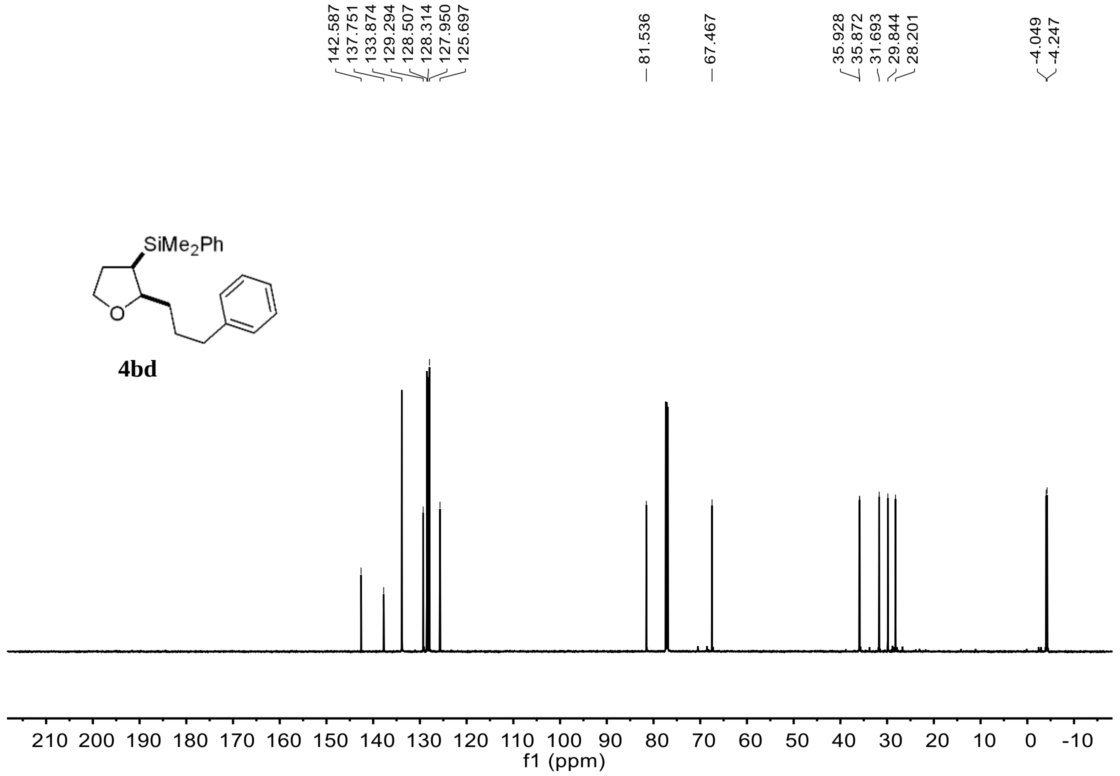

^{13}C NMR Spectrum of **4bd**

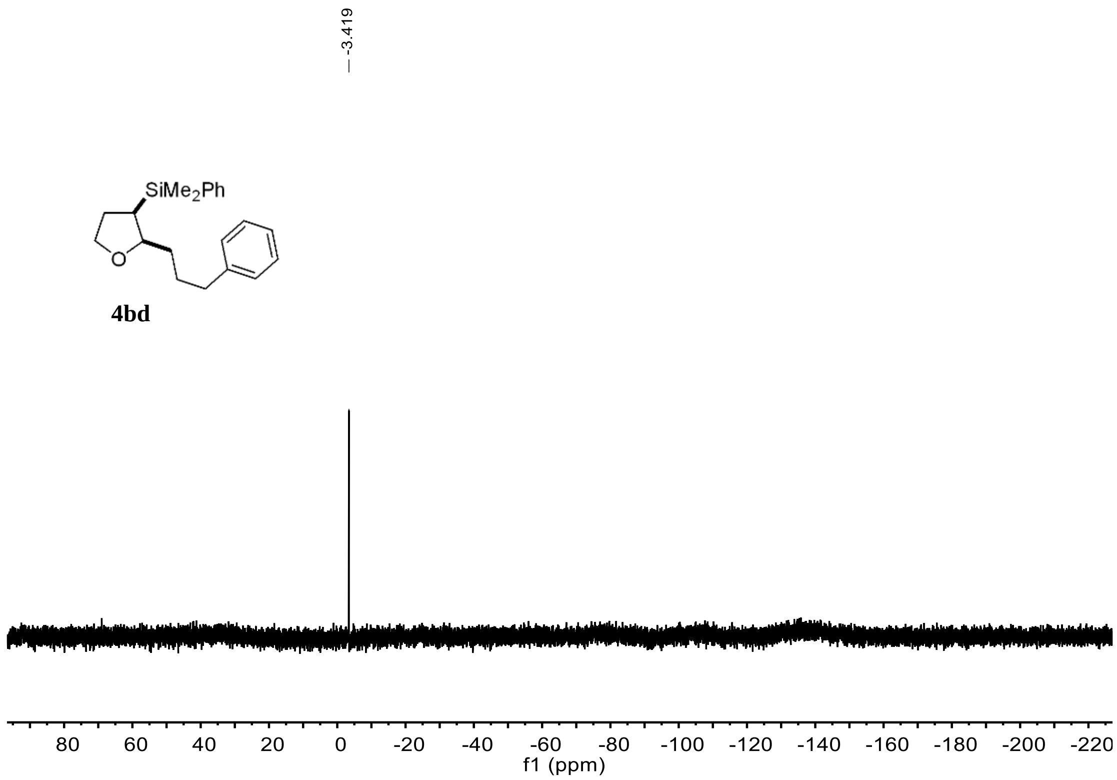

^{29}Si NMR Spectrum of **4bd**

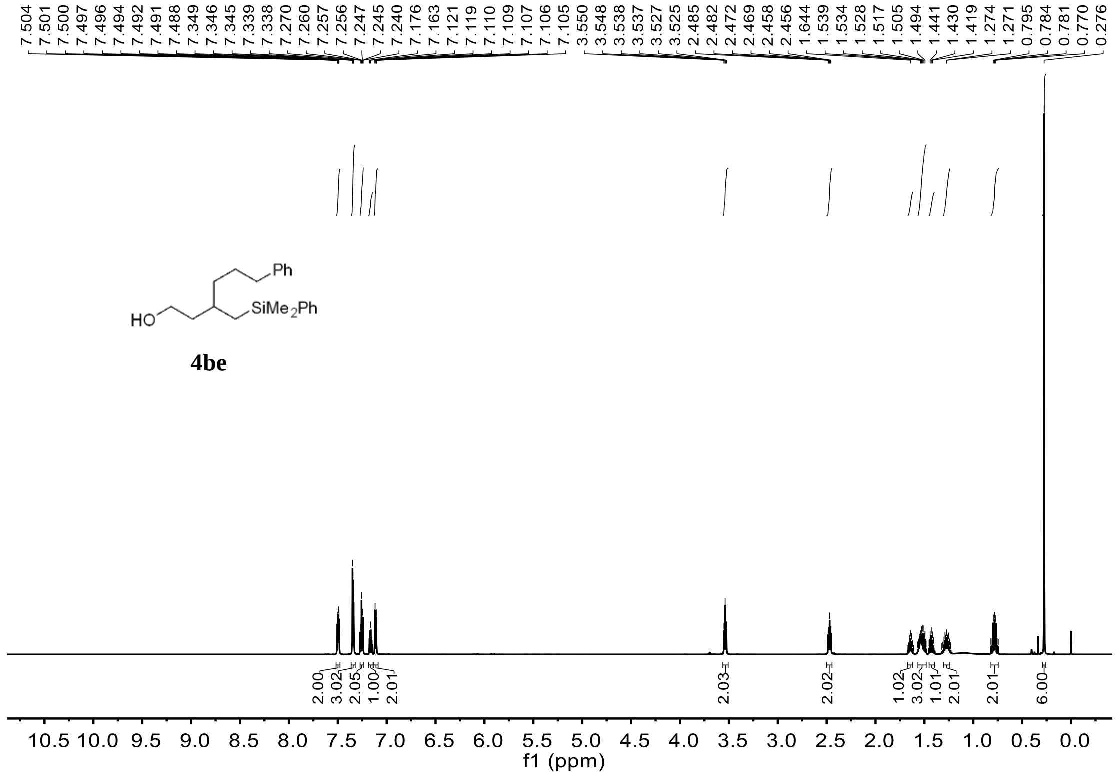

^{1}H NMR Spectrum of **4be**

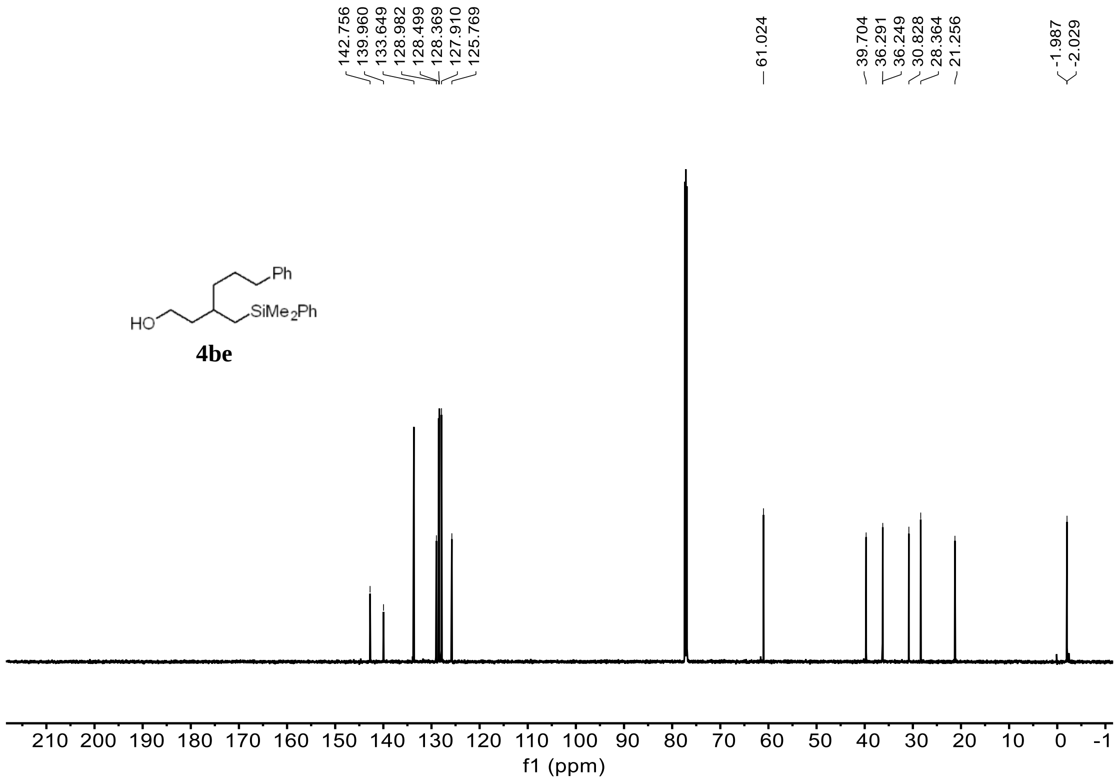

^{13}C NMR Spectrum of **4be**

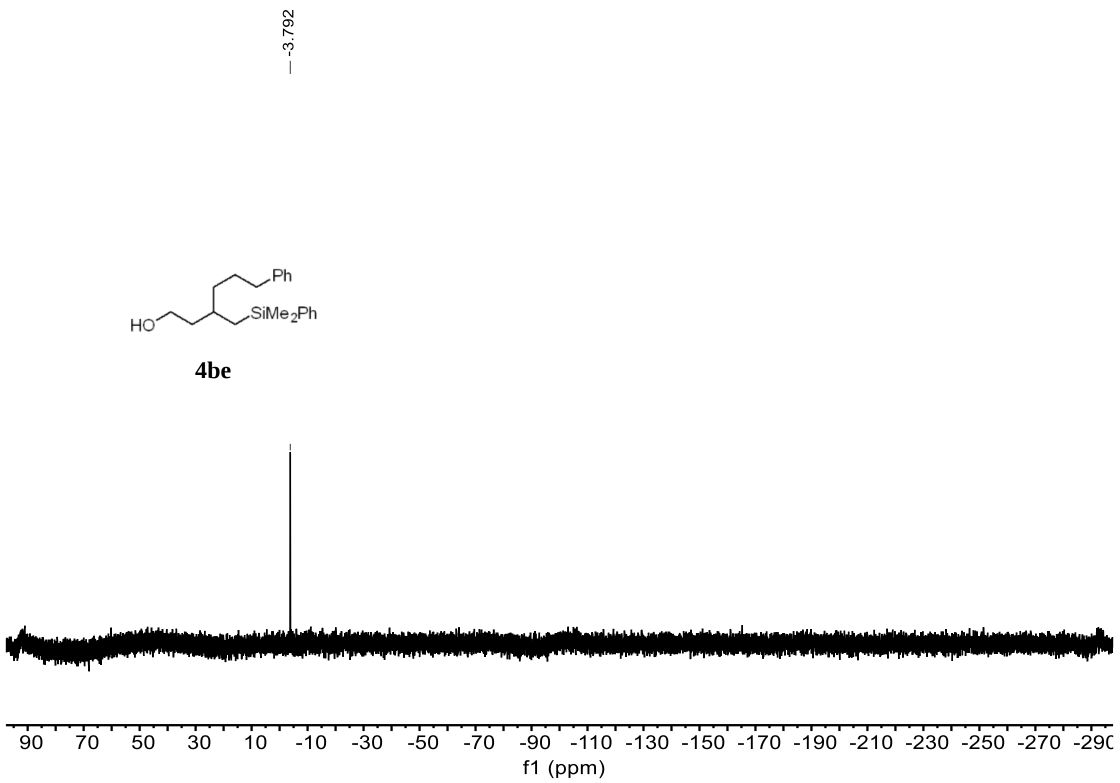

^{29}Si NMR Spectrum of **4be**

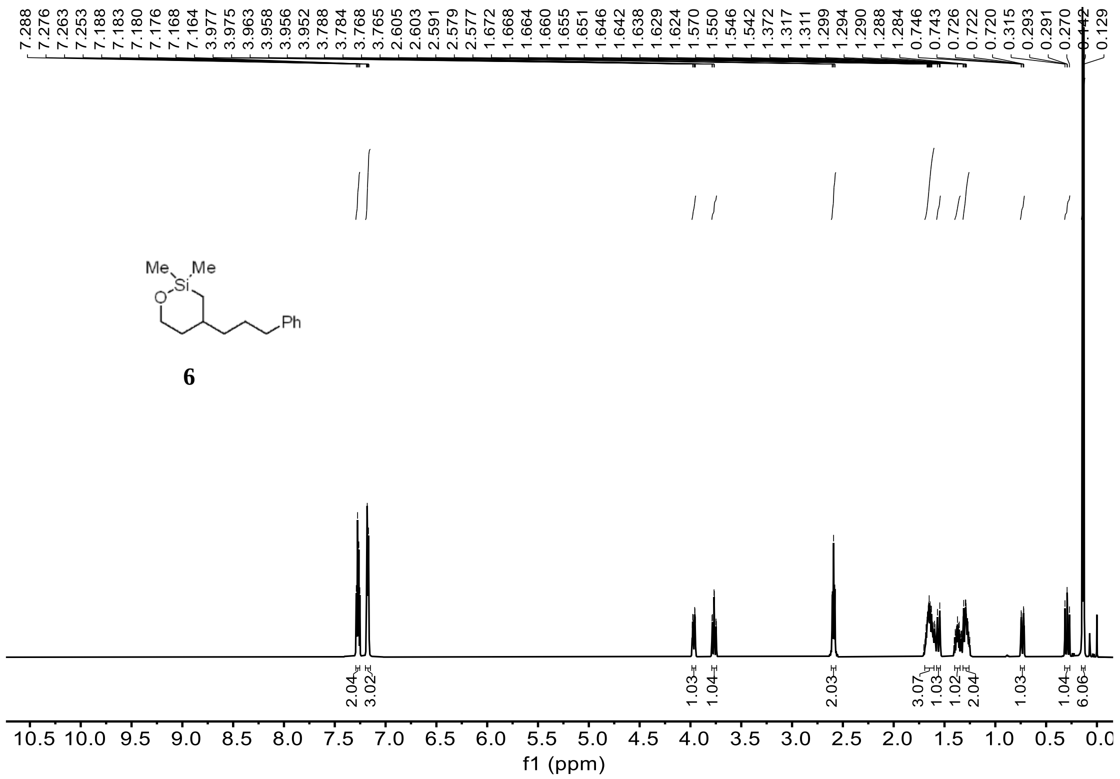

^{1}H NMR Spectrum of **6**

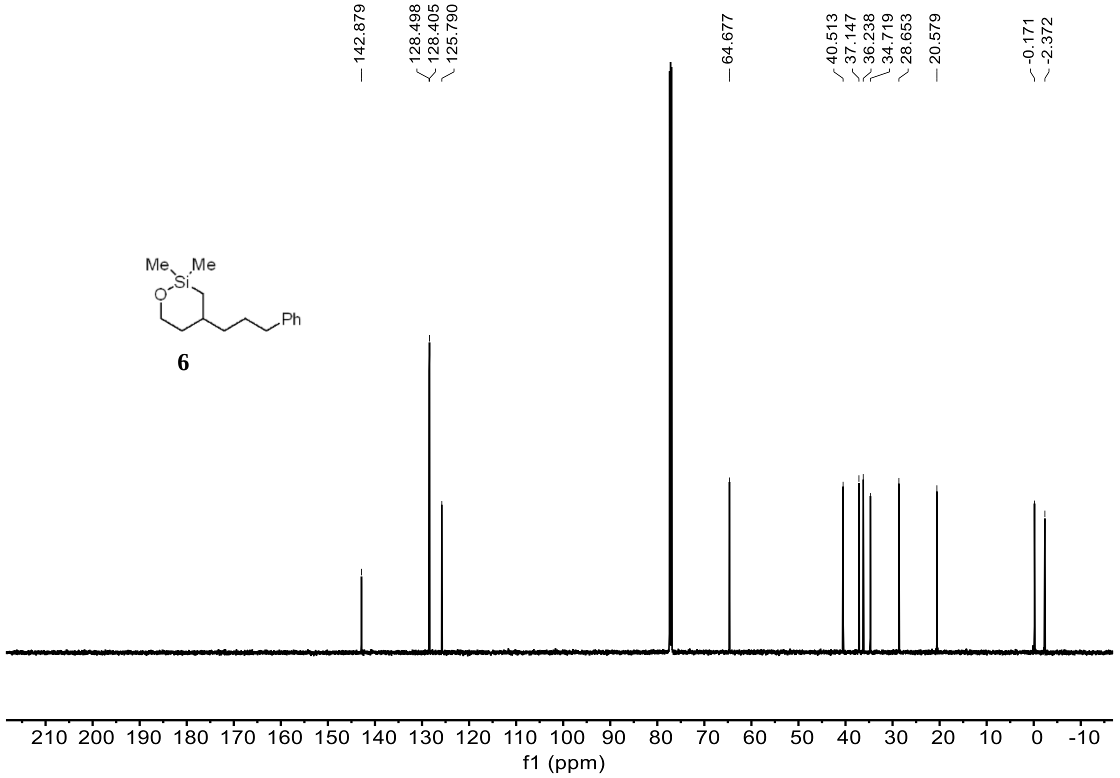

^{13}C NMR Spectrum of **6**

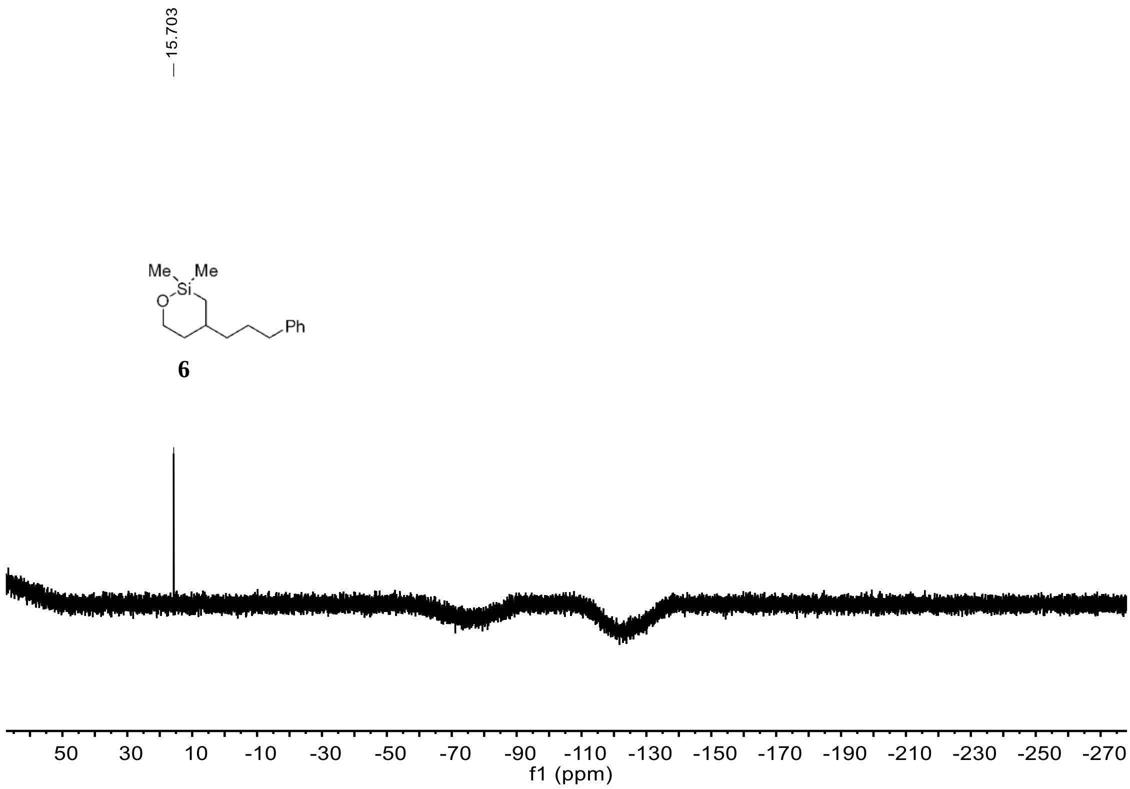

^{29}Si NMR Spectrum of **6**

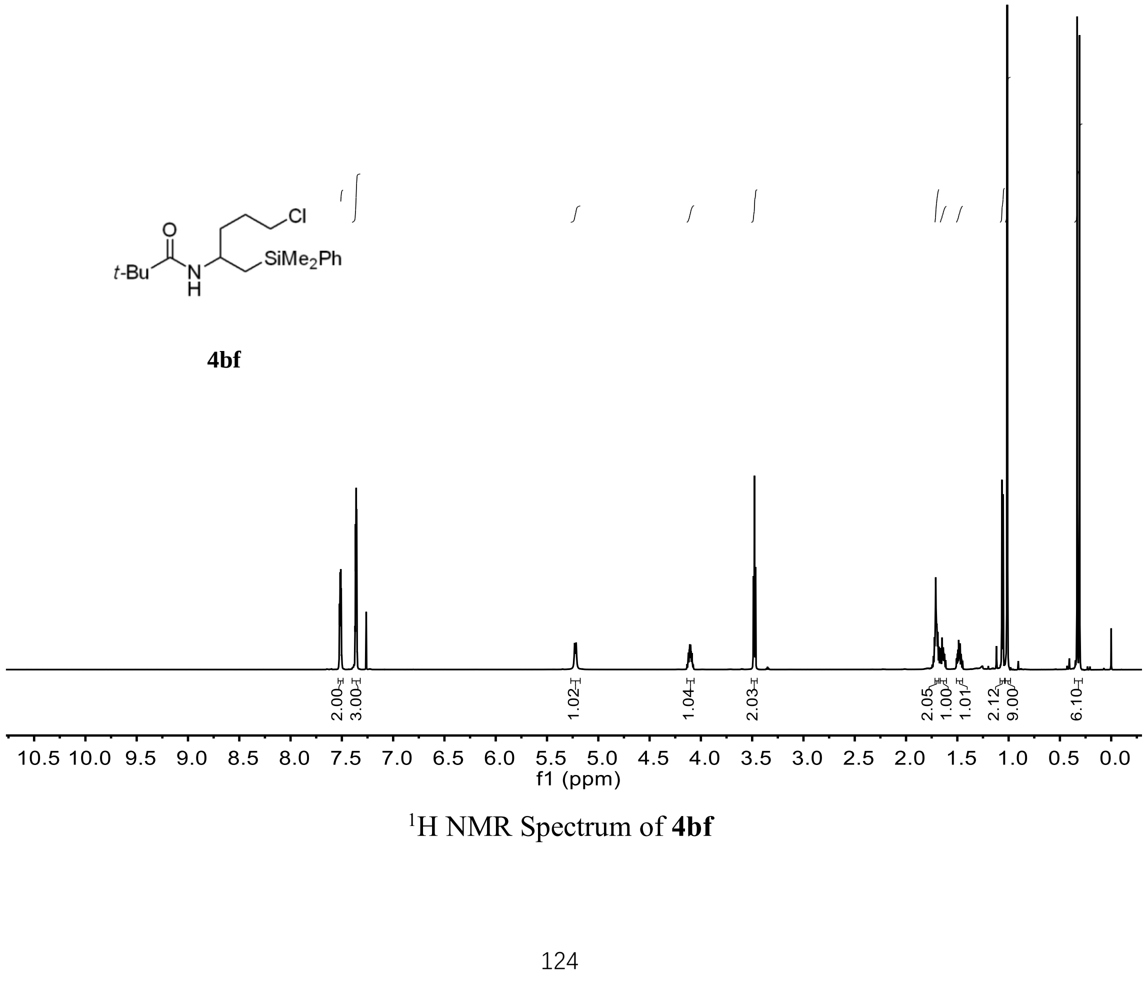

^{1}H NMR Spectrum of **4bf**

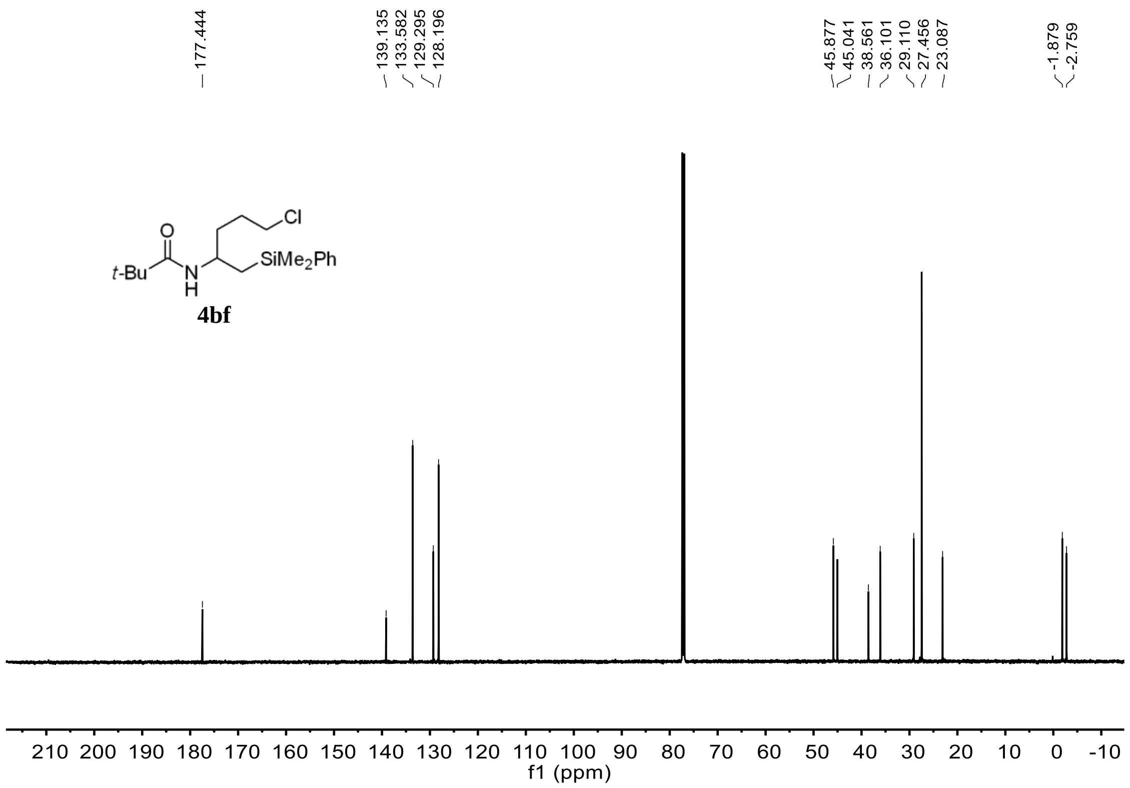

^{13}C NMR Spectrum of **4bf**

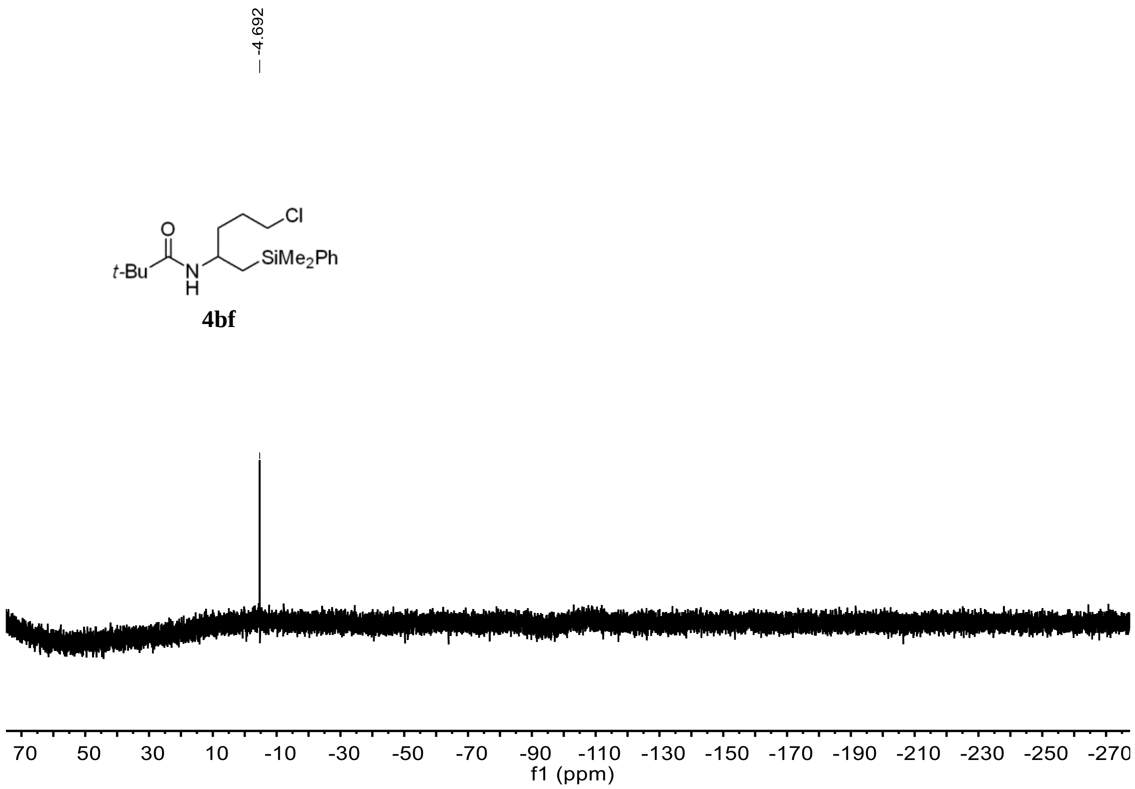

^{29}Si NMR Spectrum of **4bf**

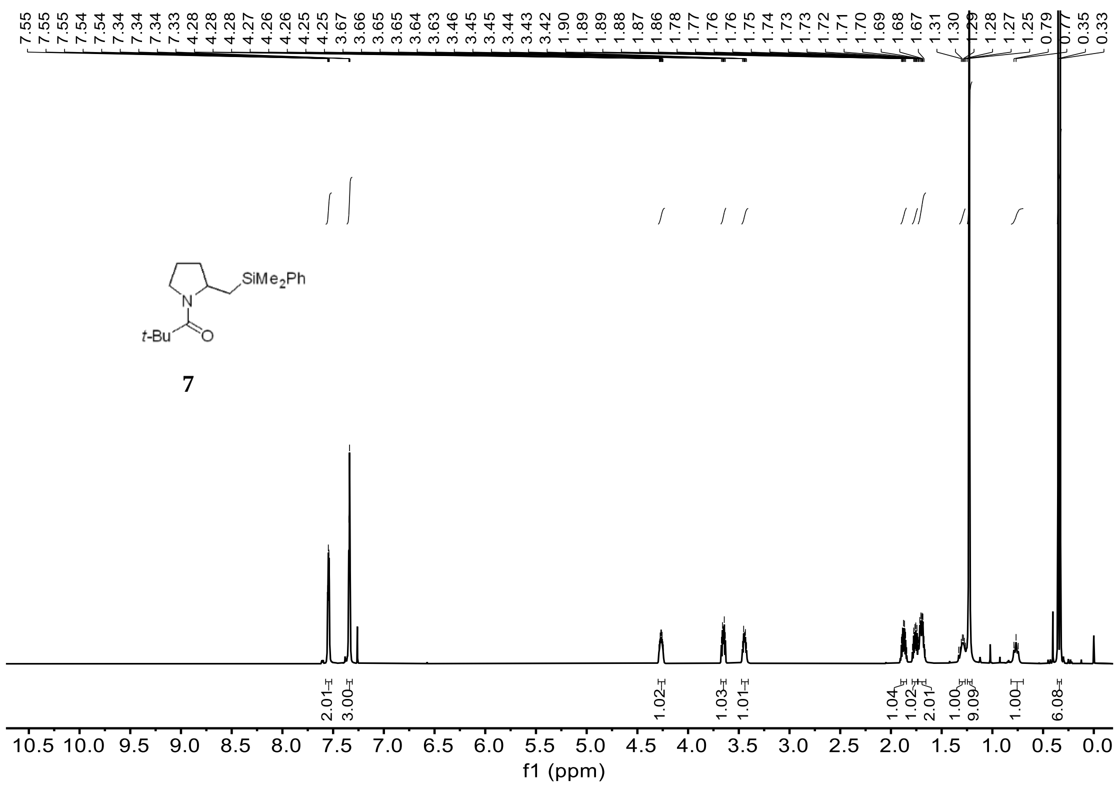

^{1}H NMR Spectrum of **7**

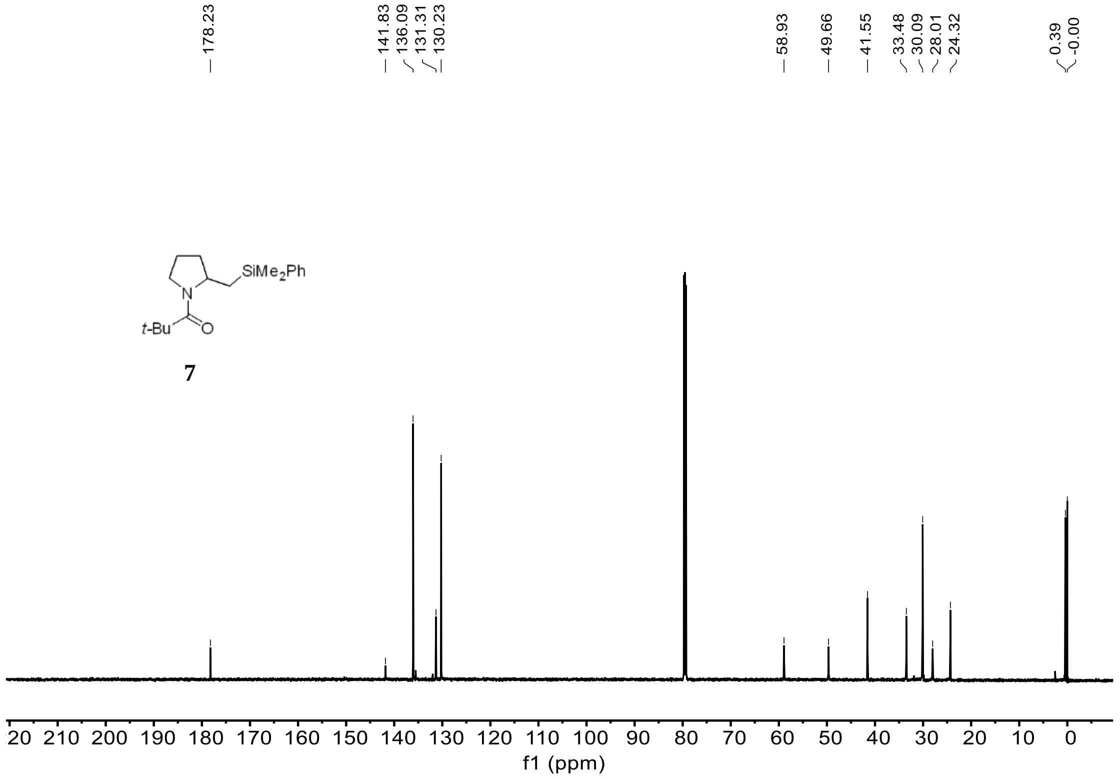

^{13}C NMR Spectrum of **7**

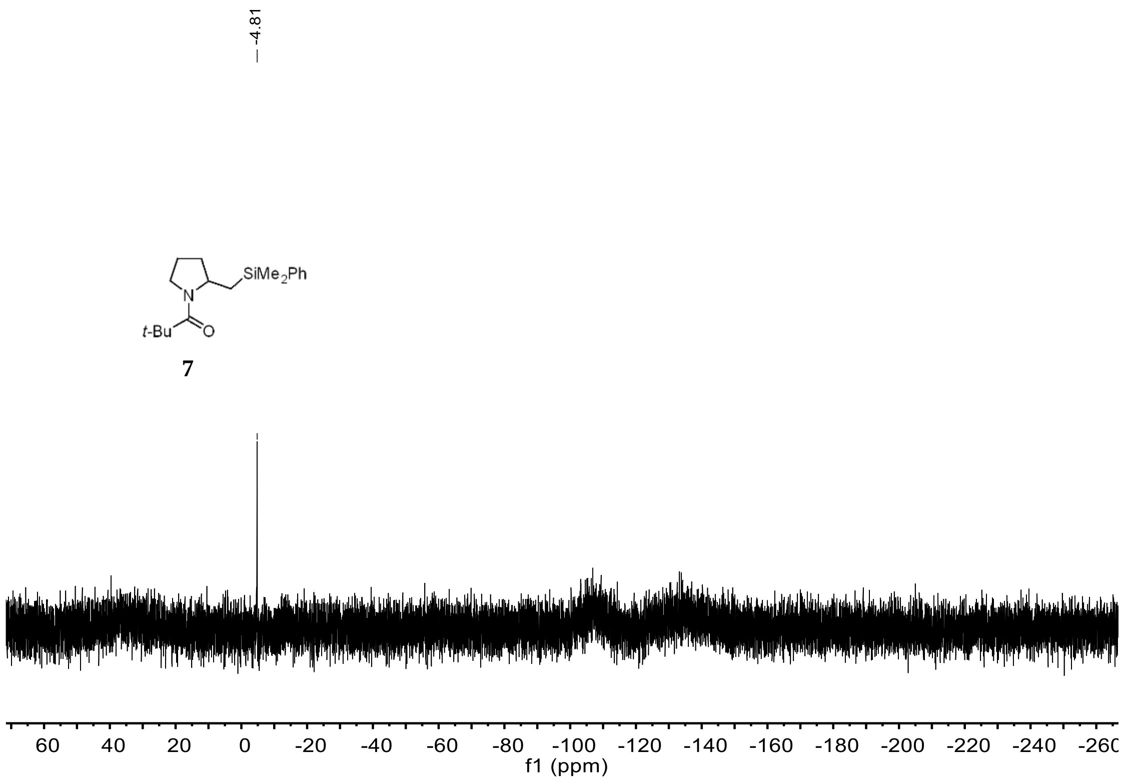

^{29}Si NMR Spectrum of **7**

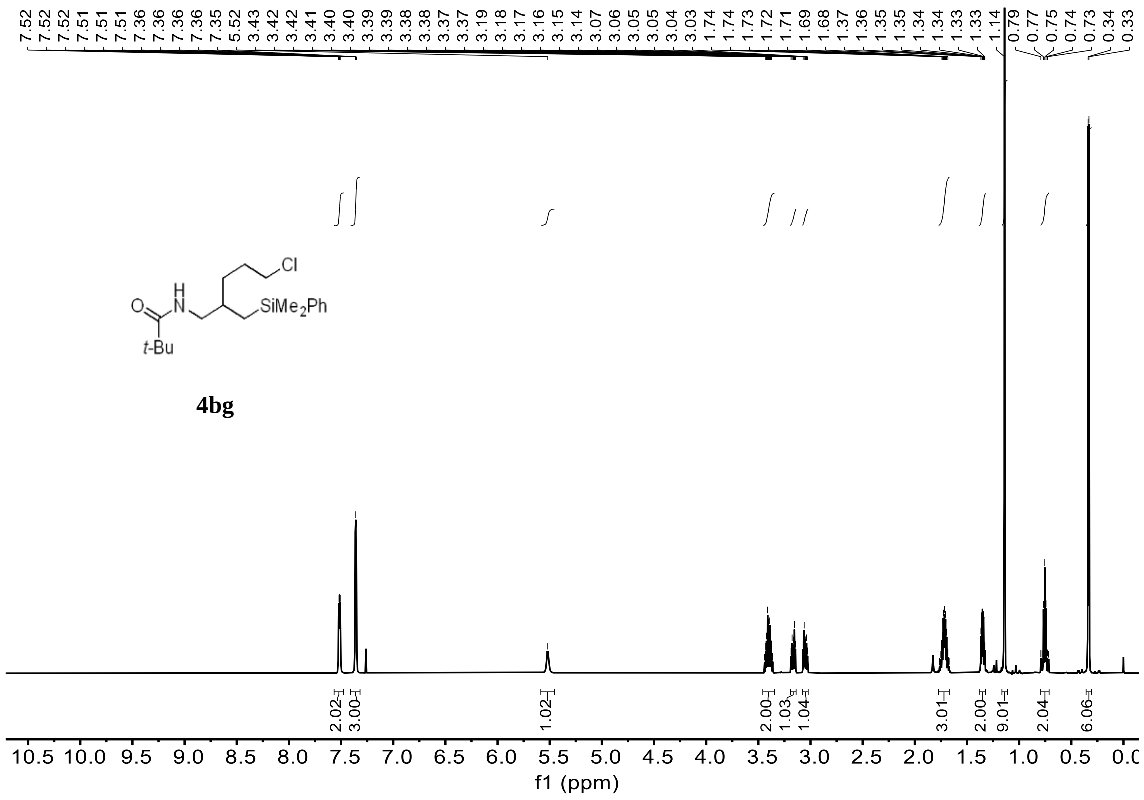

^{1}H NMR Spectrum of **4bg**

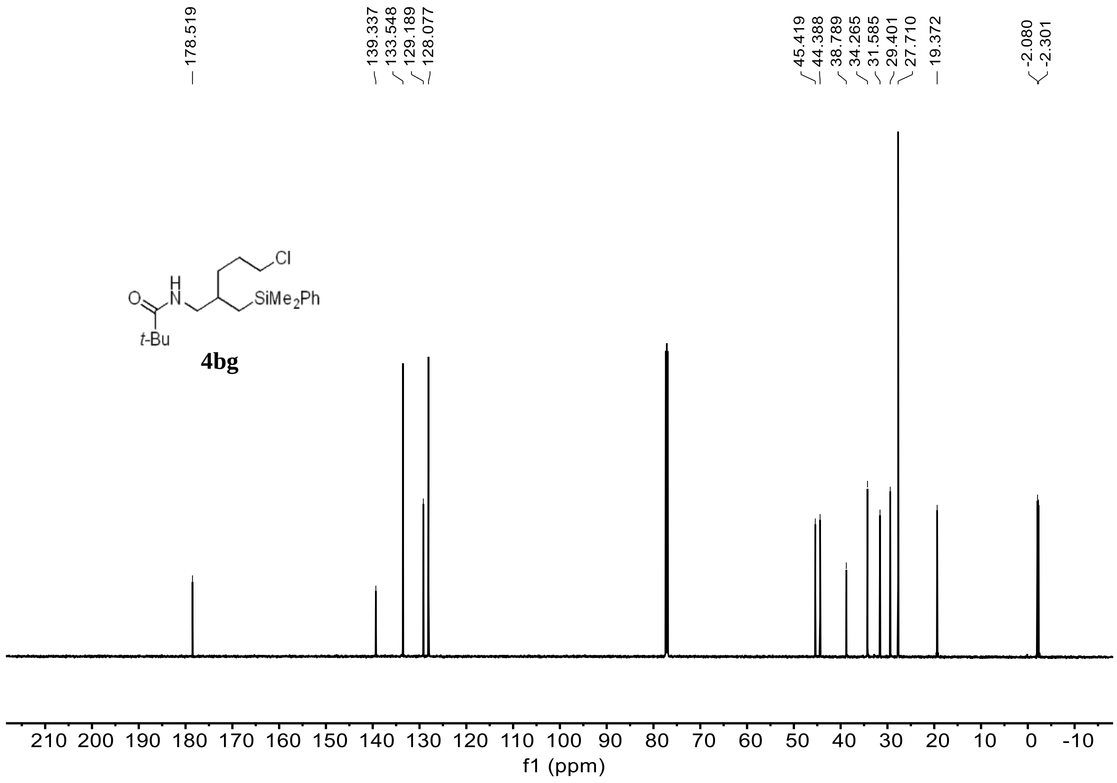

^{13}C NMR Spectrum of **4bg**

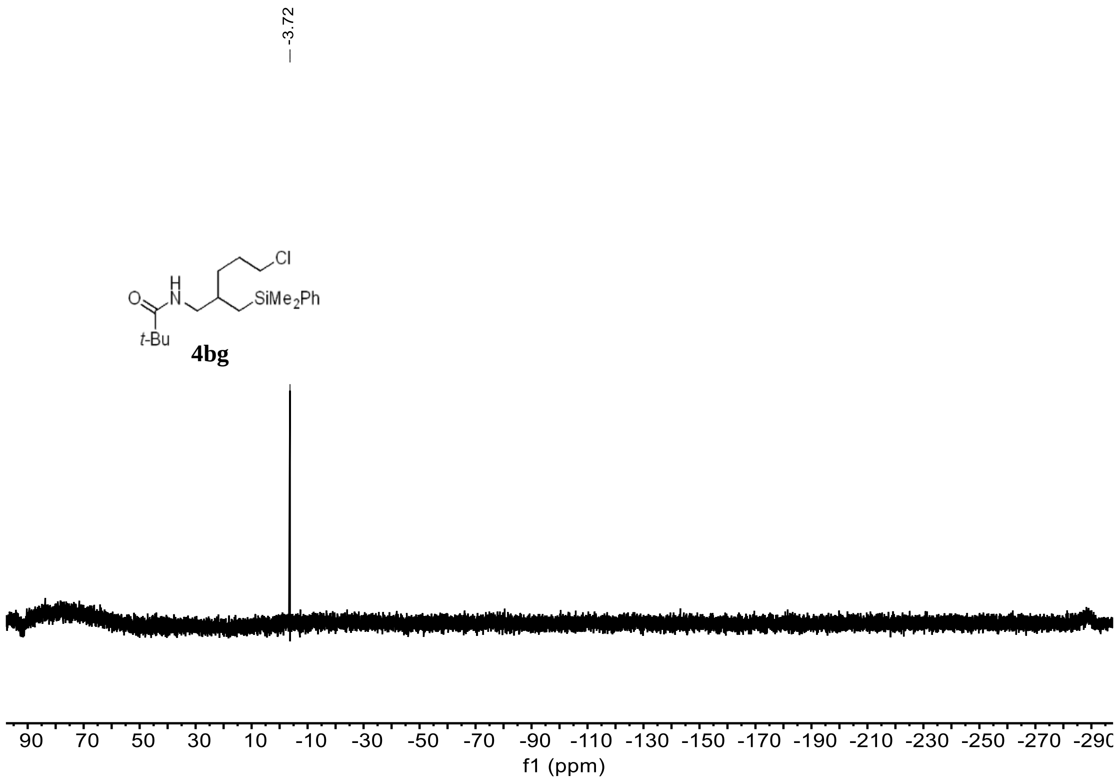

^{13}C NMR Spectrum of **4bg**

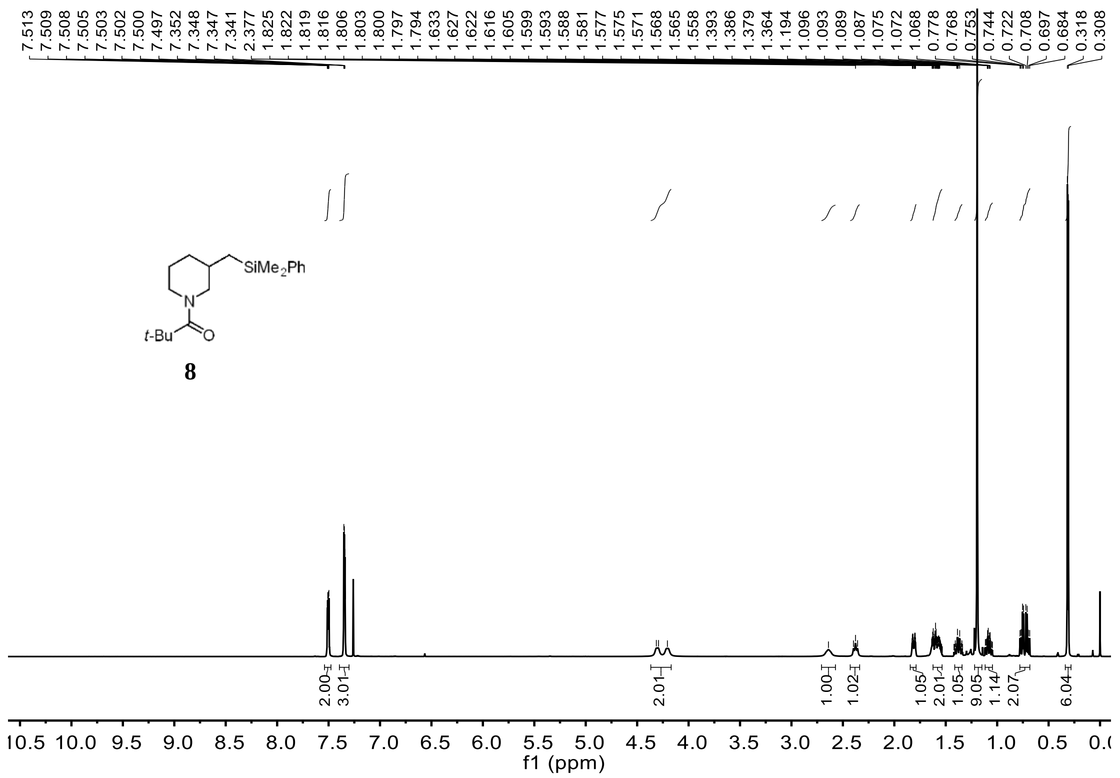

^{1}H NMR Spectrum of **8**

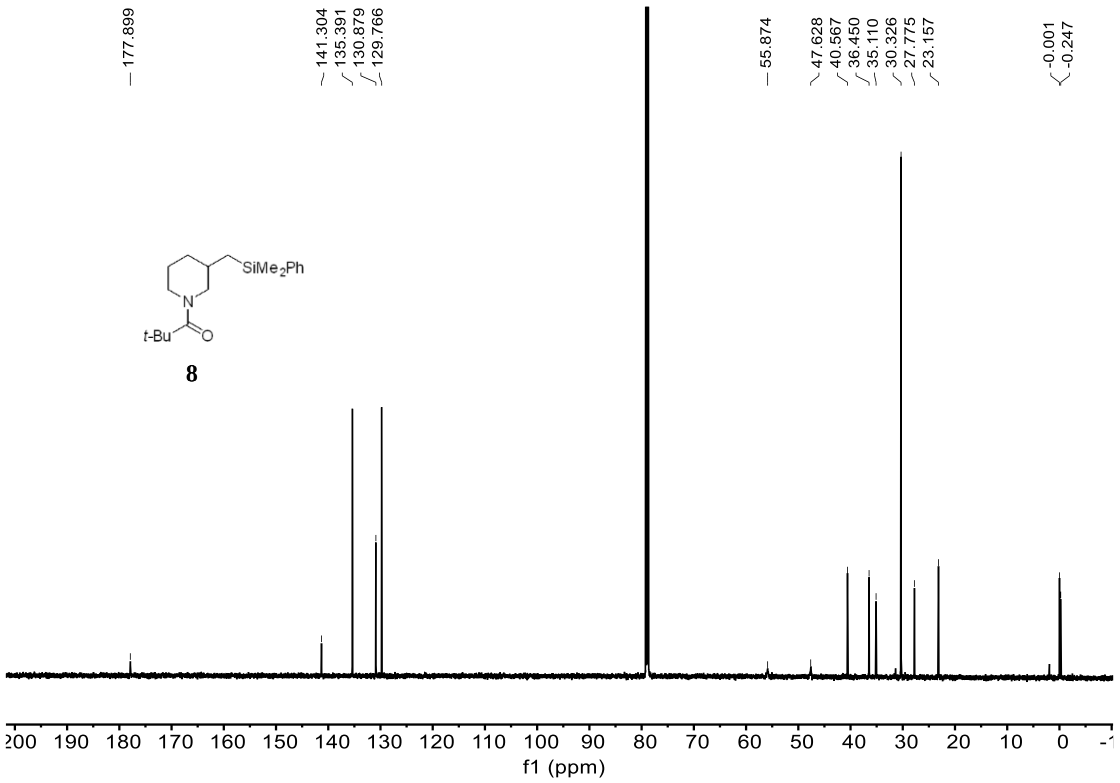

^{13}C NMR Spectrum of **8**

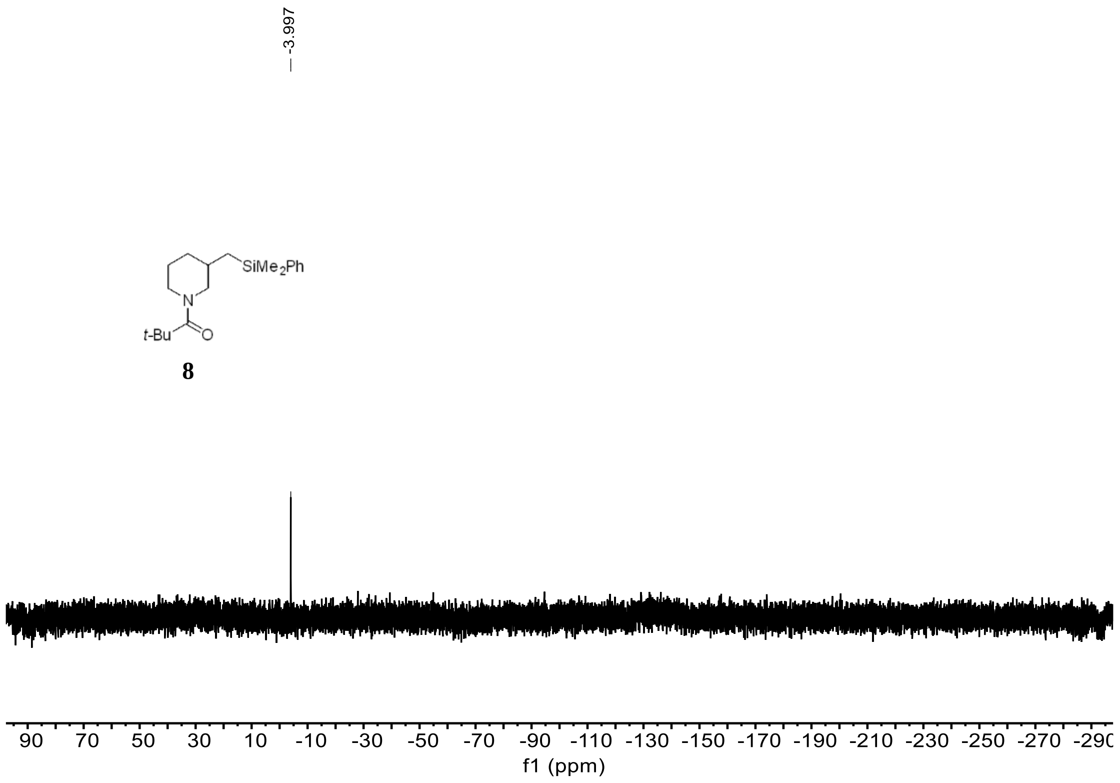

^{29}Si NMR Spectrum of **8**

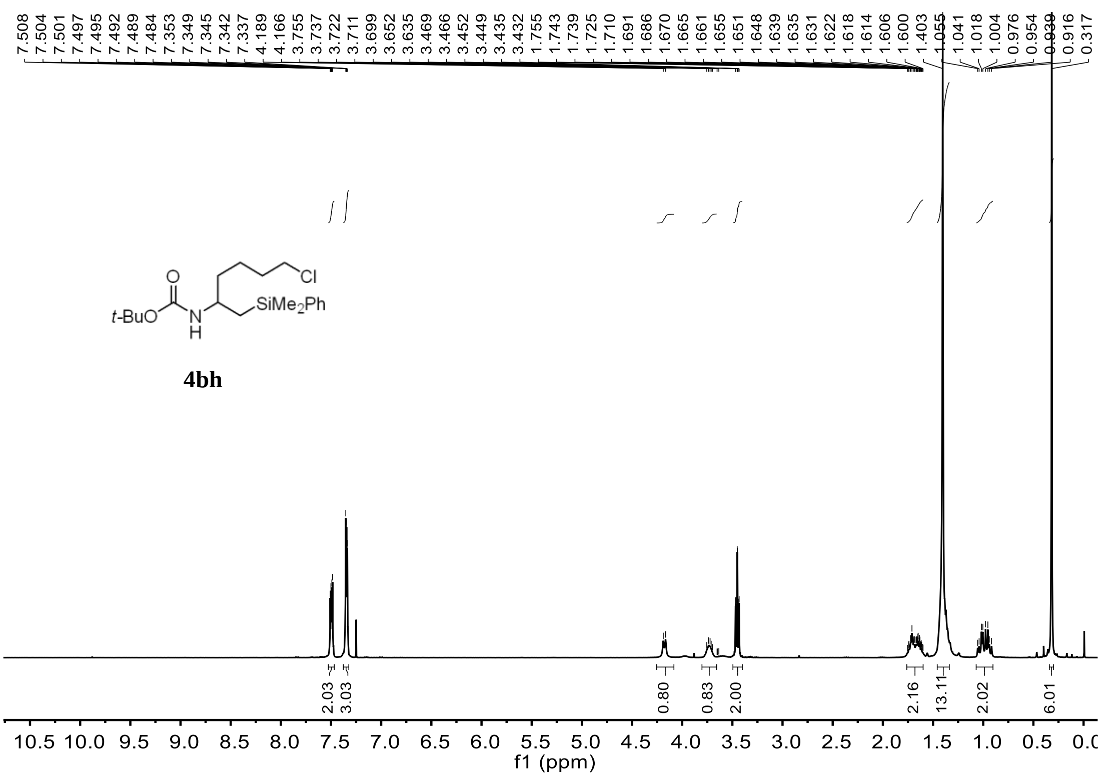

^{1}H NMR Spectrum of **4bh**

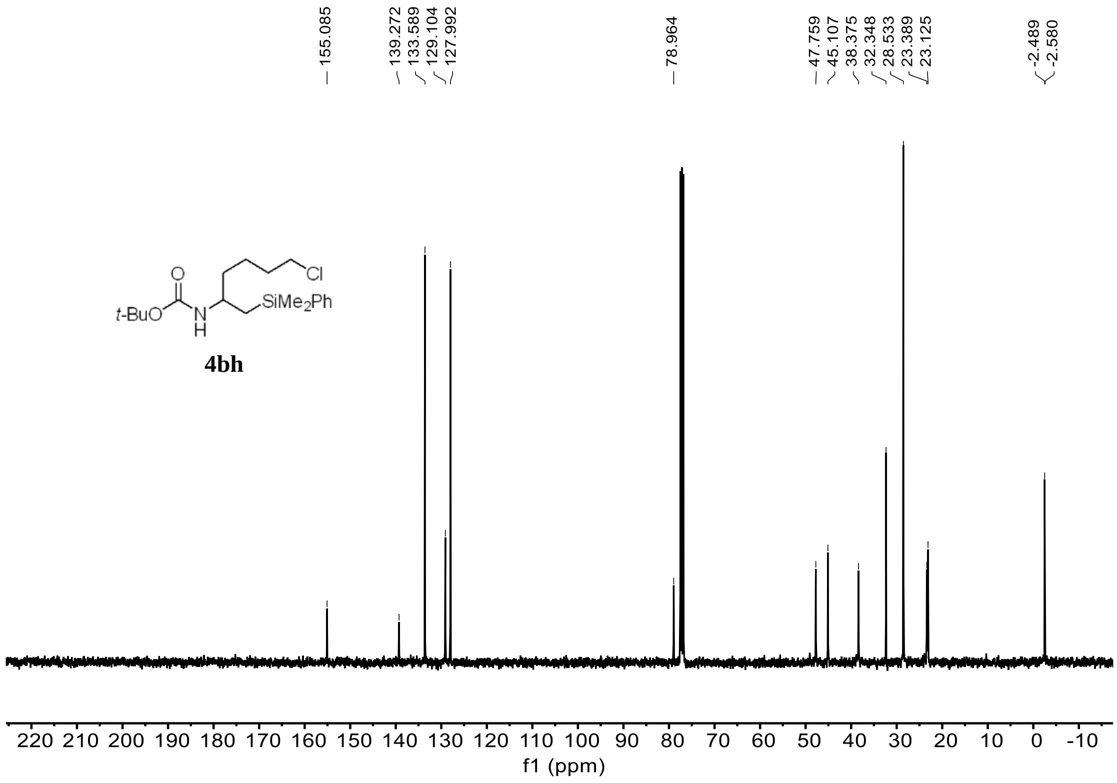

4bh

^{13}C NMR Spectrum of **4bh**

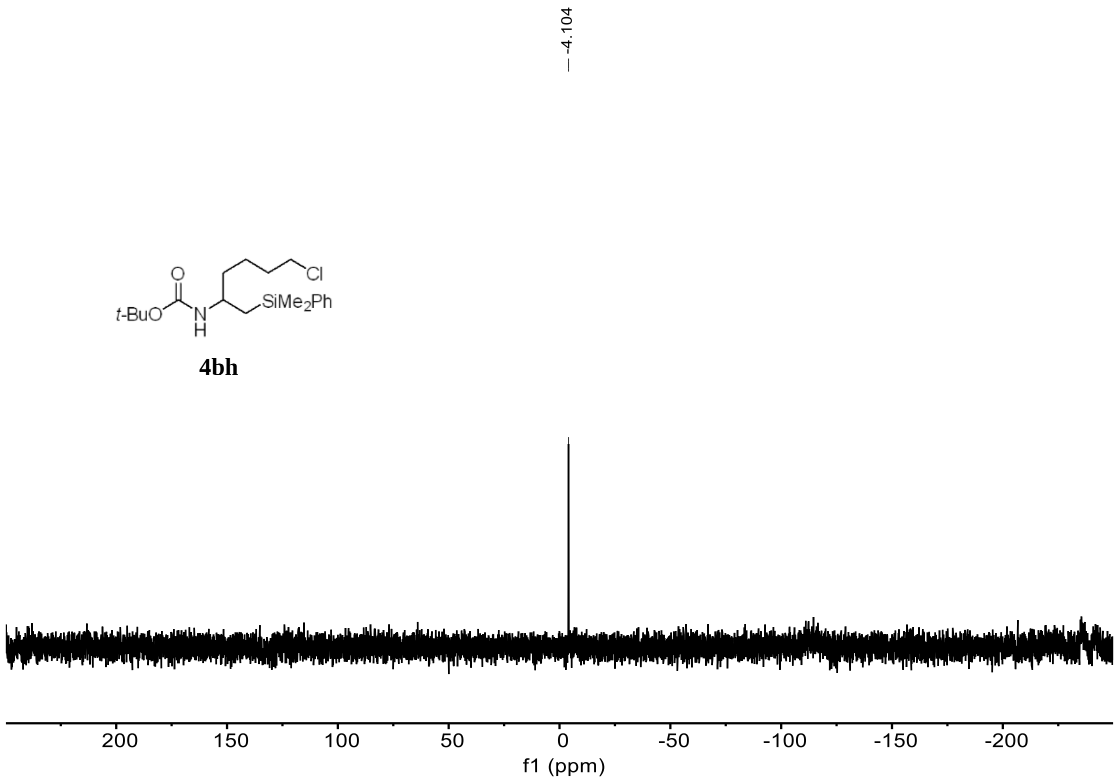

4bh

^{29}Si NMR Spectrum of **4bh**

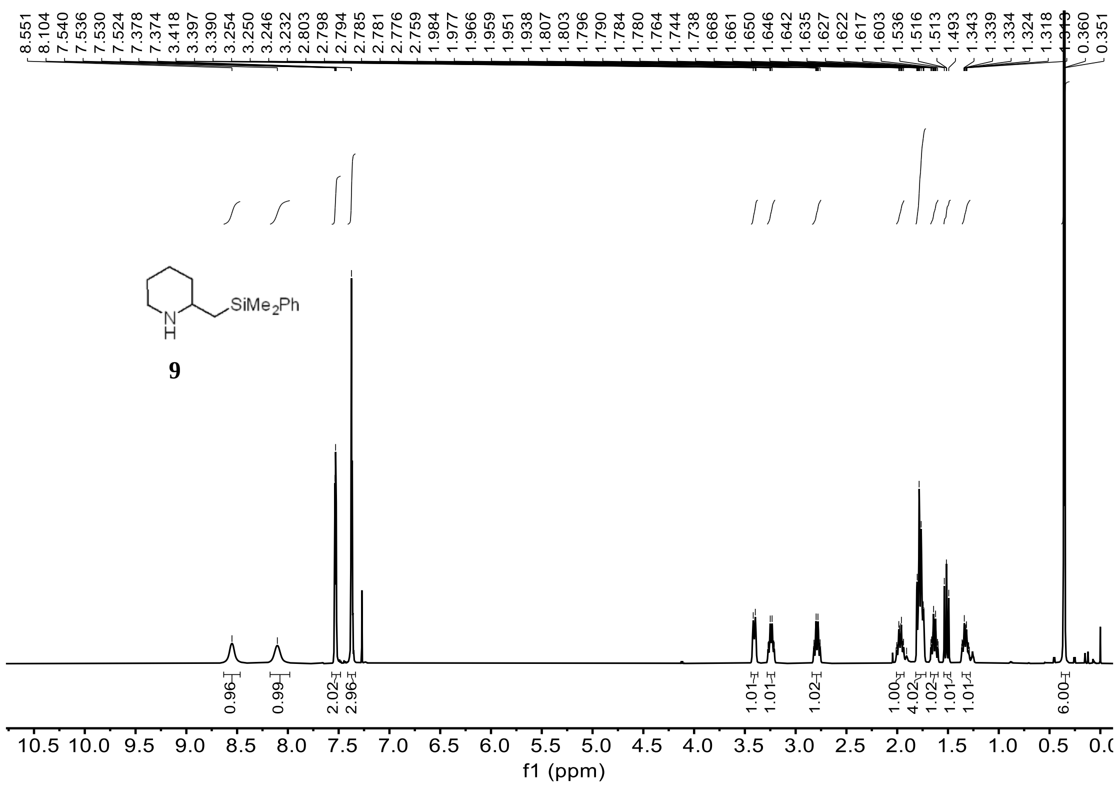

^{1}H NMR Spectrum of **9**

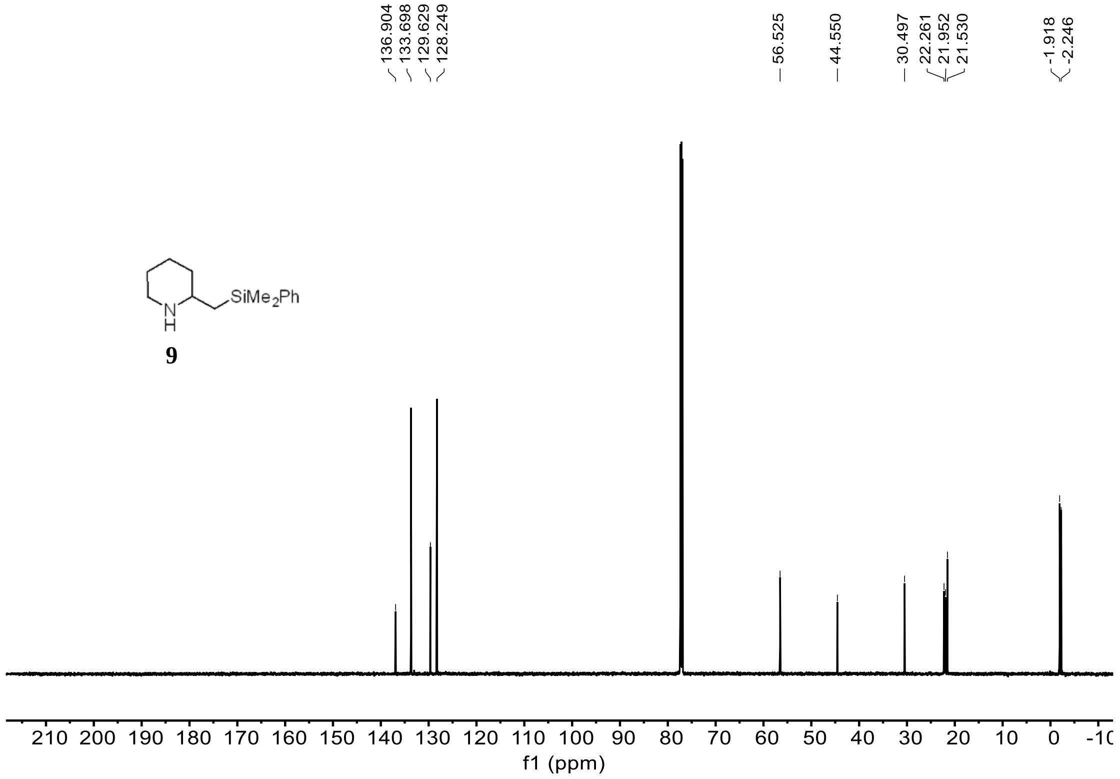

^{13}C NMR Spectrum of **9**

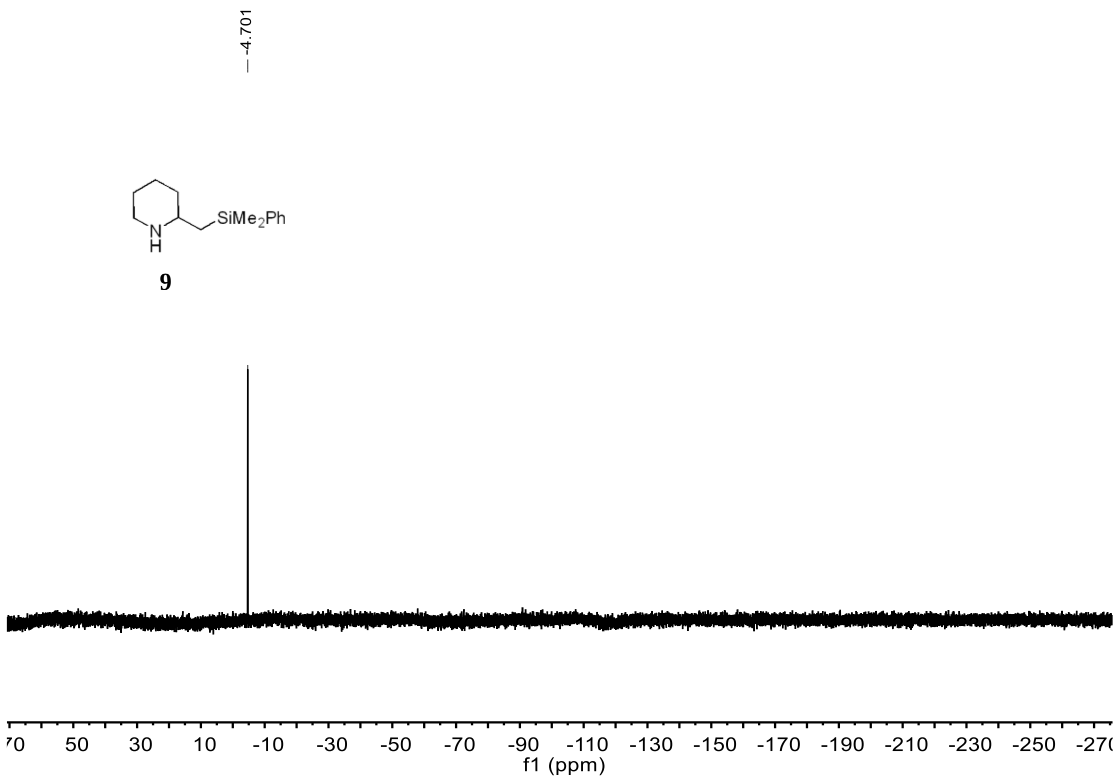

^{29}Si NMR Spectrum of **9**

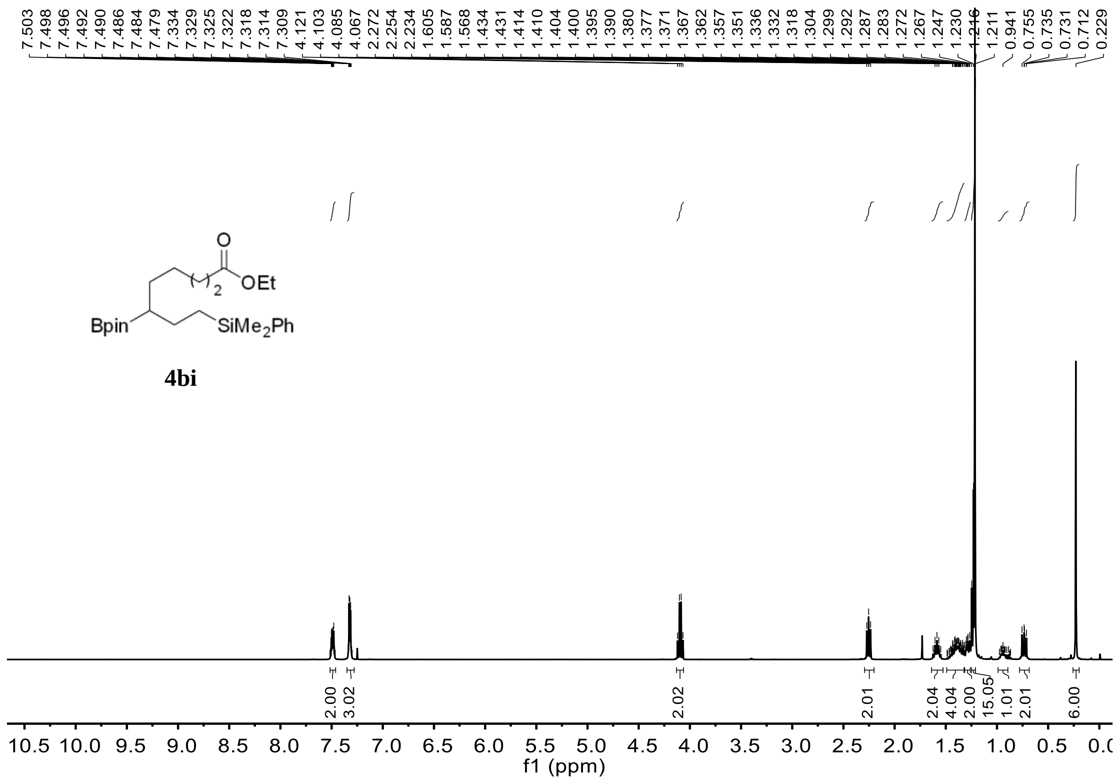

^{1}H NMR Spectrum of **4bi**

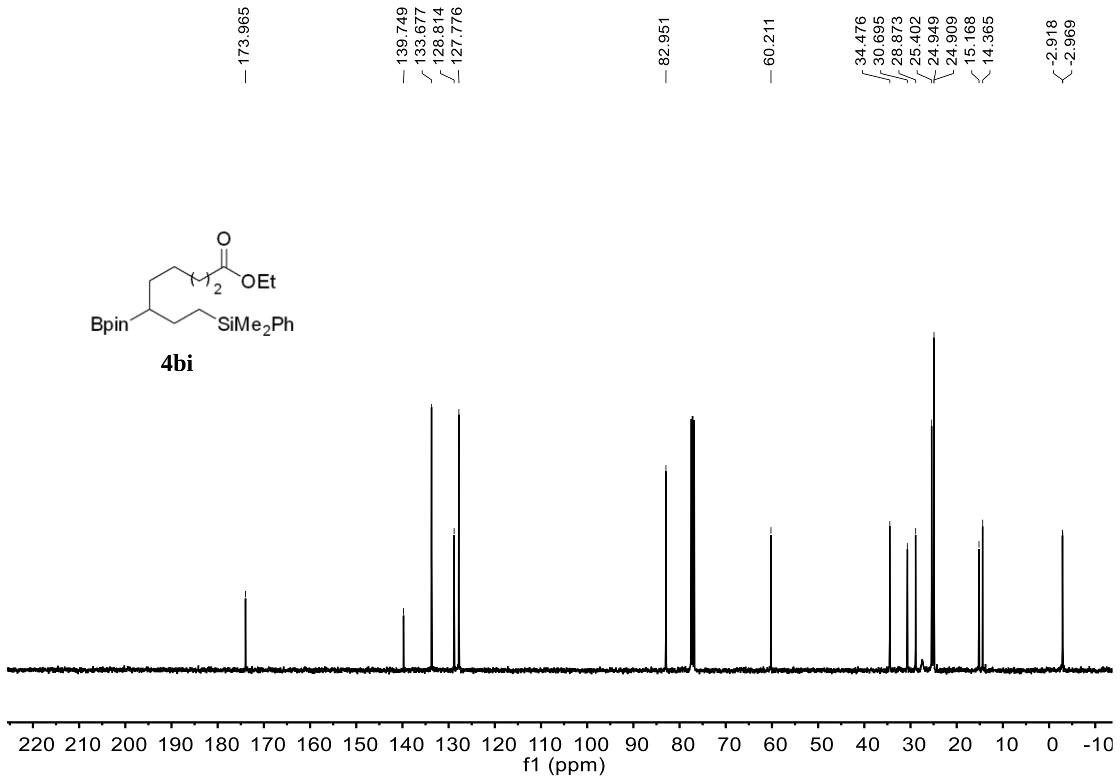

^{13}C NMR Spectrum of **4bi**

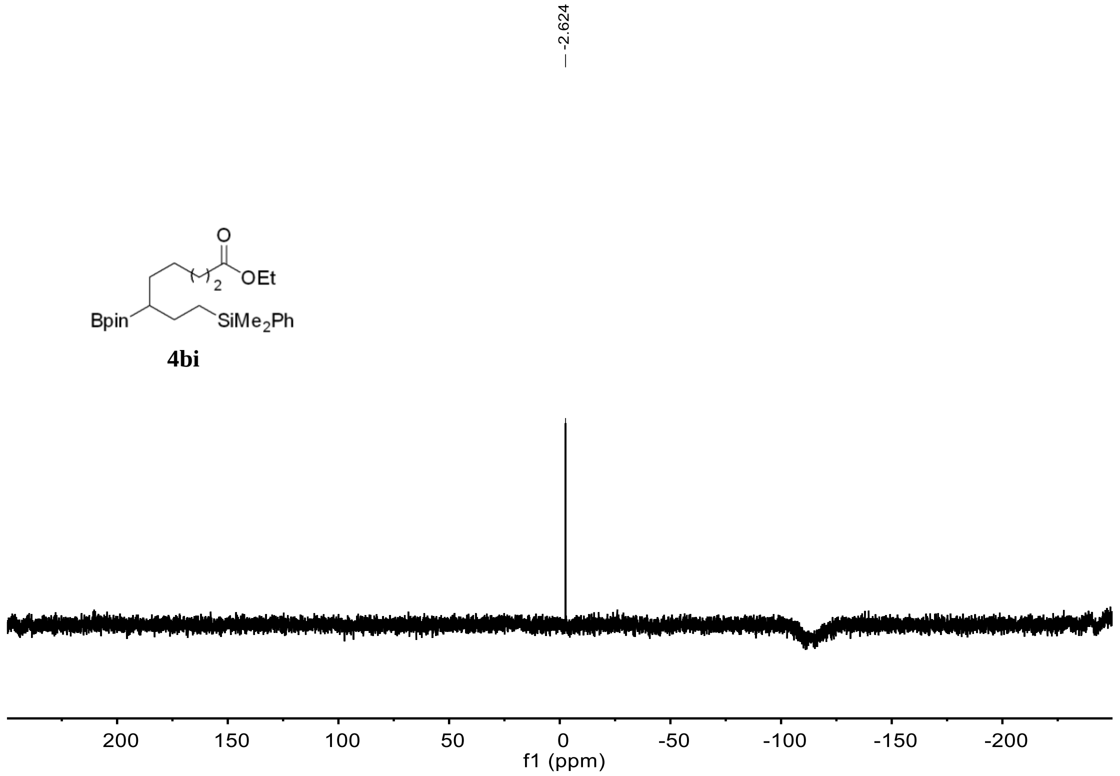

^{29}Si NMR Spectrum of **4bi**

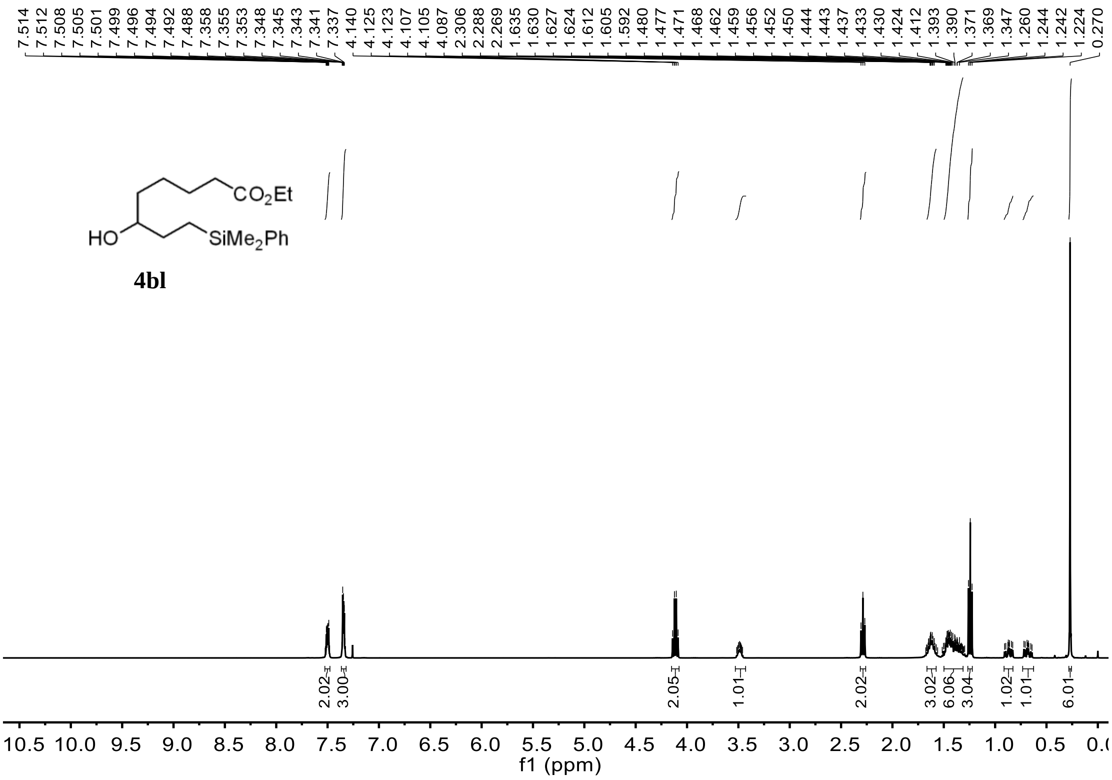

^{1}H NMR Spectrum of **4bl**

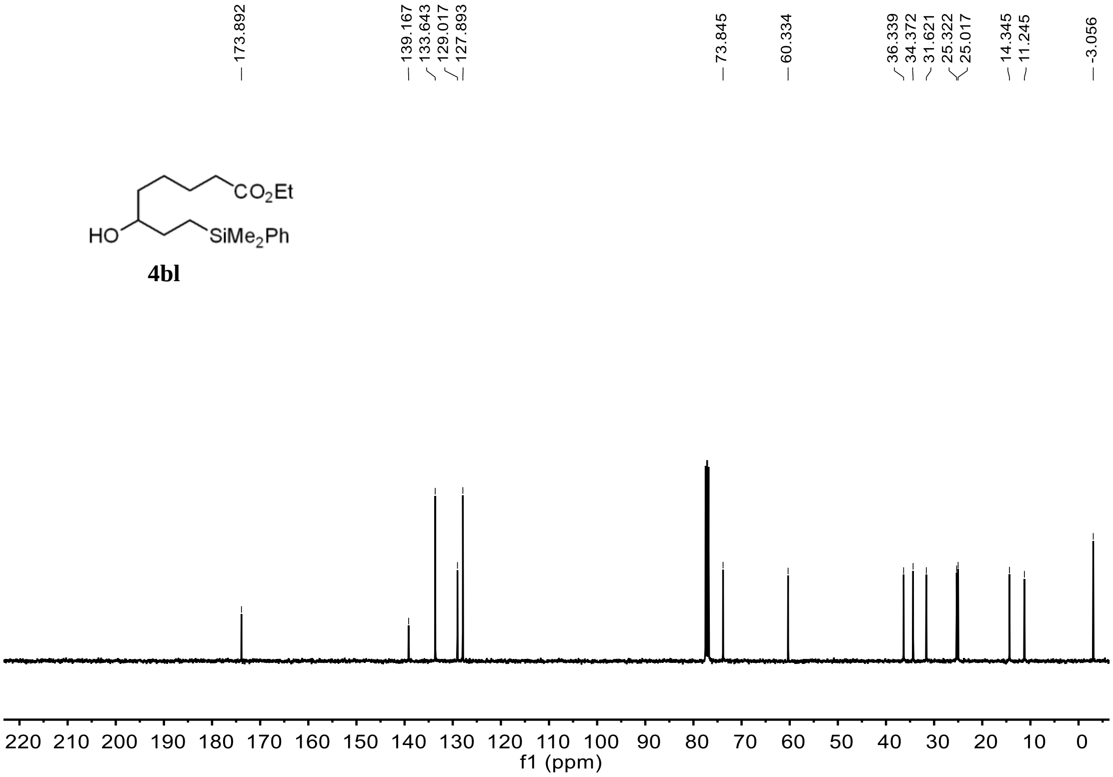

^{13}C NMR Spectrum of **4bl**

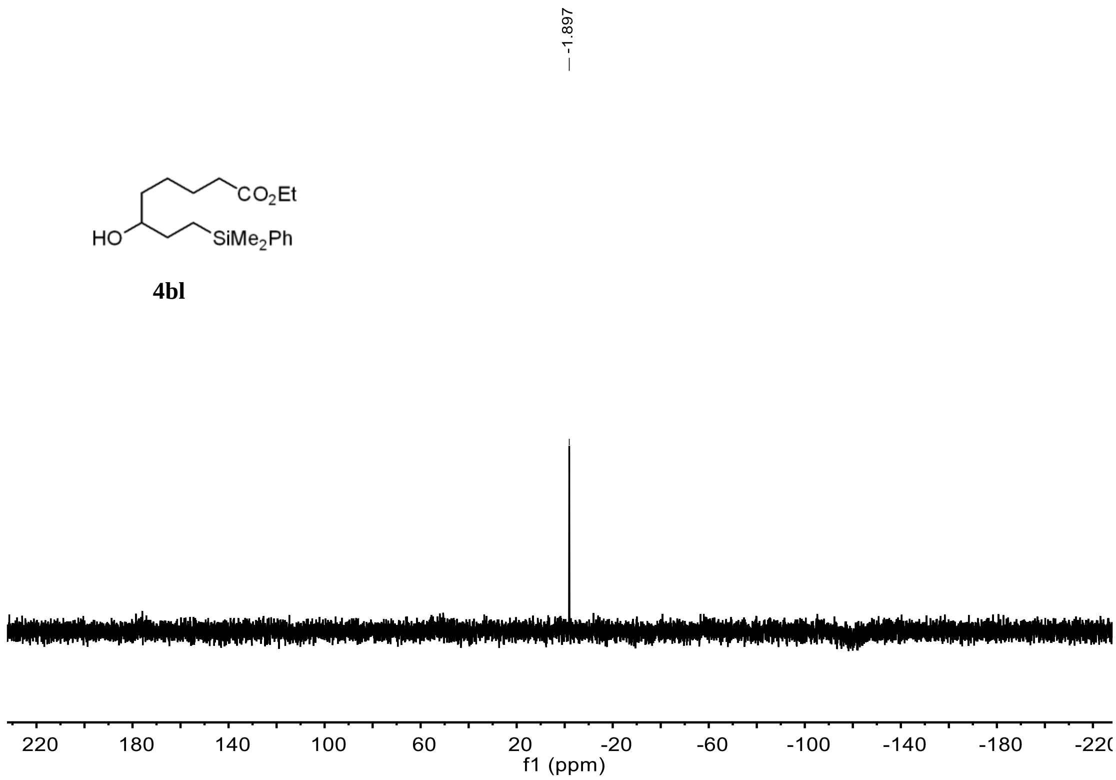

^{29}Si NMR Spectrum of **4bl**

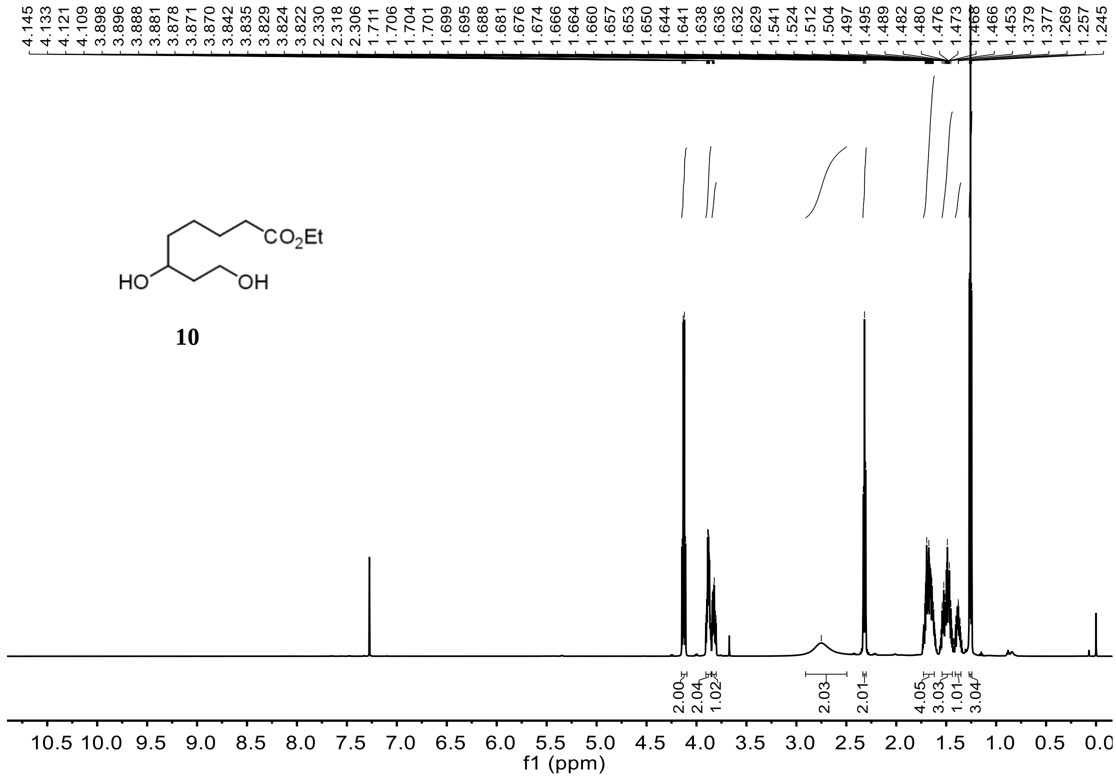

^{1}H NMR Spectrum of **10**

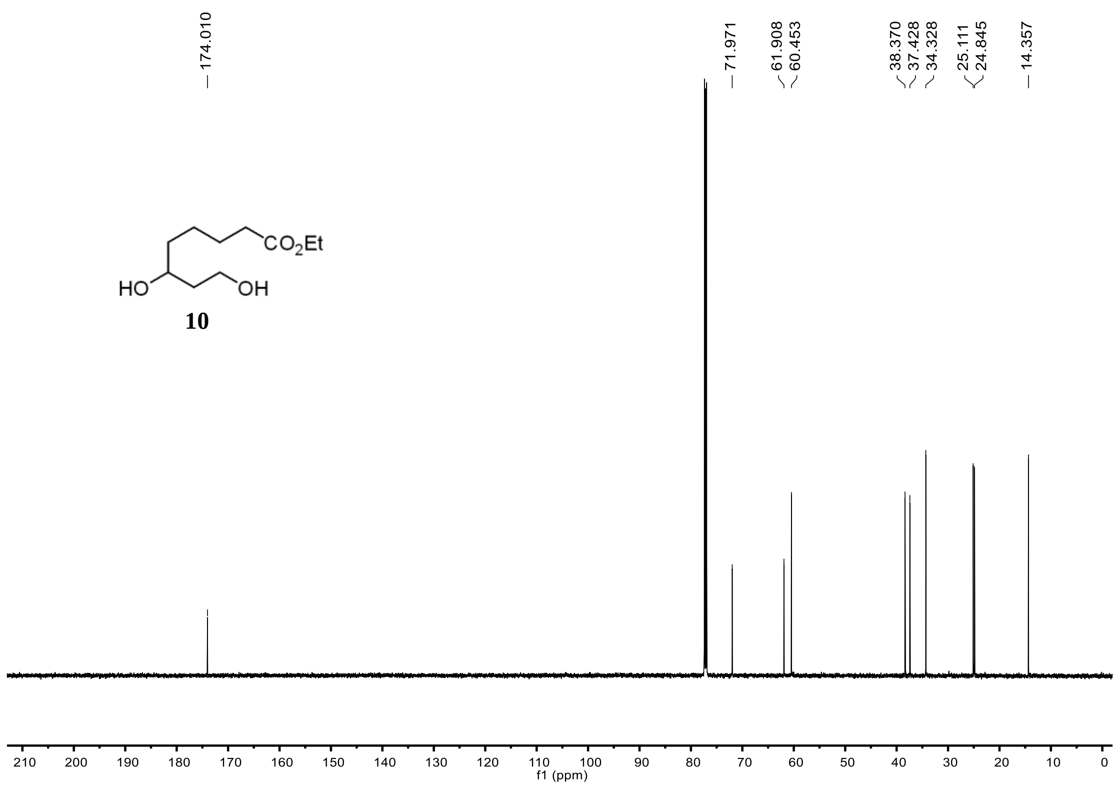

^{13}C NMR Spectrum of **10**

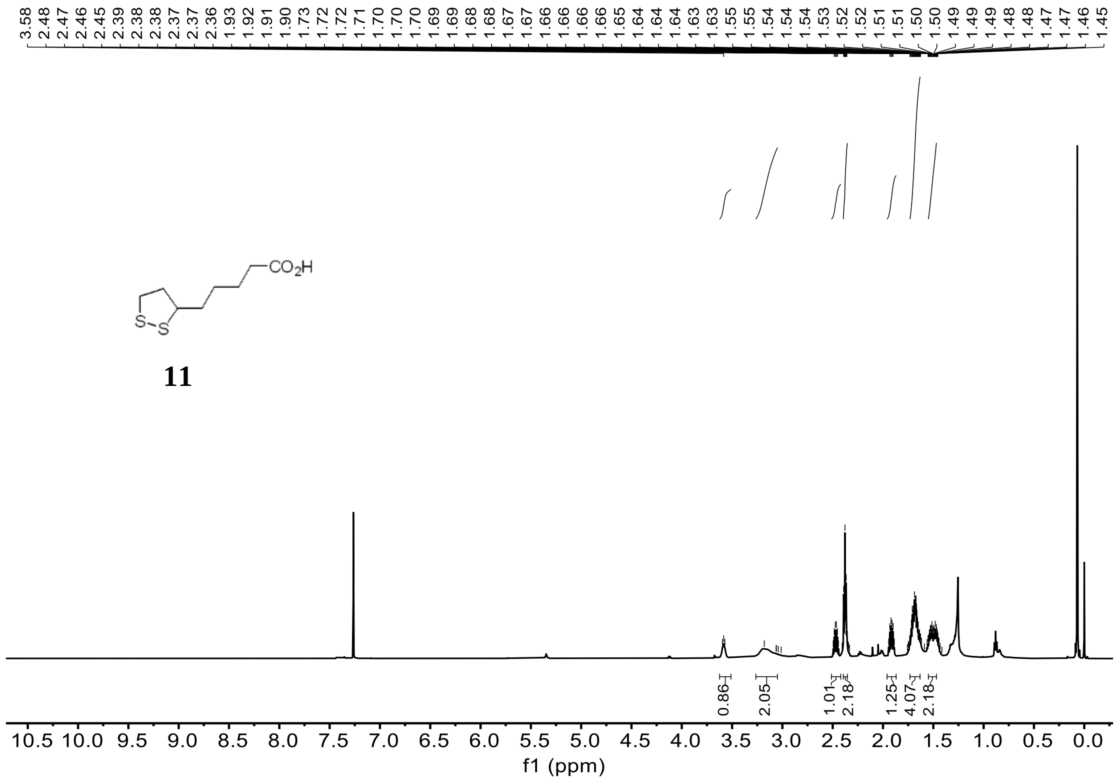

^{1}H NMR Spectrum of **11**

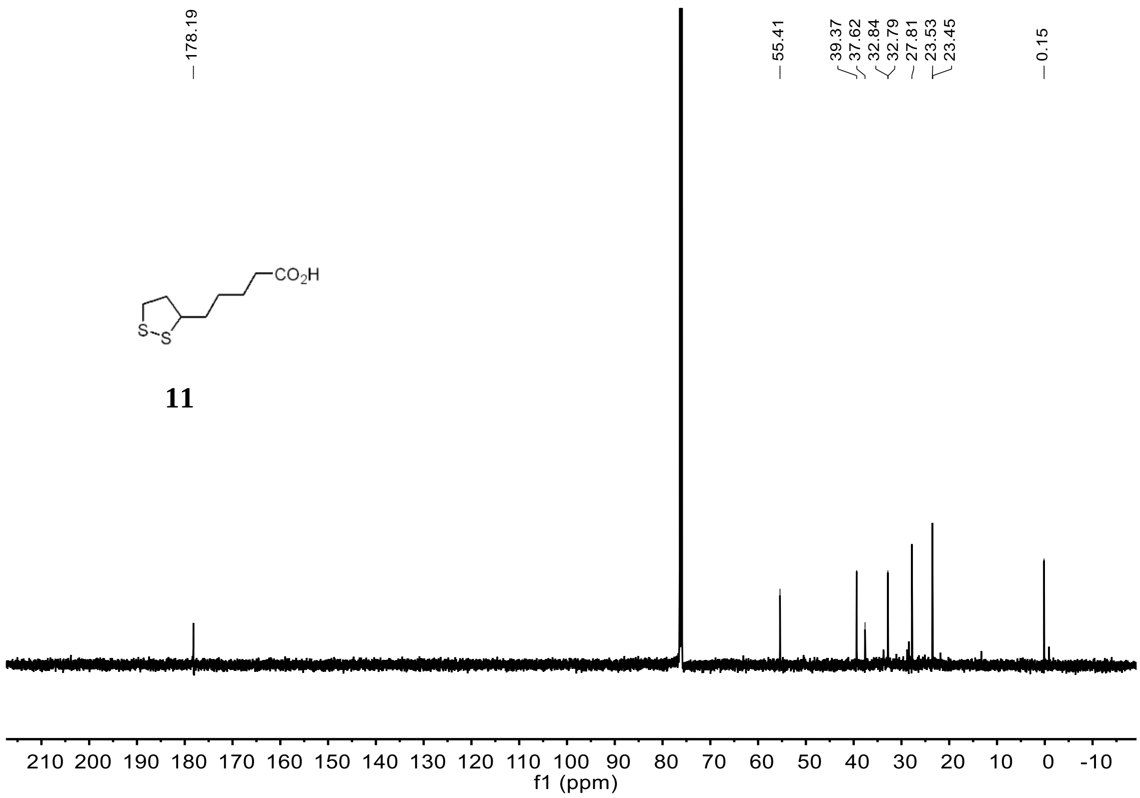

^{13}C NMR Spectrum of **11**

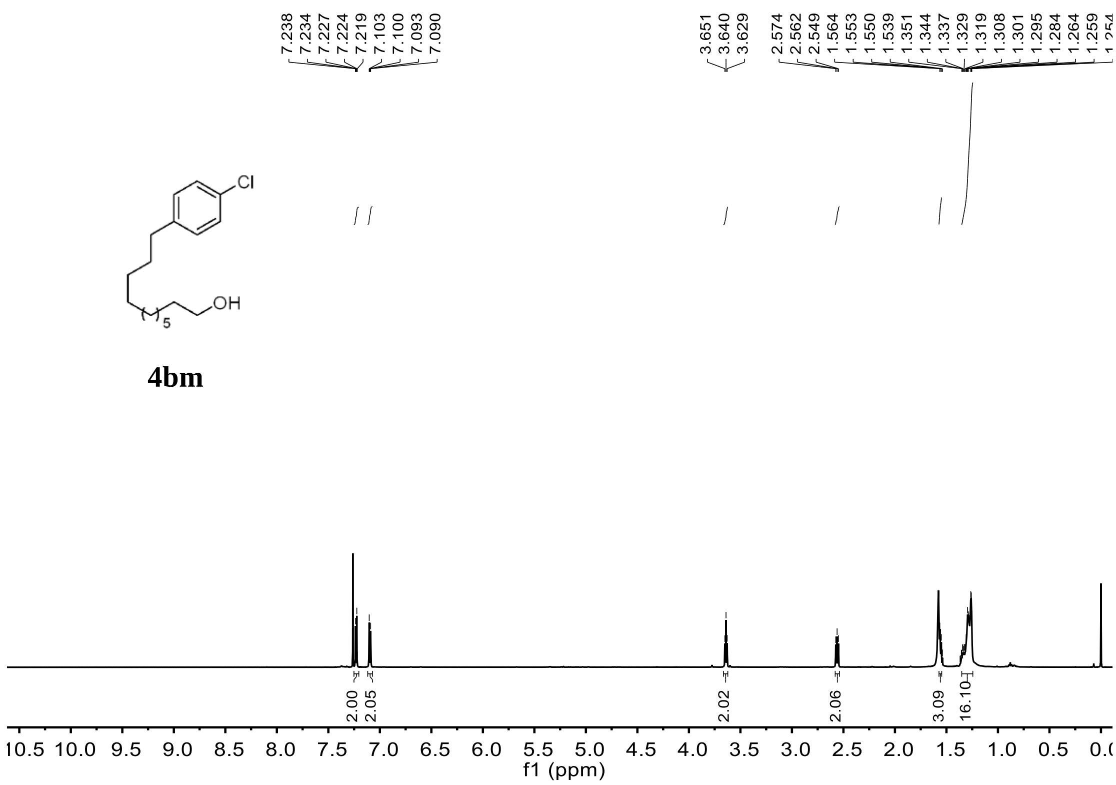

^{1}H NMR Spectrum of **4bm**

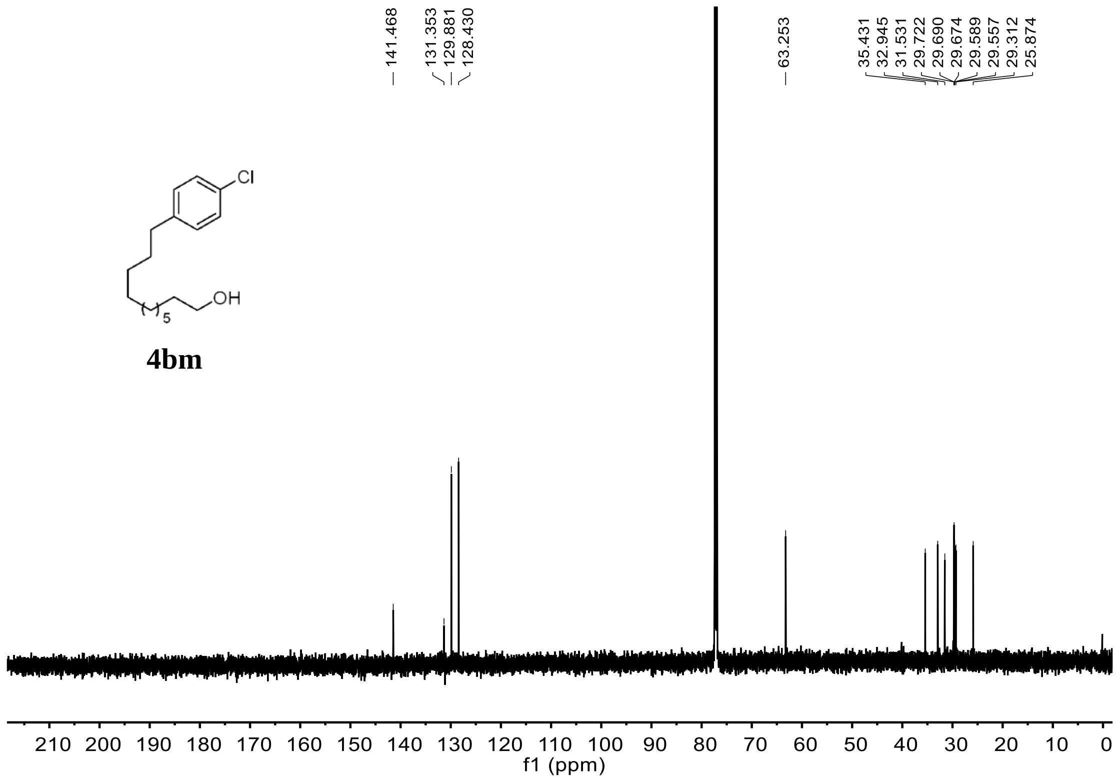

^{13}C NMR Spectrum of **4bm**

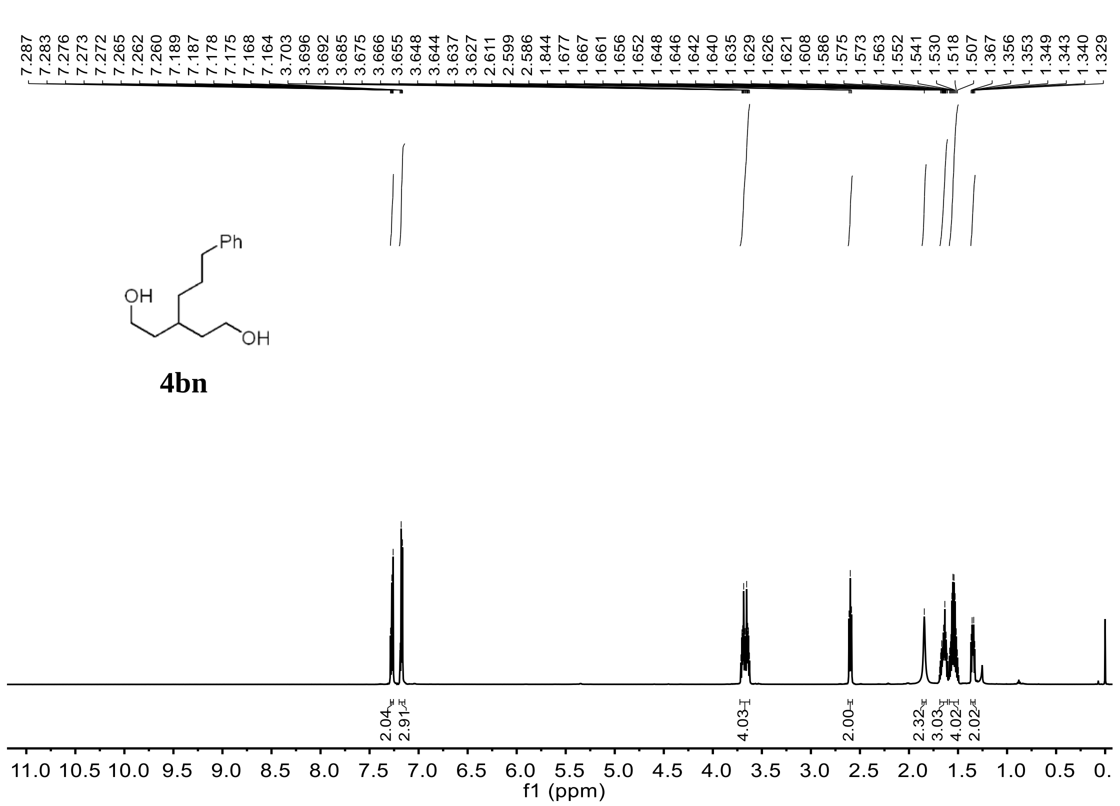

^{1}H NMR Spectrum of **4bn**

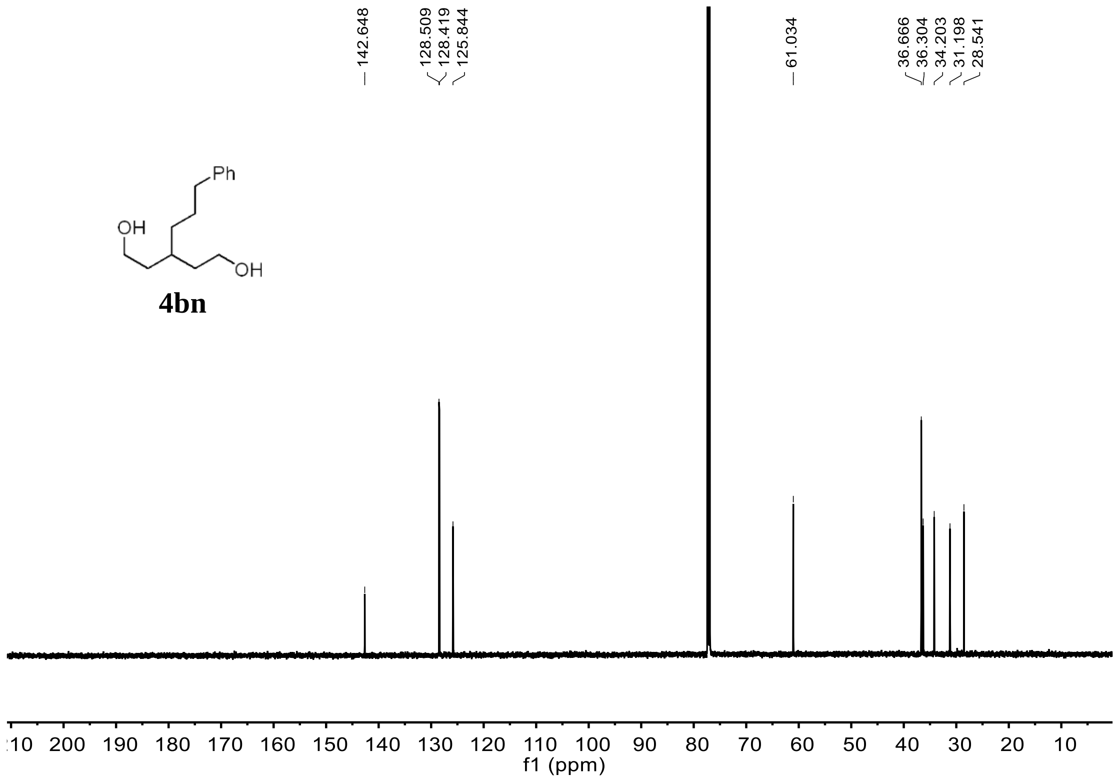

^{13}C NMR Spectrum of **4bn**

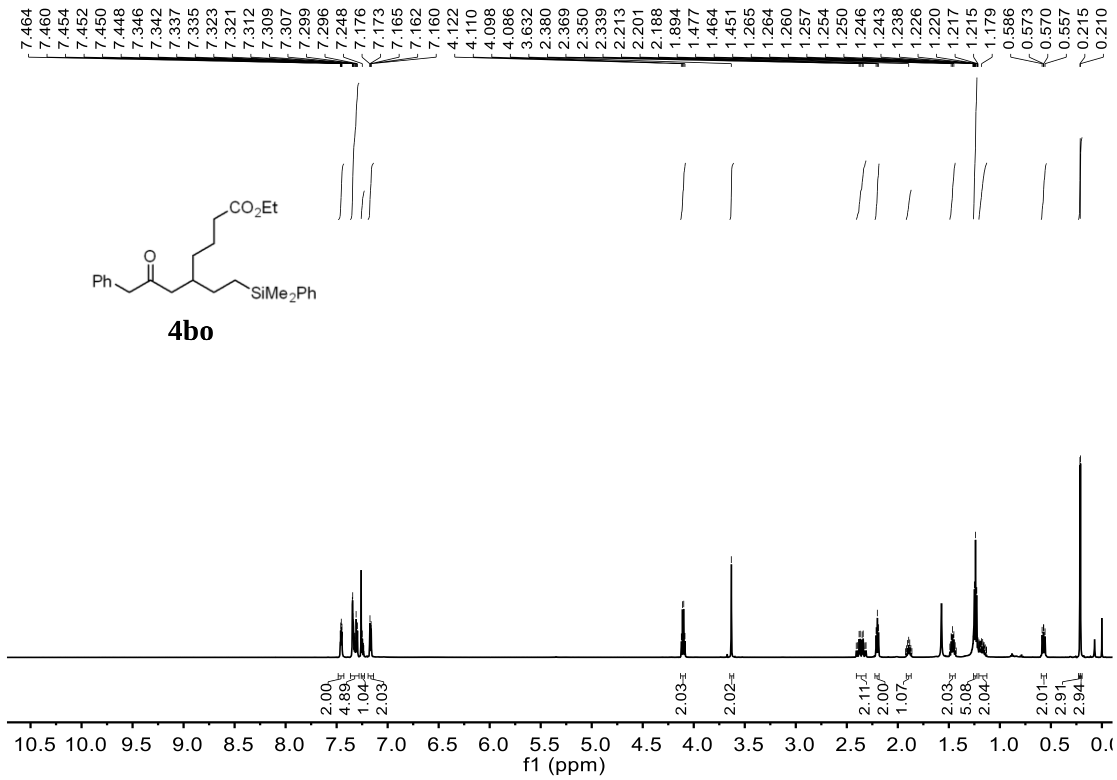

^{1}H NMR Spectrum of **4bo**

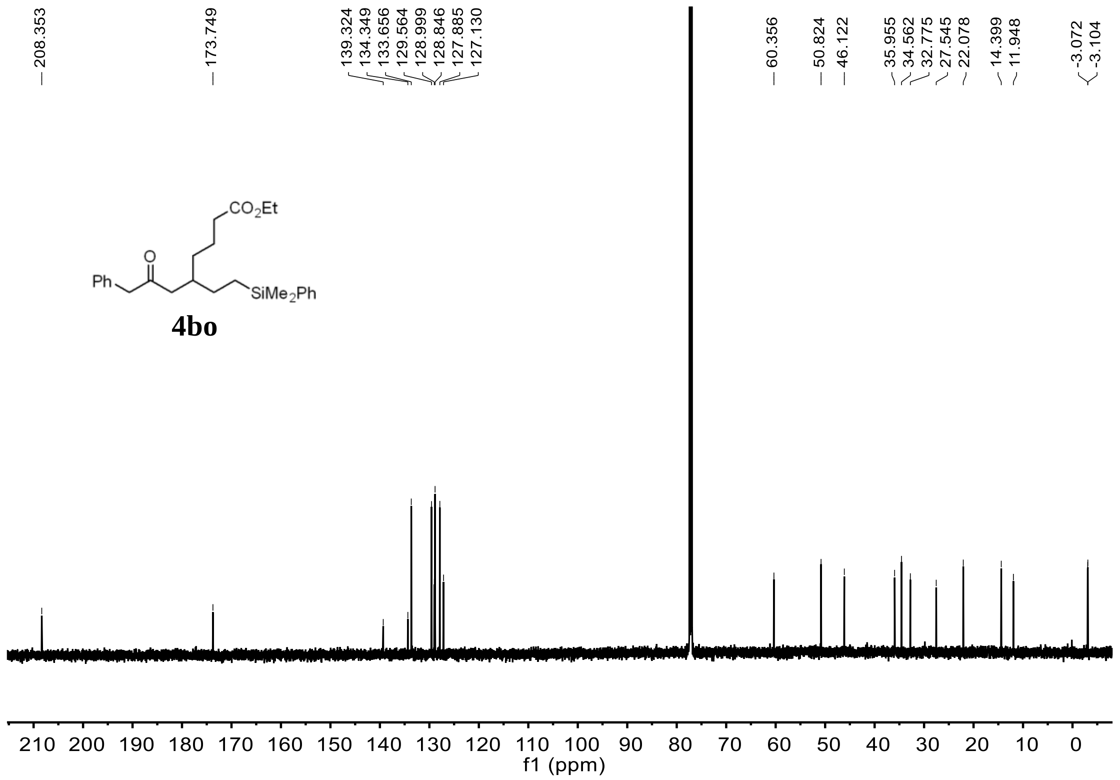

^{13}C NMR Spectrum of **4bo**

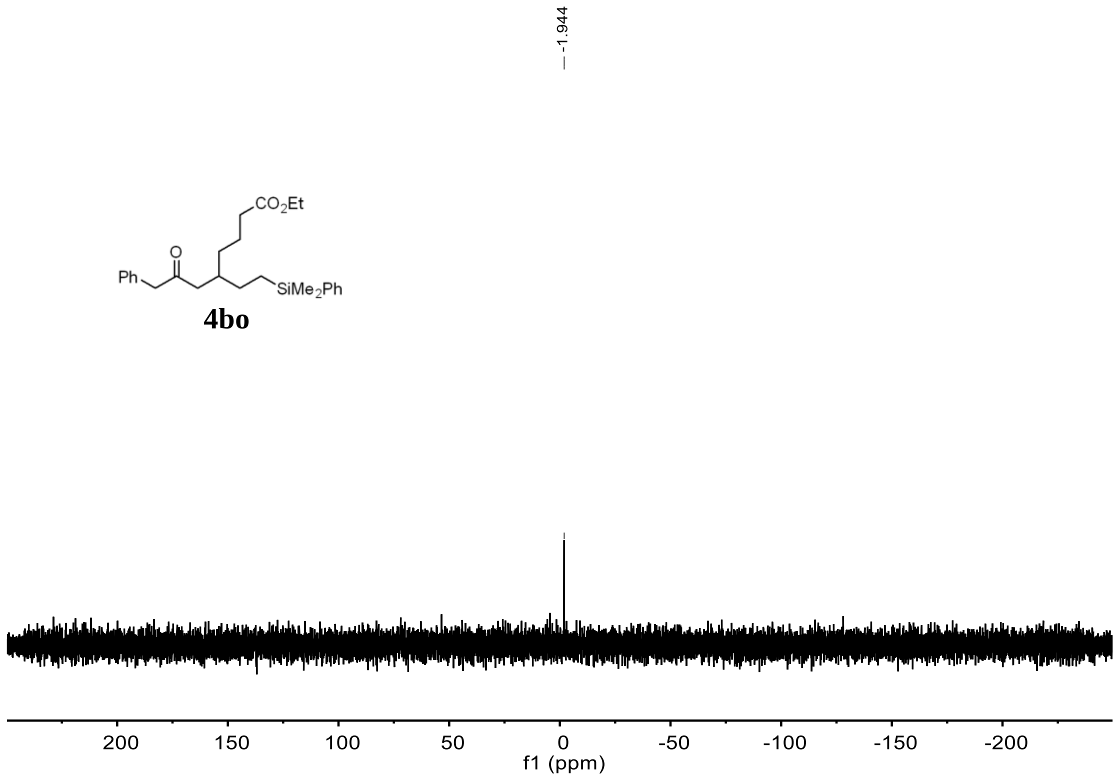

^{29}Si NMR Spectrum of **4bo**

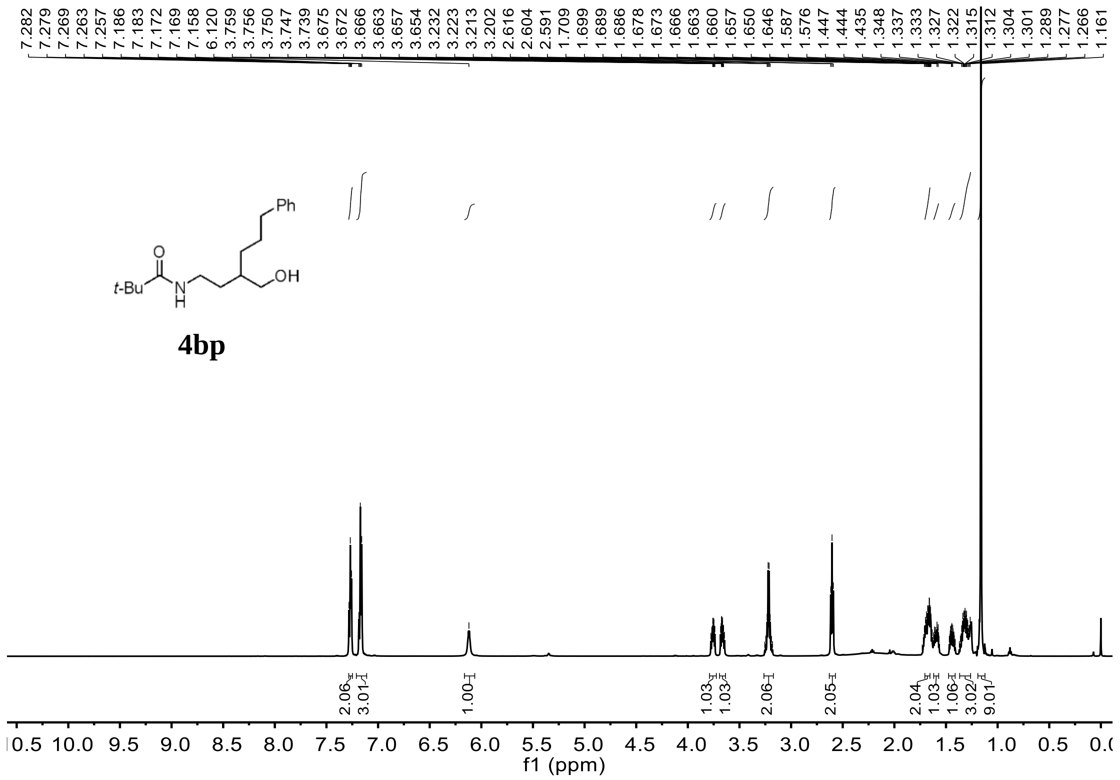

^{1}H NMR Spectrum of **4bp**

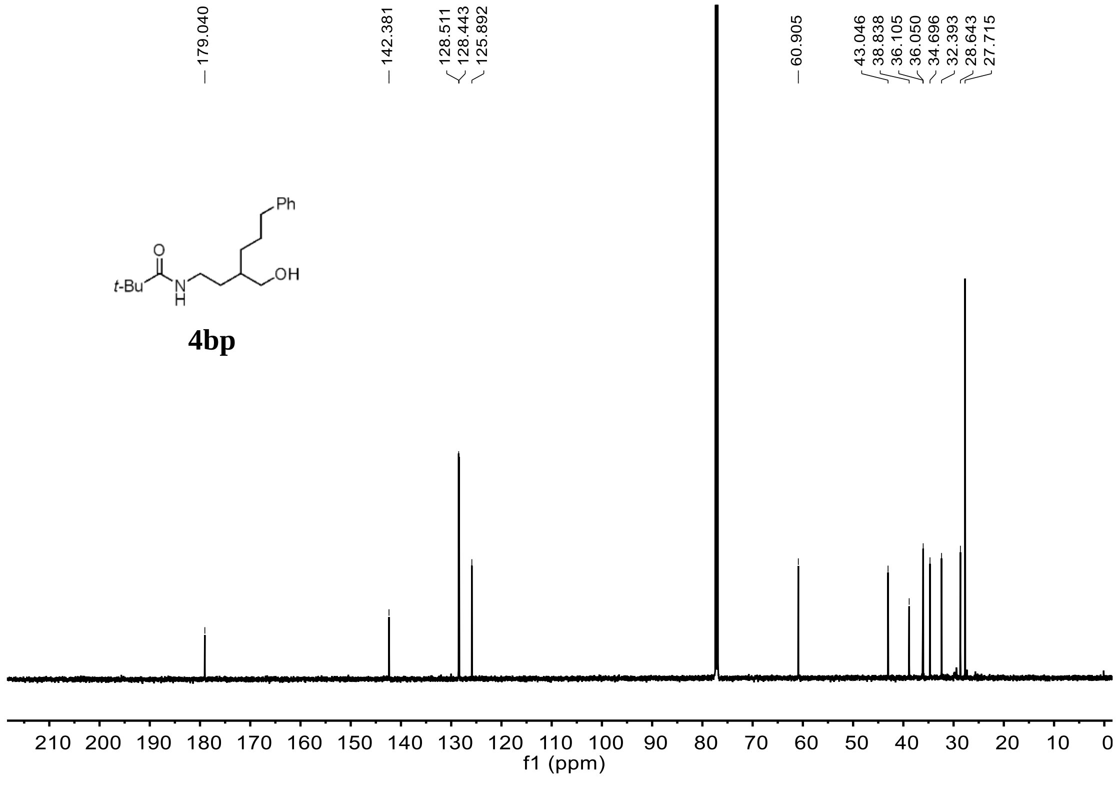

^{13}C NMR Spectrum of **4bp**

2. Supplementary References

[1] C. Nativi, N. Ravidá, A. Ricci, G. Seconi, M. Taddei, *J. Org. Chem.* **1991**, *56*,1951-1955.

[2] X. Zuo, S. Guo, R. Yang, J. Xie, Q. Zhou, *Org. Lett*. **2017**, *19*, 5240-5243.

[3] Y. Wang, H. Mii, T. Kano, K. Maruoka, *Bio. Med. Chem. Lett*. **2009**, *19*, 3795-3797.

[4] J. Wu, L. Guo, A. Noble, V. K. Aggarwal, *Angew. Chem. Int. Ed.* **2019**, *58*, 18830-18834.

[5] D. Ni, M. Kevin Brown, *ACS Catal*. **2021**, *11*, 1858-1862.

[6] K. P. Pawar, C. Praveen, N. B. Patil, S. P. Chavan, *Tetrahedron* **2015**, *71*, 4213-4218.

[7] J. Blom, A. Vidal-Albalat, J. Jørgensen, C. L. Barløse, K. S. Jessen, M. V. Iversen, K. A. Jørgensen, *Angew. Chem. Int. Ed*. **2017**, *56*, 11831-11835.
